# Effects of branched-chain amino acids on iron deficiency-induced muscle atrophy

**DOI:** 10.1016/j.bbrep.2026.102451

**Published:** 2026-01-17

**Authors:** Miki Kawanaka, Mingyuan Wang, Toru Iwahashi, Mai Konishi, Katsuyuki Konishi, Seira Sato, Hiroyuki Tanaka, Ken Nakata

**Affiliations:** a Department of Sports Medical Science, Graduate School of Medicine, The University of Osaka, Osaka, 565-0871, Japan; b Department of Medicine for Sports and Performing Arts, Graduate School of Medicine, The University of Osaka, Osaka, 565-0871, Japan; c Department of Orthopaedic Surgery, Graduate School of Medicine, The University of Osaka, Osaka, 565-0871, Japan

**Keywords:** Iron deficiency, Branched-chain amino acids, Muscle atrophy, Muscle protein synthesis, Atrogenes

## Abstract

Iron deficiency (ID) is a potential contributor to skeletal muscle atrophy through disruption of the balance between protein synthesis and degradation. This muscle loss is associated with sarcopenia and locomotive syndrome, conditions that impair mobility and reduce healthy life expectancy. While branched-chain amino acids (BCAA) are known to attenuate dexamethasone-induced muscle atrophy, its effectiveness against ID-induced atrophy has not been fully elucidated. This study aims to investigate the effects of BCAA on ID-induced muscle atrophy in C2C12 myotubes treated with the iron chelator deferoxamine (DFO). Results showed that DFO significantly reduced myotube diameter and upregulated atrogenes such as Atrogin-1 and MuRF-1, accompanied by increased p-AMPK and p-eEF2, and decreased p-Akt levels. BCAA supplementation partially suppressed Atrogin-1 expression but had no effect on MuRF-1 or myotube diameter. Additionally, p-p70S6K was significantly upregulated in the BCAA + DFO group, while p-eEF2 levels remained elevated, similar to the DFO group. These findings suggest that ID may activate alternative catabolic signaling, such as the NF-kB pathway, thereby counteracting the anabolic effects of BCAA via the Akt signaling pathway. Thus, BCAA has limited efficacy in preventing muscle atrophy under iron-deficient conditions. In conclusion, BCAA may partially promote muscle protein synthesis-related signaling pathways, but is insufficient to prevent muscle atrophy induced by ID.

## Highlights

• Iron deficiency leads to reduced myotube diameter and upregulation of atrogenes.
• BCAA suppressed *Atrogin-1* expression but had no effect on MuRF-1 or myotube diameter.
• BCAA has limited efficacy in preventing muscle atrophy under iron-deficient conditions.

## 1 Background

Non-communicable diseases (NCDs), including cardiovascular diseases, cancer, and diabetes, are chronic diseases that pose a significant global health challenge, accounting for 74 % of non-pandemic-related deaths worldwide [1]. In Japan, as the population continues to age and life expectancy increases, there remains a gap of approximately 10 years between total life expectancy and healthy life expectancy—the period during which individuals maintain good physical and mental health [2]. Lifestyle factors play a significant role in the development of many NCDs, which can be effectively prevented through physical activity and proper nutritional management [3]. Therefore, extending healthy life expectancy through these interventions is a critical medical and social issue.

Skeletal muscle, the largest organ and protein reservoir in the human body, accounting for approximately 40 % of total body weight, plays a crucial role in locomotion, respiration, and food intake [4,5]. In older adults, a decline in skeletal muscle mass further raises the risk of skeletal muscle disorders like sarcopenia, which is associated with the development of locomotive syndrome [6,7]. Furthermore, skeletal muscle disorders contribute to frailty, physical disability, and mortality [7,8], and physical inactivity in turn increases the risk of NCDs. Therefore, maintaining skeletal muscle mass plays a crucial role in preventing NCDs and extending healthy life expectancy, highlighting the necessity of early intervention.

When the balance of muscle protein synthesis and degradation, which regulates skeletal muscle mass, is disrupted, a decline in muscle mass occurs, leading to muscle atrophy. Among the various factors contributing to muscle atrophy, such as cancer, infection, diabetes, and physical inactivity, iron deficiency (ID) also plays a significant role in the loss of muscle mass [5,9]. Iron is crucial for various cellular processes, including energy metabolism, nucleotide synthesis, and a wide range of enzymatic reactions [[9], [10], [11], [12]]. It has been reported that ID increases in prevalence with age and affects approximately 24 % of individuals aged 65 years and older [13,14]. Additionally, ID may accelerate the progression of sarcopenia and locomotive syndrome in older adults, potentially reducing healthy life expectancy [9,10]. The common treatment for ID is oral iron supplementation; however, there is a risk of iron overload and concerns about side effects such as damage to the gastrointestinal mucosa [15]. Therefore, it is necessary to explore safe and effective methods to suppress muscle atrophy in the presence of ID.

On the other hand, muscle atrophy is one of the side effects of the corticosteroid drug dexamethasone (DEX), which occurs through the inhibition of muscle protein synthesis and the promotion of muscle degradation [16]. Therefore, DEX is widely used to induce muscle atrophy models in related researches [[16], [17], [18], [19], [20]]. According to previous studies, both *in vitro* and *in vivo* studies have demonstrated that branched-chain amino acids (BCAA) effectively improve DEX-induced muscle atrophy [16,17]. It is well known that amino acids are fundamental components of skeletal muscle proteins and are key factors in maintaining and promoting skeletal muscle growth [21]. As essential nutrients that the body cannot synthesize, BCAA, which refers to the three amino acids—leucine, isoleucine, and valine—must be obtained externally [22], leading to wide application in health foods, supplements, and as potential therapeutic agents for muscle atrophy [4]. According to previous studies, leucine supplementation can mitigate the inhibitory effects of adrenal glucocorticoids on protein synthesis via the mTOR and AMPK pathways [16]. In addition, it has been reported that BCAA supplementation prevents DEX-induced soleus muscle atrophy by suppressing both the ubiquitin-proteasome and autophagy-lysosomal protein degradation pathways [17]. However, there is limited research on the effects of BCAA in treating muscle atrophy induced by ID. Evidence from an *in vitro* study suggests that the efficacy of BCAA as promoters of protein synthesis appears to be highly dependent on cellular iron status, and ID makes muscle cells hyposensitive to the anabolic signaling of BCAA [23]. Moreover, an imbalance of BCAA leads to exacerbating pathological conditions such as severe anemia [24]. Therefore, investigating the impact of BCAA on ID-induced muscle atrophy is of significant importance for developing new preventive and therapeutic strategies.

The purpose of the study is to investigate the effects of BCAA on ID-induced muscle atrophy. Specifically, we first validated the efficacy of DFO in establishing an iron-deficient C2C12 myotube model. Then we measured myotube diameter and calculated the nuclear fusion index (NFI) through immunofluorescence, and analyzed gene expression of two classical atrogenes using RT-qPCR. Furthermore, we also explored the underlying molecular mechanisms by conducting western blotting (WB). The results showed that BCAA may partially promote protein synthesis-related signaling pathways, but is insufficient to prevent ID-induced-muscle atrophy.

## 2 Materials and methods

### 2.1 Myoblasts culture and treatments

The C2C12 mouse myoblast cell line (RIKEN BioResource Research Center, Ibaraki, Japan; Cat# RCB0987) was used for all experiments described. The cell line was certified as mycoplasma-free by the supplier. Cells from passage 2 to passage 5 were used in this study. Myoblasts culture and *in vitro* treatments were performed as previously described with some modifications [16]. In brief, cells were seeded at a density of 8.7 × 10^3^ cells/cm^2^ in growth medium (GM) consisting of Dulbecco's Modified Eagle Medium (4.5 g/l Glucose) with L-Gln and Sodium Pyruvate (DMEM; #08458-16, Nacalai Tesque, Kyoto, Japan) supplemented with 10 % fetal bovine serum (FBS; #173012, Sigma-Aldrich, MO, USA) and 1 % penicillin/streptomycin (P/S; #15140122, Thermo Fisher Scientific, MA, USA). When the cells reached 80 % confluence, the medium was switched to differentiation medium (DM), and the cells were cultured for five days to induce myotube formation. DM comprised DMEM supplemented with 2 % horse serum (HS; #16050130, Thermo Fisher Scientific) and 1 % P/S. All cell cultures were maintained at 37 °C in a humidified incubator with 5 % CO_2_, and the medium was replaced every two days. After five days (designated as Day 0) of culture in DM, the iron chelator deferoxamine (DFO; #D9533, Sigma-Aldrich), which was applied by previous studies to establish an iron deficient *in vitro* model, was administered at a concentration of 100 μM in the DFO and DFO + BCAA groups for 2 or 4 days [10,23]. For BCAA stimulation, serum and BCAA were excluded from the medium for 90 min to starve myotubes, then BCAA (l-Leucine, l-Isoleucine, and l-Valine, Tokyo Chemical Industry Co., Ltd., Tokyo, Japan) were added at a concentration of 5 mM (l-leucine: l-isoleucine: l-valine = 2:1:1) for 45 min or 24 h before harvesting cells for protein extraction [16,23]. The experimental design of this study is shown in Fig. 1.

**Fig. 1:**
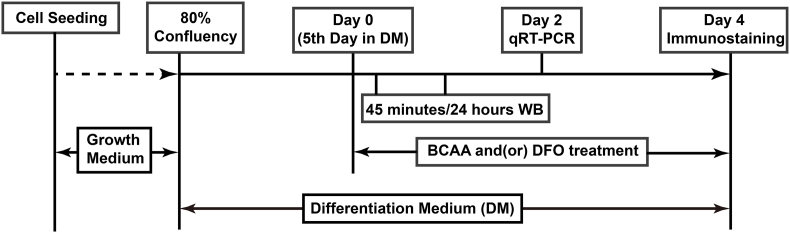
The experimental design of the present study.

### 2.2 Measurement of iron levels

Intracellular total iron levels were measured from harvested C2C12 myotubes of three experimental groups, the control, the DFO only, and the DFO + FeCl_3_ groups, using an iron colorimetric assay kit (K390-100; BioVision, Milpitas,CA, USA) according to the manufacturer's instructions on Day 2. Both DFO and FeCl_3_ (#157740, Sigma-Aldrich) were administered at a concentration of 100 μM [23]. Measurement of iron levels was performed twice for each sample, and statistical analysis was performed on data from three independent experiments (N = 3).

### 2.3 RNA extraction and quantitative real-time polymerase chain reaction (RT-qPCR)

Total RNA was extracted from C2C12 myotubes on Day 2 using the RNeasy Mini Kit (#74106; QIAGEN, Hilden, Germany). Reverse transcription was then performed using the SuperScript VILO cDNA Synthesis Kit (#11754050; Thermo Fisher Scientific). RT-qPCR was conducted using the Fast SYBR Green Master Mix (#3009682; Thermo Fisher Scientific) on a StepOnePlus Real-Time PCR System (Thermo Fisher Scientific) following the manufacturer's protocol. Target gene expression levels were normalized to β-actin, and fold changes were calculated relative to the control group using the 2^−ΔΔCt^ method. The primer sequences are listed in Table 1. RT-qPCR was performed twice for each sample, and statistical analysis was performed on data from three independent experiments (N = 3).

**Table 1:** RT-qPCR primer sequences for target genes.

Primers	Forward (5’ --- 3′)	Reverse (5’ --- 3′)
β-actin	AGAGGGAAATCGTGCGTGAC	CAATAGTGATGACCTGGCCGT
Atrogin-1	CAGCAGCTGAATAGCATCCAGAT	TCTGCATGATGTTCAGTTGTAAGC
MuRF-1	ACGAGAAGAAGAGGCGAGCTG	CTTGGCACTTGAGAGGAAGG

### 2.4 WB

Total protein was extracted with RIPA buffer from C2C12 myotubes and was then separated by sodium dodecyl sulfate polyacrylamide gel electrophoresis (SDS-PAGE). After electrophoresis, proteins were transferred to polyvinylidene difluoride membranes, which were blocked with 5 % non-fat milk dissolved in Tris-buffered saline containing 0.05 % Tween 20 (TBS-T) for 1 h at room temperature. Membranes were then incubated overnight at 4 °C with primary antibodies. Finally, membranes were incubated for 1 h with second antibodies, and visualized using an enhanced chemiluminescence system. Antibodies used in this study are listed in Table 2. Integrated density of each band was measured using ImageJ with Fiji (version 1.35). WB was performed twice for each sample, and statistical analysis was performed on data from three independent experiments (N = 3).

**Table 2:** Antibodies for WB.

Antibody Name	Antigen	Catalog Number	Dilution Ratio	Manufacturer
*p*-AMPKα (Thr172)	Rabbit	2535	1:1000	Cell Signaling Technology
AMPK	Mouse	2793	1:1000	Cell Signaling Technology
p-Acetyl-CoA Carboxylase (Ser79)	Rabbit	3676	1:1000	Cell Signaling Technology
Acetyl-CoA Carboxylase	Rabbit	3676	1:1000	Cell Signaling Technology
p-Akt (Ser473)	Rabbit	4060	1:2000	Cell Signaling Technology
Akt	Rabbit	9272	1:1000	Cell Signaling Technology
p-mTOR (Ser2448)	Rabbit	5536	1:1000	Cell Signaling Technology
mTOR	Rabbit	2983	1:1000	Cell Signaling Technology
p-p70 S6 Kinase (Thr389)	Rabbit	9234	1:1000	Cell Signaling Technology
p70 S6 Kinase	Rabbit	9202	1:1000	Cell Signaling Technology
p-4E-BP1	Rabbit	9451	1:1000	Cell Signaling Technology
4E-BP1	Rabbit	9452	1:1000	Cell Signaling Technology
p-eEF2 (Thr56)	Rabbit	2331	1:1000	Cell Signaling Technology
eEF2	Rabbit	2332	1:1000	Cell Signaling Technology
p-FoxO1 (Ser256)	Rabbit	84192	1:1000	Cell Signaling Technology
FoxO1	Rabbit	9454	1:1000	Cell Signaling Technology
Anti-Atrogin-1 antibody	Rabbit	ab168372	1:1000	Abcam
MuRF1 Antibody	Mouse	sc-398608	1:1000	Santa Cruz Biotechnology
p–NF–κB p65 (Ser536)	Rabbit	3033	1:1000	Cell Signaling Technology
NF-κB p65	Rabbit	8242	1:1000	Cell Signaling Technology
β-Actin	Rabbit	4970	1:1000	Cell Signaling Technology
GAPDH	Rabbit	2118	1:1000	Cell Signaling Technology
Anti-Rabbit IgG HRP-conjugated secondary antibody	Donkey	NA934	1:2000	Cytiva
Anti-Mouse IgG HRP-conjugated secondary antibody	Sheep	NA931	1:2000	Cytiva

### 2.5 Immunofluorescence (IF)

On Day 4, C2C12 myotubes were washed with phosphate buffered saline (PBS) and then fixed with 4 % paraformaldehyde (#09154-85; Nacalai Tesque, Kyoto, Japan) at room temperature for 20 min. The cells were then permeabilized with 0.1 % TritonX-100 for 15 min and then blocked with 5 % bovine serum albumin (BSA; #A2153; Sigma-Aldrich, St. Louis, MO, USA) for 20 min, and incubated overnight at 4 °C with the primary antibody, a mouse monoclonal anti-myosin heavy chain (MyHC) antibody (#ab11083; Abcam plc., Cambridge, UK). After washing with 0.1 % BSA, the cells were incubated with the secondary antibody, Alexa Fluor-conjugated antibody (#A32723; Invitrogen, Carlsbad, CA, USA), at room temperature for 1 h. Nuclei were counterstained using an anti-fade mounting medium with 4’,6-diamidino-2- Phenylindole (DAPI) (#2454141; Invitrogen). Fluorescence images were acquired using an inverted fluorescence microscope (#Ts2-FL; Nikon Corporation, Tokyo, Japan). For each treatment group, three independent biological replicates were included (N = 3). From each well, five images were randomly taken at 200 × magnification. For each image, MyHC-positive myotubes containing two or more nuclei were counted, and their diameter was measured. The average myotube diameter for each well was calculated for statistical analysis. Image analysis was performed using ImageJ with Fiji (version 2.14.0) by an investigator who was blinded to the group identities.

Moreover, to quantify myoblast fusion, the open-source program MyoCount (Version 1.3.1) via MATLAB Runtime (Version 9.4) was used to determine the NFI and the number of total nuclei within myotube [25]. NFI was calculated by dividing the number of nuclei inside MyHC-positive myotubes by the total count of nuclei [25]. For each treatment group, three independent biological replicates were included (N = 3). From each well, three images were randomly taken at 200 × magnification. The average NFI or the number of total nuclei within myotube for each well was calculated for statistical analysis.

### 2.6 Quantification and statistical analysis

Results were obtained from three independent experiments. Data are presented as the mean ± SD. Multiple comparisons were performed using one-way analysis of variance (ANOVA) with eta squared (η^2^) used to measure overall effect sizes, followed by a post hoc Tukey–Kramer test. Effect sizes for differences between individual groups were measured using Cohen's d. Assumptions of normality and homogeneity of variance were verified for all ANOVA tests using Shapiro-Wilk and Levene's tests, respectively. Statistical analyses were carried out using GraphPad Prism v9.5, and p < 0.05 was considered statistically significant.

## 3 Results

### 3.1 DFO treatment had no toxic or off target effects on C2C12 myotubes

**Fig. 2:**
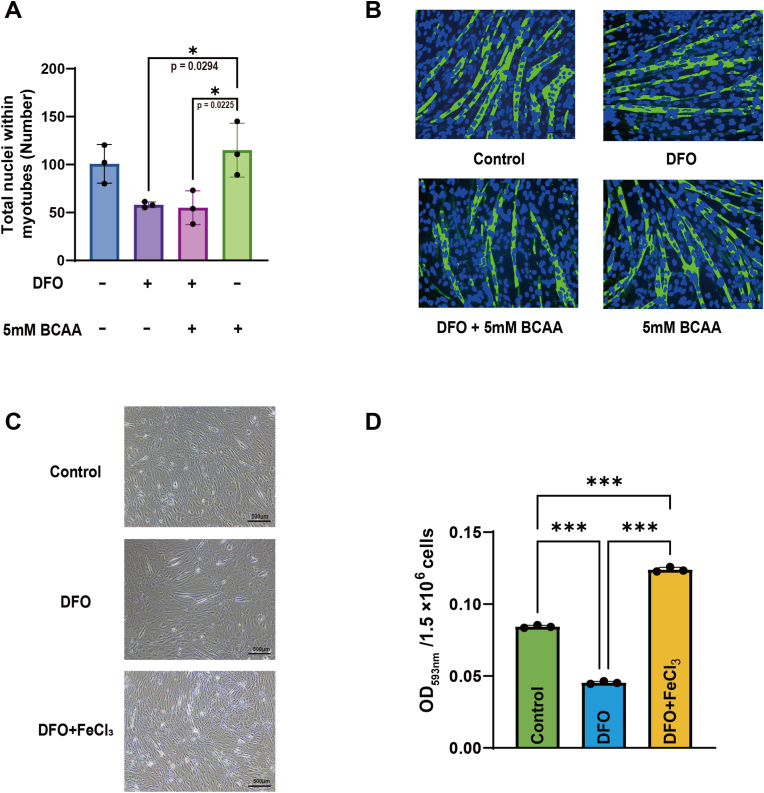
**DFO treatment had no toxic or off target effects on C2C12 myotubes.** (A) Quantification of total nuclei within myotubes (ANOVA; F (3, 8) = 7.248, p = 0.0114, η^2^ = 0.731) (N = 3) (B) Representative analysis images in each group generated by the MyoCount program, used to quantify total nuclei within myotubes. Green, myotubes. Blue dots, nuclei. (C) Representative images of C2C12 myotubes after treatment with DFO, DFO + FeCl_3_, or control. Scale bars, 500 μm. Magnification, 100x. (D) Quantification of OD583nm/1.5 × 10^6^ cells (ANOVA; F (2, 6) = 3166, p < 0.001, η^2^ = 0.999) (N = 3). Data are presented as the mean ± SD (error bars) from 3 independent experiments, and were analyzed using one-way analysis of variance with eta squared (η^2^) used to measure effect sizes, followed by a post hoc Tukey–Kramer test. ∗p < 0.05, ∗∗∗p < 0.001. NFI, nuclear fusion index.

DFO is a highly effective Fe^3+^ chelator. Mechanistically, DFO binds to the labile iron pool and functionally mimics iron deficiency by limiting iron availability for essential metabolic processes [26]. Moreover, DFO can markedly downregulate ferritin expression while upregulating transferrin receptor 1 (TfR1) and ferroportin [27]. To evaluate the potential toxicity of DFO on C2C12 myotubes, we quantified the number of total nuclei within myotube on Day 4 using MyoCount software after IF. Results showed that no significant difference in the myotube nuclei count was observed among the control, DFO, and DFO + BCAA groups (Fig. 2A and B). However, when compared to the BCAA only group, both the DFO, and DFO + BCAA groups exhibited a significant decrease in the number of total nuclei within myotube (p = 0.0294, Cohen's d = 2.92; p = 0.0225, Cohen's d = 3.08, respectively). Moreover, to confirm the on-target, iron-chelating effect of DFO on C2C12 myotubes, intracellular iron levels were measured on Day 2 using an iron colorimetric assay kit. Results showed that compared to the control group, iron levels in the DFO group were significantly decreased (p < 0.001, Cohen's d = 32.26) (Fig. 2C and D). Moreover, the effect of DFO on intracellular iron level was rescued by co-treatment with FeCl_3_, as iron levels in the DFO + FeCl_3_ group were significantly increased compared to that of the DFO group (p < 0.001, Cohen's d = 64.95). Results verified that DFO treatment had no toxic or off target effects on C2C12 myotubes.

### 3.2 BCAA had minimal effects on myotube diameter

**Fig. 3:**
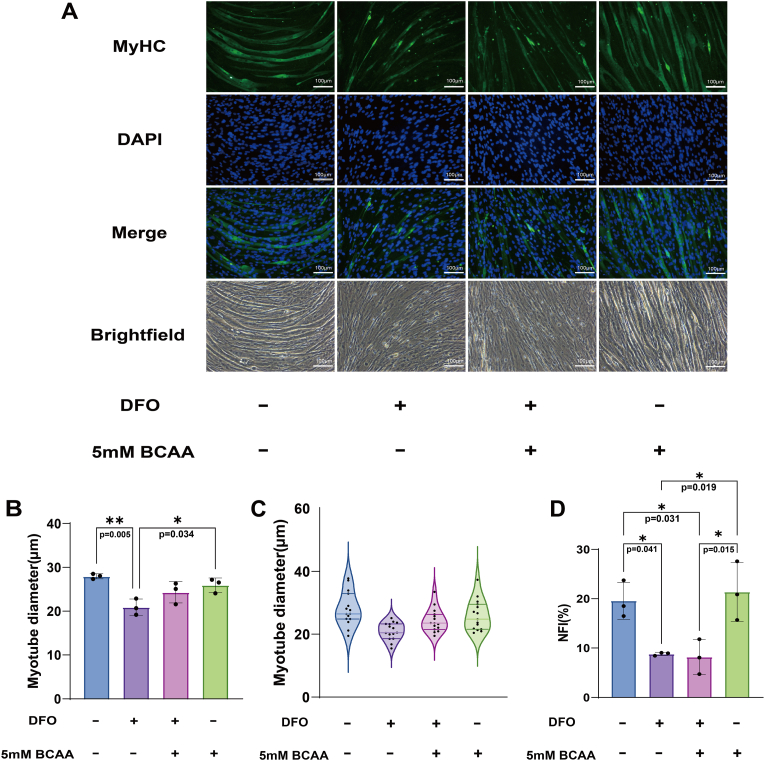
**BCAA had minimal effects on myotube diameter.** (A) Representative immunocytochemical images of myotubes. Green: Myotubes (MyHC staining). Blue: Nuclei (DAPI staining). Scale bars, 100 μm. Magnification, 200x. (B) Quantification of the myotube diameter (N = 3). Data are presented as the mean ± SD (error bars) from 3 independent experiments, and were analyzed using one-way analysis of variance with eta squared (η^2^) used to measure effect sizes (ANOVA; F (3, 8) = 8.454, p = 0.007, η^2^ = 0.760), followed by a post hoc Tukey–Kramer test. ∗p < 0.05, ∗∗∗p < 0.001. (C) The violin plot of the relationships between myotube diameter and four experimental groups. The distribution of data in each group was evaluated. The horizontal axis presents the myotube diameter while the vertical axis presents four experimental groups. (D) Quantification of NFI (N = 3). Data are presented as the mean ± SD (error bars) from 3 independent experiments and were analyzed using one-way analysis of variance with eta squared (η^2^) used to measure effect sizes (ANOVA; F (3, 8) = 9.374, p = 0.005, η^2^ = 0.779), followed by a post hoc Tukey–Kramer test. ∗p < 0.05, ∗∗∗p < 0.001. NFI, nuclear fusion index.

A decrease in myotube diameter is an indicator of muscle atrophy [18]. A previous study showed that *in vitro* myotubes from patients exhibit reduced diameters, which are associated with enhanced atrophic signaling pathways [28]. To evaluate the effect of BCAA on myotube diameter, IF was performed on Day 4 (Fig. 3). Compared to the control and BCAA only groups, the myotube diameter in the DFO group was significantly reduced by approximately 25.0 % (p = 0.005, Cohen's d = 3.97), and 19.35 % (p = 0.034, Cohen's d = 2.83), respectively (Fig. 3A and B). However, the DFO + BCAA group showed no significant difference compared to the other three groups. The distribution of all data in each group was shown in the violin plot (Fig. 3C). Moreover, a similar pattern was observed for NFI, where DFO induced a significant decrease both in the DFO and DFO + BCAA groups, compared to the control and BCAA only groups (control vs. DFO: p = 0.041, Cohen's d = 2.74; BCAA vs. DFO: p = 0.019, Cohen's d = 3.19; Control vs. DFO + BCAA: p = 0.031, Cohen's d = 2.89; BCAA vs. DFO + BCAA: p = 0.015, Cohen's d = 3.35) (Fig. 3D). These results indicated that BCAA had little effect on reversing the ID-induced myotube atrophy.

### 3.3 BCAA reduced *Atrogin-1* expression in DFO-treated myotubes, but had no effect on *MuRF-1* expression

**Fig. 4:**
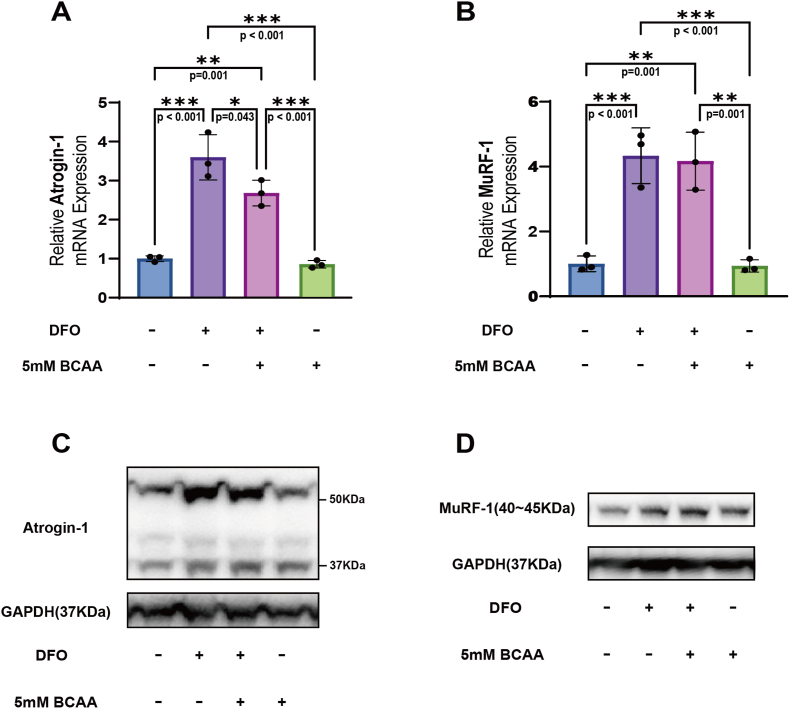
**BCAA reduced *Atrogin-1* expression in DFO-treated myotubes, but had no effect on *MuRF-1* expression.** (A) Relative expression of Atrogin-1(F (3, 8) = 46.837, p < 0.001, η^2^ = 0.946) (N = 3). (B) Relative expression of MuRF-1(F (3, 8) = 26.29, p < 0.001, η^2^ = 0.908) (N = 3). (C) (D) Representative WB blot images showing the protein expression of Atrogin-1 and MuRF-1. Protein was extracted on 24 h after BCAA treatment. Data are presented as the mean ± SD (error bars) from three independent experiments, and were analyzed using one-way analysis of variance (ANOVA) with eta squared (η^2^) used to measure effect sizes, followed by a post hoc Tukey–Kramer test. ∗p < 0.05, ∗∗p < 0.01, ∗∗∗p < 0.001.

To evaluate the effect of BCAA on mRNA expressions of *Atrogin-1* and *MuRF-1*, which are atrogenes associated with muscle degradation, RT-qPCR was performed on Day 2. Compared to the control and BCAA only groups, mRNA expression levels of *Atrogin-1* and *MuRF-1* were significantly increased in both the DFO group and DFO + BCAA group (Fig. 4A and B). However, *Atrogin-1* expression was significantly lower in the DFO + BCAA group than in the DFO group (p = 0.043, Cohen's d = 2.70) (Fig. 4A). Additionally, no significant difference was observed in *MuRF-1* expression between the DFO and the DFO + BCAA groups (Fig. 4B). Furthermore, we also investigated protein expression levels of Atrogin-1 and MuRF-1 using WB. Representative blot images (Fig. 4C and D) showed the protein expression of Atrogin-1 and MuRF-1 among four groups, which was consistent with the results of RT-qPCR. These results demonstrated that BCAA could suppress the expression of *Atrogin-1*, but had no effect on *MuRF-1* expression.

### 3.4 BCAA increased the expression level of p-Akt in DFO-treated myotubes after 45 min of treatment

**Fig. 5:**
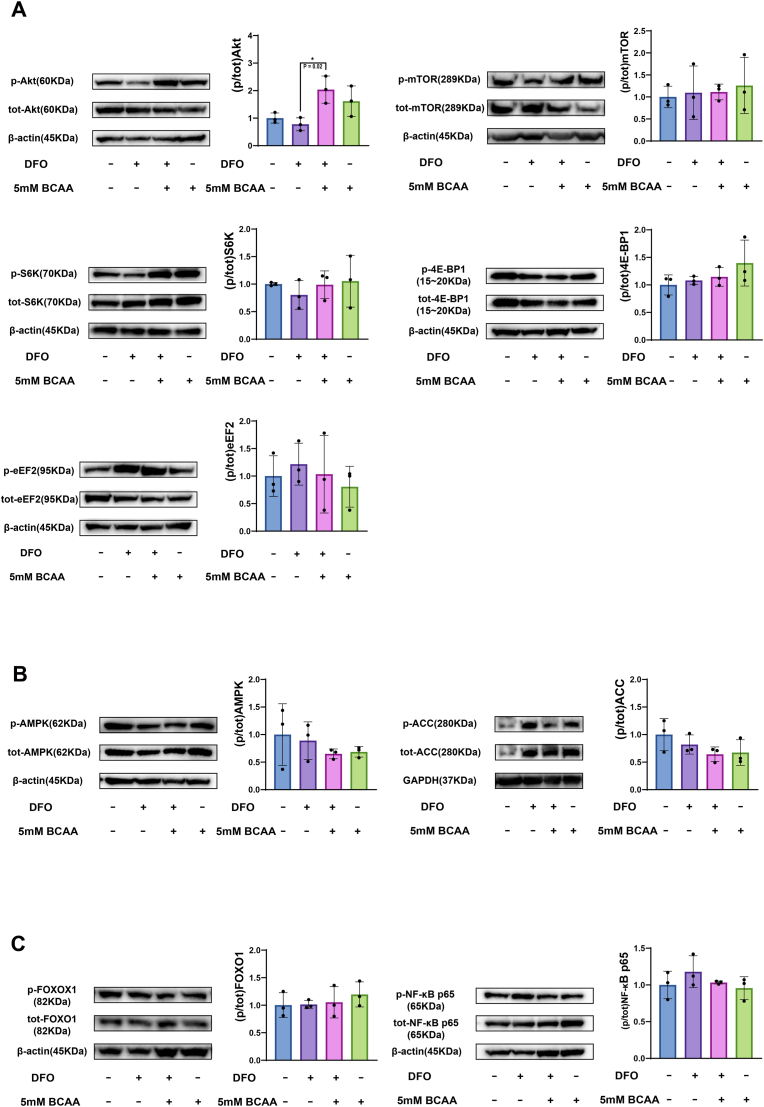
**BCAA increased the p-Akt in DFO-treated myotubes after 45 min of BCAA treatment, but had no statistically significant effects on other signaling molecules at the same time point.** Representative blots for quantification of (A) p-Akt (F (3, 8) = 6.166, p = 0.018, η^2^ = 0.6981), p-mTOR, p-p70S6K, p-4E-BP1, and p-eEF2, (B) p-AMPK, and p-ACC, and (C) p-FOXO1, and p–NF–κB p65. Protein was extracted at 45 min after BCCA treatment. Data are presented as the mean ± SD (error bars) from three independent experiments (N = 3), and were analyzed using one-way analysis of variance (ANOVA) with eta squared (η^2^) used to measure effect sizes, followed by a post hoc Tukey–Kramer test. ∗p < 0.05.

BCAA promote protein synthesis by activating the Akt/mTORC1, which subsequently regulates its two major downstream effectors, ribosomal protein S6 kinase 1 (S6K1) and eukaryotic initiation factor 4E binding protein 1 (4EBP1) [29,30]. Activated S6K1 subsequently inhibits eEF2K, preventing the inactivating phosphorylation of eEF2 and thus promoting translation elongation [31,32]. Therefore, to explore the underlying signaling mechanism of the C2C12 myotube response to BCAA stimulation, we measured the protein levels of Akt, mTOR, p70S6K, 4E-BP1, and eEF2 by WB after 45 min of BCAA treatment (Fig. 5A). Results demonstrated that p-Akt was significantly downregulated in the DFO group, compared to the DFO + BCAA group (p = 0.02, Cohen's d = 3.14).

Moreover, AMPK phosphorylation is considered an indicator of muscle degradation, and critically, eEF2K is a known downstream target regulated by AMPK [33]. To confirm whether the AMPK signaling pathway was activated or not during BCAA stimulation, we also analyzed the protein levels of AMPK, and Acetyl-CoA Carboxylase (ACC), which is downstream target of AMPK. However, no evidence was found that p-AMPK or p-ACC was upregulated after 45 min of BCAA treatment (Fig. 5B).

On the other hand, previous studies indicated that Akt inhibits the transcription factor FOXO, which in turn controls the expression of Atrogin-1 and MuRF-1 [34,35]. Additionally, MuRF-1 expression is also directly regulated by NF-κB [36]. To further elucidate the regulatory status of the signaling pathways which FOXO and NF-κB involve, we measured the protein levels of FOXO and NF-kB P65 (Fig. 5C). Our analysis revealed no statistically significant differences in the expression levels of FOXO or NF-κB P65 across the experimental groups.

### 3.5 BCAA increased the p-p70S6K in DFO-treated myotubes after 24 h of treatment

**Fig. 6:**
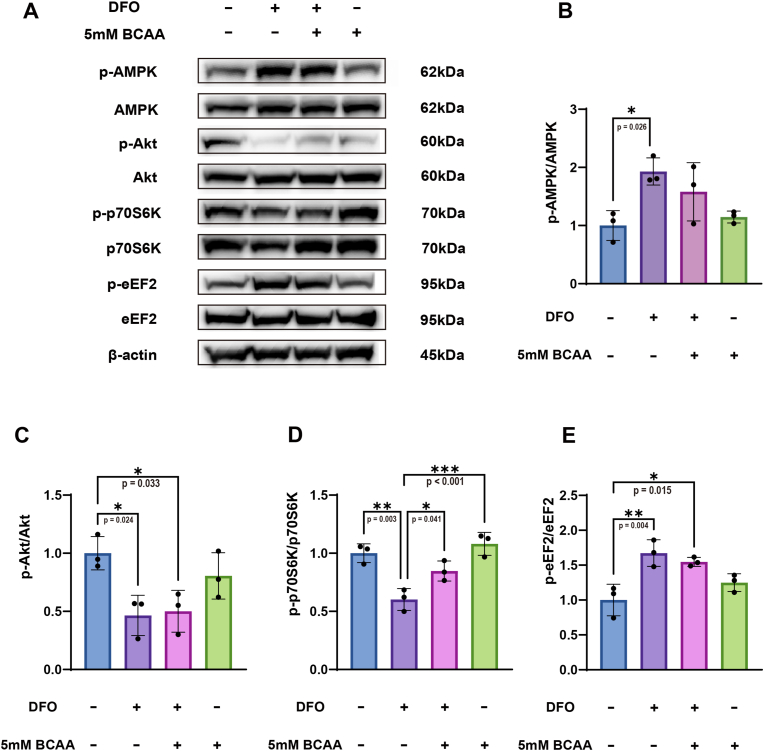
**BCAA increased the phosphorylation of p70S6K in DFO-treated myotubes after 24 h of BCAA treatment, but had no effect on the phosphorylation of AMPK, Akt, or eEF2.** (A) Representative blots for quantification of (B) p-AMPK(F (3, 8) = 5.620, p = 0.023, η^2^ = 0.678), (C) p-Akt (F (3, 8) = 6.394, p = 0.016, η^2^ = 0.706), (D) p-p70S6K(F (3, 8) = 16.43, p < 0.001, η^2^ = 0.860), and (E) p-eEF2 (F (3, 8) = 10.23, p = 0.004, η^2^ = 0.793). Protein was extracted 24 h after BCCA treatment. Data are presented as the mean ± SD (error bars) from three independent experiments (N = 3), and were analyzed using one-way analysis of variance (ANOVA) with eta squared (η^2^) used to measure effect sizes, followed by a post hoc Tukey–Kramer test. ∗p < 0.05, ∗∗p < 0.01.

Effects of BCAA on the phosphorylation of AMPK, Akt, p70S6K, and eEF2 after 24 h treatment were further investigated by conducting WB (Fig. 6A). Compared to the control group, *p*-AMPK was significantly increased in the DFO group (p = 0.026, Cohen's d = 3.01) (Fig. 6B). Meanwhile, compared to the control group, the p-Akt levels were significantly decreased in the DFO (p = 0.024, Cohen's d = 3.06) and DFO + BCAA groups (p = 0.033, Cohen's d = 2.85) (Fig. 6C). This result could be also verified by the gel band (Fig. 6A). In addition, compared to the control and BCAA only groups, the p-p70S6K level was significantly reduced in the DFO group (p = 0.003, Cohen's d = 4.42; p < 0.001, Cohen's d = 5.32). In contrast, BCAA supplementation significantly increased p-p70S6K expression in the DFO + BCAA group compared to the DFO group, restoring it to levels not significantly different from the control group (p = 0.041, Cohen's d = 2.71) (Fig. 6D). Lastly, p-eEF2, which suppresses muscle synthesis, was significantly elevated in both the DFO and DFO + BCAA groups compared to the control group (p = 0.004, Cohen's d = 4.11; p = 0.015, Cohen's d = 3.34, respectively) (Fig. 6E). Our results demonstrated that BCAA treatment for 24 h significantly elevated p-p70S6K levels in DFO-treated myotubes, but had no statistically significant effects on the phosphorylation of AMPK, Akt or eEF2.

## 4 Discussion

The purpose of this study was to investigate the effects of BCAA on ID-induced muscle atrophy. To induce an ID environment, DFO was used, and the effects of BCAA on muscle atrophy were evaluated. As a result, under DFO-induced suppression, BCAA partially upregulated protein synthesis–related pathways and attenuated protein degradation–related processes, as evidenced by the recovery of Akt and p70S6K phosphorylation and the downregulation of Atrogin-1 expression (Figs. 4A, 5A and 6D). However, BCAA did not suppress the reduction in myotube diameter (Fig. 3B), nor did it inhibit MuRF-1 expression (Fig. 4B) or AMPK phosphorylation (Figs. 5B and 6B). These results suggest that the inhibitory effects of BCAA on the expression of genes involved in muscle protein degradation are limited. On the other hand, though BCAA promoted the phosphorylation of p70S6K, the lack of effects on (p/total-)eEF2 suggests that BCAA only partially upregulated the muscle protein synthesis-related signaling pathways. Therefore, the effects of BCAA observed in the ID-induced muscle atrophy model of this study are different from those previously reported in DEX-induced muscle atrophy models. Several aspects of our findings merit comparison with previous researches.

First, DFO treatment induces functional iron deprivation by chelating Fe^3+^ and limiting cellular access to labile iron. Mechanistically, DFO stimulates autophagic ferritin degradation, upregulates TfR1/DMT1, disrupts mitochondrial electron transport, and mimics cellular responses typically observed under true iron deficiency [26,27]. Although culturing cells in iron-depleted medium is one approach to mimic iron deficiency, this method is limited by the residual iron present in serum, and serum deprivation itself can trigger complex cellular responses and activates numerous signaling pathways unrelated to iron metabolism, such as proliferation inhibition, apoptosis and suppression of calcium signaling activity [[37], [38], [39]]. In contrast, DFO treatment, which rapidly induces a functional iron-deficient state, allows for precise temporal and dose-dependent control of iron deprivation effectively, providing better reproducibility across experiments [40]. In addition, the present study demonstrated that DFO treatment had no toxic or off target effects on C2C12 myotubes (Fig. 2).

Moreover, atrogenes, such as Atrogin-1 and MuRF-1, are E3 ubiquitin ligases of the ubiquitin–proteasome system (UPS) [5,21,41,42]. UPS, along with autophagy–lysosome system and the calcium-dependent system (calpains), is recognized as the major cellular proteolytic systems [5,19]. Results of the present study showed that BCAA suppressed the expression of Atrogin-1 (Fig. 4A). However, it did not significantly affect the expression of MuRF-1 (Fig. 4B). It suggests that Atrogin-1 and MuRF-1 may respond differently to BCAA. Previous studies demonstrated that Atrogin-1 expression is primarily regulated through the Akt signaling pathway [43]. In this study, p-Akt and p-p70S6K levels were enhanced in the DFO + BCAA group (Figs. 5A and 6D), indicating the activation of the Akt pathway by BCAA. Furthermore, Atrogin-1 promotes protein degradation by targeting factors involved in muscle protein synthesis [44]. Therefore, suppression of Atrogin-1 expression may partially reduce muscle protein degradation. On the contrary, MuRF-1 expression has been reported to be regulated not only by the Akt signaling pathway but also by the NF-κB signaling pathway [36,45]. In the present study, no suppression of MuRF-1 expression was observed following BCAA treatment (Fig. 4B). These results suggest that ID may activate alternative catabolic signaling, thereby promoting MuRF-1 expression and counteracting the anabolic effects of BCAA via the Akt signaling pathway. Furthermore, MuRF-1 is known to primarily target structural muscle proteins, such as MyHC, for degradation [44]. Combined with our result that BCAA did not prevent the reduction in myotube diameter in our study (Fig. 3), it is possible that under iron-deficient conditions, non-Akt signaling pathways may have exerted a dominant influence, resulting in increased MuRF-1 expression and enhanced degradation of MyHC. Further investigation and statistically significant results are required to clarify why the inhibitory effect of BCAA is insufficient to inhibit MuRF-1 expression and prevent the reduction in myotube size.

Interestingly, although p-p70S6K was enhanced in the DFO + BCAA group (Fig. 6D), p-eEF2 was not downregulated compared to the DFO group (Fig. 6E). As a well-established downstream target of mTOR, p70S6K is directly regulated by the mTOR signaling pathway [46]. In contrast, eEF2 has been reported to be regulated not only by mTOR indirectly but also regulated by AMPK directly [33,47]. These findings suggest that under iron-deficient conditions, the activation of AMPK may predominate, thereby sustaining the level of p-eEF2 expression and counteracting the mTOR-mediated effect induced by BCAA. In the present study, p-AMPK levels were significantly increased in the DFO group (Fig. 6B). However, p-ACC, a canonical downstream target of AMPK, did not show a corresponding increase compared to other groups (Fig. 5B). Therefore, further investigation is required to validate the hypothesis that the activation of the AMPK pathway predominates in regulating p-eEF2 levels compared to the Akt/mTOR pathway.

Finally, it is well established that DEX influences both muscle protein synthesis and degradation via inhibition of the PI3K/Akt signaling pathway [19]. The inhibition of the PI3K/Akt signaling pathway leads to the activation of FOXO and inhibition of mTOR [19]. Inhibition of mTOR further suppresses the phosphorylation of downstream signaling molecules such as p70S6K and prevents the dephosphorylation of eEF2, ultimately leading to reduced muscle protein synthesis [16,33]. In addition, DEX promotes the expression of atrogenes and upregulates the expression of p-AMPK, which in turn promotes the expression of catabolic genes and prevents protein synthesis [16]. On the other hand, BCAA has been shown to enhance the phosphorylation of Akt and mTOR, which are suppressed by DEX, and thereby inhibit muscle protein degradation by downregulating the expression of ubiquitin ligases [48]. Additionally, BCAA exerts an effect against DEX-induced muscle atrophy by inhibiting the activation of AMPK [16,17,20]. Through these mechanisms, BCAA is considered capable of attenuating DEX-induced muscle atrophy. In our study, the protein expression of p-AMPK in the DFO group was significantly increased compared to the control group (Fig. 6B), which is consistent with the previous study that the iron deprivation induced by DFO resulted in AMPK upregulation [23]. However, in contrast to previous findings in the DEX-induced muscle atrophy model, where BCAA was reported to effectively suppress AMPK phosphorylation, the present study did not observe such an inhibitory effect in the ID-induced muscle atrophy (Fig. 6B). Combined with previous studies, our results highlight the complexity of the mechanisms underlying ID-induced muscle atrophy and suggest that preventive or therapeutic strategies may need to differ from those effective in DEX-induced muscle atrophy models.

There were some limitations to this study. First, effects of BCAA on mitochondrial function under iron-deficient conditions were not fully analyzed in the present study. Future research should focus on the mechanisms underlying mitochondrial dysfunction induced by ID, and investigate the potential effects of BCAA on this dysfunction. Additionally, it is important to comprehensively investigate the signaling pathways related to muscle atrophy, including processes such as oxidative stress, energy metabolism, and autophagy. Moreover, the standard DMEM used for all conditions, including the control group, contains a significant basal concentration of BCAA, totaling approximately 2.4 mM (with l-leucine, l-isoleucine, and l-valine each present at ∼0.8 mM). This means that our control group does not represent a true 'zero-BCAA' baseline. Consequently, the effects observed in our treatment groups are additive, reflecting the cellular response to BCAA supplementation above this existing basal level. It is possible that this background presence of BCAA could mask or attenuate the more subtle effects of our experimental treatments. However, there are few commercial DMEM medium with BCAA deletion. Future studies should consider a custom-formulated, BCAA-deficient DMEM. Finally, the present study was limited to *in vitro* experiments. Based on previous *in vitro* studies related, the concentration of BCAA treatment in the present study was 5 mM [16,23], which was much more than the concentration of BCAA in the normal human's blood (0.4–0.5 mM) [49], although even in the previous *in vivo* study, the concentration of BCAA was much higher than that level [17]. Therefore, future research should build upon these findings by conducting *in vivo* studies to further validate the effects of BCAA under iron-deficient conditions.

## 5 Conclusion

In this study, we investigated the effects of BCAA on ID-induced muscle atrophy. The results revealed that although BCAA exerted partial effects on muscle synthesis and degradation related signaling pathways, it showed no significant inhibitory effects on ID-induced muscle atrophy.

## Ethical approval

This article does not contain any studies with human participants or animals performed by any of the authors.

## Funding

This research did not receive any specific grant from funding agencies in the public, commercial, or not-for-profit sectors.

## CRediT authorship contribution statement

**Miki Kawanaka:** Formal analysis, Investigation, Writing – original draft. **Mingyuan Wang:** Data curation, Formal analysis, Investigation, Validation, Visualization, Writing – original draft, Writing – review & editing. **Toru Iwahashi:** Investigation, Methodology, Resources, Writing – review & editing. **Mai Konishi:** Investigation, Methodology. **Katsuyuki Konishi:** Investigation, Methodology. **Seira Sato:** Methodology, Resources. **Hiroyuki Tanaka:** Conceptualization, Methodology, Resources, Supervision, Writing – review & editing. **Ken Nakata:** Conceptualization, Supervision.

## Declaration of competing interest

The authors declare that they have no known competing financial interests or personal relationships that could have appeared to influence the work reported in this paper.

## Figures and Tables

**figs1:**
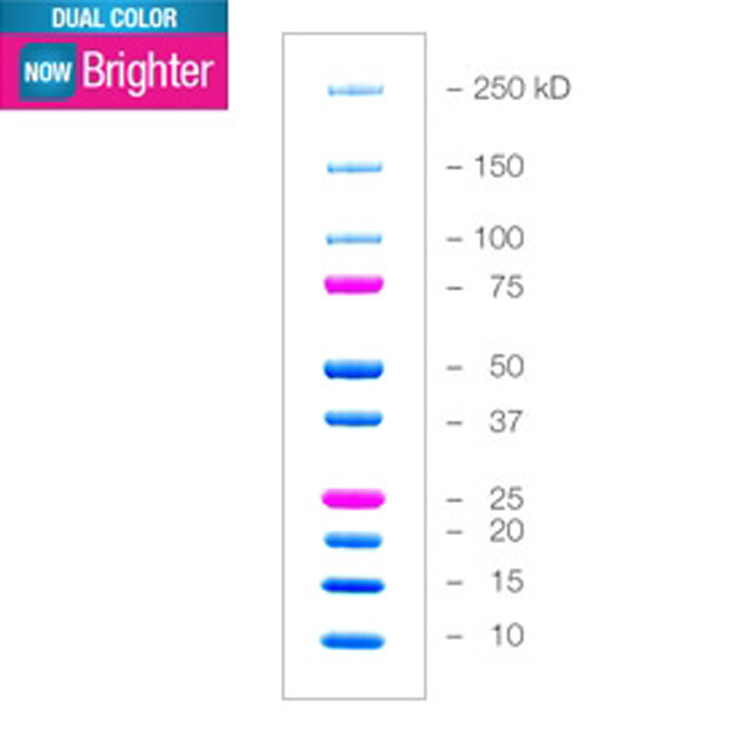


## Data Availability

The datasets generated and/or analyzed during the current study, including uncropped WB images and raw densitometry data, are available in the Zenodo repository under the accession code [DOI: 10.5281/zenodo.17247838].
