# Supplementary figures and images for: Effects of branched-chain amino acids on iron deficiency-induced muscle atrophy (part 2 of 2)
Source: Biochem Biophys Rep. 2026 Jan 17;45:102451. doi: 10.1016/j.bbrep.2026.102451 (PMC12854053; doi:10.1016/j.bbrep.2026.102451)

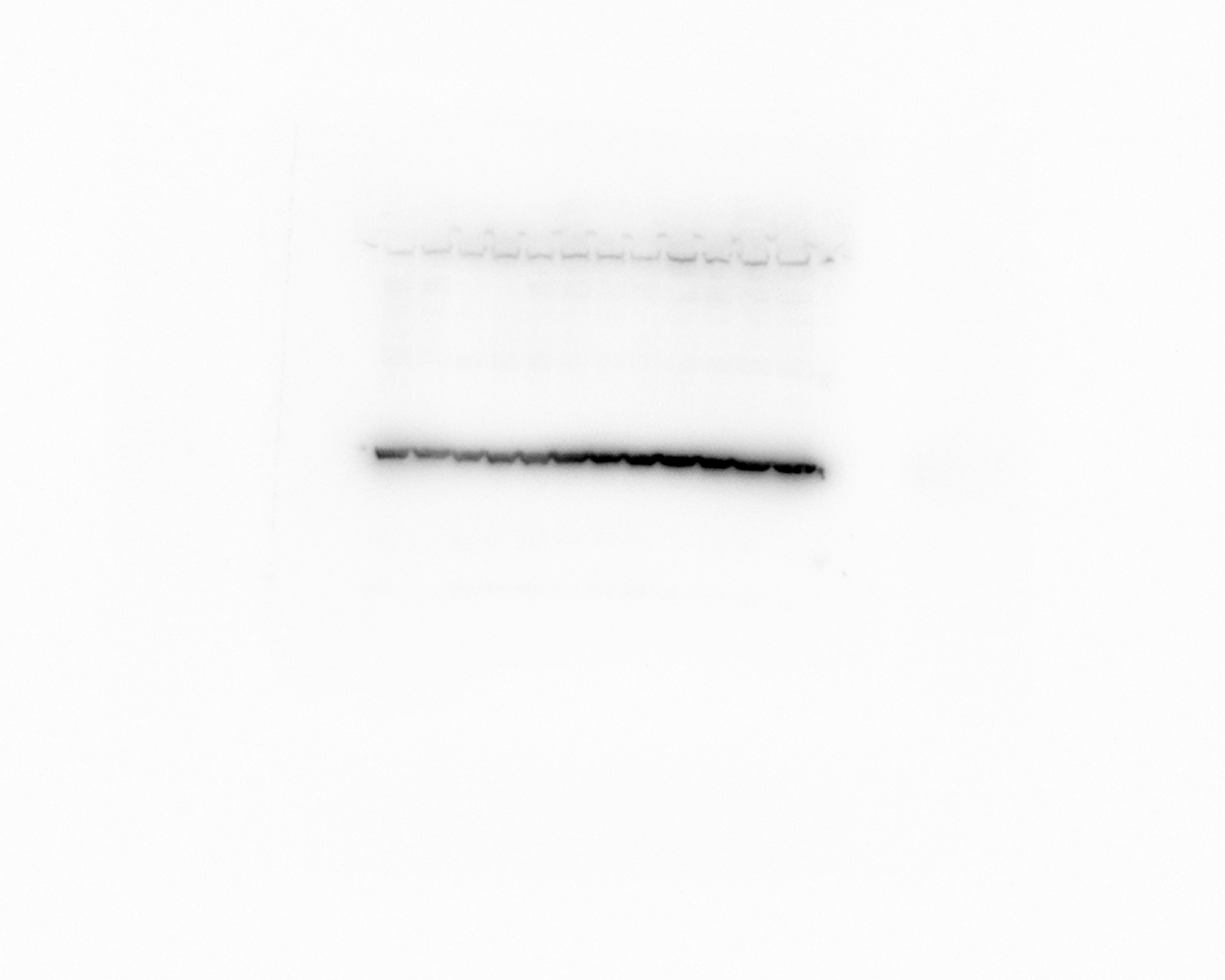

Supplement: Multimedia component 1 [file mmc1.zip › WB bands & raw densitometry/WB bands(45min)/10.(P-)mTOR/B-actin(mTOR)2(Chemiluminescence).tif]

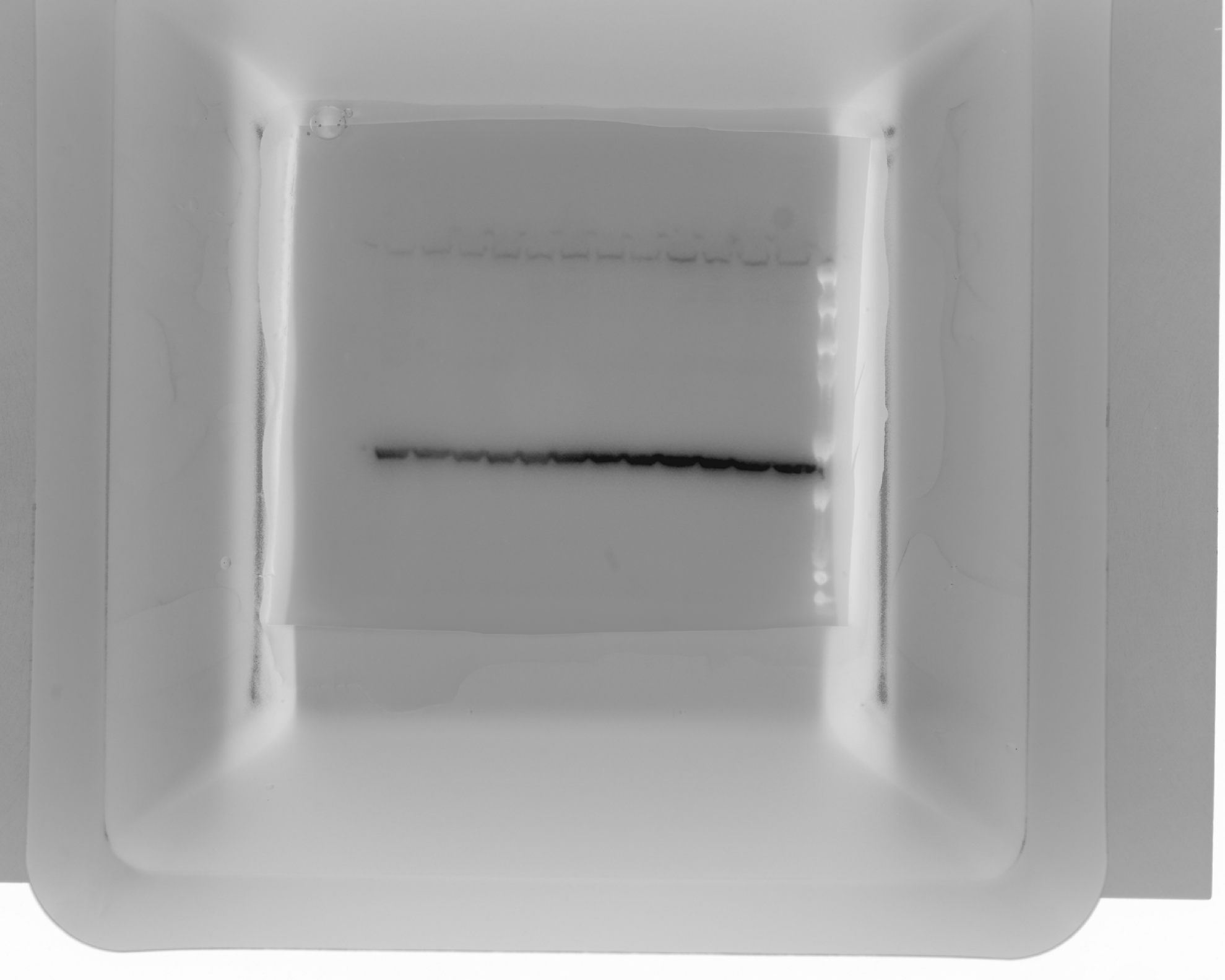

Supplement: Multimedia component 1 [file mmc1.zip › WB bands & raw densitometry/WB bands(45min)/10.(P-)mTOR/B-actin(mTOR)2(Composite).tif]

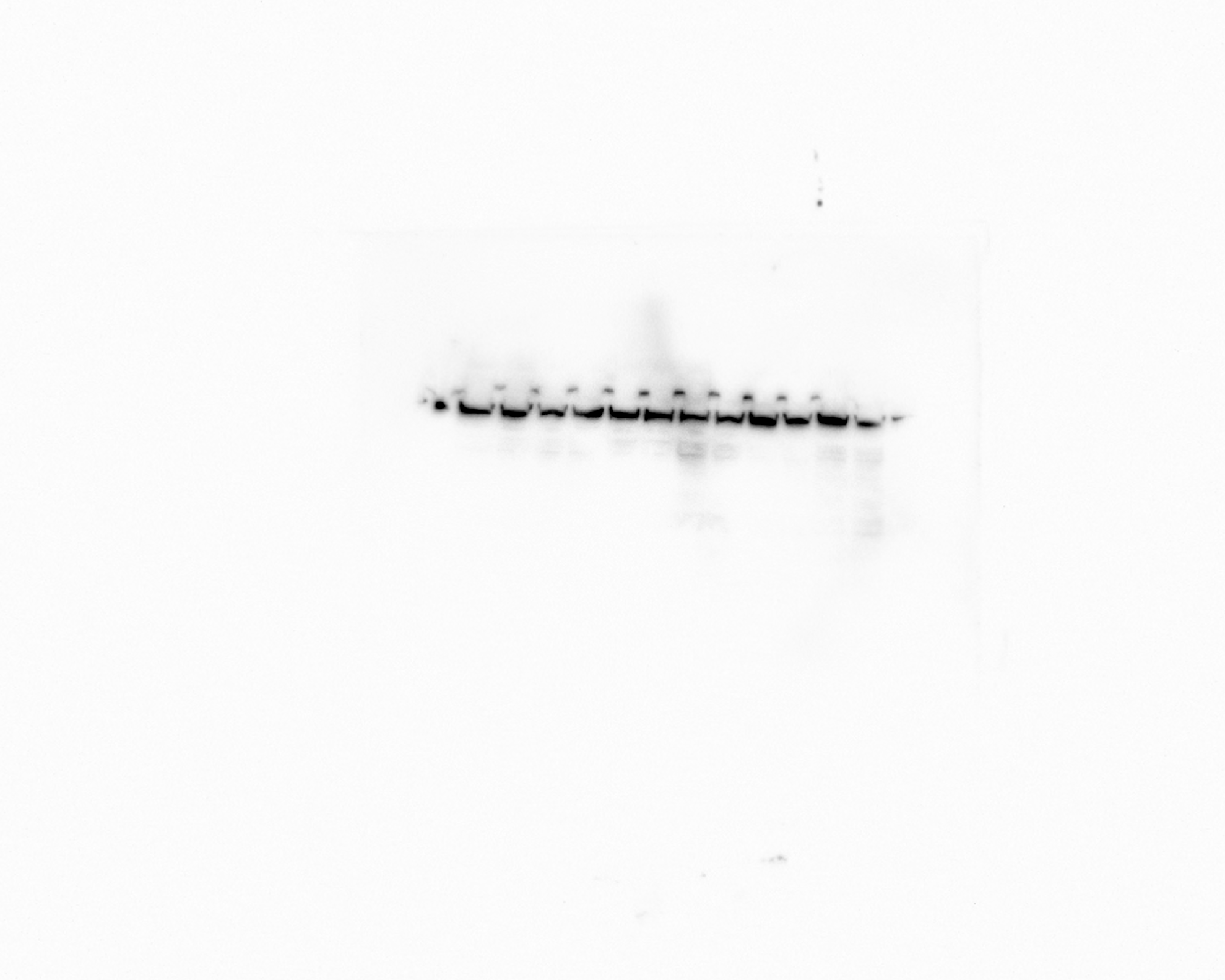

Supplement: Multimedia component 1 [file mmc1.zip › WB bands & raw densitometry/WB bands(45min)/10.(P-)mTOR/p-mTOR/p-mTOR(Chemiluminescence).tif]

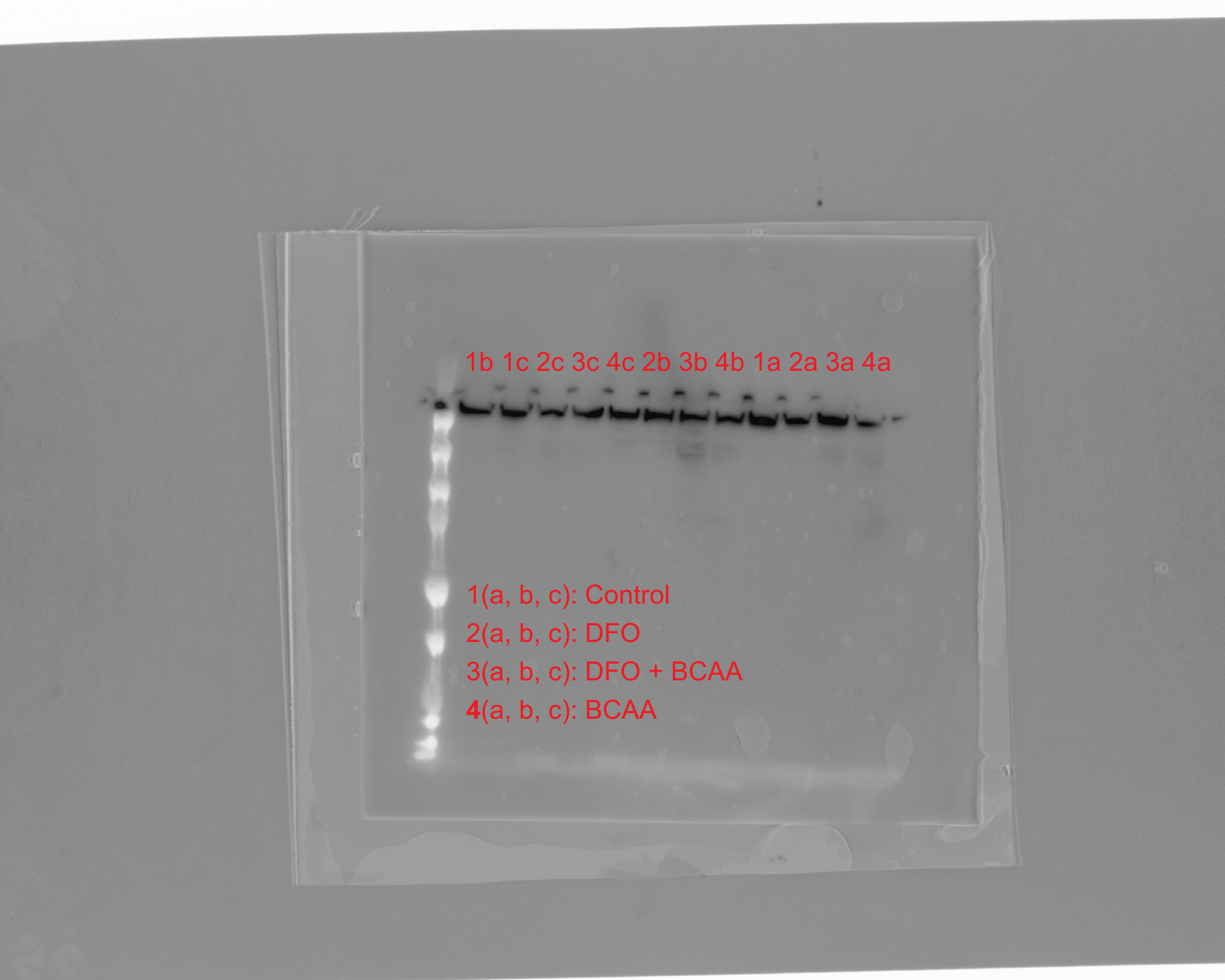

Supplement: Multimedia component 1 [file mmc1.zip › WB bands & raw densitometry/WB bands(45min)/10.(P-)mTOR/p-mTOR/p-mTOR(Composite).tif]

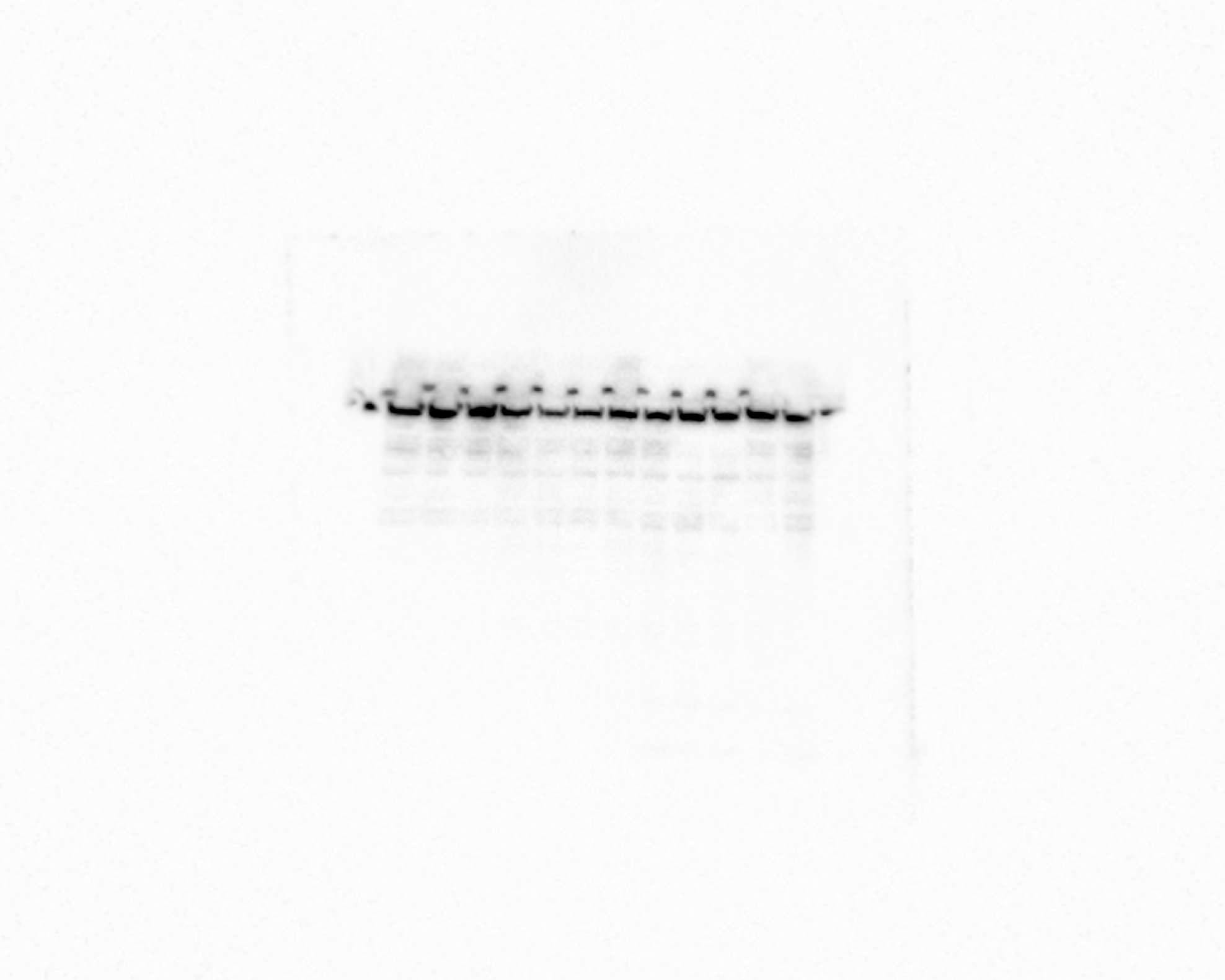

Supplement: Multimedia component 1 [file mmc1.zip › WB bands & raw densitometry/WB bands(45min)/10.(P-)mTOR/T-mTOR/mTOR(Chemiluminescence).tif]

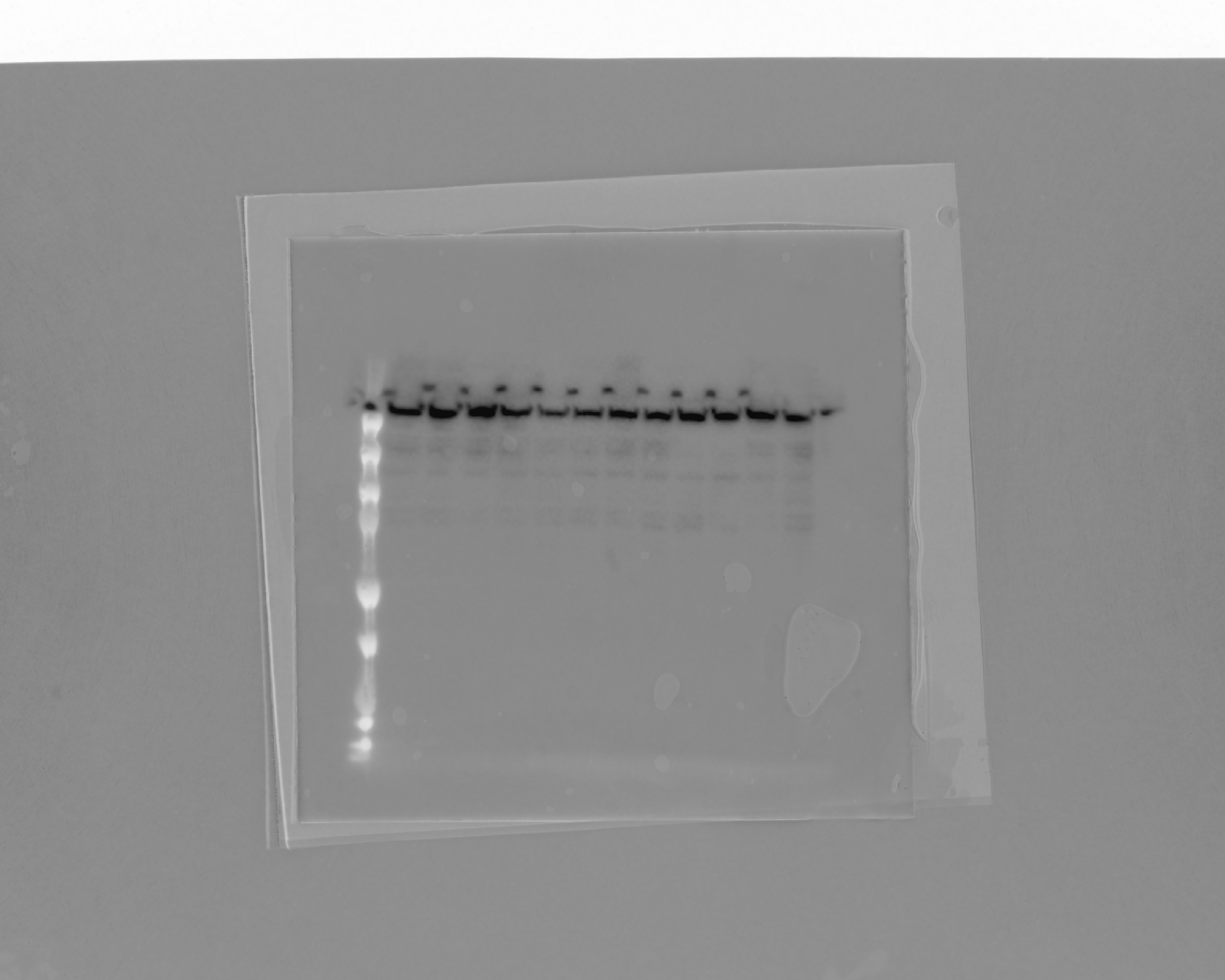

Supplement: Multimedia component 1 [file mmc1.zip › WB bands & raw densitometry/WB bands(45min)/10.(P-)mTOR/T-mTOR/mTOR(Composite).tif]

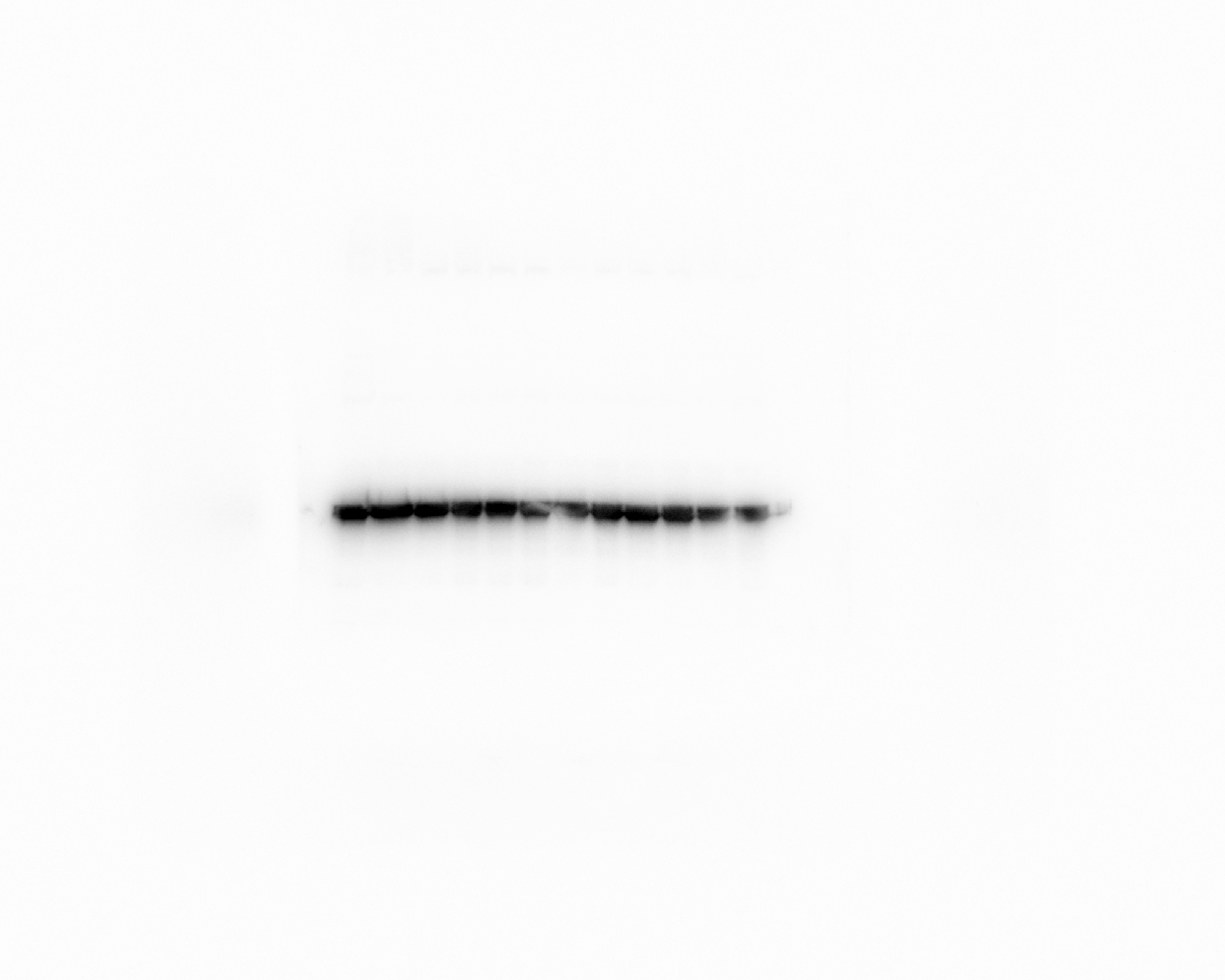

Supplement: Multimedia component 1 [file mmc1.zip › WB bands & raw densitometry/WB bands(45min)/11.(P-)ACC/GAPDH(ACC)(Chemiluminescence).tif]

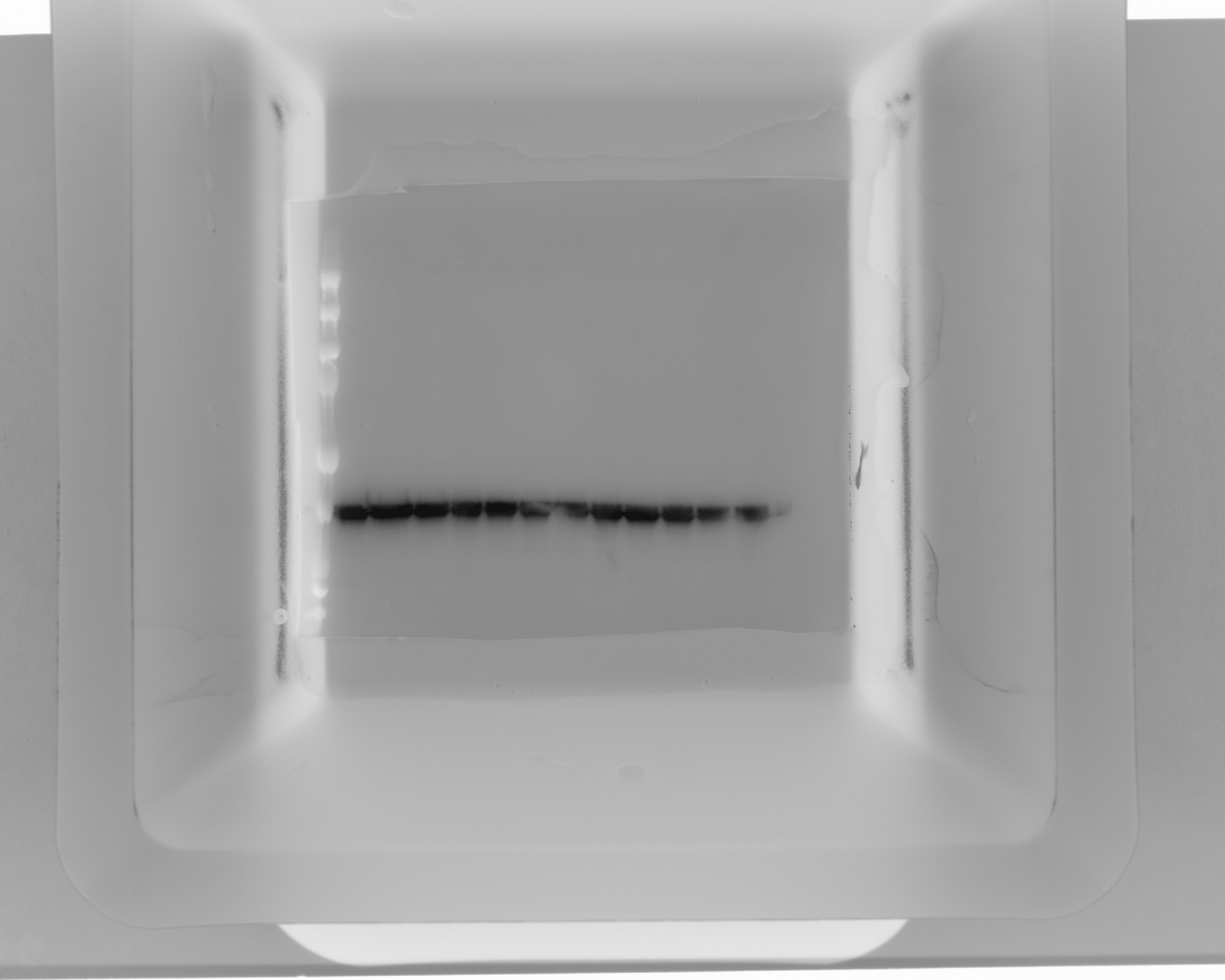

Supplement: Multimedia component 1 [file mmc1.zip › WB bands & raw densitometry/WB bands(45min)/11.(P-)ACC/GAPDH(ACC)(Composite).tif]

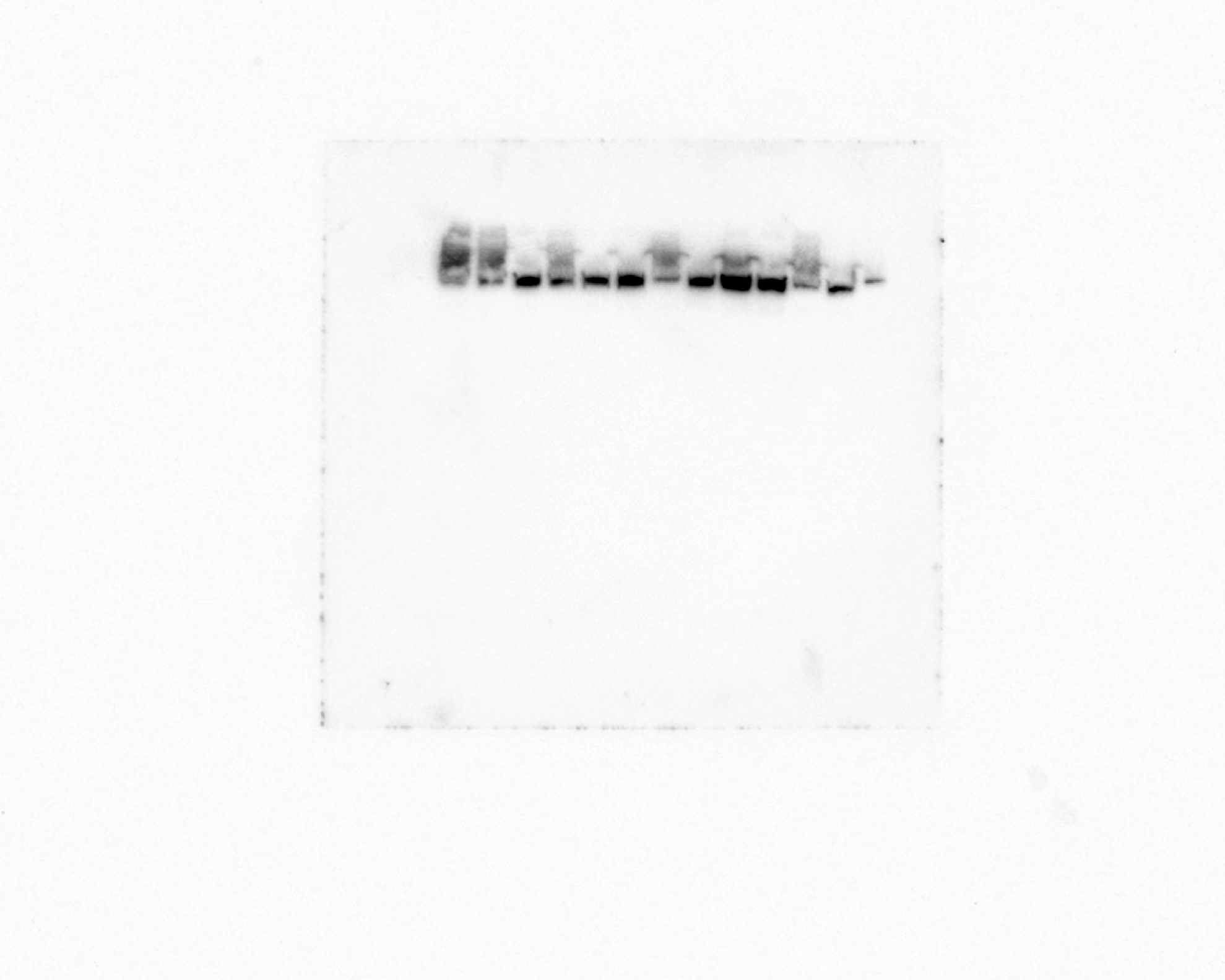

Supplement: Multimedia component 1 [file mmc1.zip › WB bands & raw densitometry/WB bands(45min)/11.(P-)ACC/p-ACC/p-ACC(Chemiluminescence).tif]

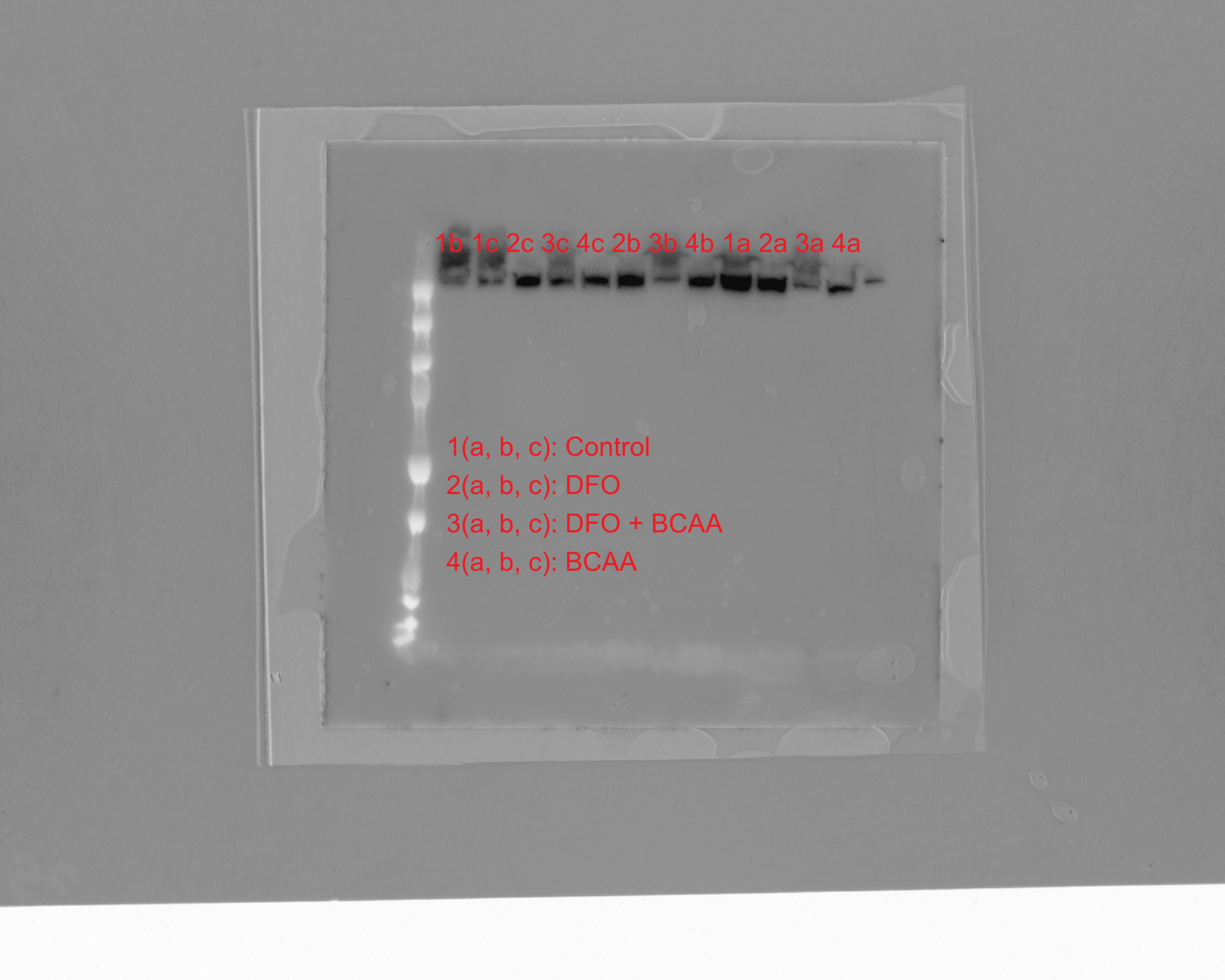

Supplement: Multimedia component 1 [file mmc1.zip › WB bands & raw densitometry/WB bands(45min)/11.(P-)ACC/p-ACC/p-ACC(Composite).tif]

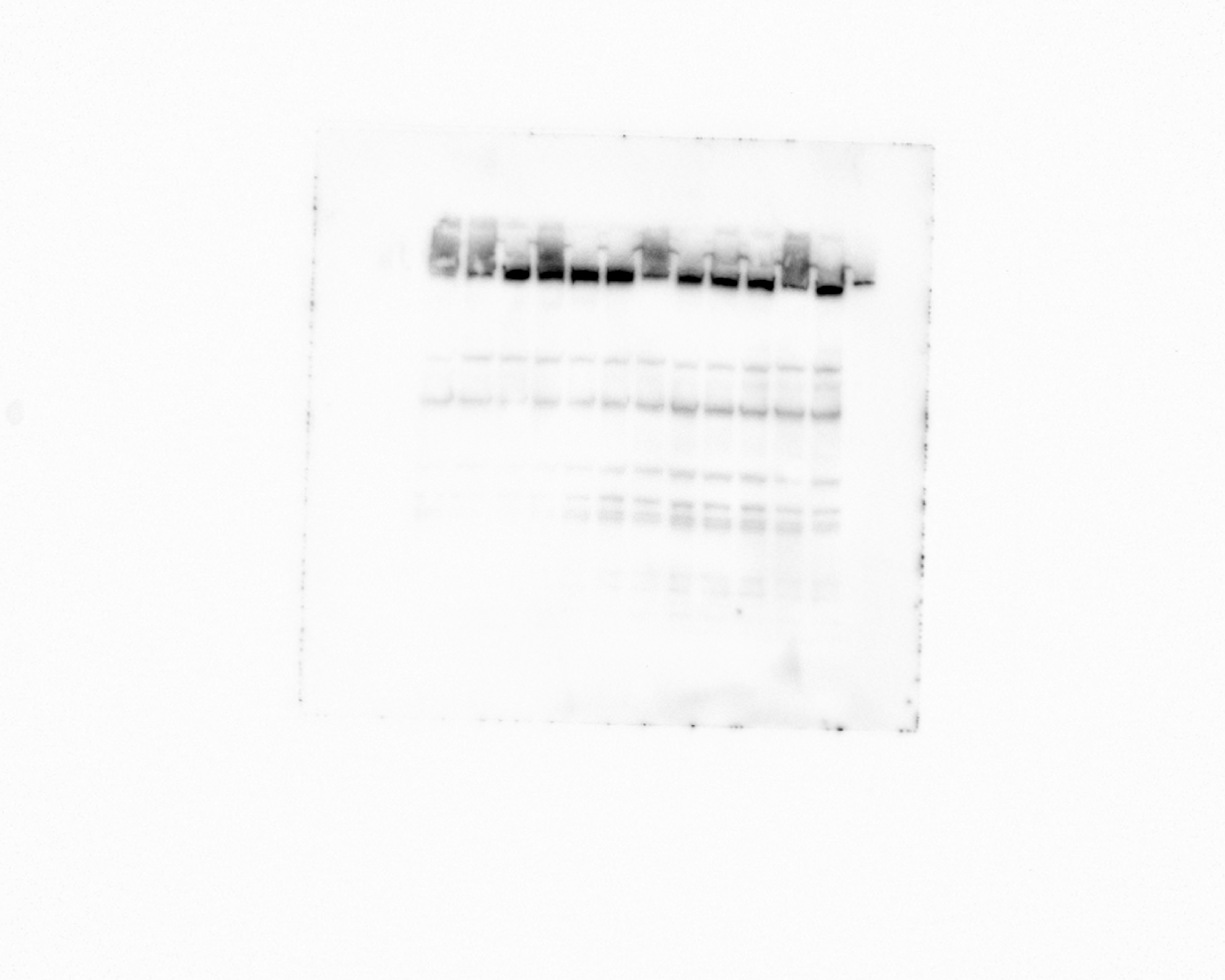

Supplement: Multimedia component 1 [file mmc1.zip › WB bands & raw densitometry/WB bands(45min)/11.(P-)ACC/T-ACC/ACC(Chemiluminescence).tif]

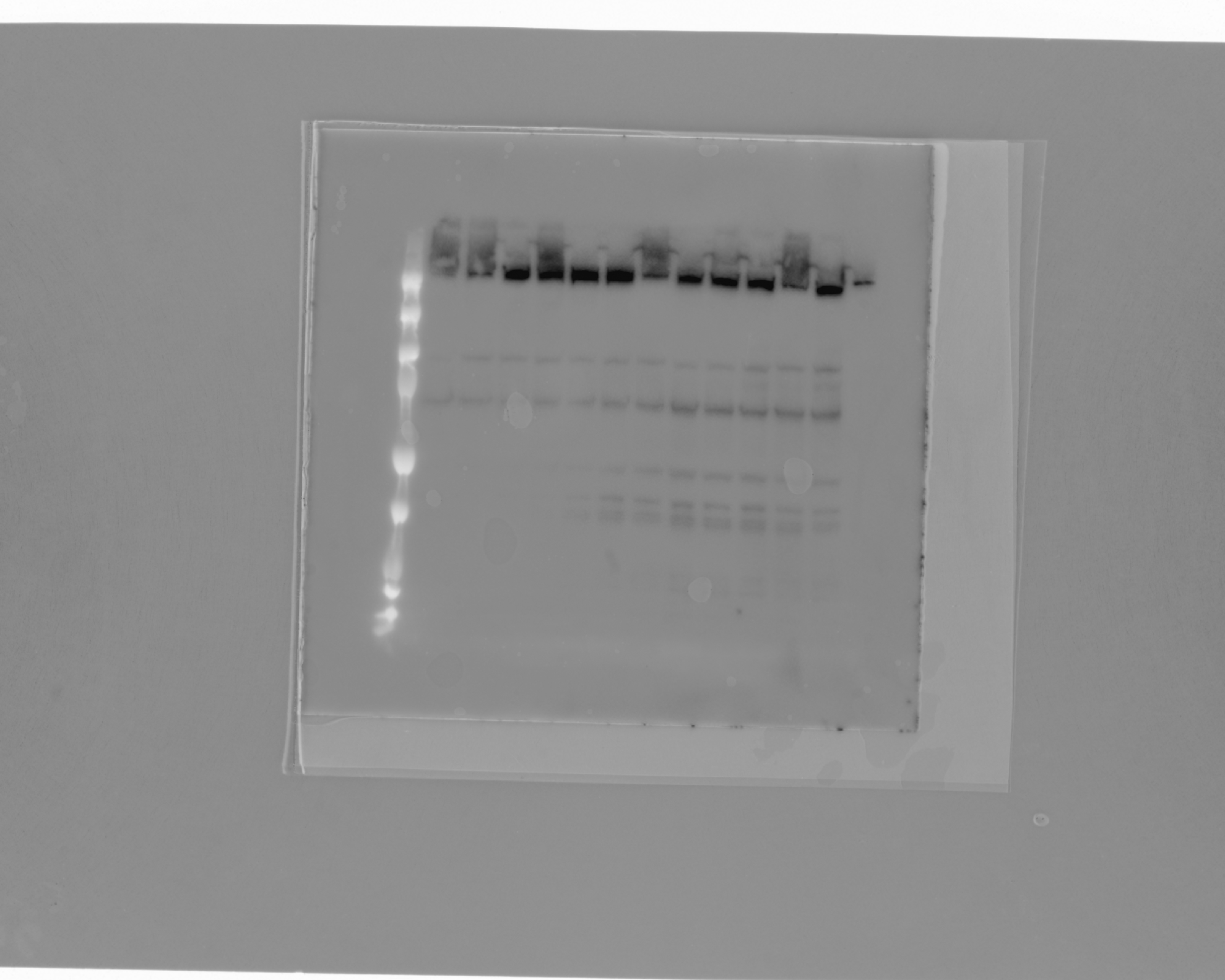

Supplement: Multimedia component 1 [file mmc1.zip › WB bands & raw densitometry/WB bands(45min)/11.(P-)ACC/T-ACC/ACC(Composite).tif]

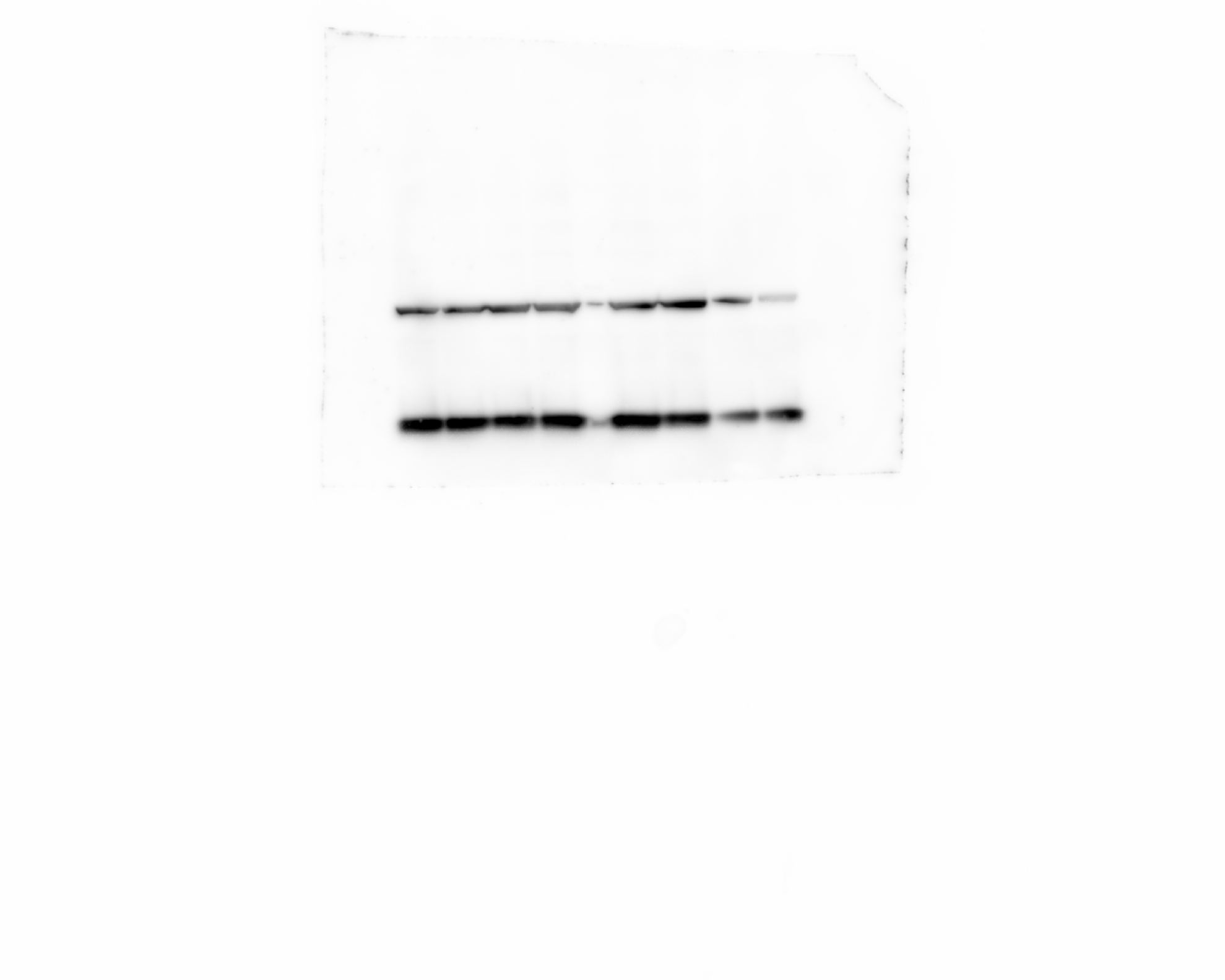

Supplement: Multimedia component 1 [file mmc1.zip › WB bands & raw densitometry/WB bands(45min)/3.(P-)S6K/B-actin(S6K)(1)-1(Chemiluminescence).tif]

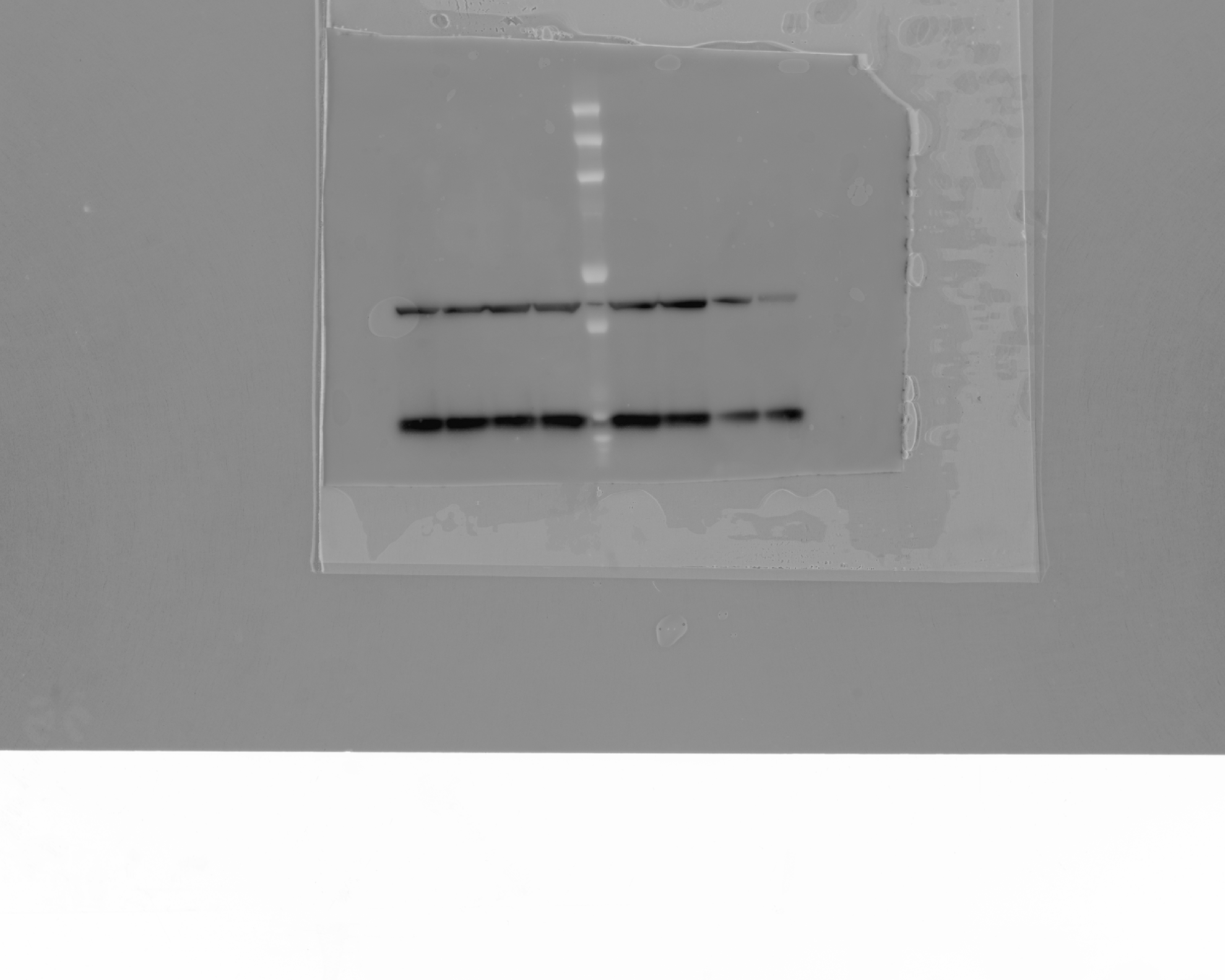

Supplement: Multimedia component 1 [file mmc1.zip › WB bands & raw densitometry/WB bands(45min)/3.(P-)S6K/B-actin(S6K)(1)-1(Composite).tif]

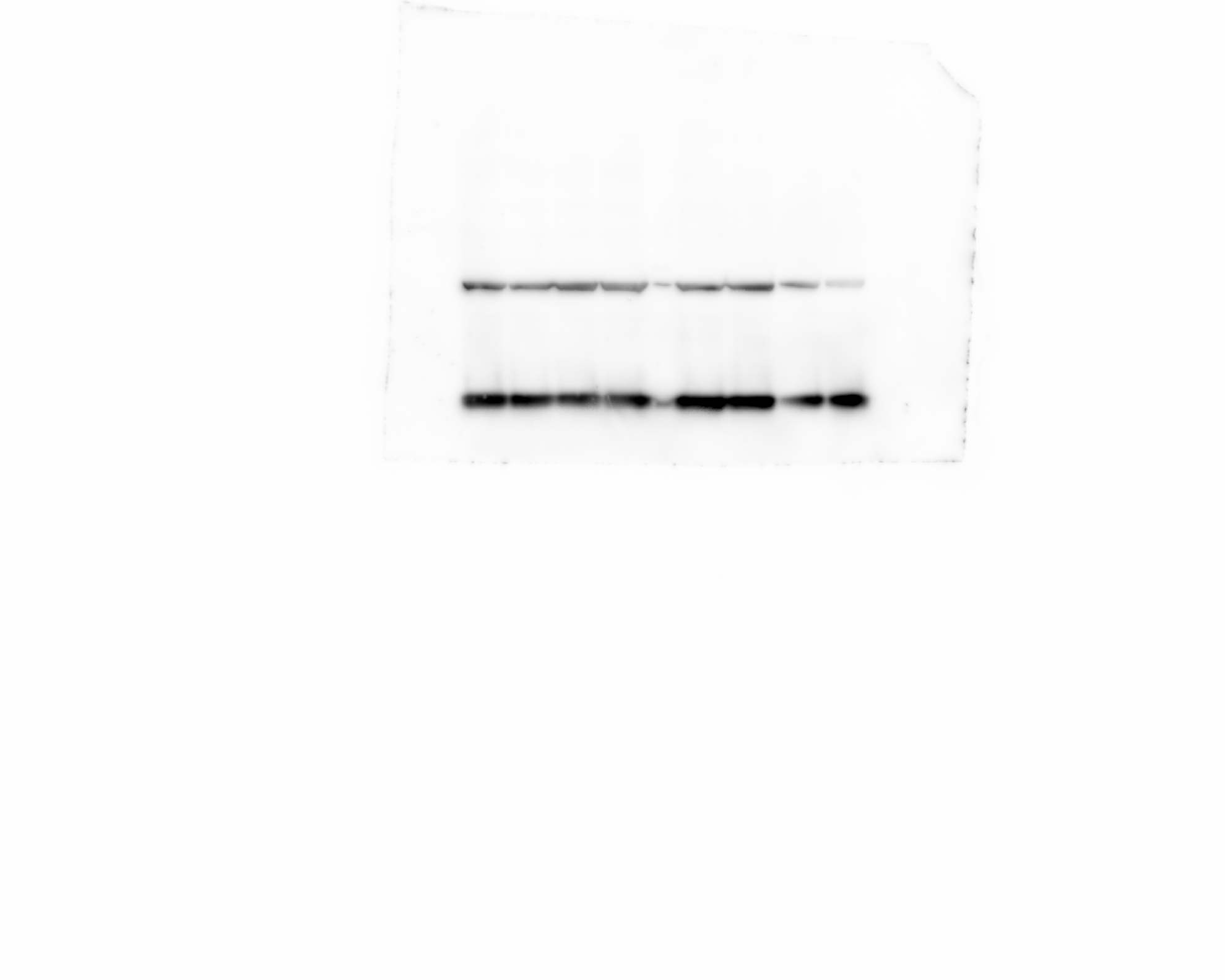

Supplement: Multimedia component 1 [file mmc1.zip › WB bands & raw densitometry/WB bands(45min)/3.(P-)S6K/B-actin(S6K)(1)-2(Chemiluminescence).tif]

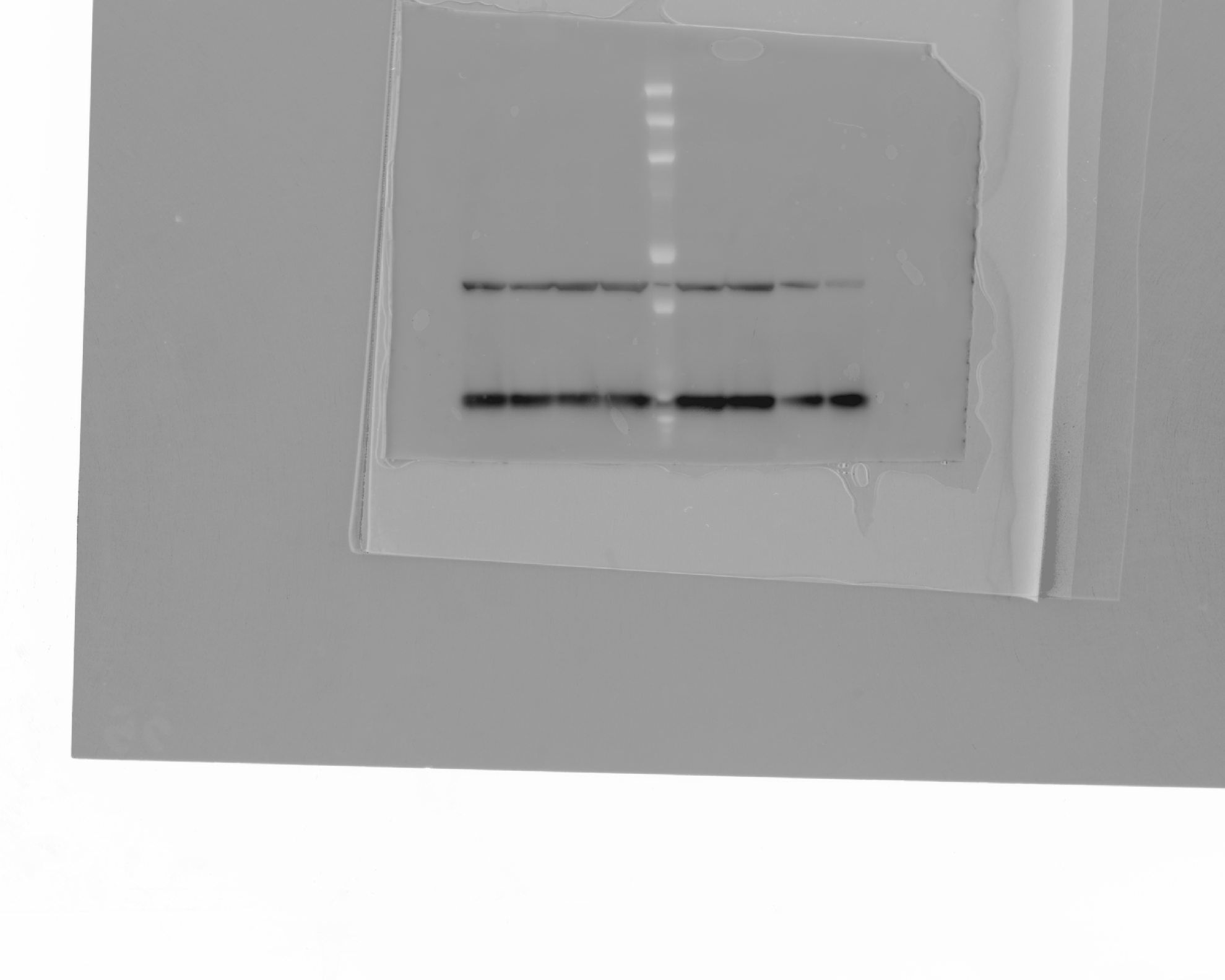

Supplement: Multimedia component 1 [file mmc1.zip › WB bands & raw densitometry/WB bands(45min)/3.(P-)S6K/B-actin(S6K)(1)-2(Composite).tif]

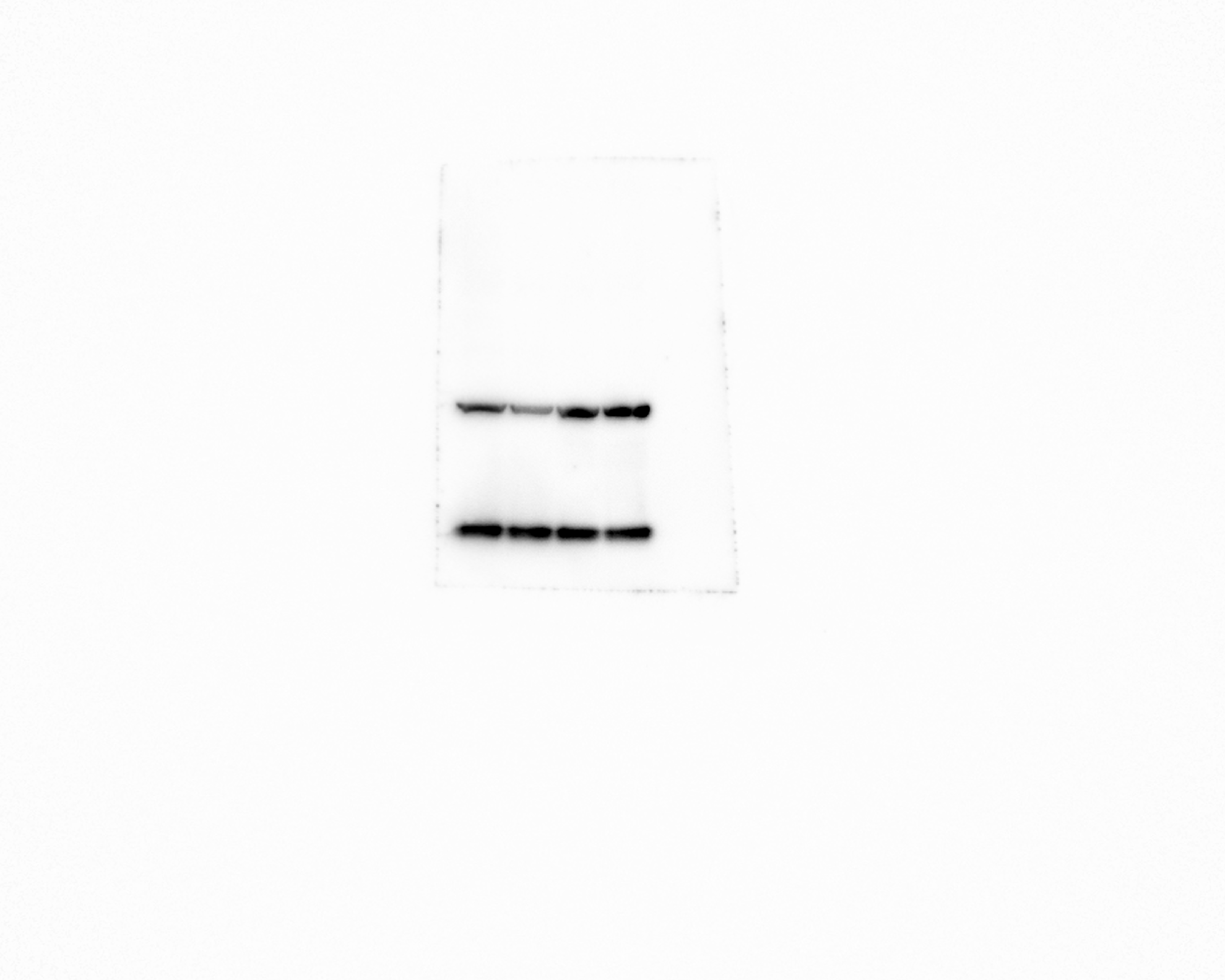

Supplement: Multimedia component 1 [file mmc1.zip › WB bands & raw densitometry/WB bands(45min)/3.(P-)S6K/B-actin(S6K)(2)(Chemiluminescence).tif]

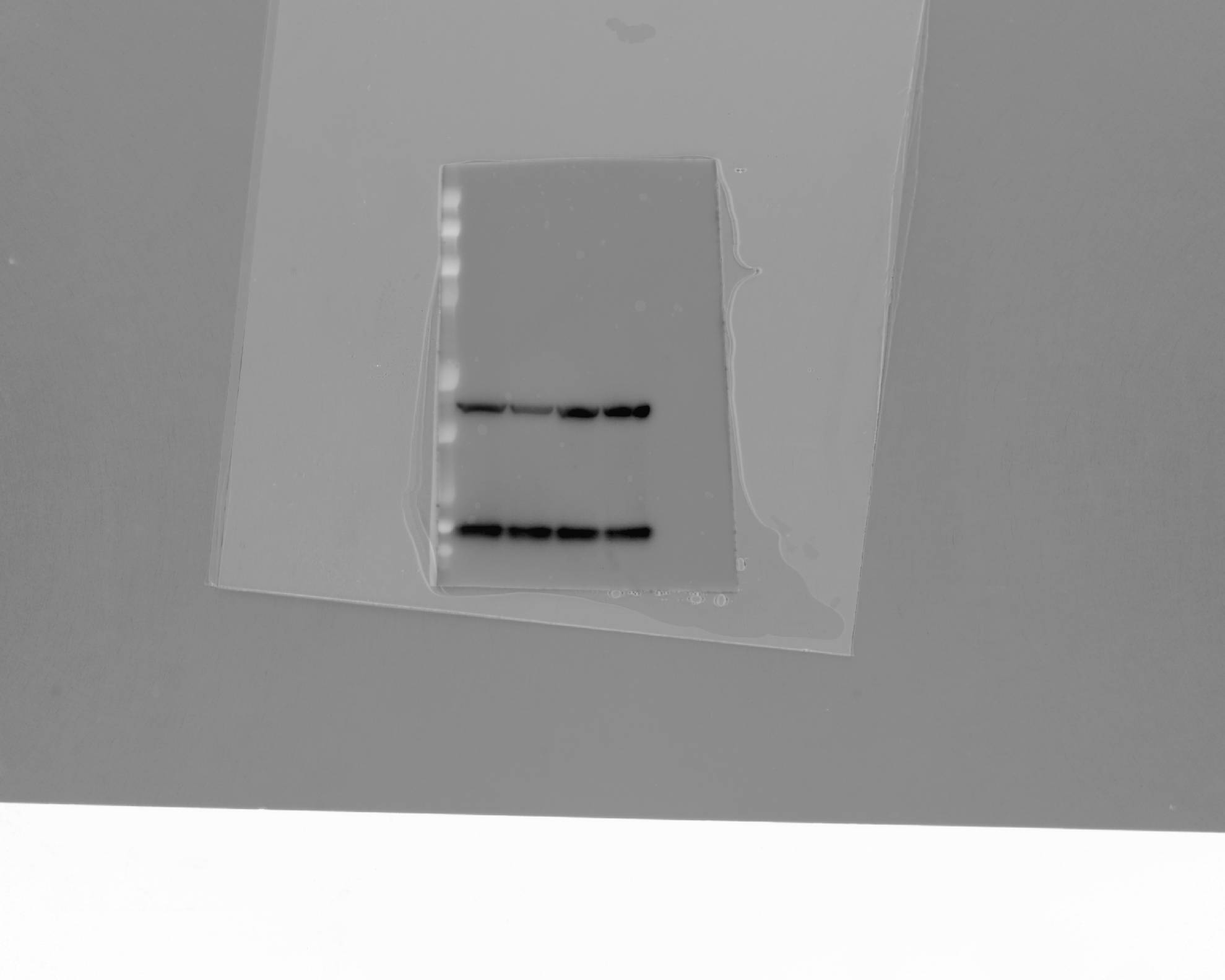

Supplement: Multimedia component 1 [file mmc1.zip › WB bands & raw densitometry/WB bands(45min)/3.(P-)S6K/B-actin(S6K)(2)(Composite).tif]

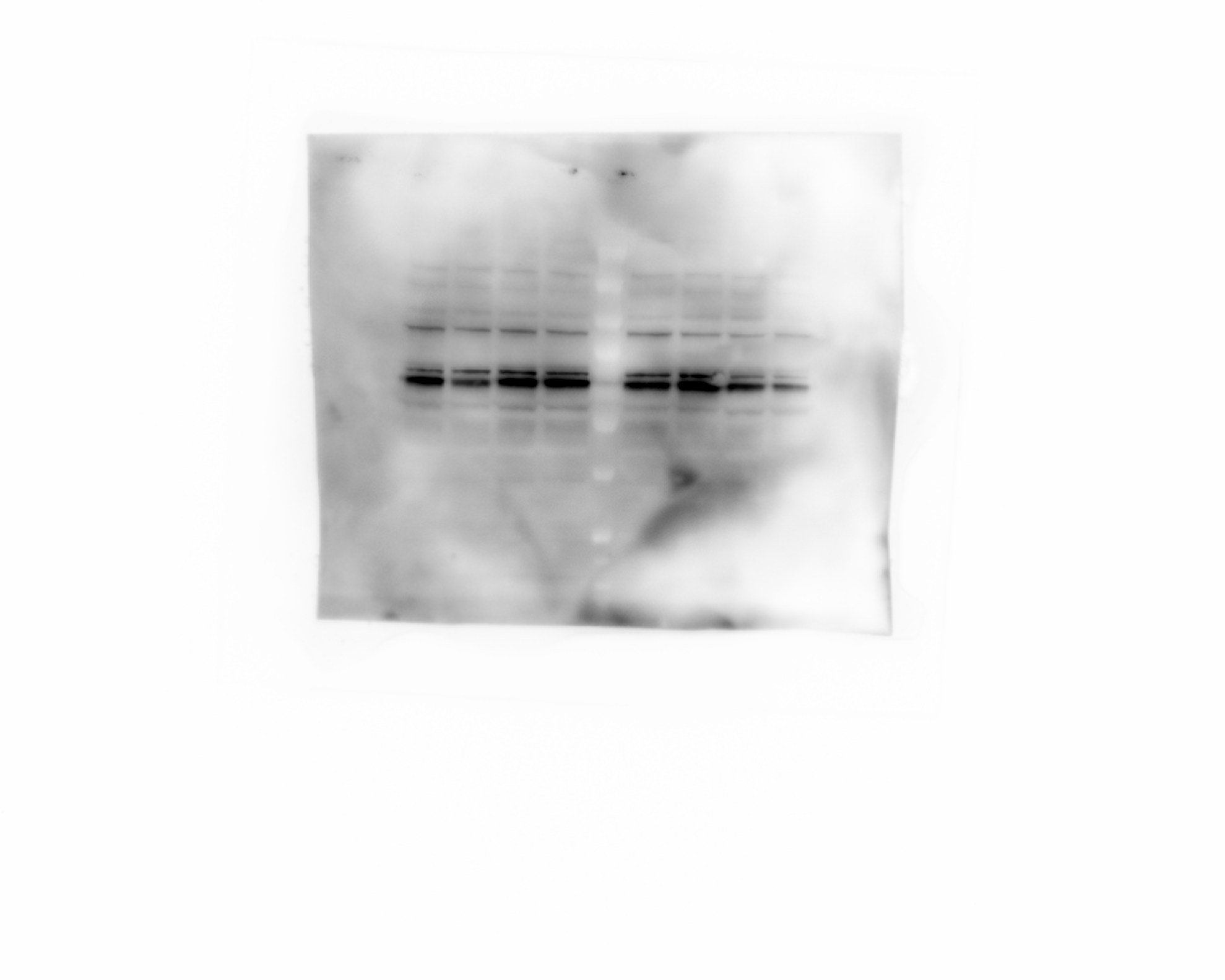

Supplement: Multimedia component 1 [file mmc1.zip › WB bands & raw densitometry/WB bands(45min)/3.(P-)S6K/User 2025-09-17 45min p-S6K(1)(Chemiluminescence).tif]

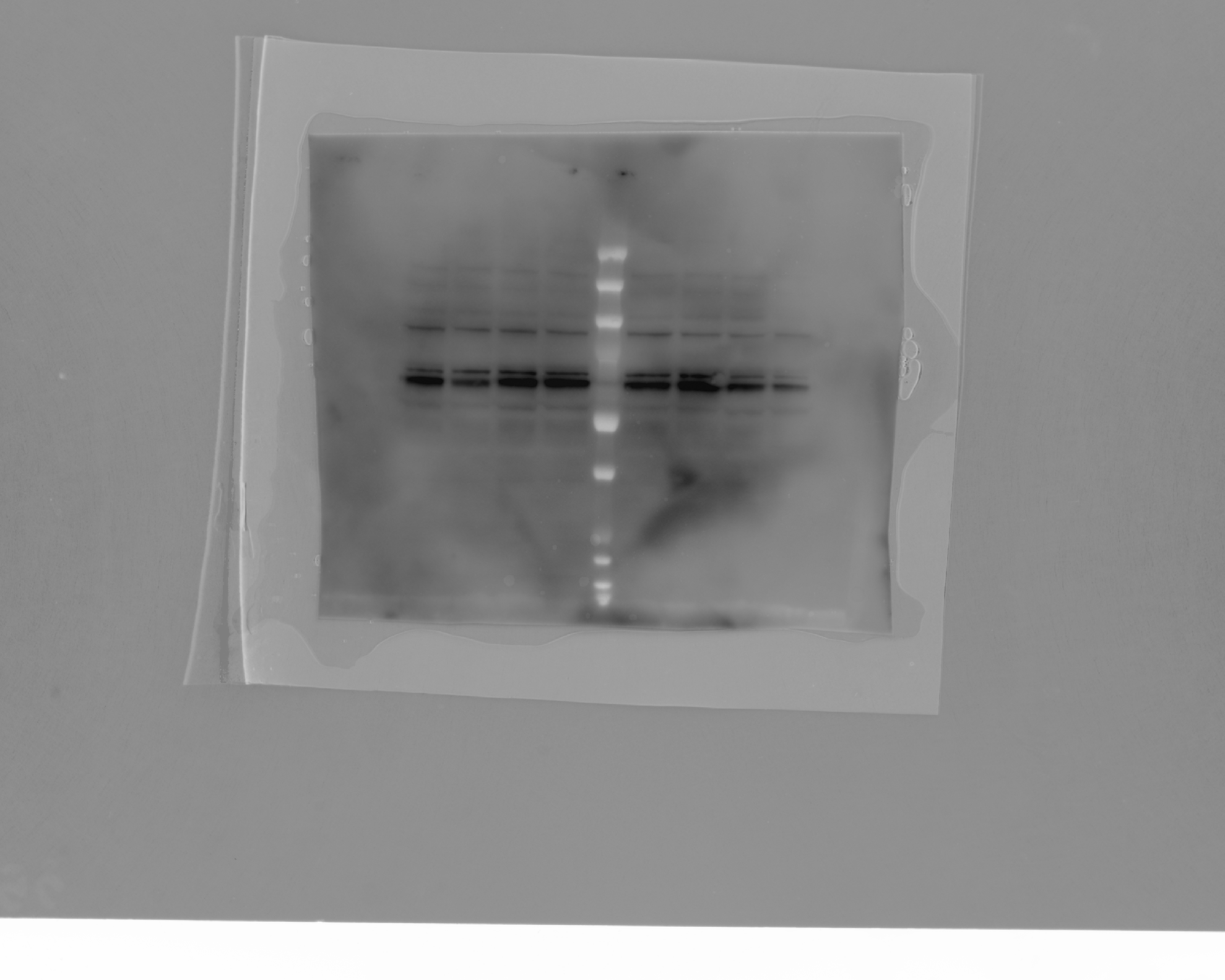

Supplement: Multimedia component 1 [file mmc1.zip › WB bands & raw densitometry/WB bands(45min)/3.(P-)S6K/User 2025-09-17 45min p-S6K(1)(Composite).tif]

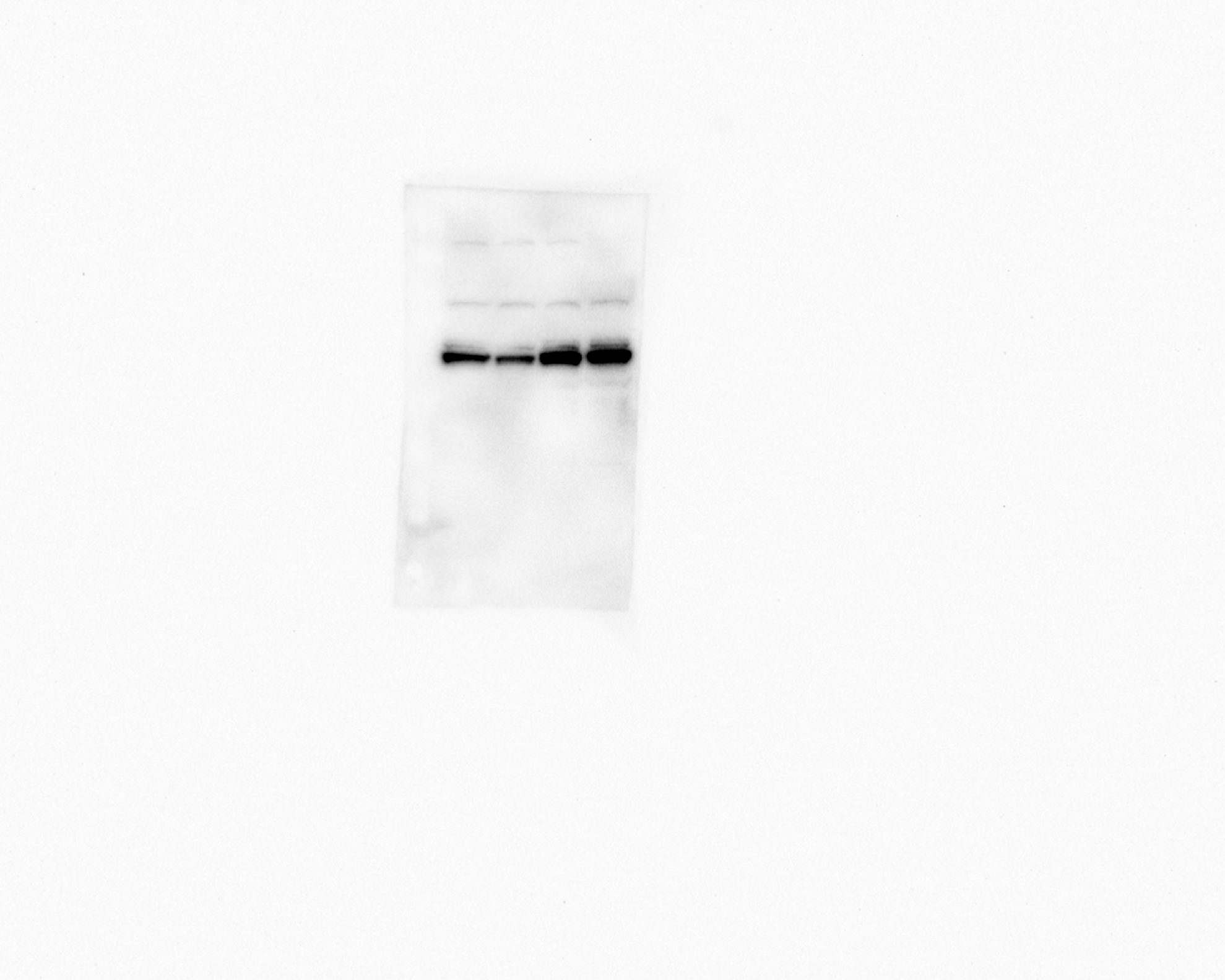

Supplement: Multimedia component 1 [file mmc1.zip › WB bands & raw densitometry/WB bands(45min)/3.(P-)S6K/User 2025-09-17 45min p-S6K(2)(Chemiluminescence).tif]

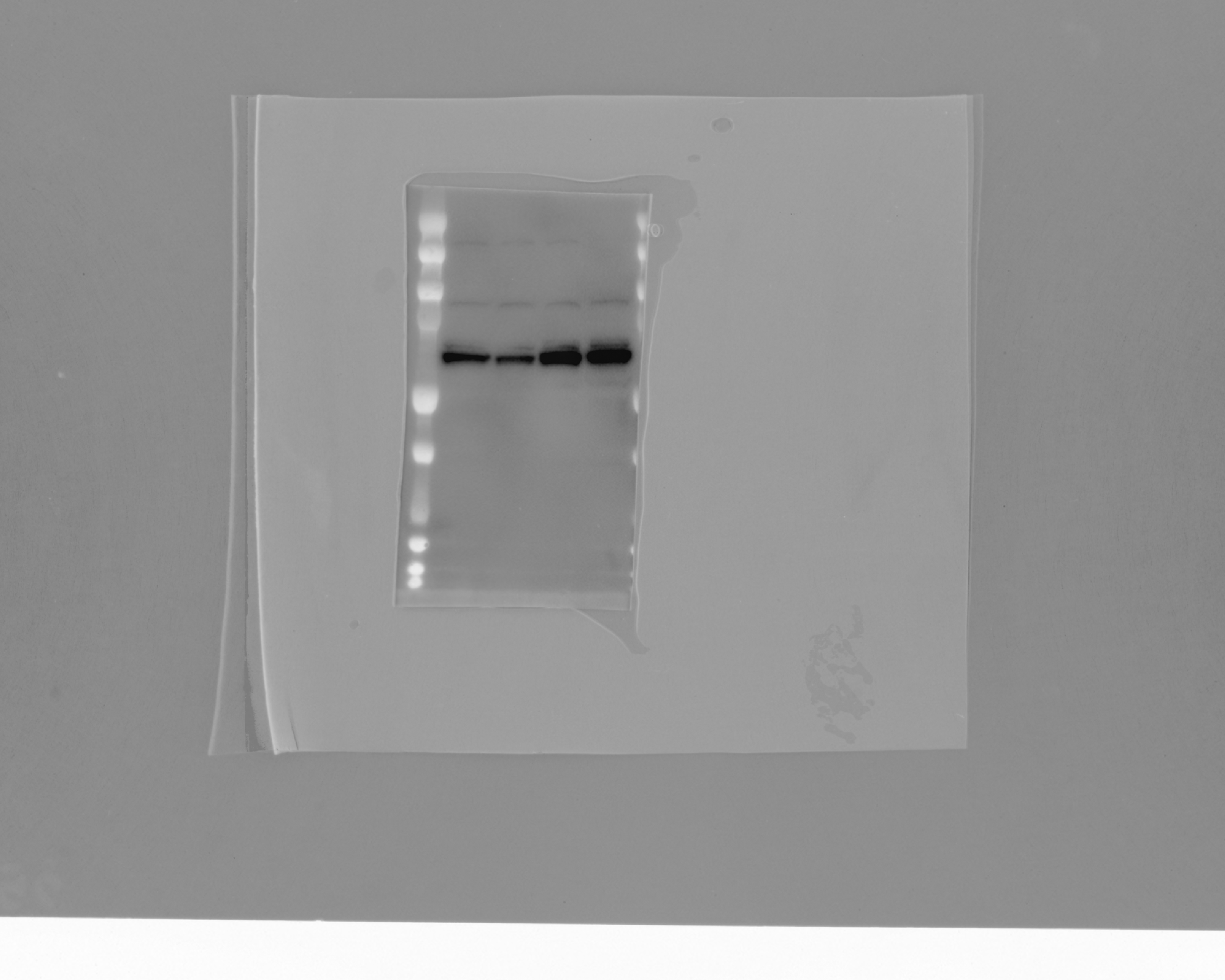

Supplement: Multimedia component 1 [file mmc1.zip › WB bands & raw densitometry/WB bands(45min)/3.(P-)S6K/User 2025-09-17 45min p-S6K(2)(Composite).tif]

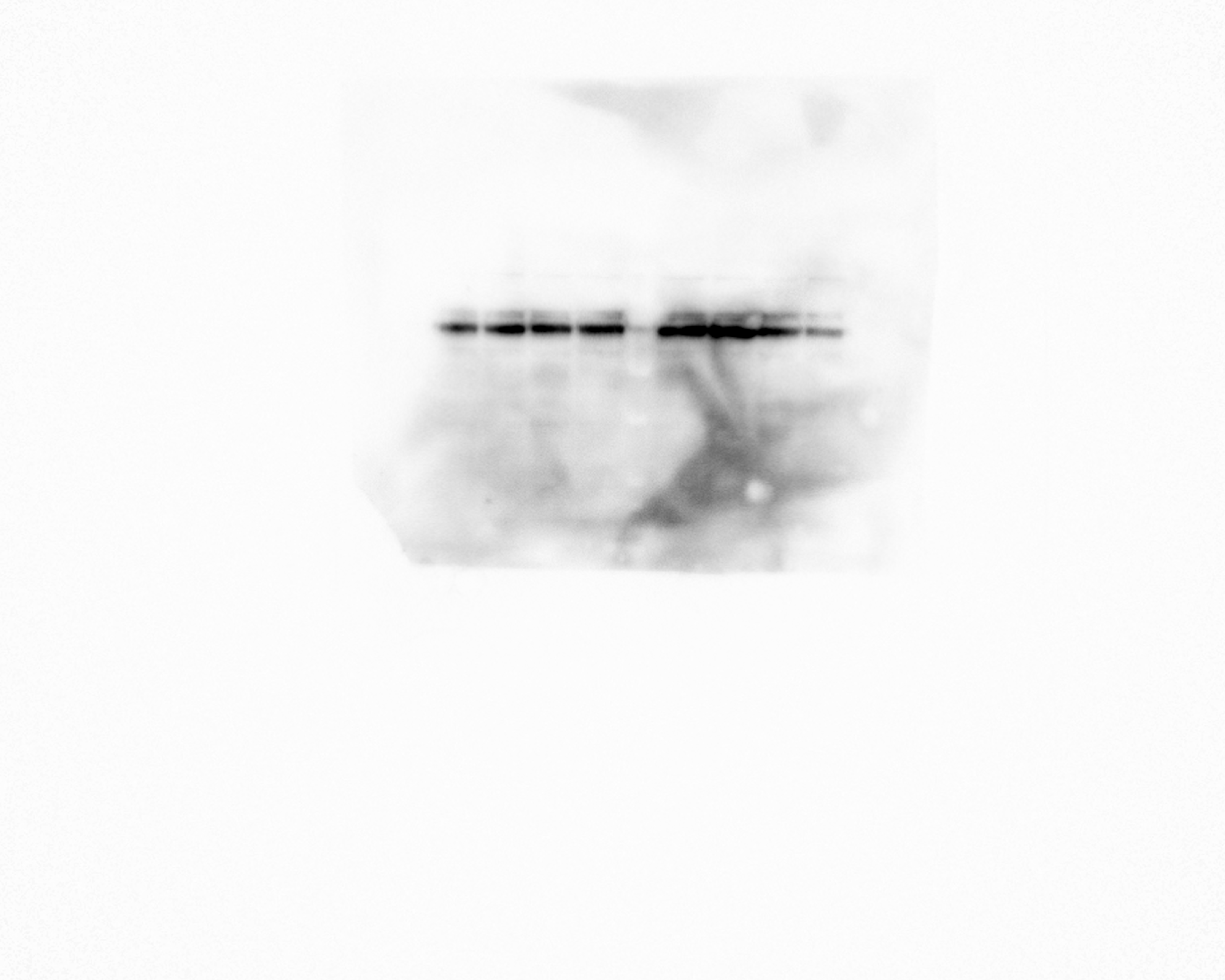

Supplement: Multimedia component 1 [file mmc1.zip › WB bands & raw densitometry/WB bands(45min)/3.(P-)S6K/User 2025-09-18 S6K(1)(Chemiluminescence).tif]

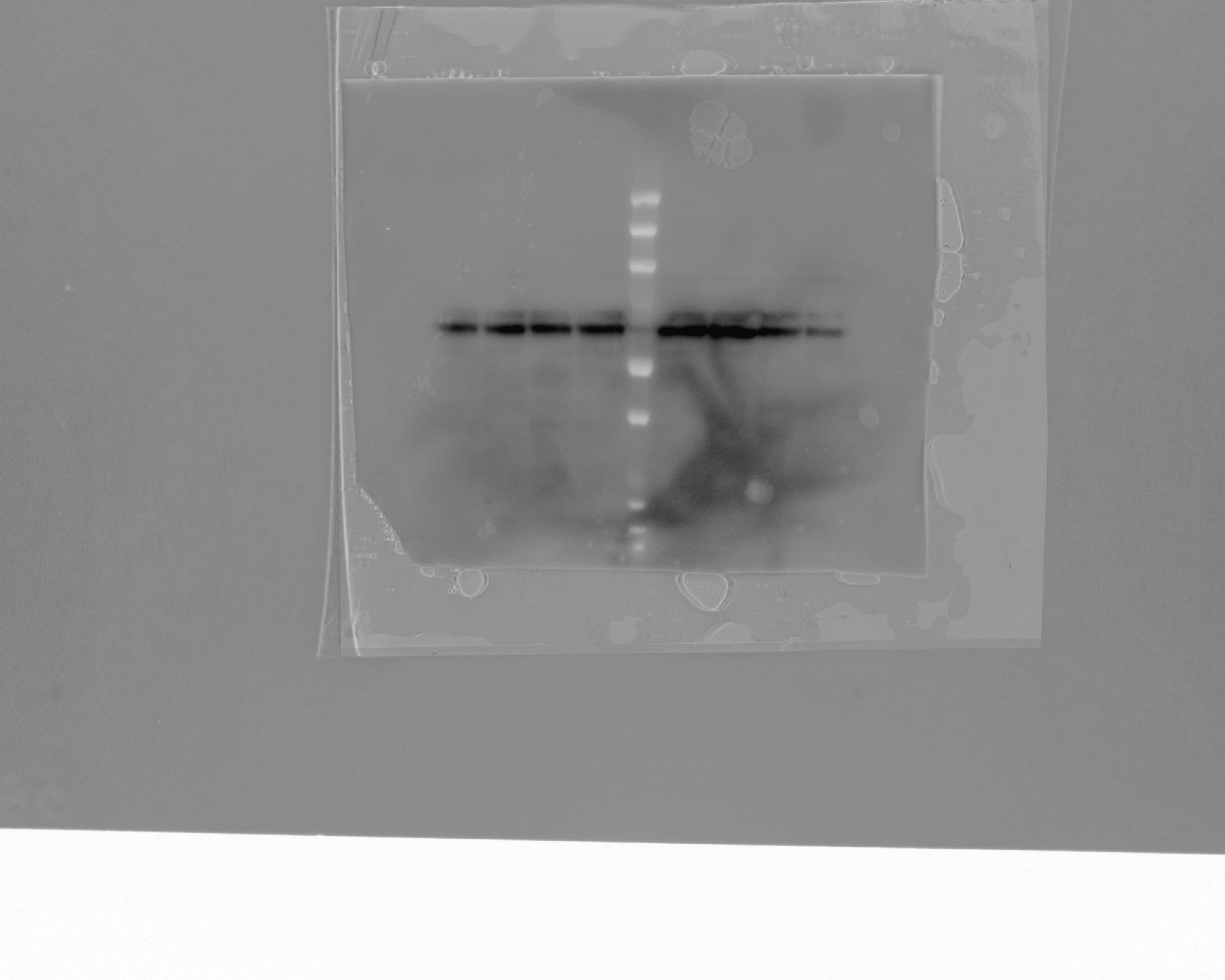

Supplement: Multimedia component 1 [file mmc1.zip › WB bands & raw densitometry/WB bands(45min)/3.(P-)S6K/User 2025-09-18 S6K(1)(Composite).tif]

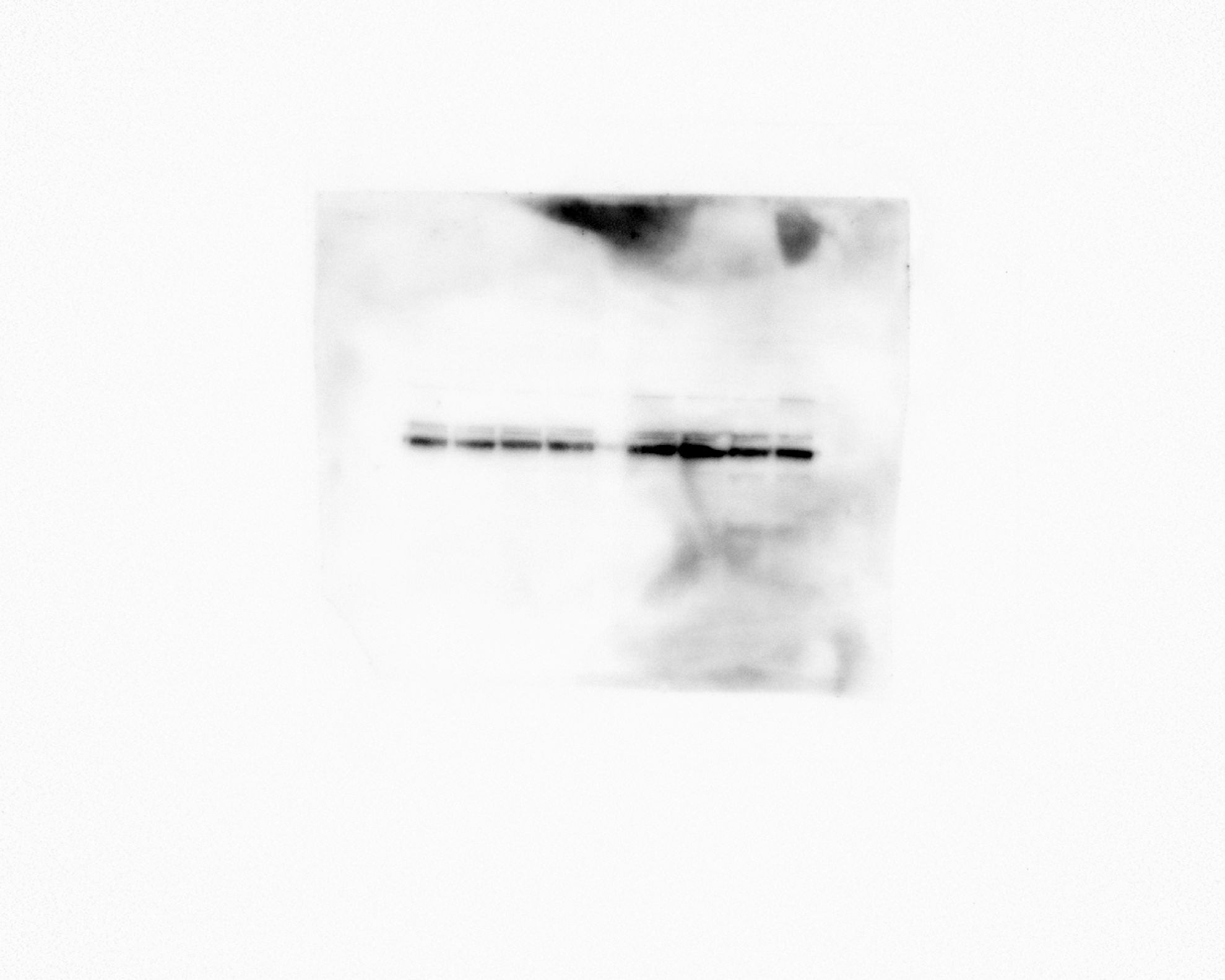

Supplement: Multimedia component 1 [file mmc1.zip › WB bands & raw densitometry/WB bands(45min)/3.(P-)S6K/User 2025-09-18 S6K(1)-1(Chemiluminescence).tif]

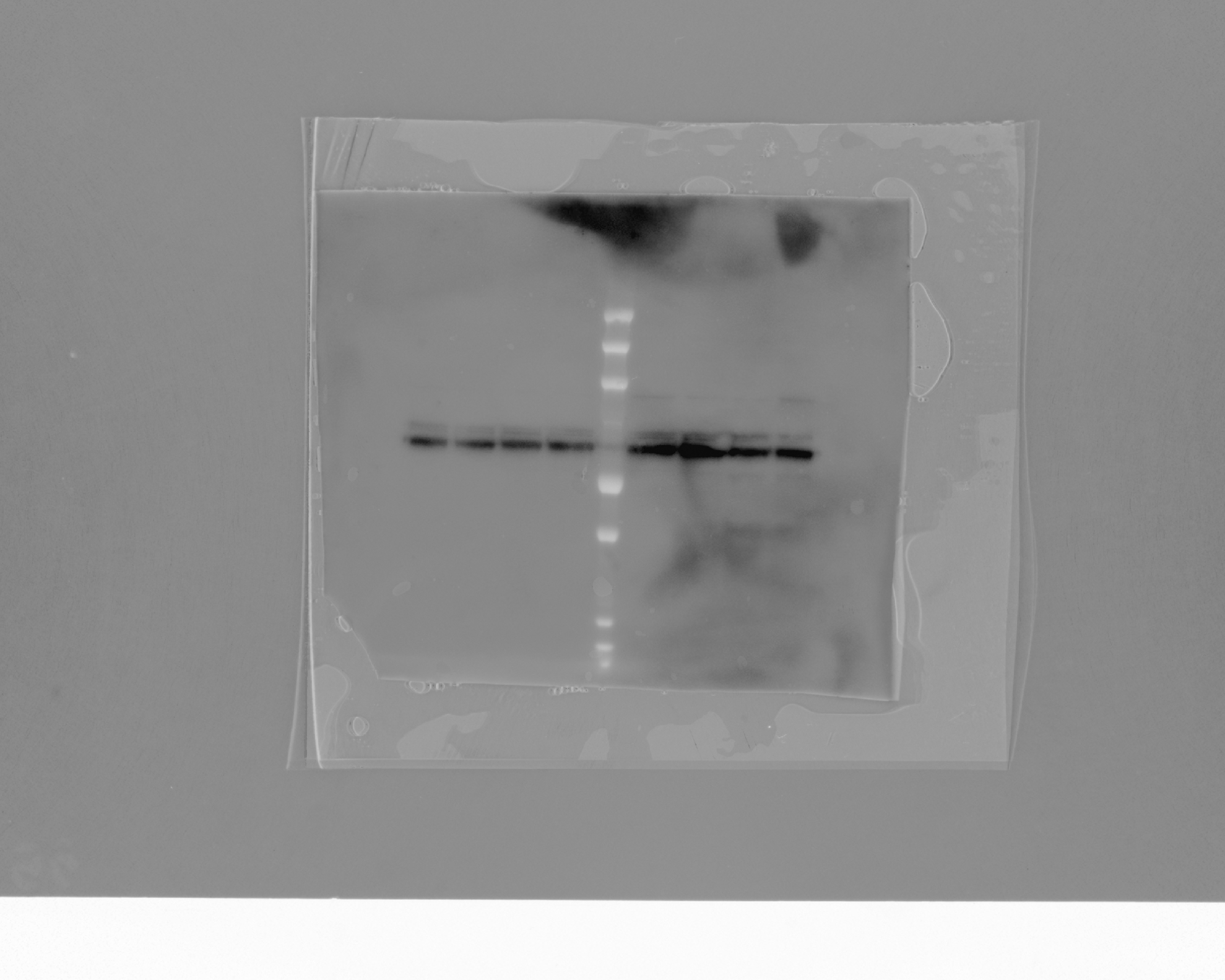

Supplement: Multimedia component 1 [file mmc1.zip › WB bands & raw densitometry/WB bands(45min)/3.(P-)S6K/User 2025-09-18 S6K(1)-1(Composite).tif]

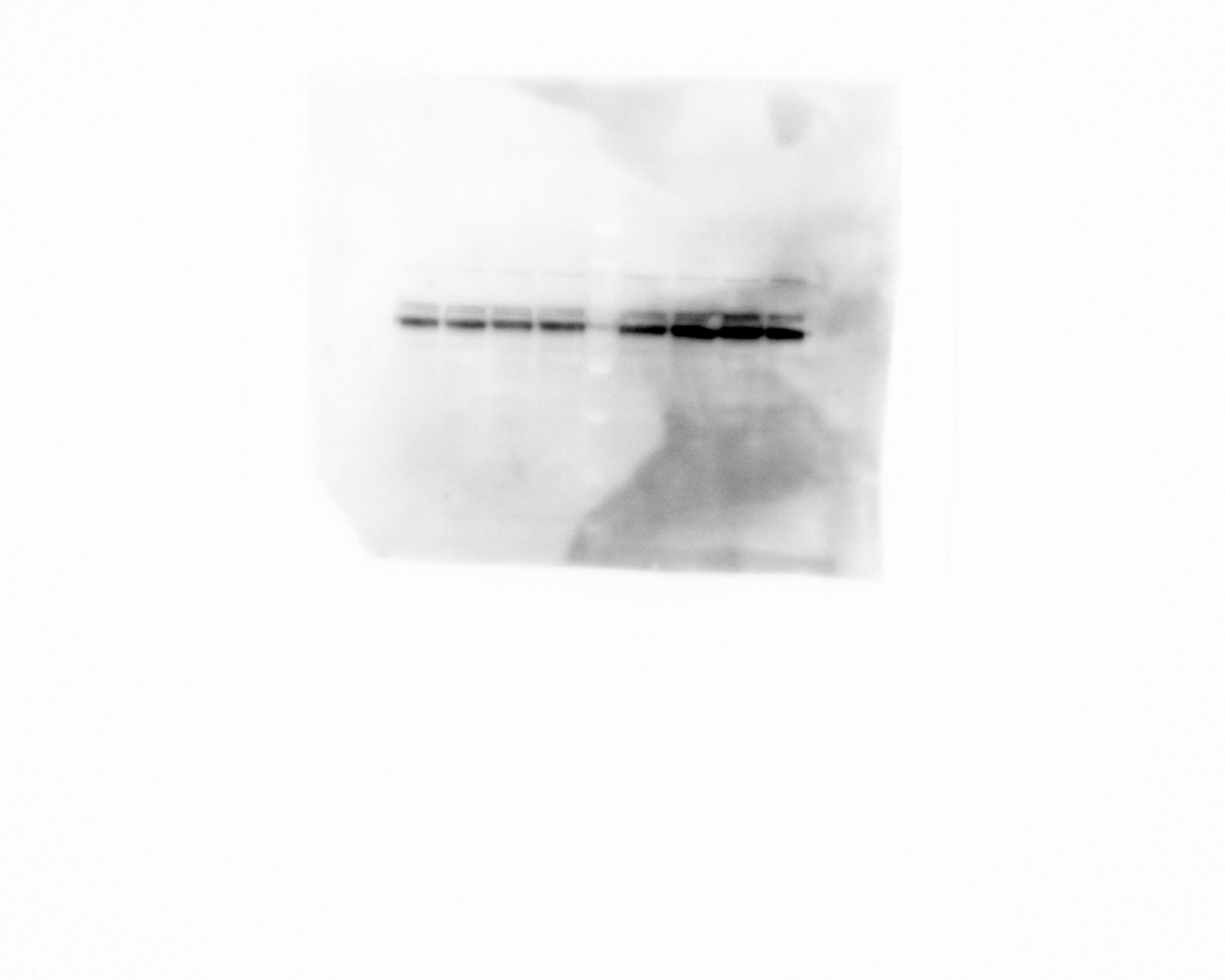

Supplement: Multimedia component 1 [file mmc1.zip › WB bands & raw densitometry/WB bands(45min)/3.(P-)S6K/User 2025-09-18 S6K(1)-3(Chemiluminescence).tif]

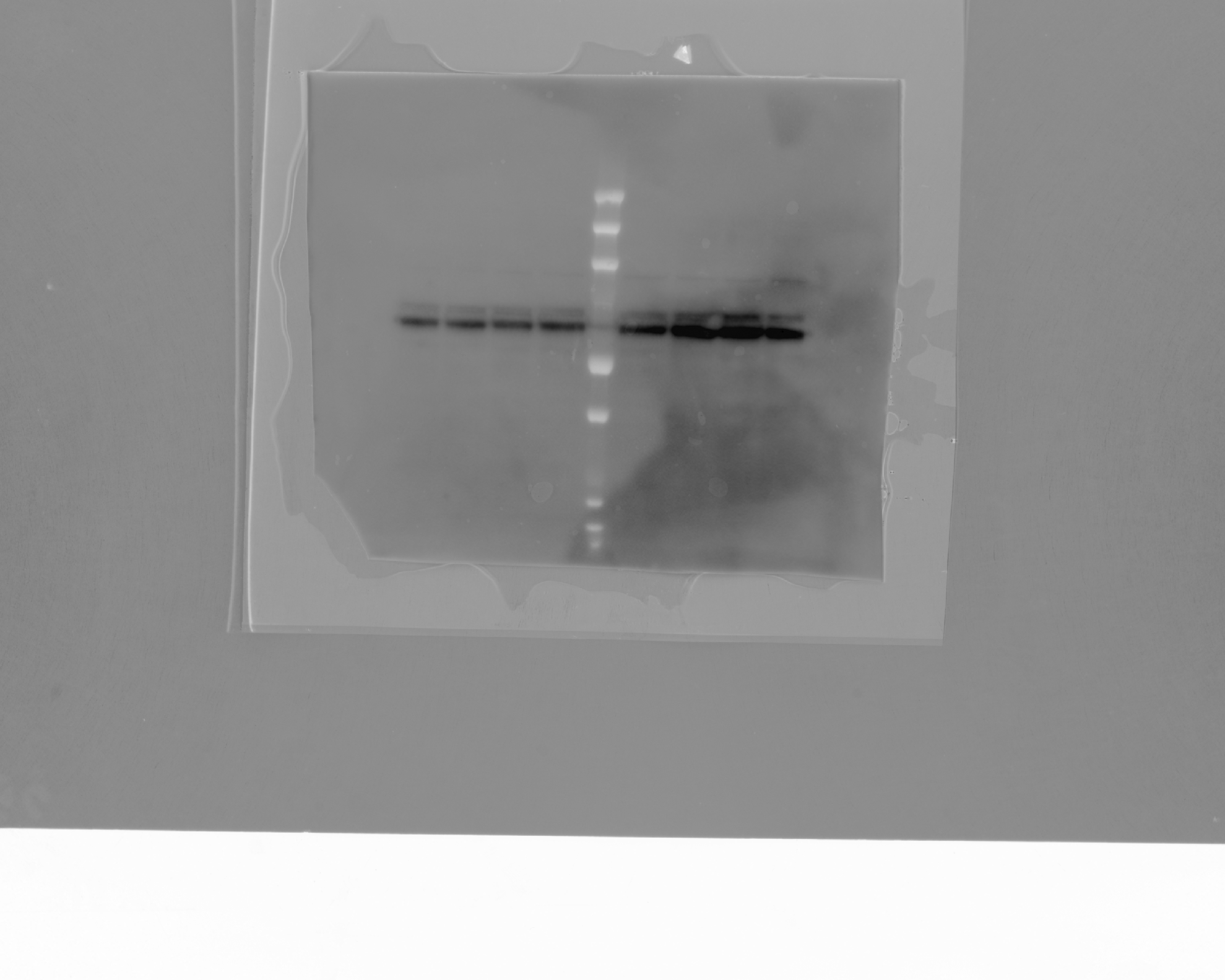

Supplement: Multimedia component 1 [file mmc1.zip › WB bands & raw densitometry/WB bands(45min)/3.(P-)S6K/User 2025-09-18 S6K(1)-3(Composite).tif]

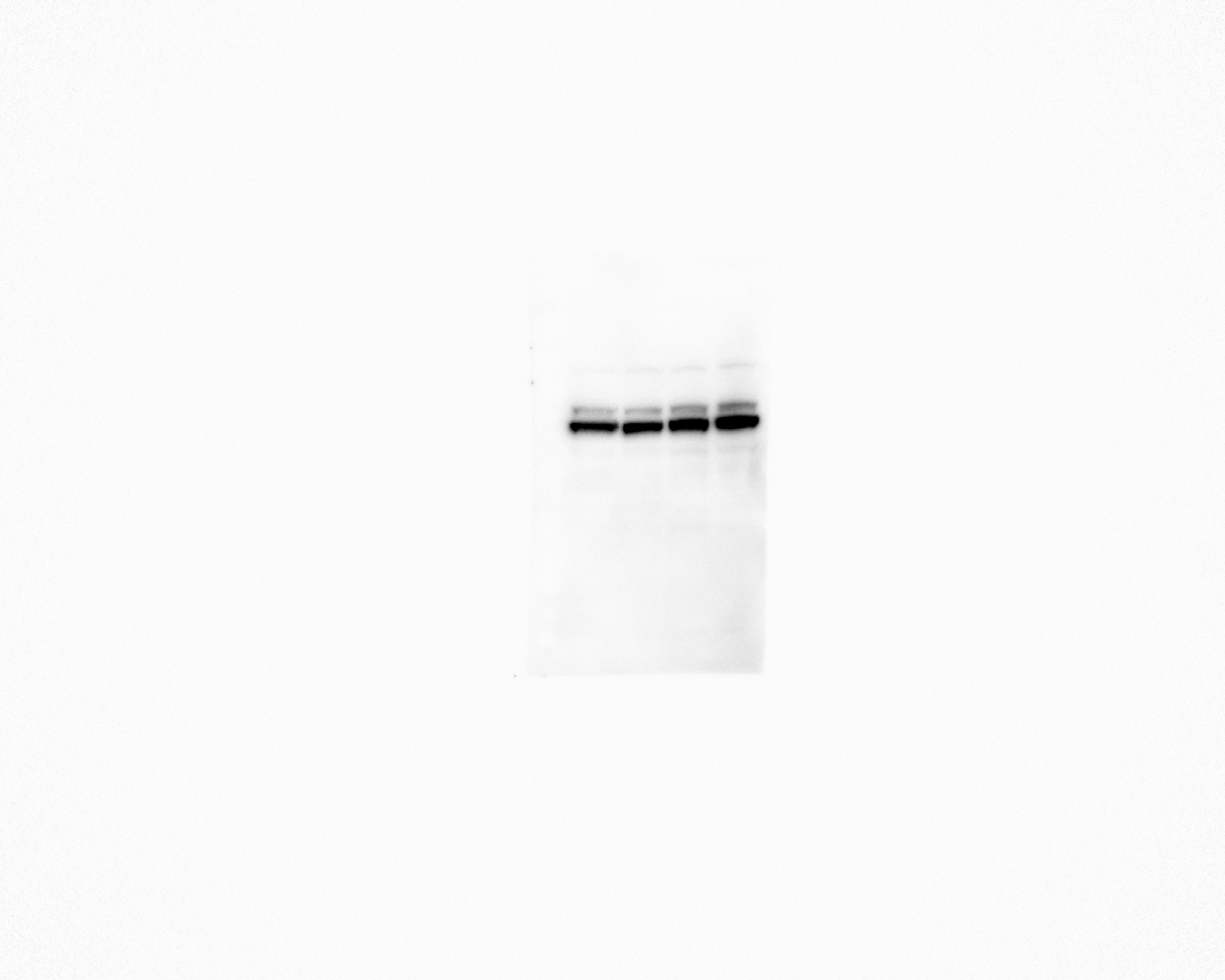

Supplement: Multimedia component 1 [file mmc1.zip › WB bands & raw densitometry/WB bands(45min)/3.(P-)S6K/User 2025-09-18 S6K(2)(Chemiluminescence).tif]

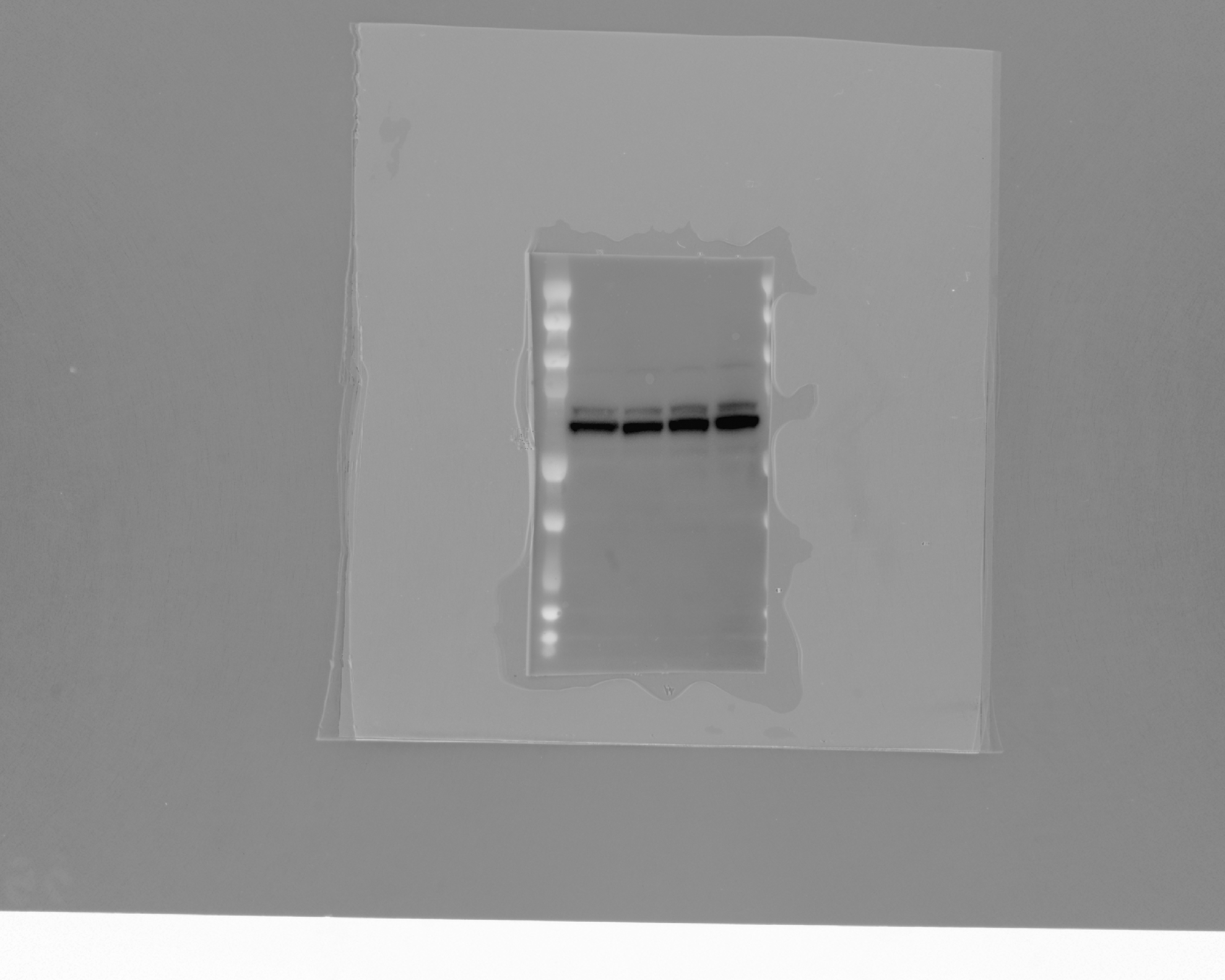

Supplement: Multimedia component 1 [file mmc1.zip › WB bands & raw densitometry/WB bands(45min)/3.(P-)S6K/User 2025-09-18 S6K(2)(Composite).tif]

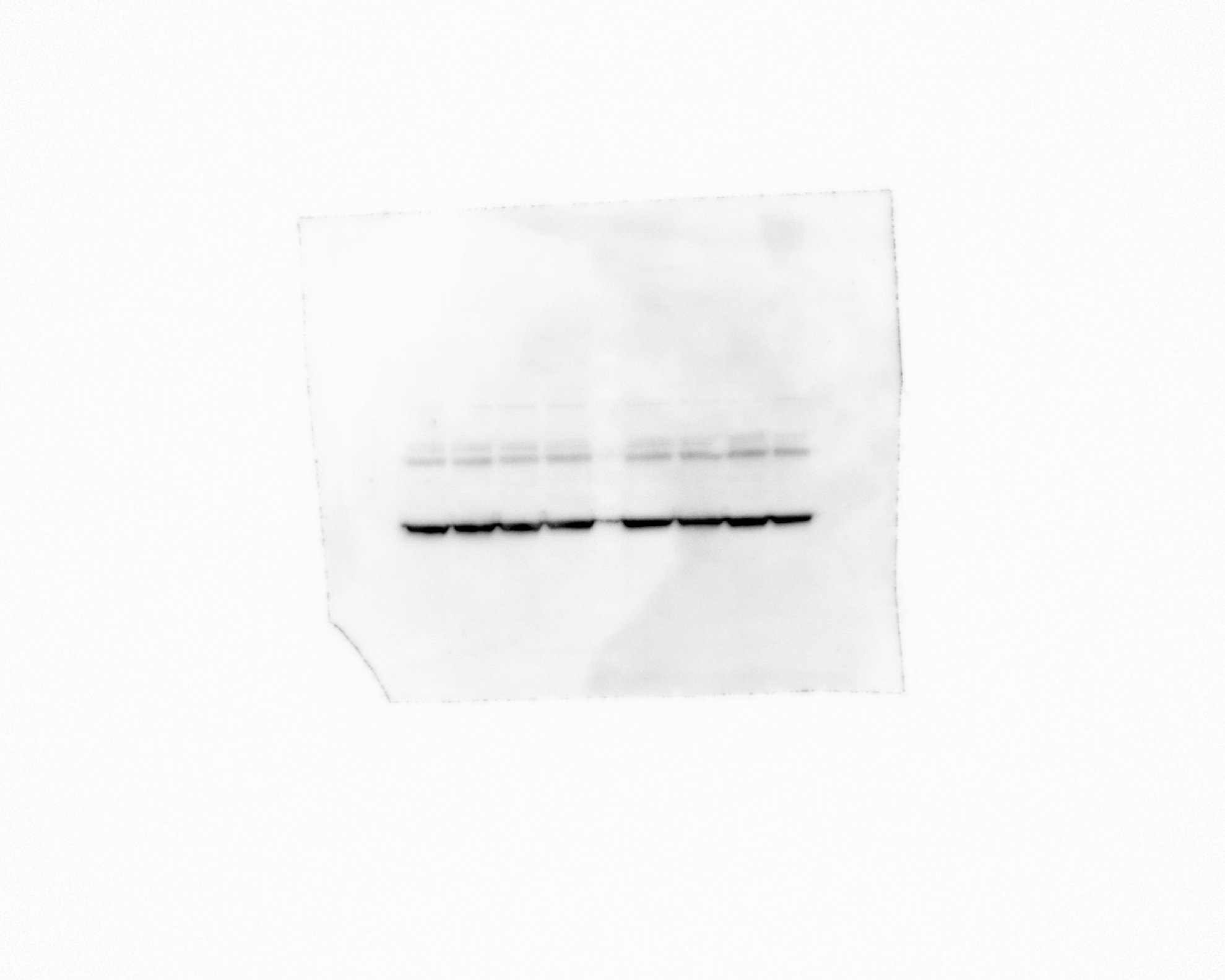

Supplement: Multimedia component 1 [file mmc1.zip › WB bands & raw densitometry/WB bands(45min)/4.(P-)4E-BP1/B-actin(4EBP1)(1)(Chemiluminescence).tif]

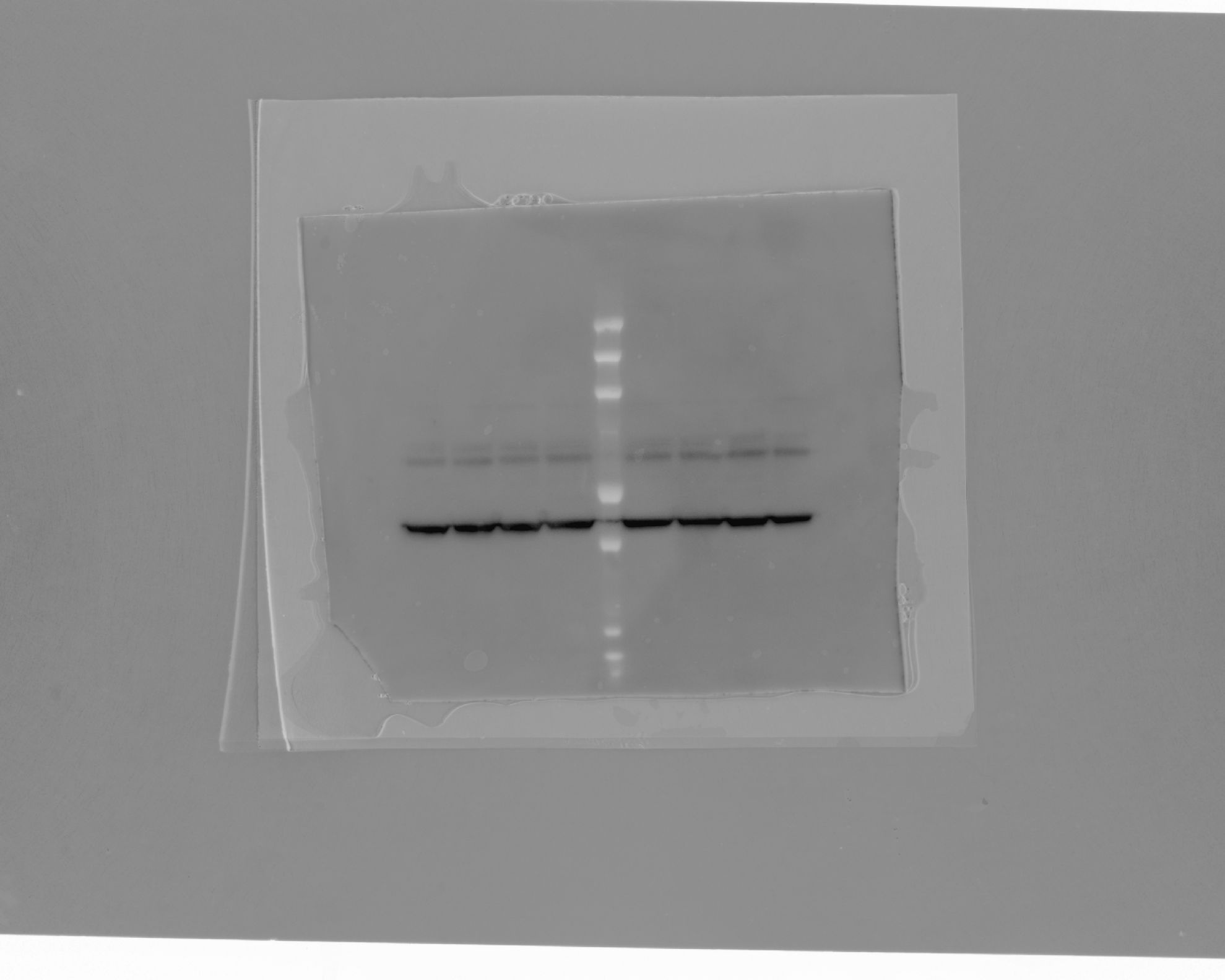

Supplement: Multimedia component 1 [file mmc1.zip › WB bands & raw densitometry/WB bands(45min)/4.(P-)4E-BP1/B-actin(4EBP1)(1)(Composite).tif]

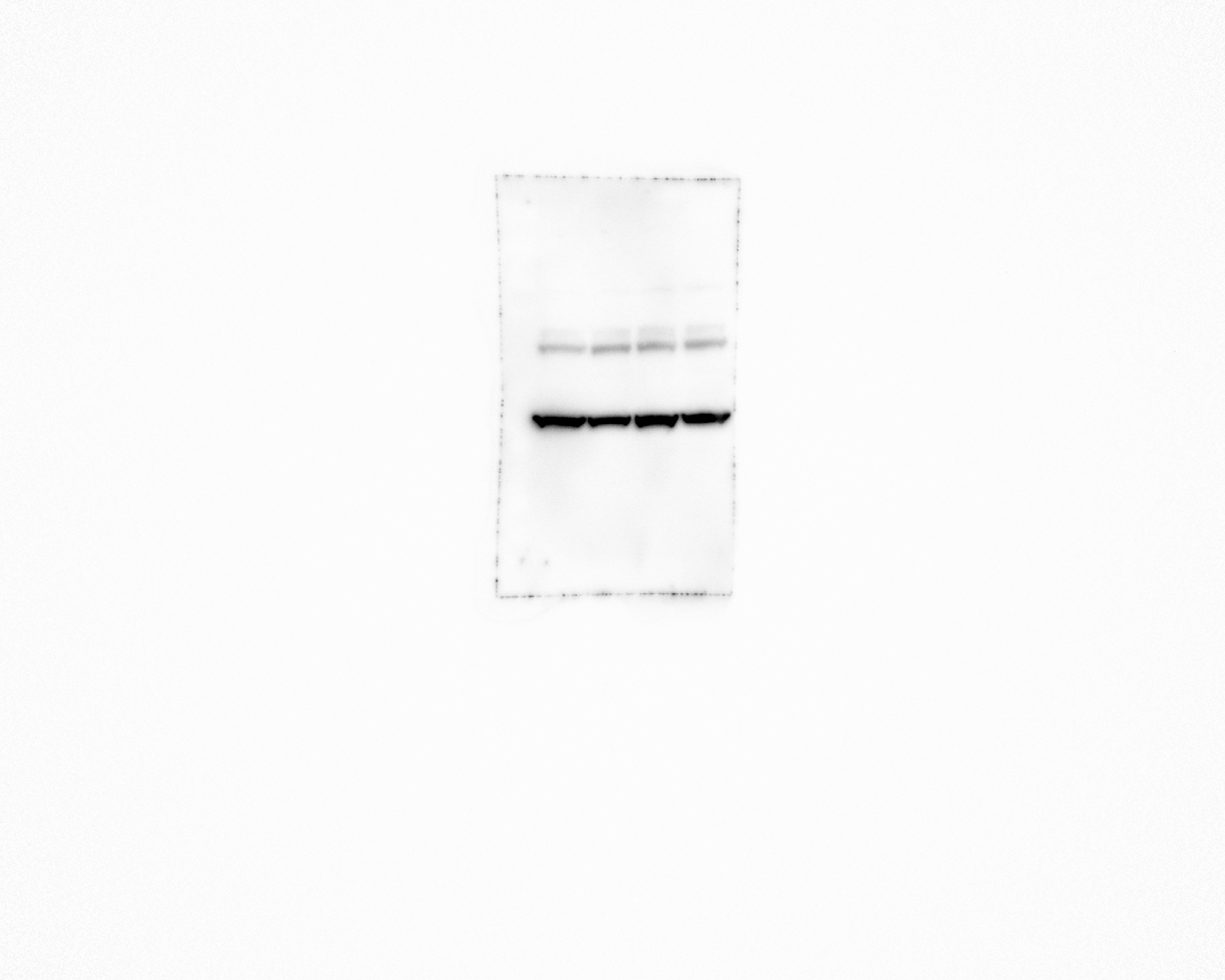

Supplement: Multimedia component 1 [file mmc1.zip › WB bands & raw densitometry/WB bands(45min)/4.(P-)4E-BP1/B-actin(4EBP1)(2)(Chemiluminescence).tif]

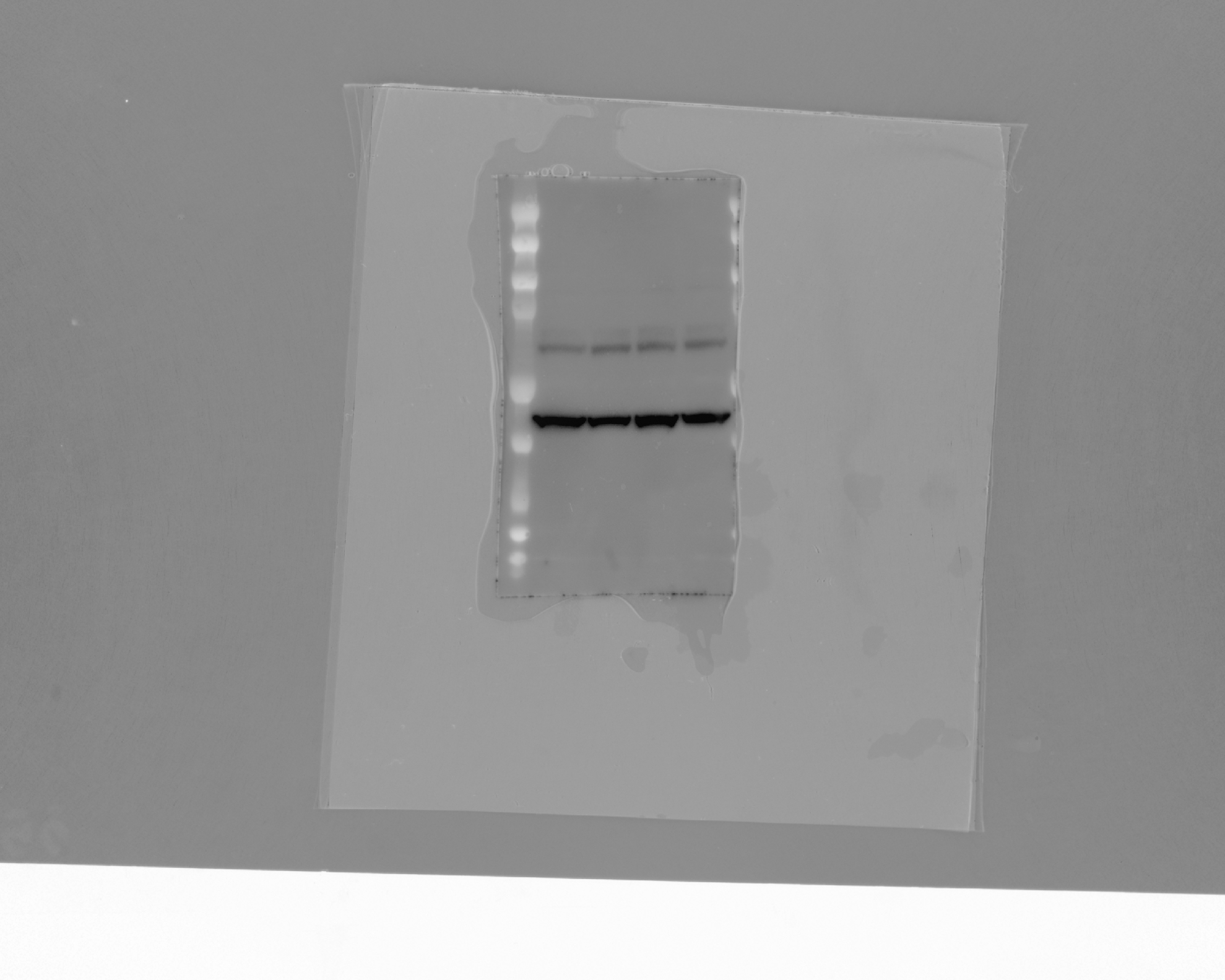

Supplement: Multimedia component 1 [file mmc1.zip › WB bands & raw densitometry/WB bands(45min)/4.(P-)4E-BP1/B-actin(4EBP1)(2)(Composite).jpg]

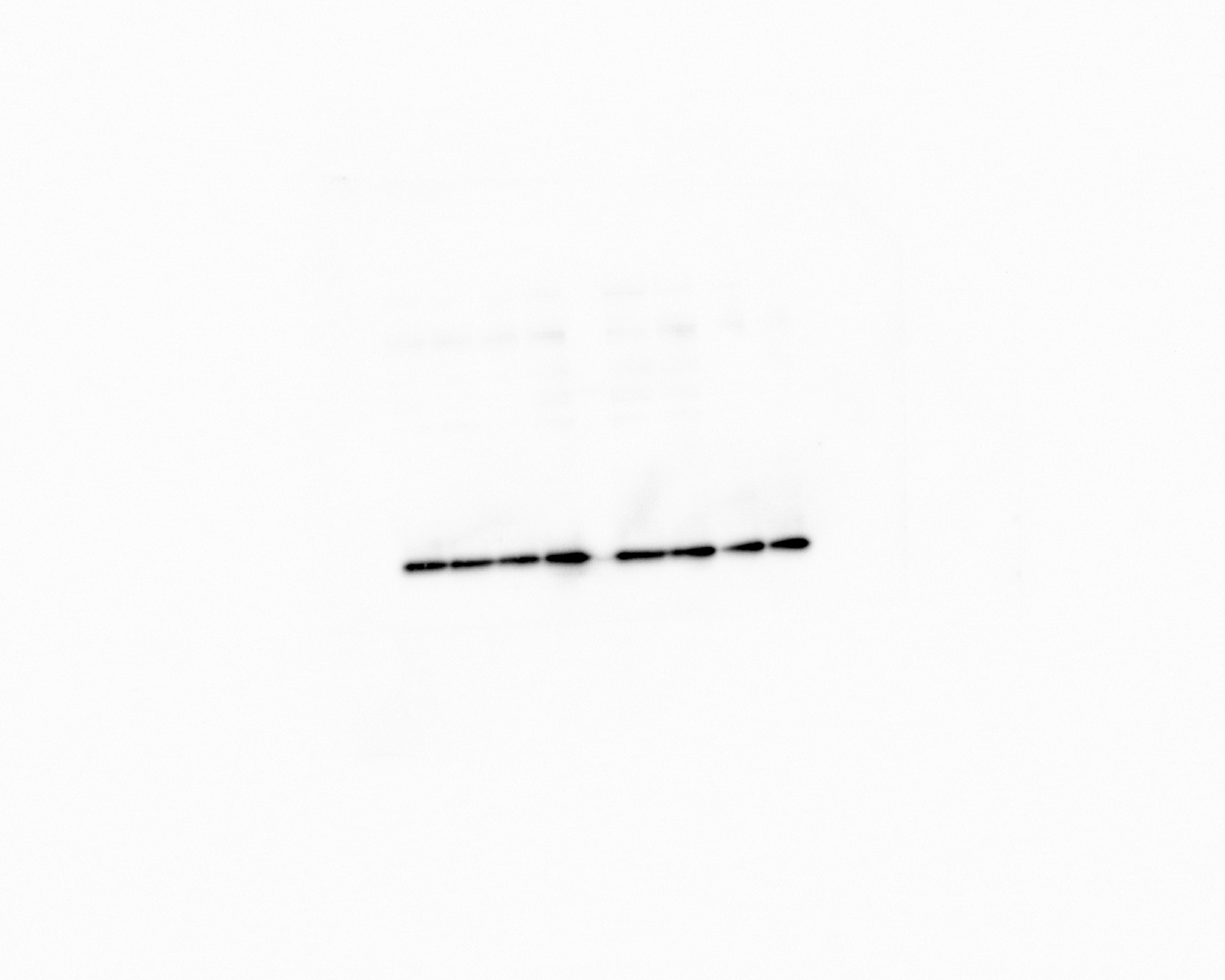

Supplement: Multimedia component 1 [file mmc1.zip › WB bands & raw densitometry/WB bands(45min)/4.(P-)4E-BP1/User 2025-09-17 45min p-4EBP1(1)(Chemiluminescence).tif]

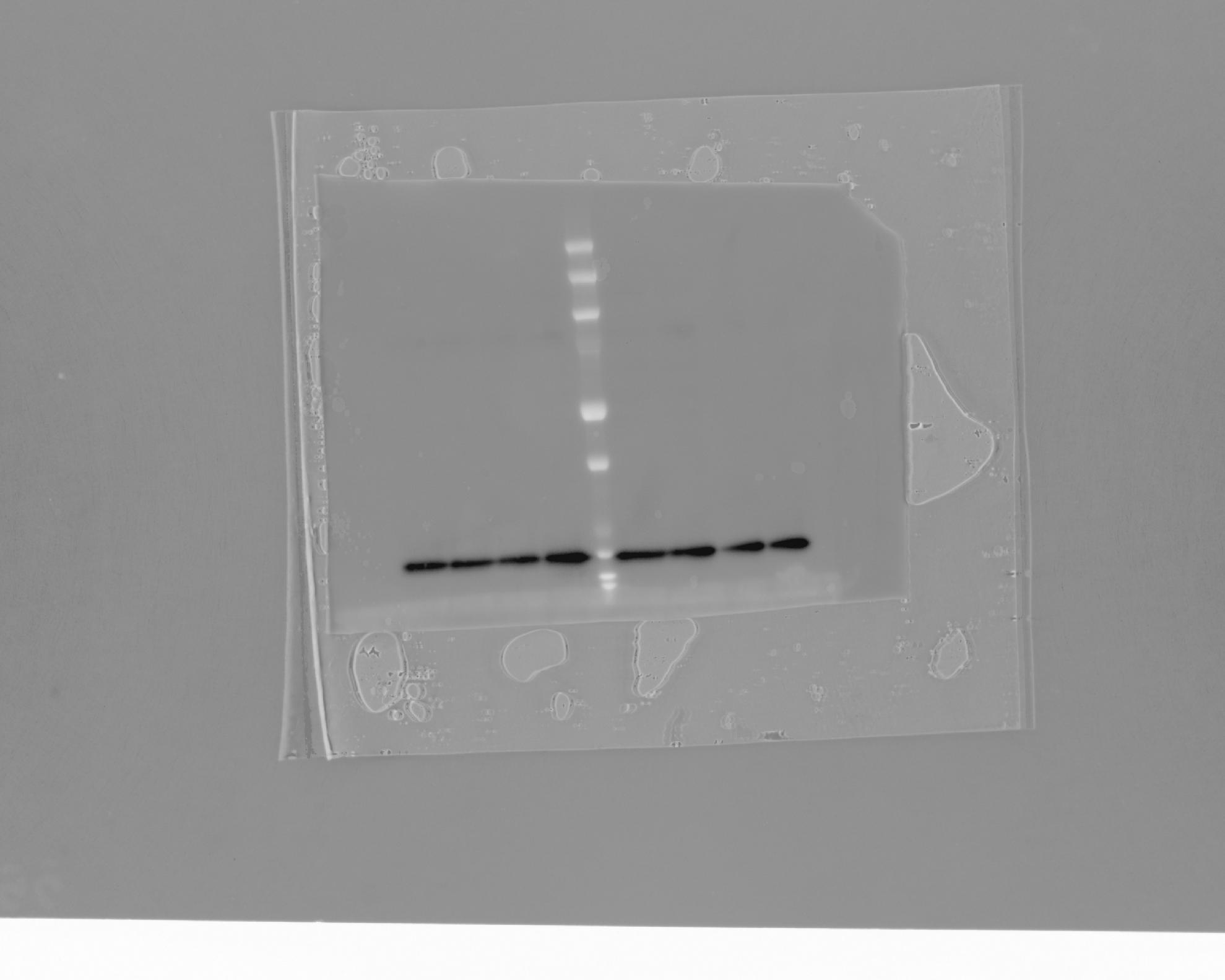

Supplement: Multimedia component 1 [file mmc1.zip › WB bands & raw densitometry/WB bands(45min)/4.(P-)4E-BP1/User 2025-09-17 45min p-4EBP1(1)(Composite).tif]

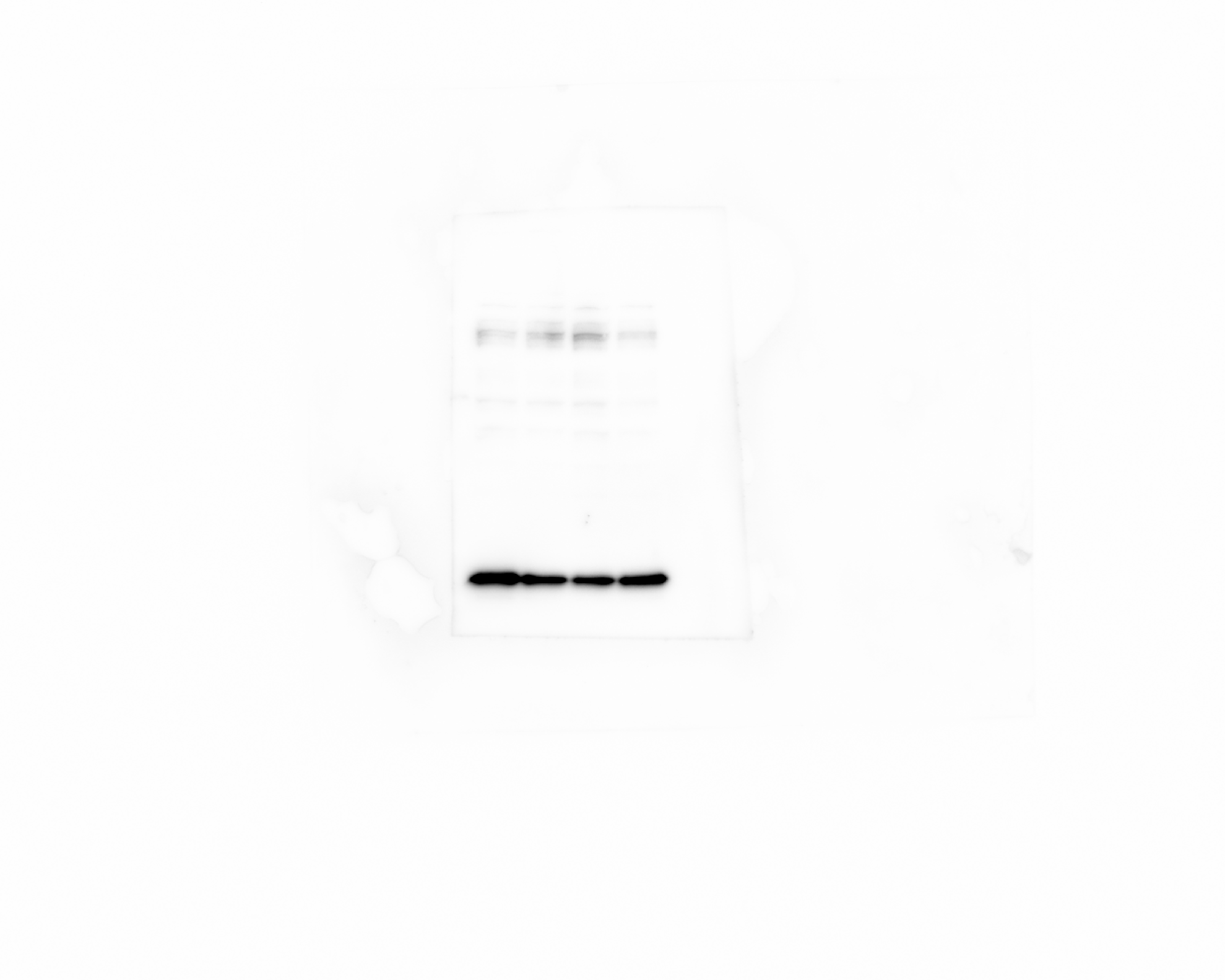

Supplement: Multimedia component 1 [file mmc1.zip › WB bands & raw densitometry/WB bands(45min)/4.(P-)4E-BP1/User 2025-09-17 45min p-4EBP1(2)(Chemiluminescence).tif]

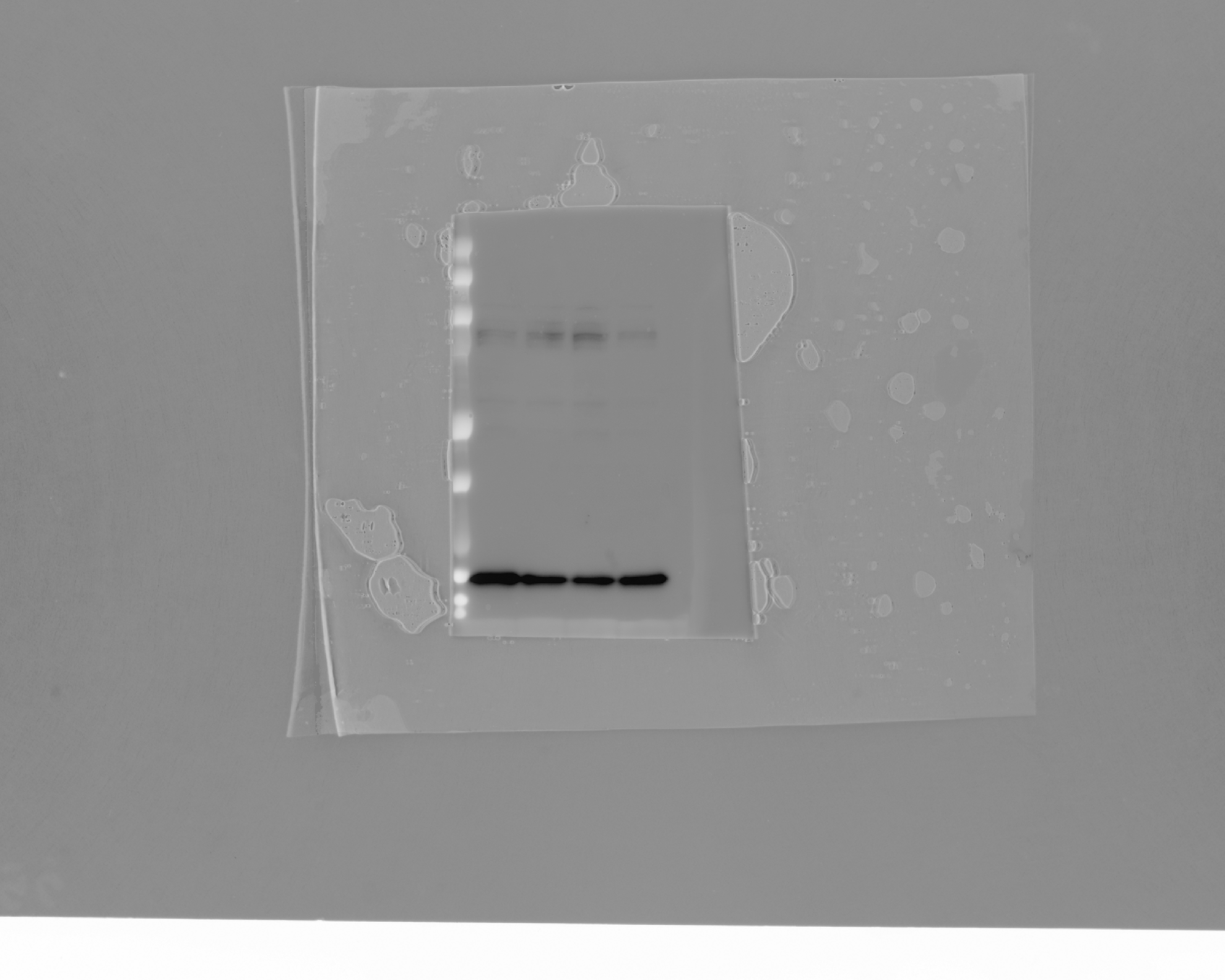

Supplement: Multimedia component 1 [file mmc1.zip › WB bands & raw densitometry/WB bands(45min)/4.(P-)4E-BP1/User 2025-09-17 45min p-4EBP1(2)(Composite).tif]

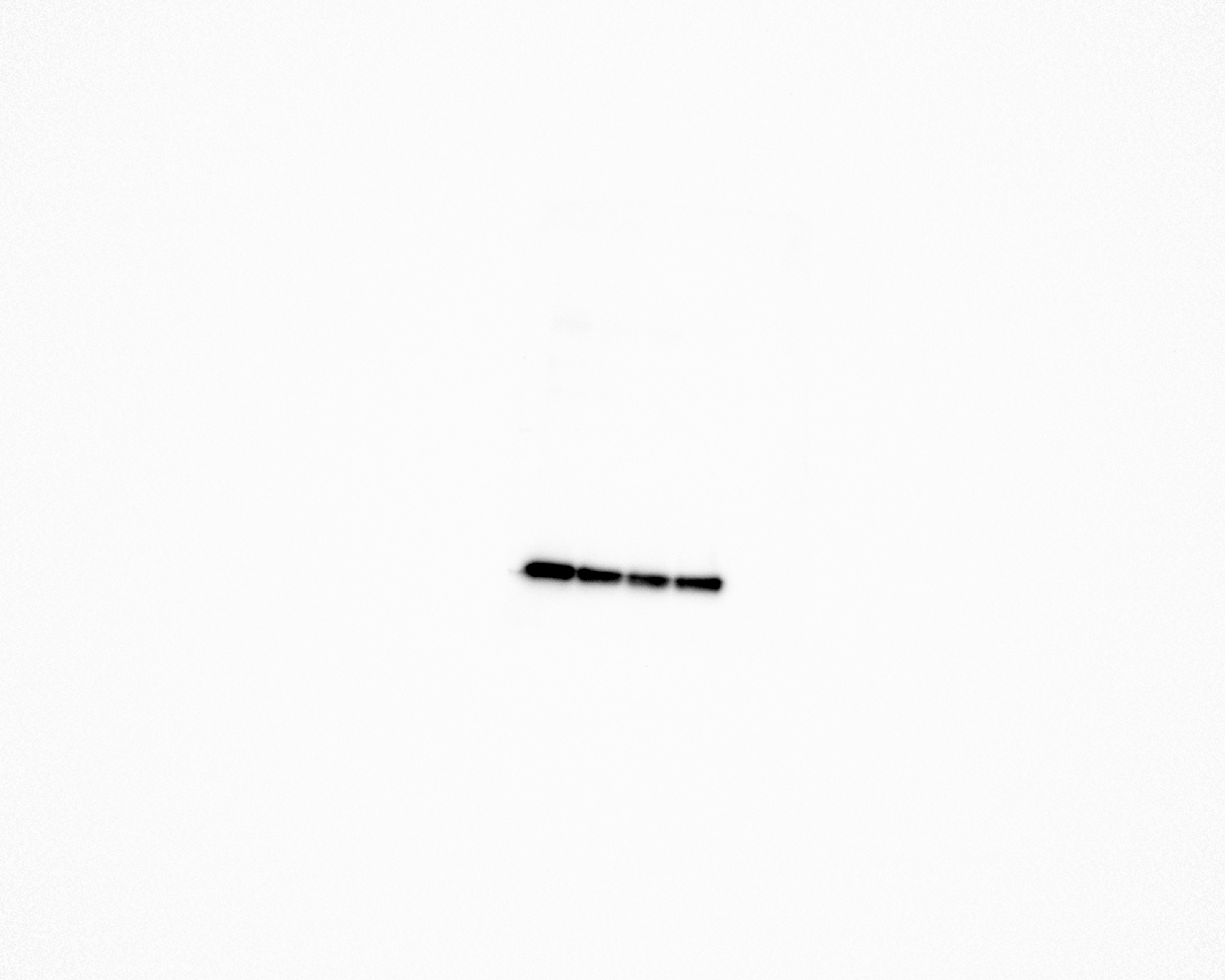

Supplement: Multimedia component 1 [file mmc1.zip › WB bands & raw densitometry/WB bands(45min)/4.(P-)4E-BP1/User 2025-09-18 4E-BP1(2)(Chemiluminescence).tif]

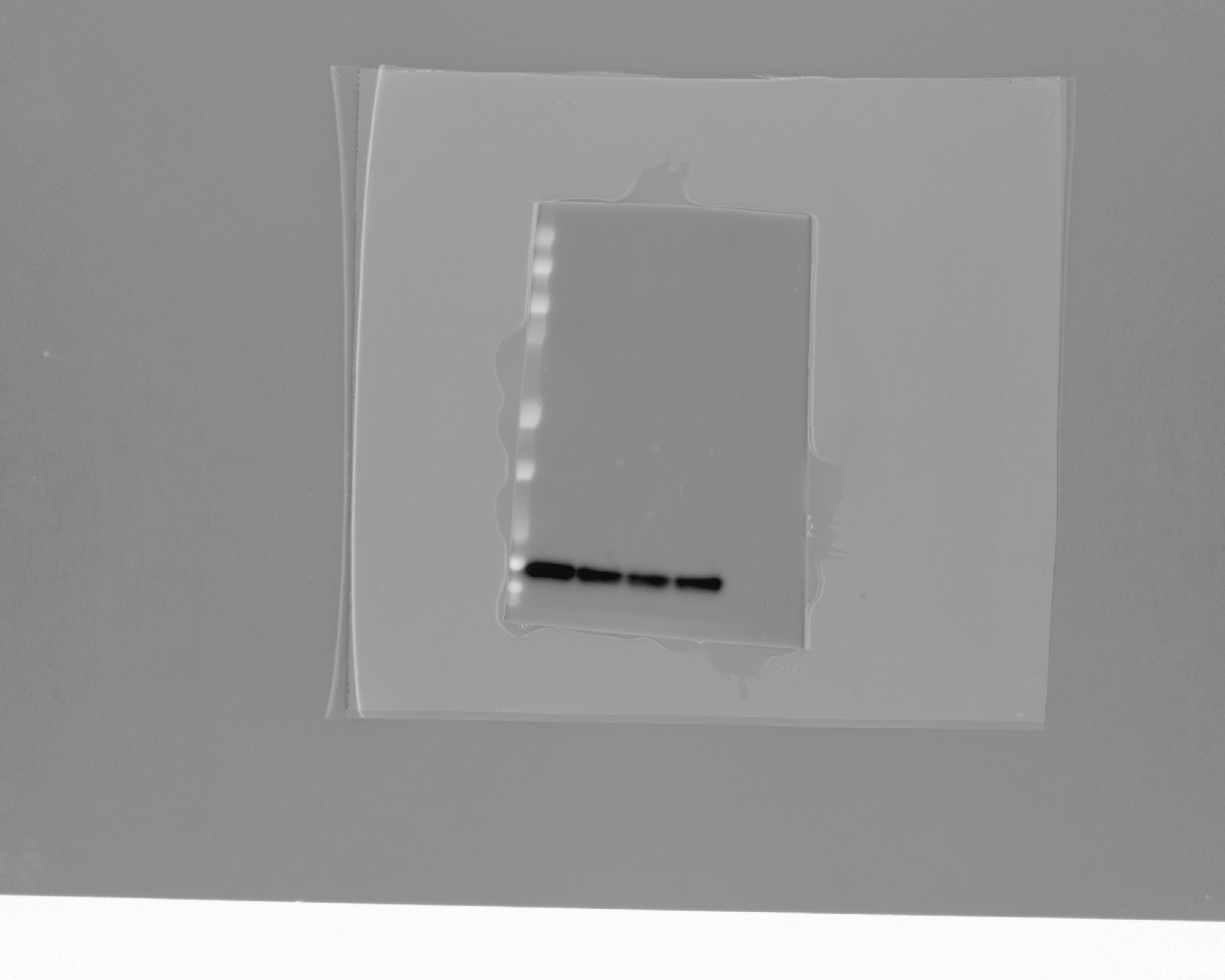

Supplement: Multimedia component 1 [file mmc1.zip › WB bands & raw densitometry/WB bands(45min)/4.(P-)4E-BP1/User 2025-09-18 4E-BP1(2)(Composite).tif]

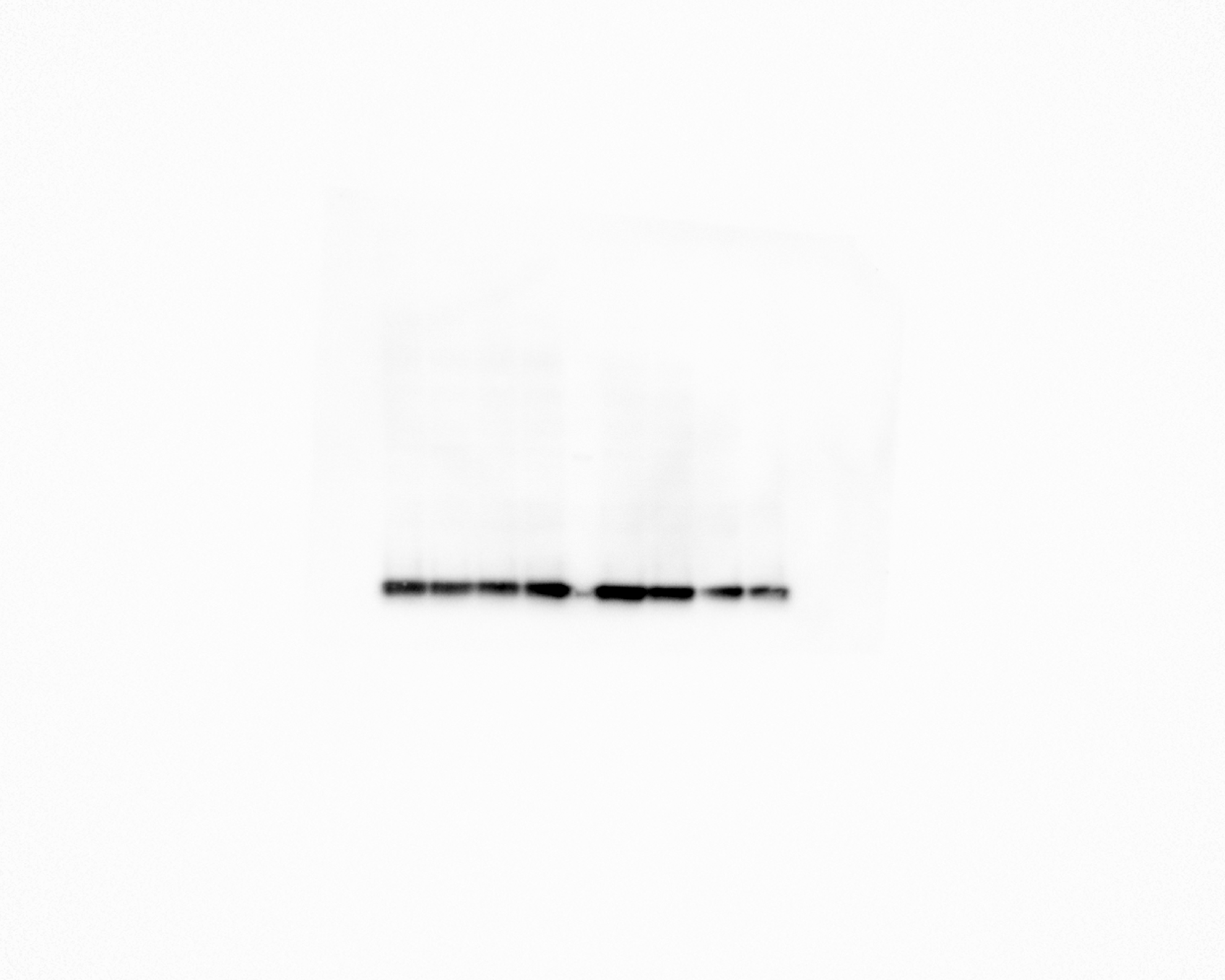

Supplement: Multimedia component 1 [file mmc1.zip › WB bands & raw densitometry/WB bands(45min)/4.(P-)4E-BP1/User 2025-09-18 4E-BP1(Chemiluminescence).tif]

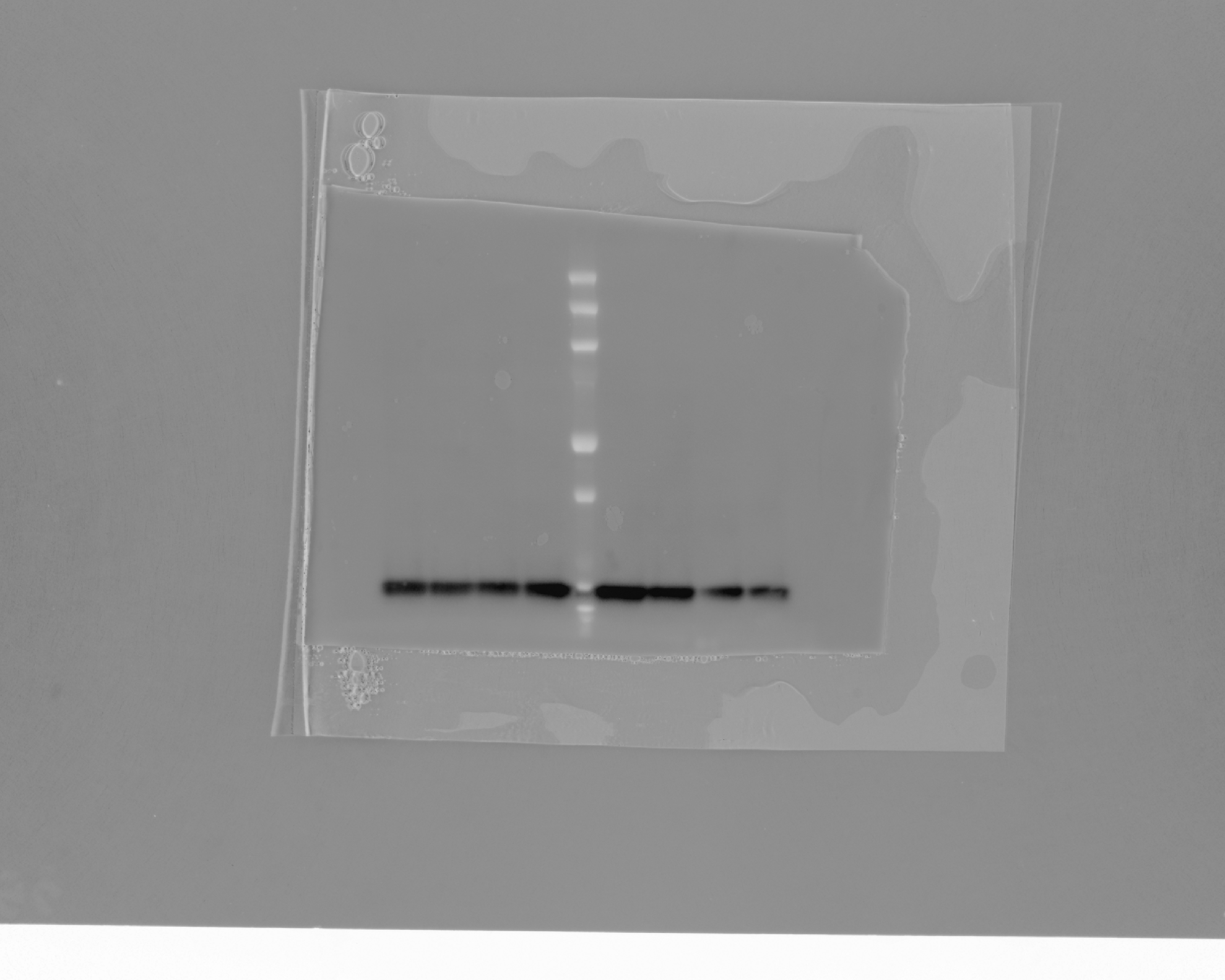

Supplement: Multimedia component 1 [file mmc1.zip › WB bands & raw densitometry/WB bands(45min)/4.(P-)4E-BP1/User 2025-09-18 4E-BP1(Composite).tif]

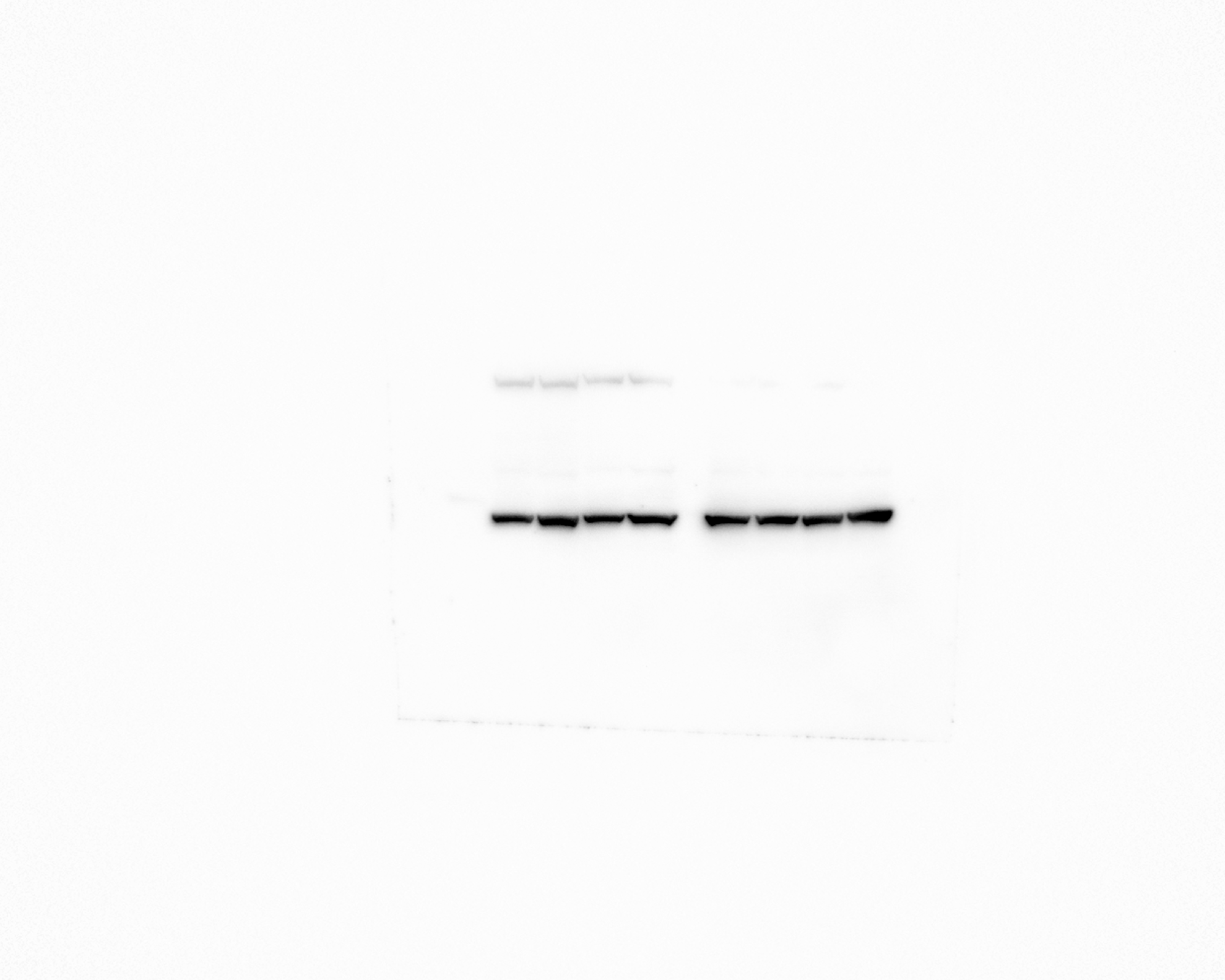

Supplement: Multimedia component 1 [file mmc1.zip › WB bands & raw densitometry/WB bands(45min)/5.(P-)eEF2/B-actin(eEF2)1(Chemiluminescence).tif]

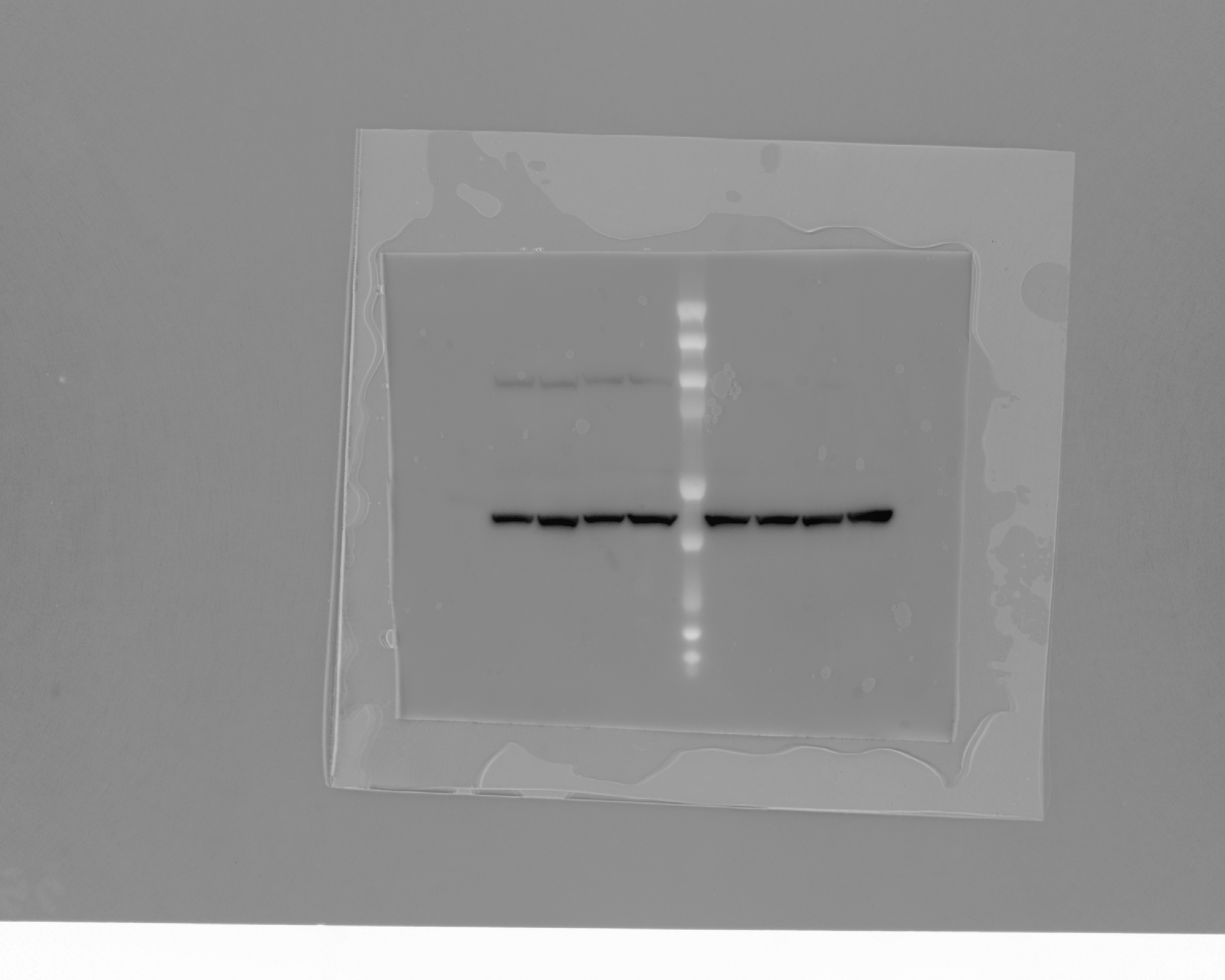

Supplement: Multimedia component 1 [file mmc1.zip › WB bands & raw densitometry/WB bands(45min)/5.(P-)eEF2/B-actin(eEF2)1(Composite).tif]

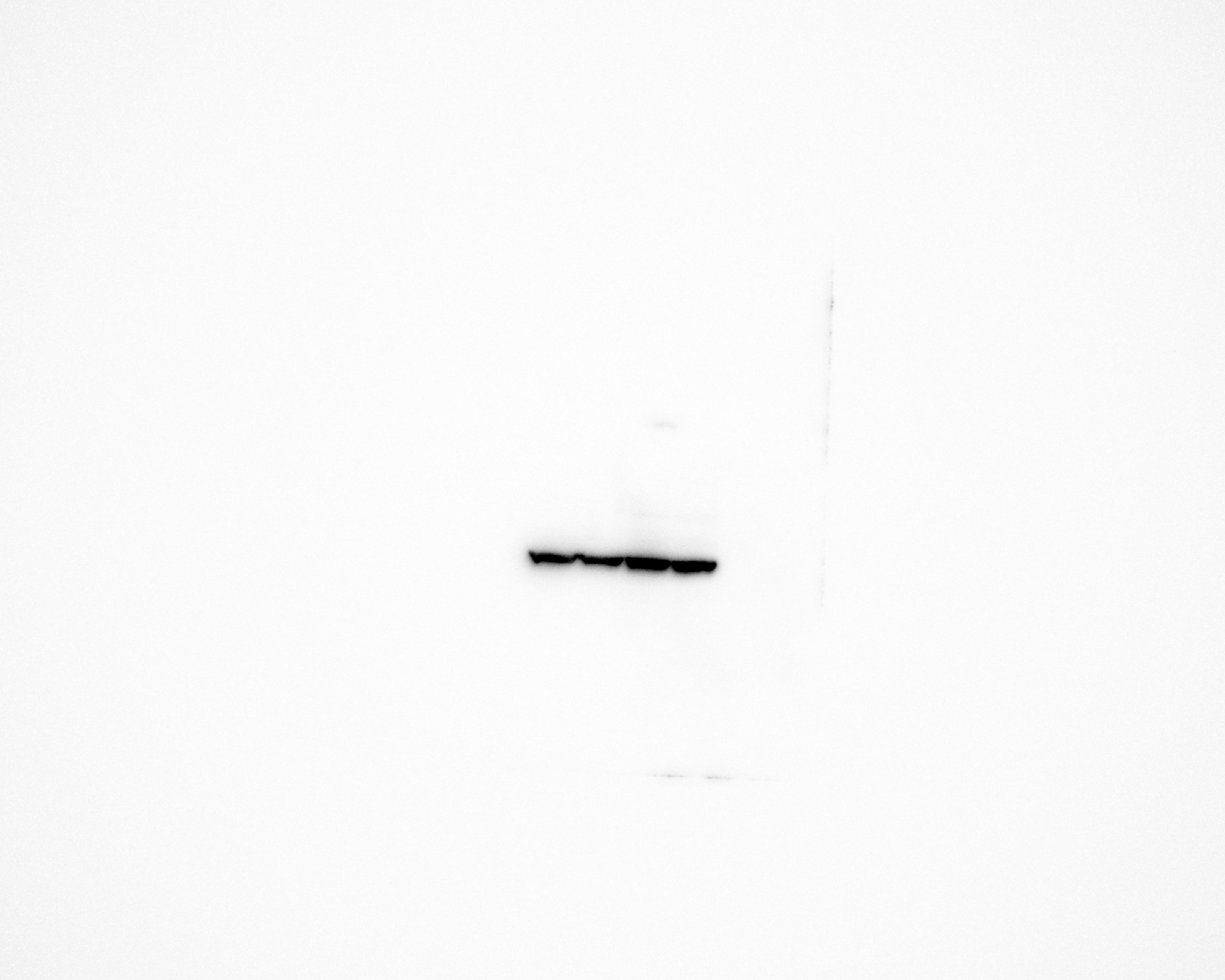

Supplement: Multimedia component 1 [file mmc1.zip › WB bands & raw densitometry/WB bands(45min)/5.(P-)eEF2/B-actin(eEF2)2(Chemiluminescence).tif]

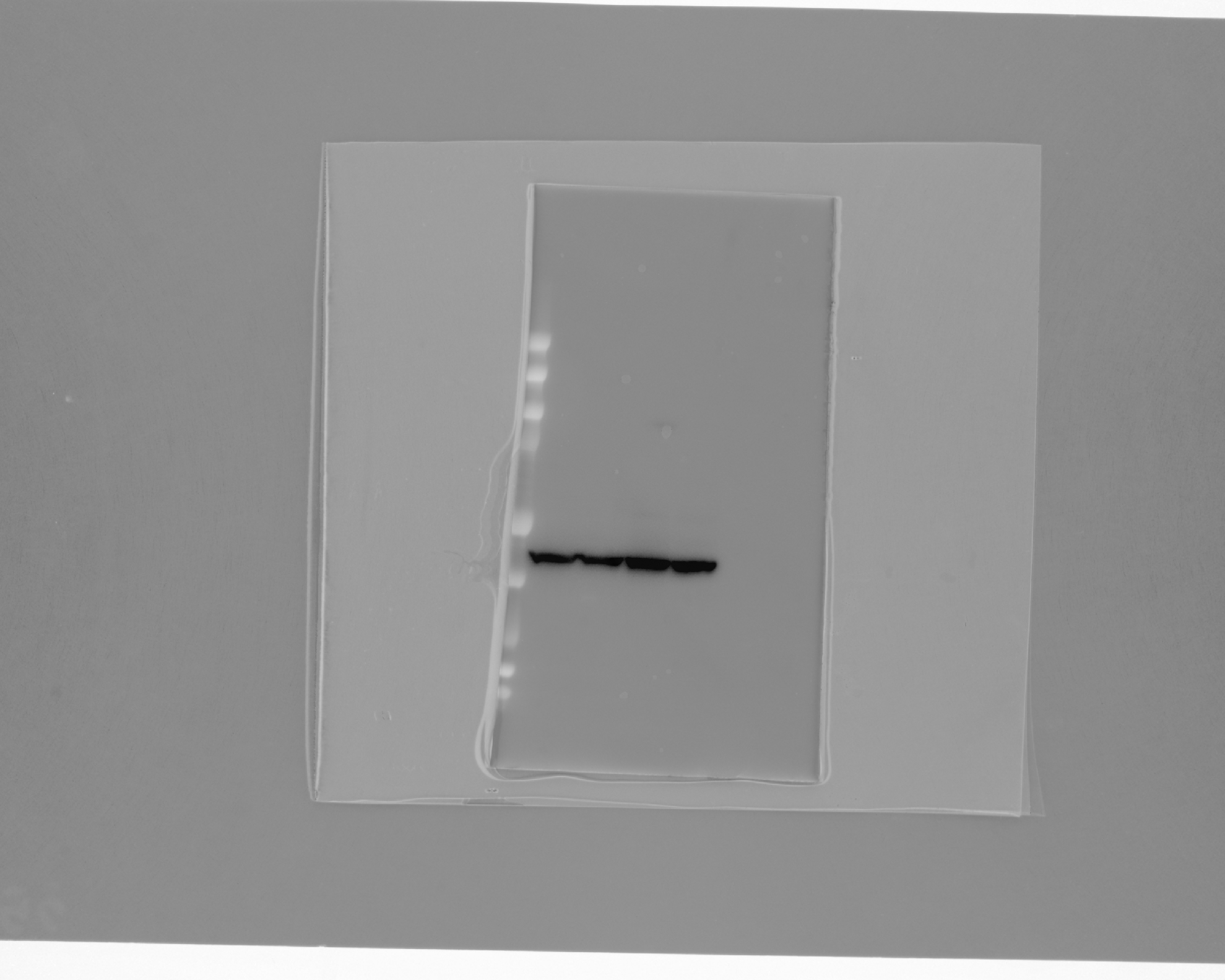

Supplement: Multimedia component 1 [file mmc1.zip › WB bands & raw densitometry/WB bands(45min)/5.(P-)eEF2/B-actin(eEF2)2(Composite).tif]

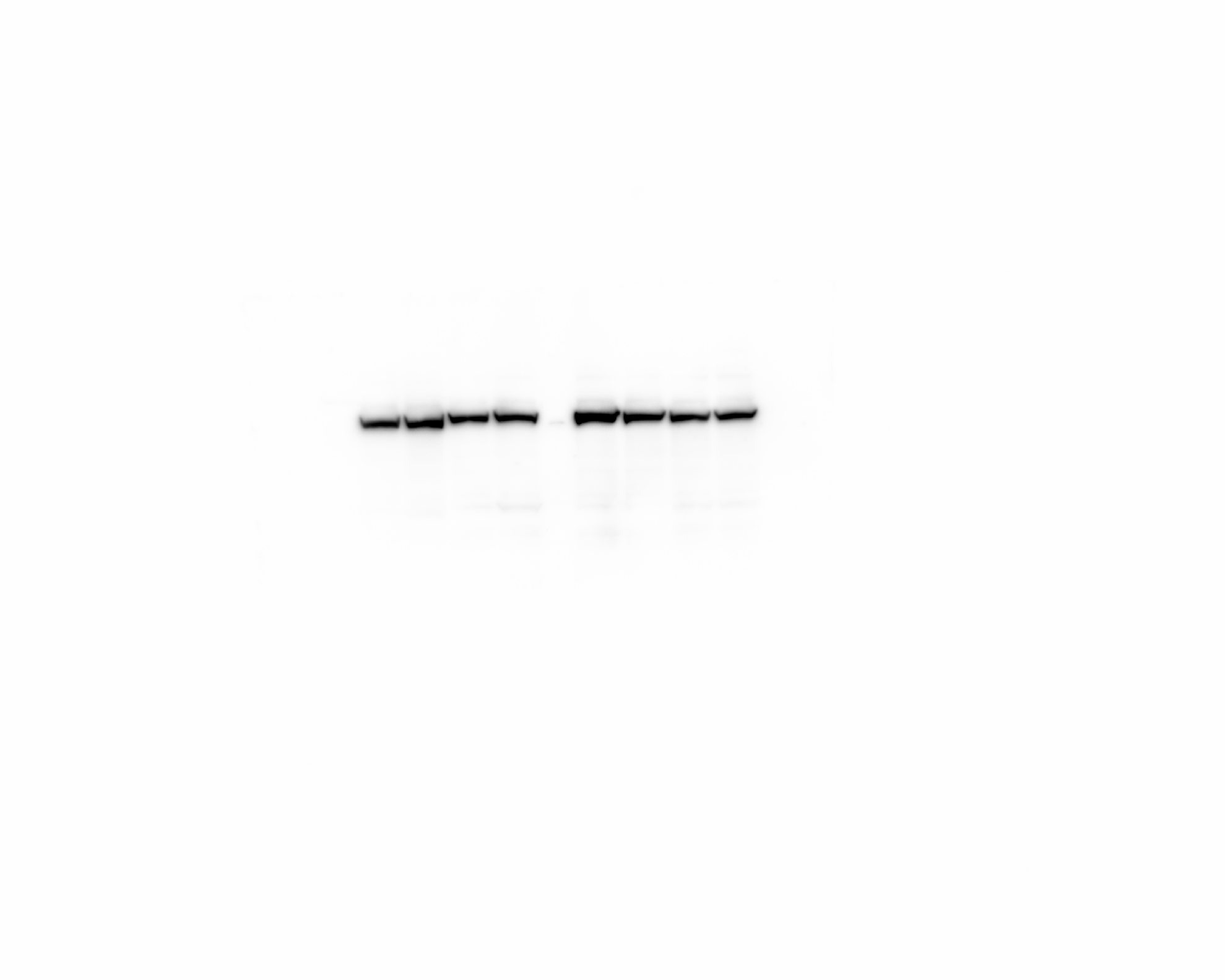

Supplement: Multimedia component 1 [file mmc1.zip › WB bands & raw densitometry/WB bands(45min)/5.(P-)eEF2/eEF2(1-3)(Chemiluminescence).tif]

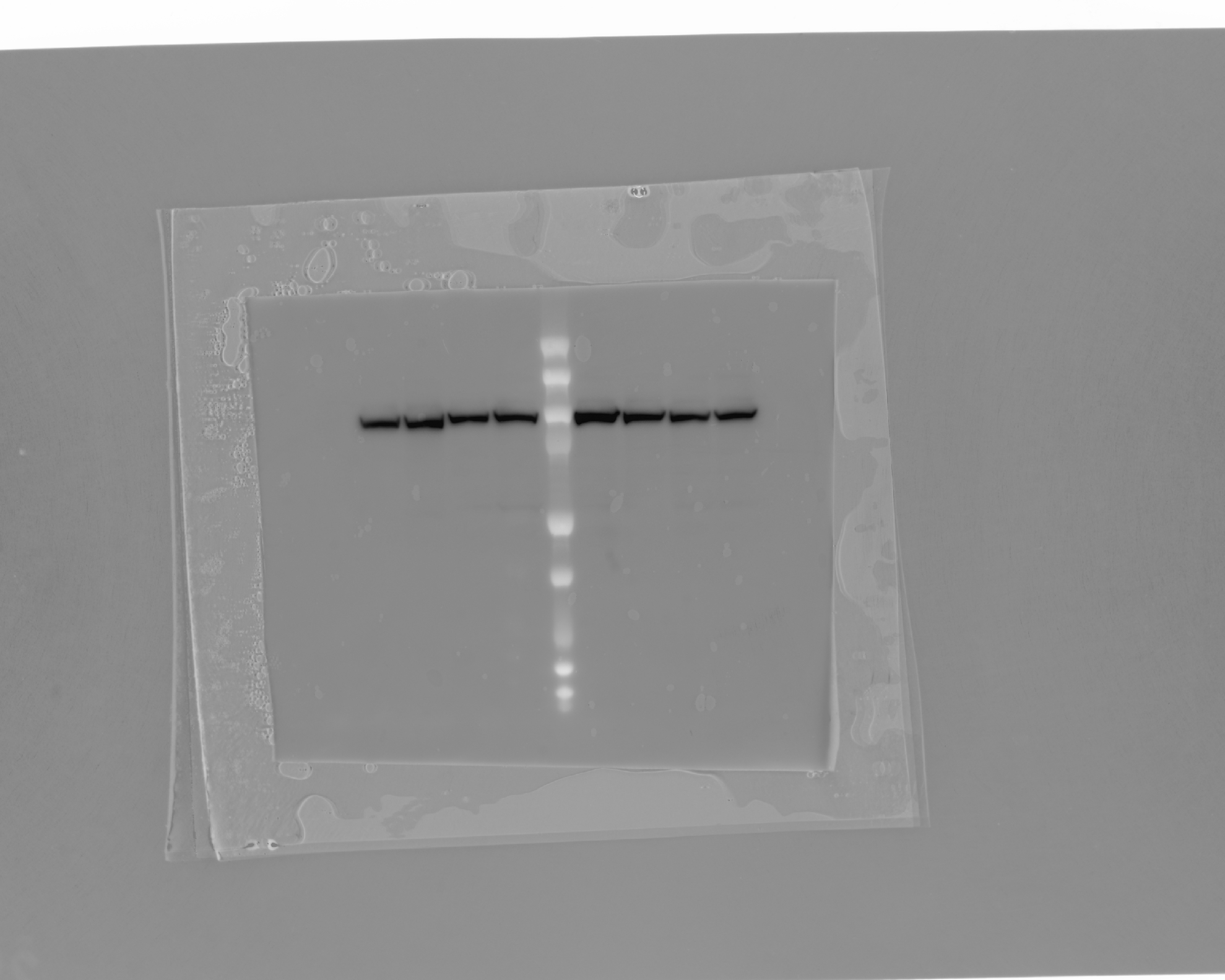

Supplement: Multimedia component 1 [file mmc1.zip › WB bands & raw densitometry/WB bands(45min)/5.(P-)eEF2/eEF2(1-3)(Composite).jpg]

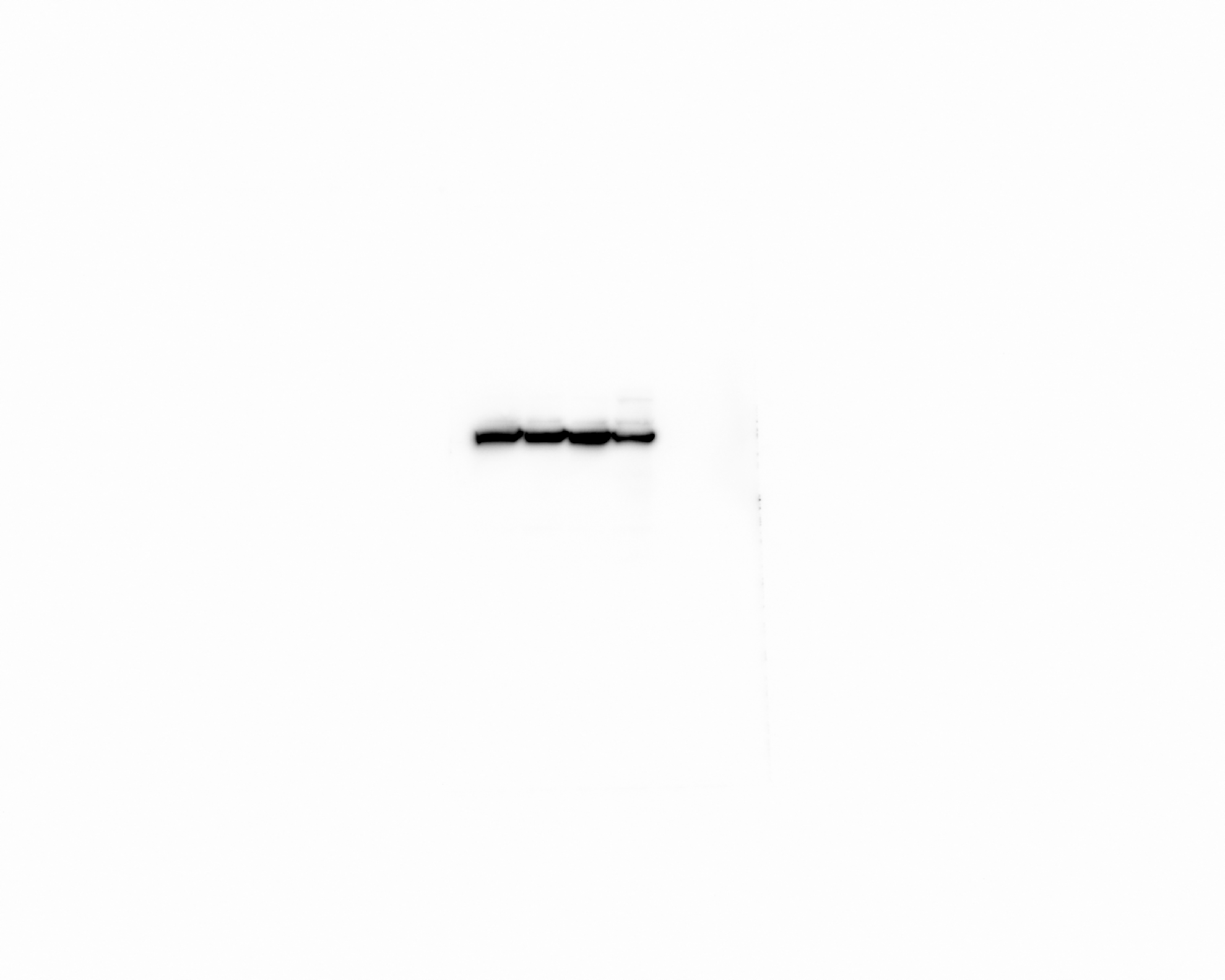

Supplement: Multimedia component 1 [file mmc1.zip › WB bands & raw densitometry/WB bands(45min)/5.(P-)eEF2/eEF2(2)-1(Chemiluminescence).tif]

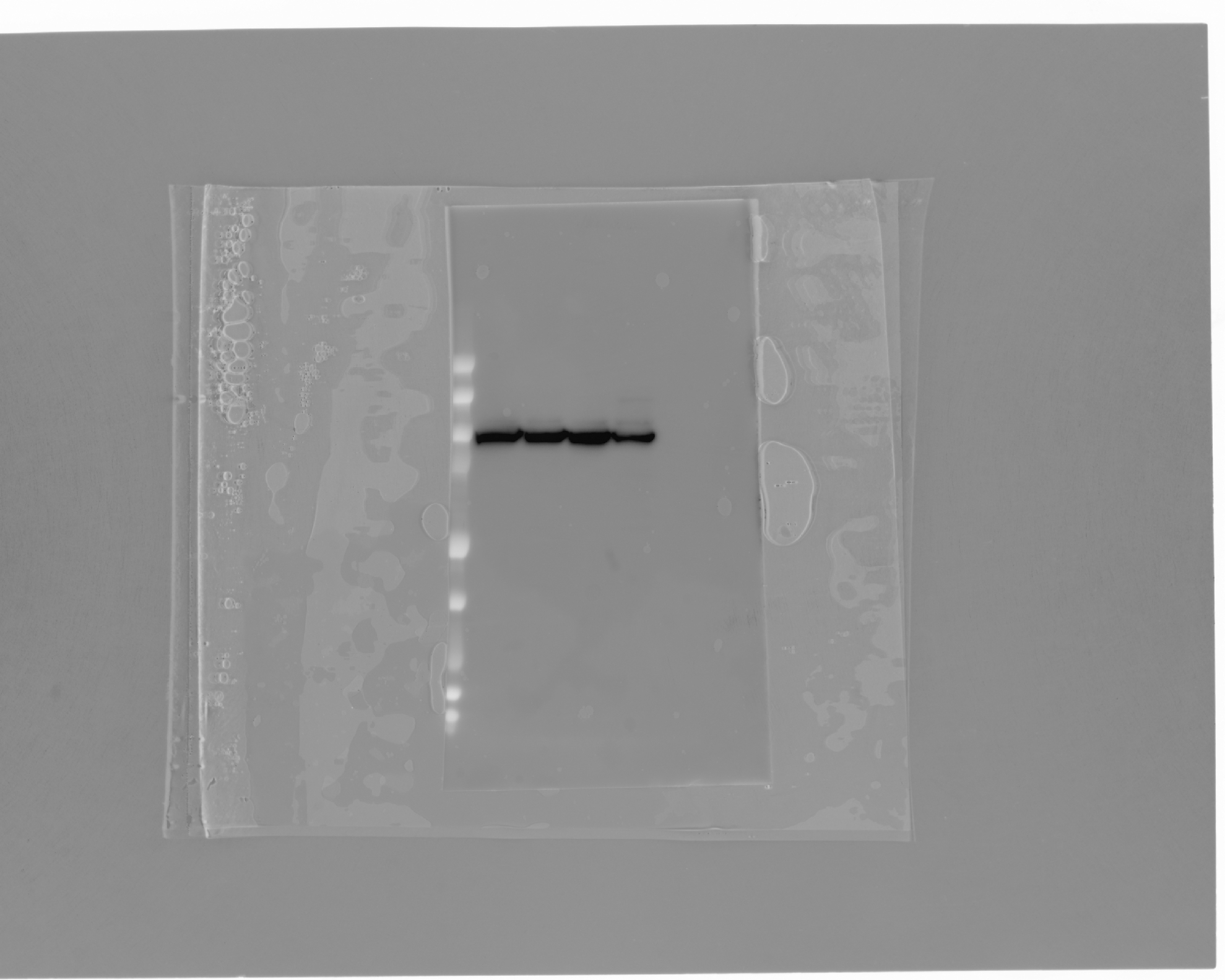

Supplement: Multimedia component 1 [file mmc1.zip › WB bands & raw densitometry/WB bands(45min)/5.(P-)eEF2/eEF2(2)-1(Composite).jpg]

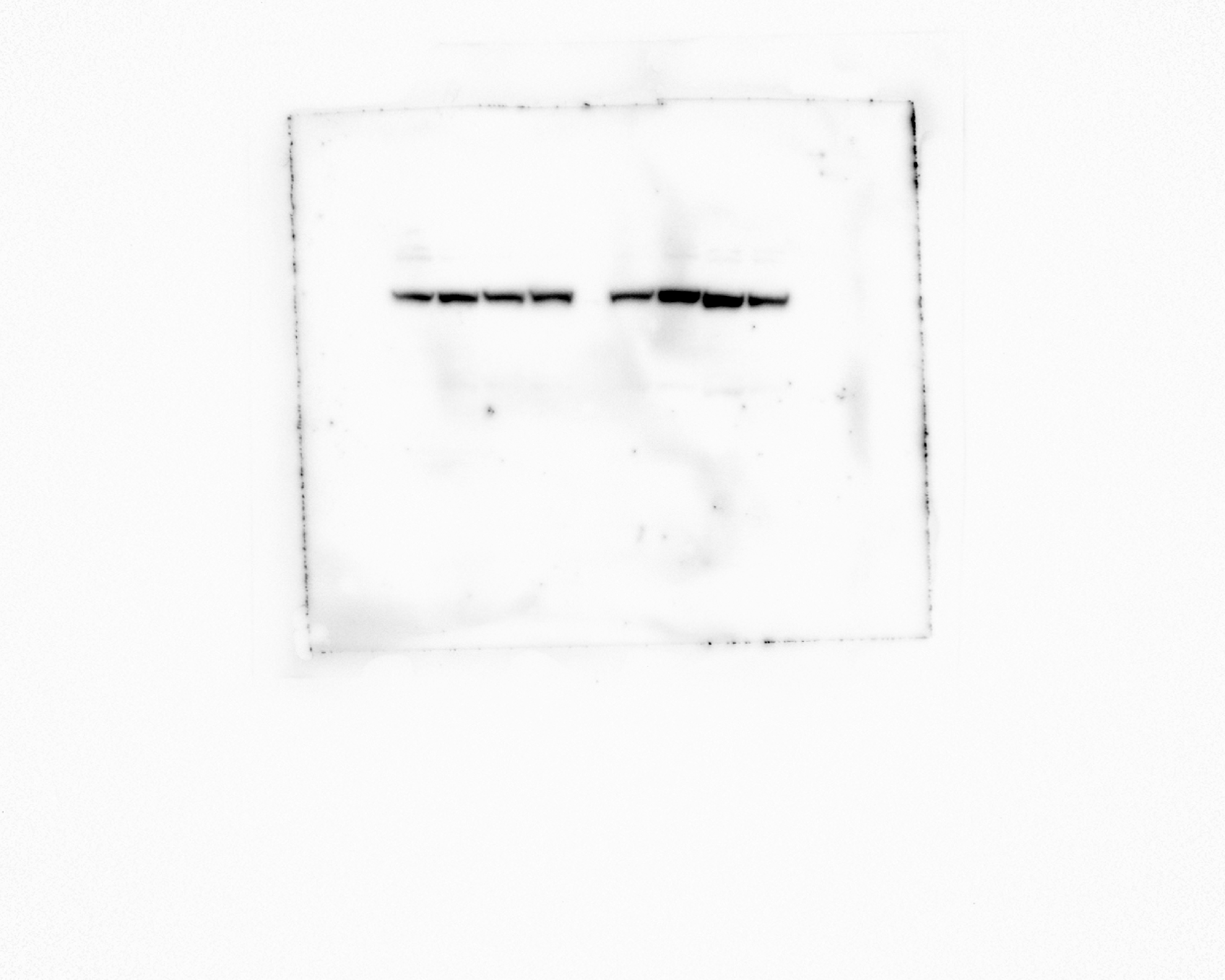

Supplement: Multimedia component 1 [file mmc1.zip › WB bands & raw densitometry/WB bands(45min)/5.(P-)eEF2/User 2025-09-18 p-eEF2(1)(Chemiluminescence).tif]

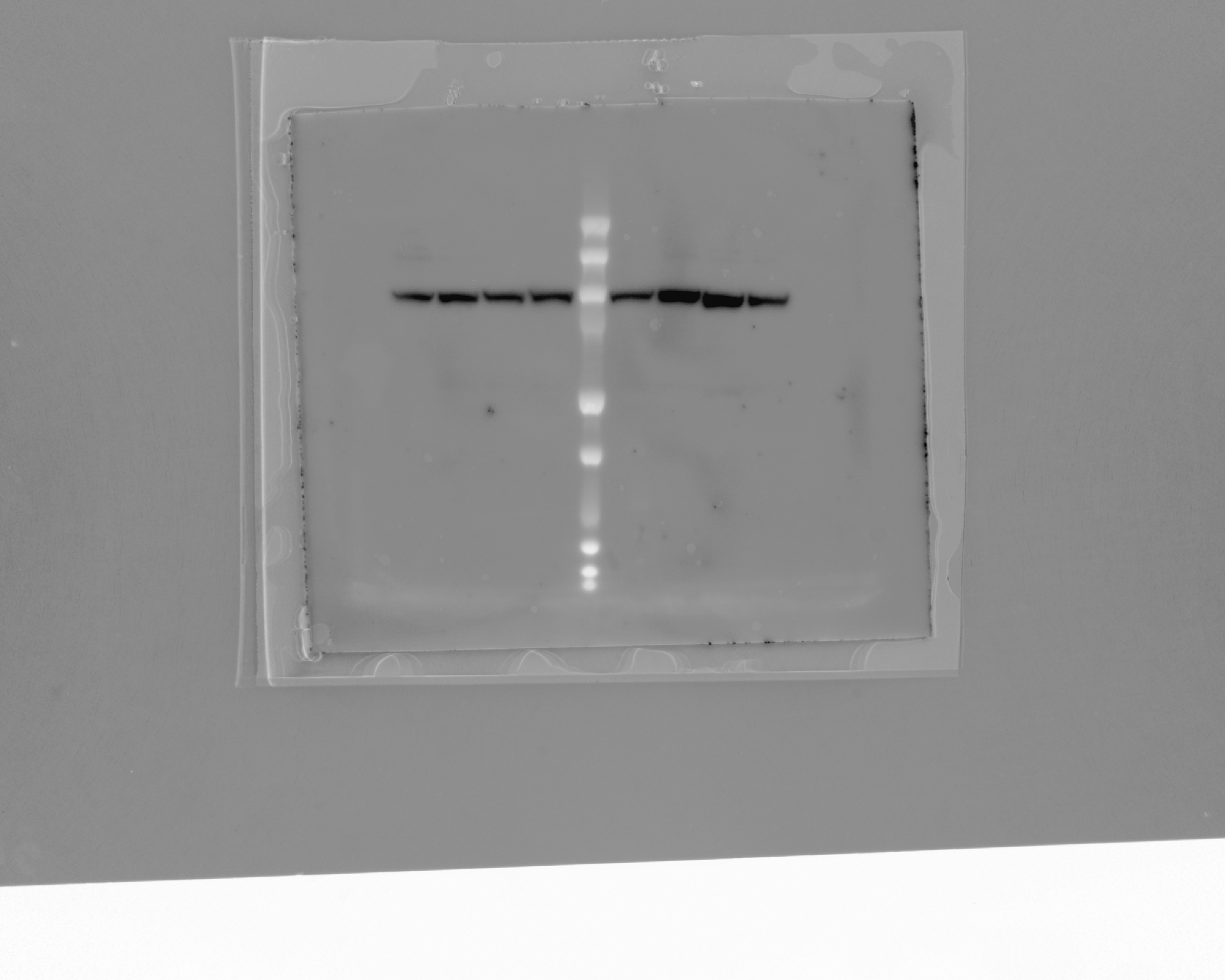

Supplement: Multimedia component 1 [file mmc1.zip › WB bands & raw densitometry/WB bands(45min)/5.(P-)eEF2/User 2025-09-18 p-eEF2(1)(Composite).tif]

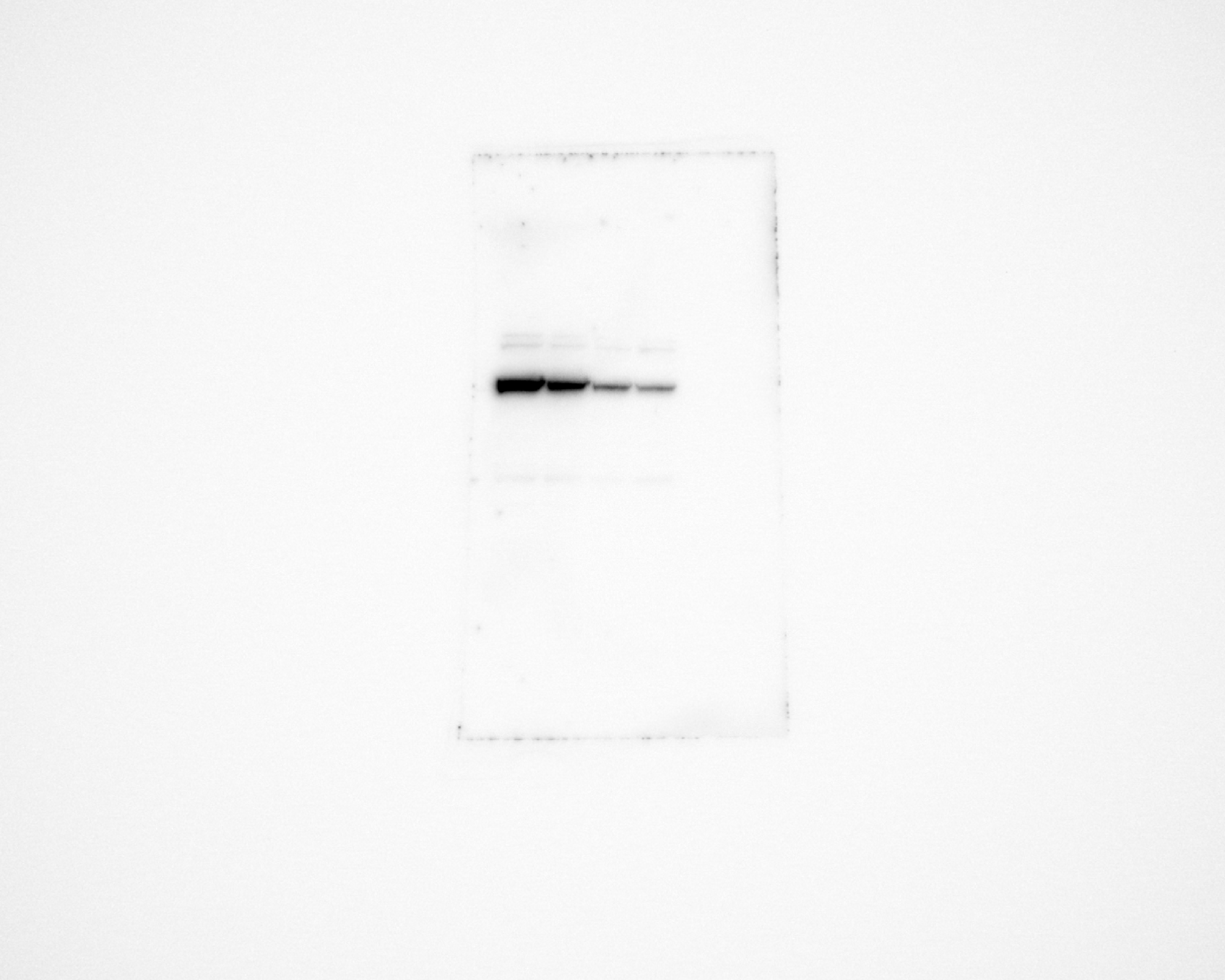

Supplement: Multimedia component 1 [file mmc1.zip › WB bands & raw densitometry/WB bands(45min)/5.(P-)eEF2/User 2025-09-18 p-eEF2(2)(Chemiluminescence).tif]

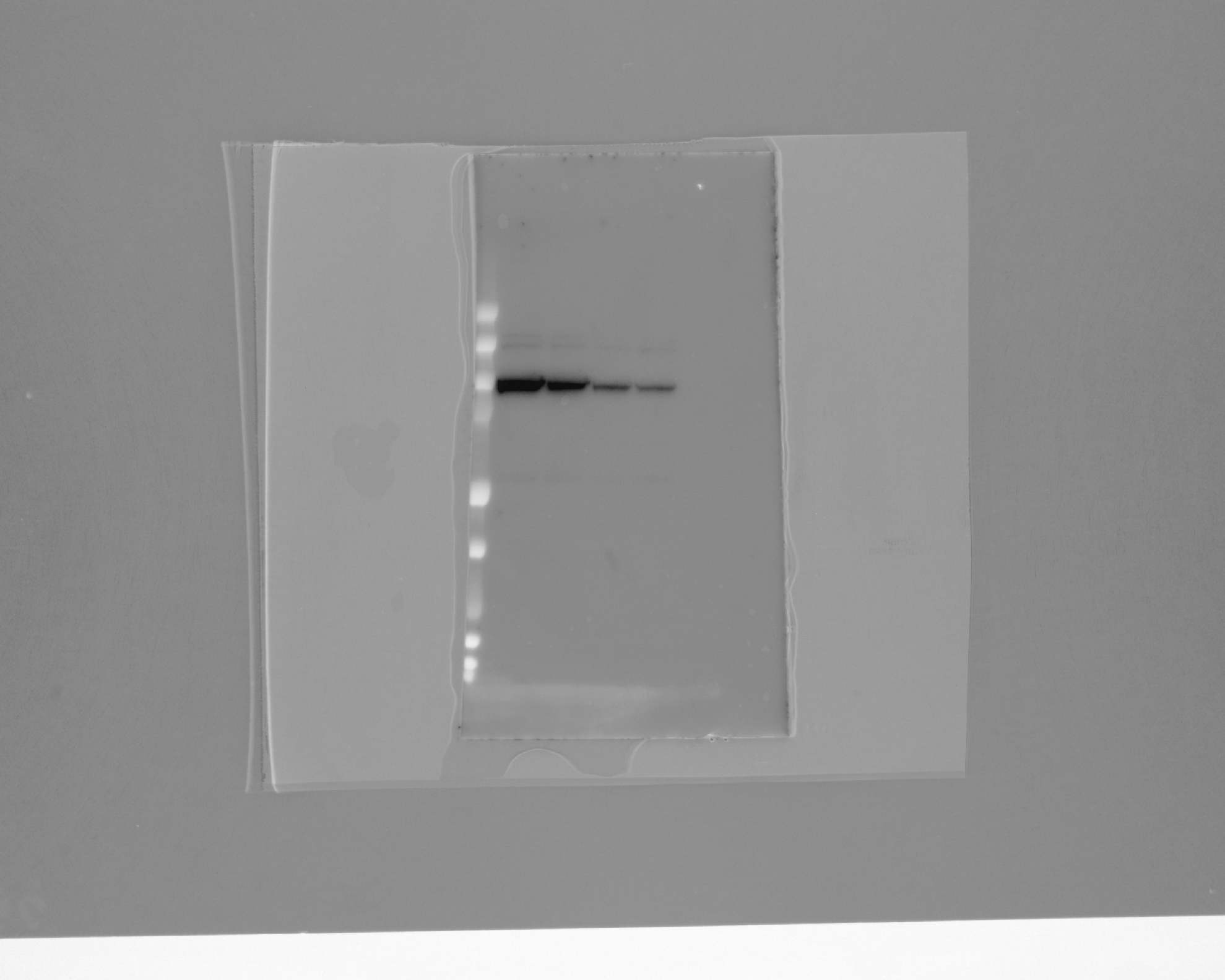

Supplement: Multimedia component 1 [file mmc1.zip › WB bands & raw densitometry/WB bands(45min)/5.(P-)eEF2/User 2025-09-18 p-eEF2(2)(Composite).tif]

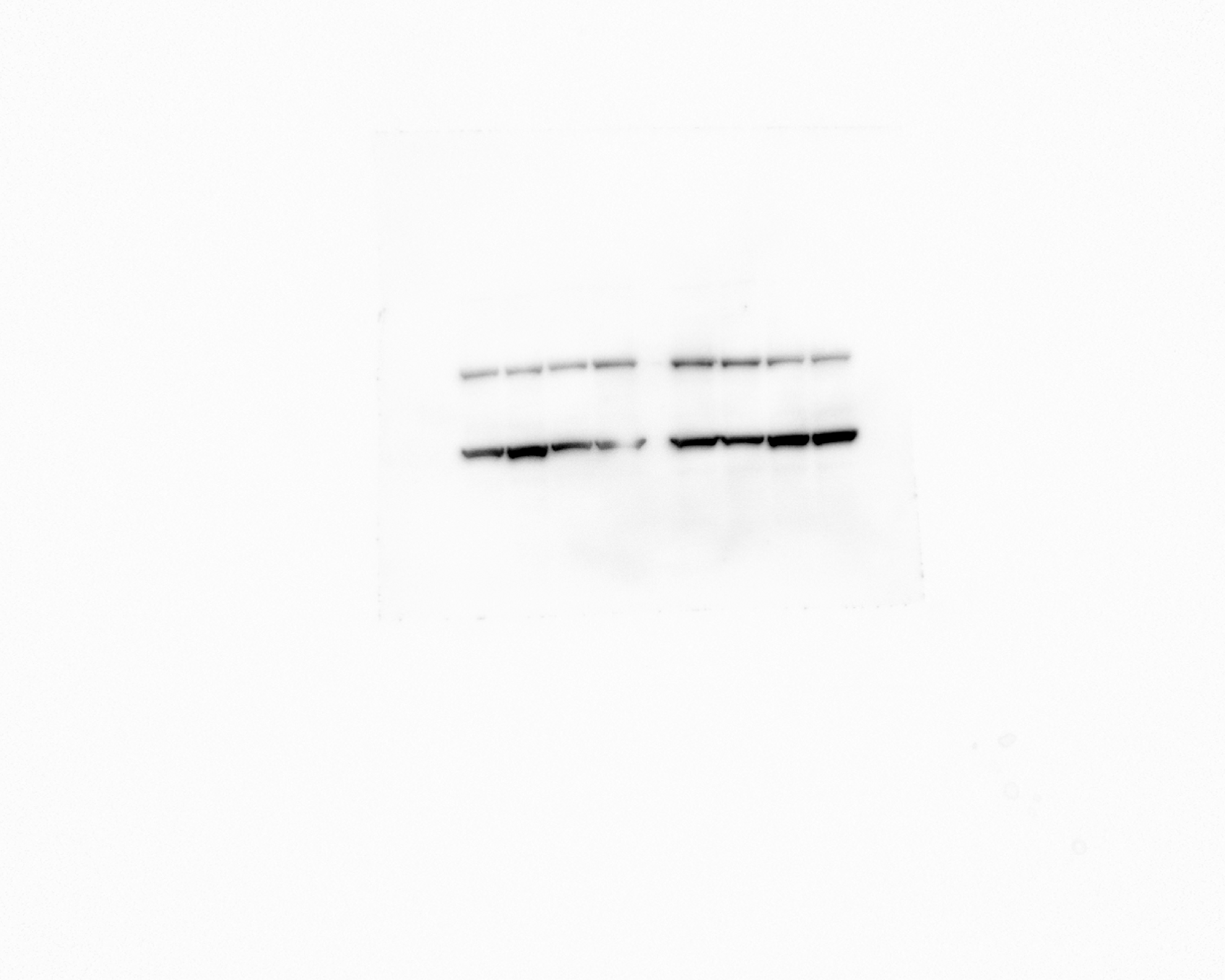

Supplement: Multimedia component 1 [file mmc1.zip › WB bands & raw densitometry/WB bands(45min)/6.(P-)NF-kB/B-actin(NF-kB)(1-1)(Chemiluminescence).tif]

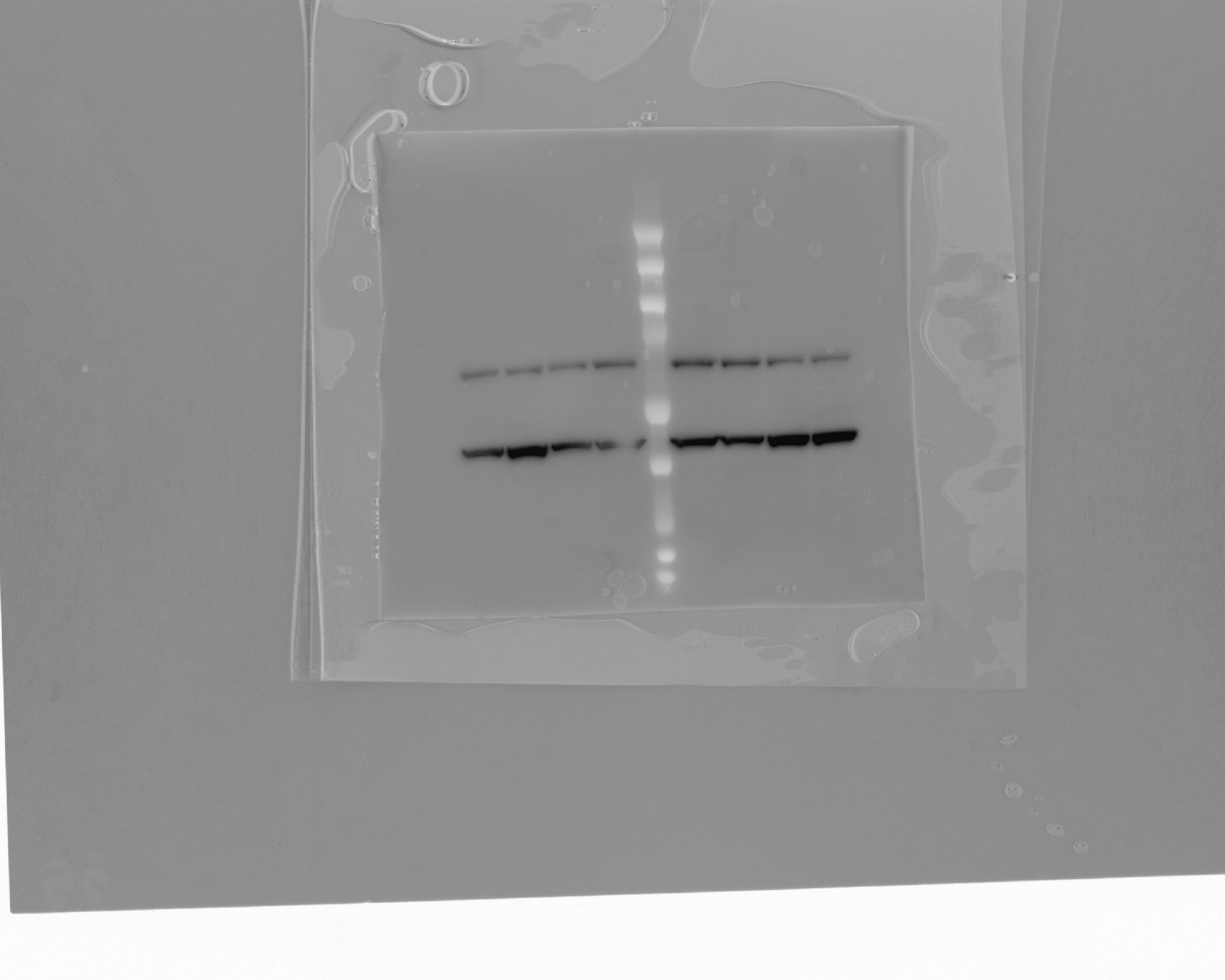

Supplement: Multimedia component 1 [file mmc1.zip › WB bands & raw densitometry/WB bands(45min)/6.(P-)NF-kB/B-actin(NF-kB)(1-1)(Composite).tif]

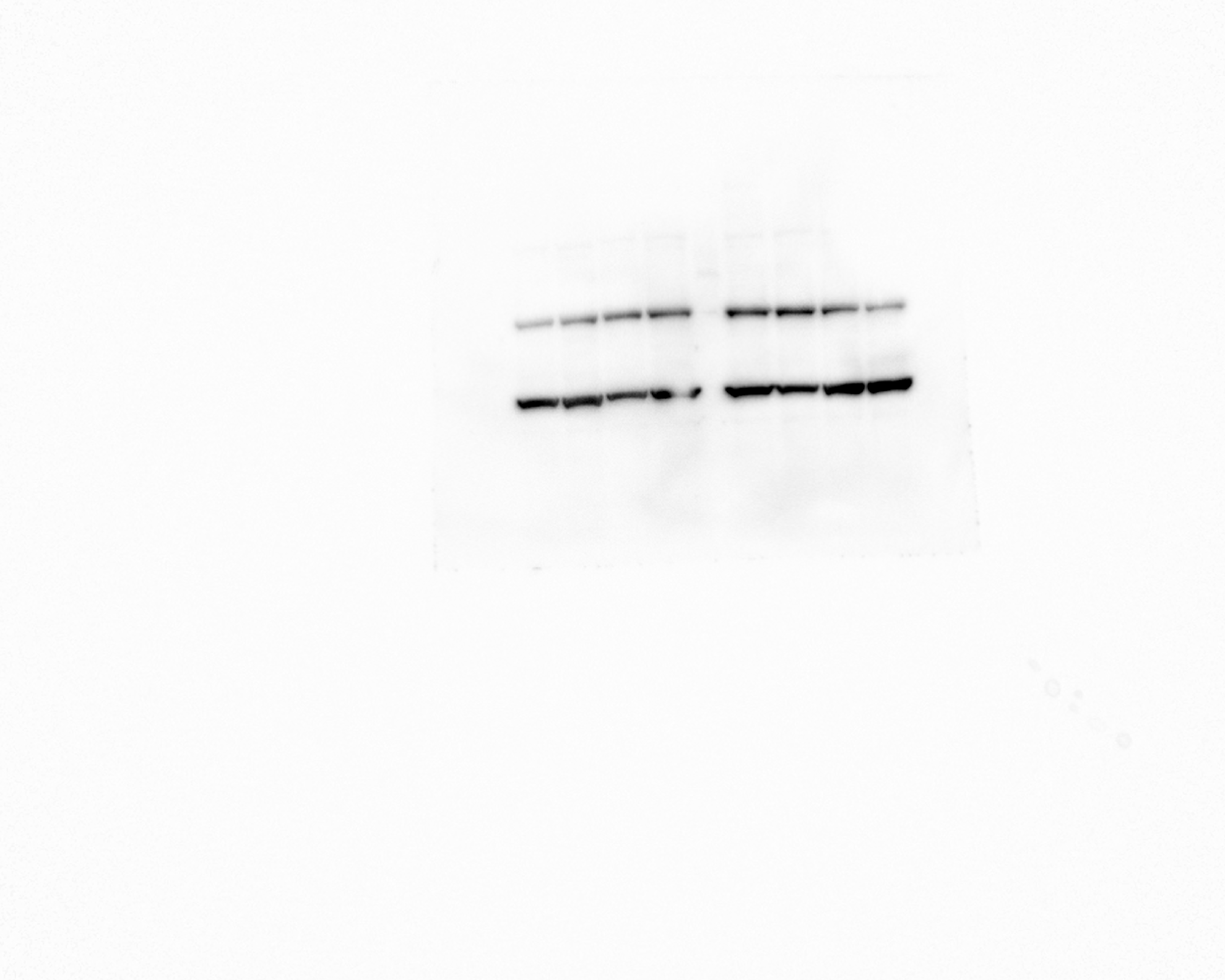

Supplement: Multimedia component 1 [file mmc1.zip › WB bands & raw densitometry/WB bands(45min)/6.(P-)NF-kB/B-actin(NF-kB)(1-2)(Chemiluminescence).tif]

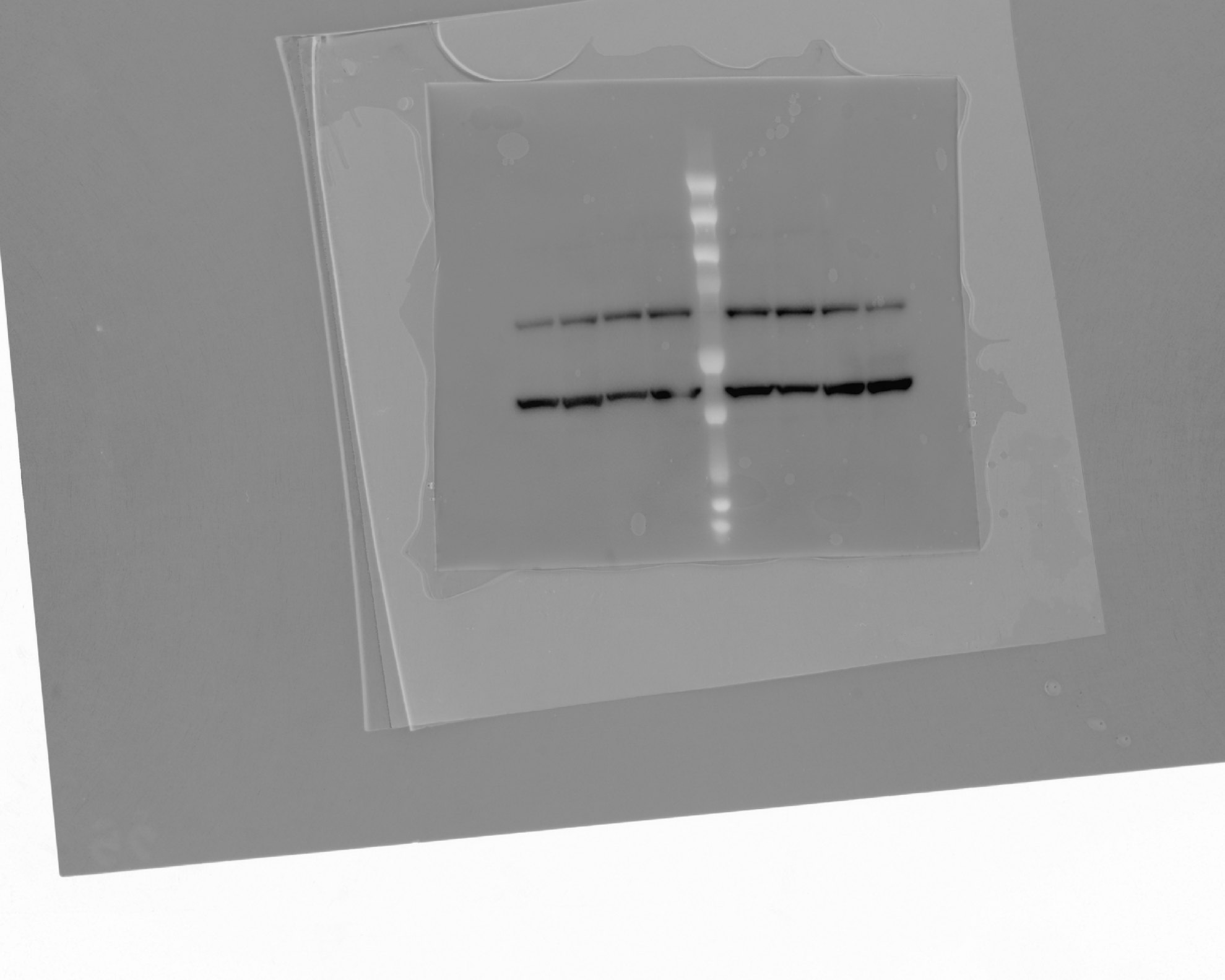

Supplement: Multimedia component 1 [file mmc1.zip › WB bands & raw densitometry/WB bands(45min)/6.(P-)NF-kB/B-actin(NF-kB)(1-2)(Composite).tif]

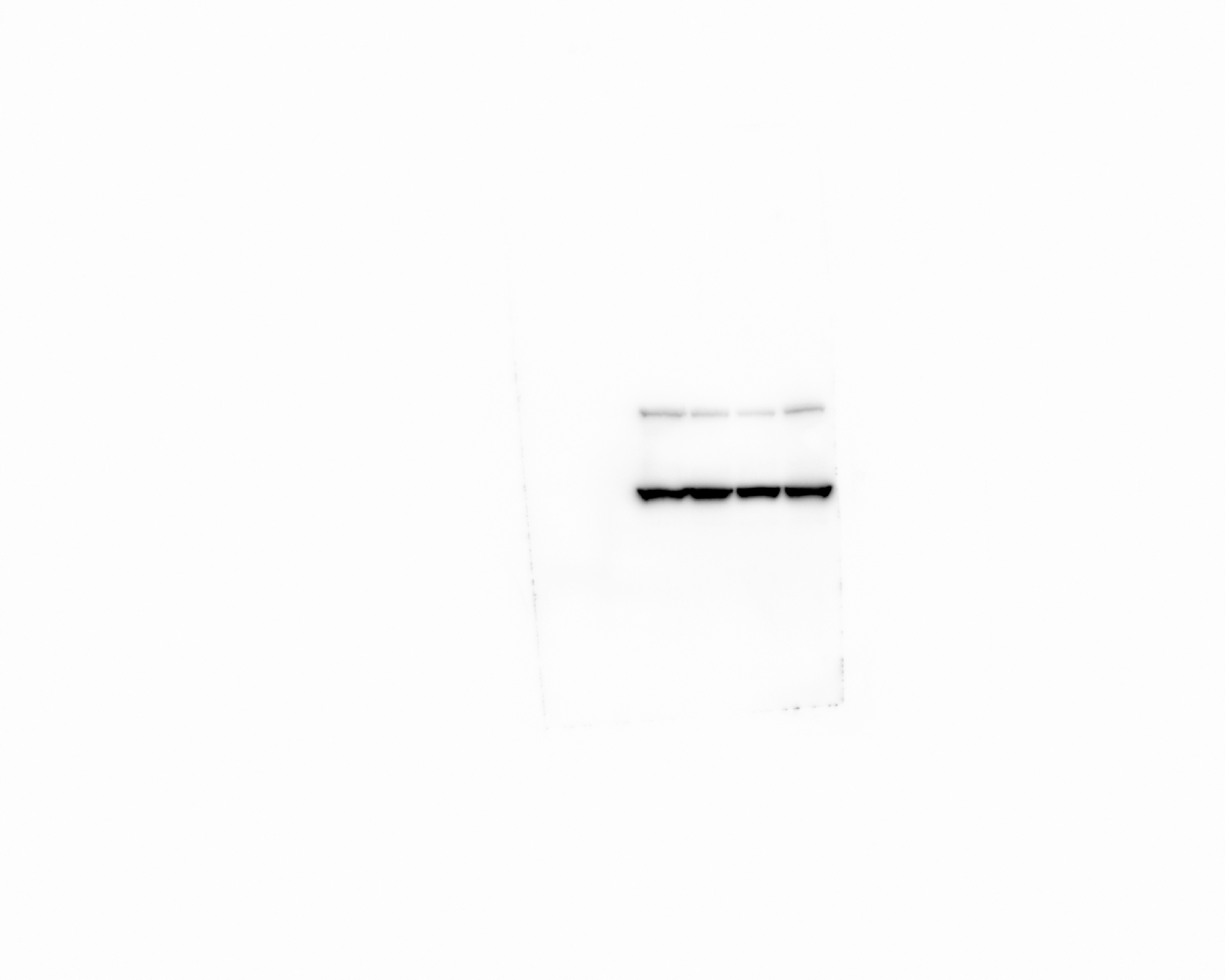

Supplement: Multimedia component 1 [file mmc1.zip › WB bands & raw densitometry/WB bands(45min)/6.(P-)NF-kB/B-actin(NF-kB)(2)(Chemiluminescence).tif]

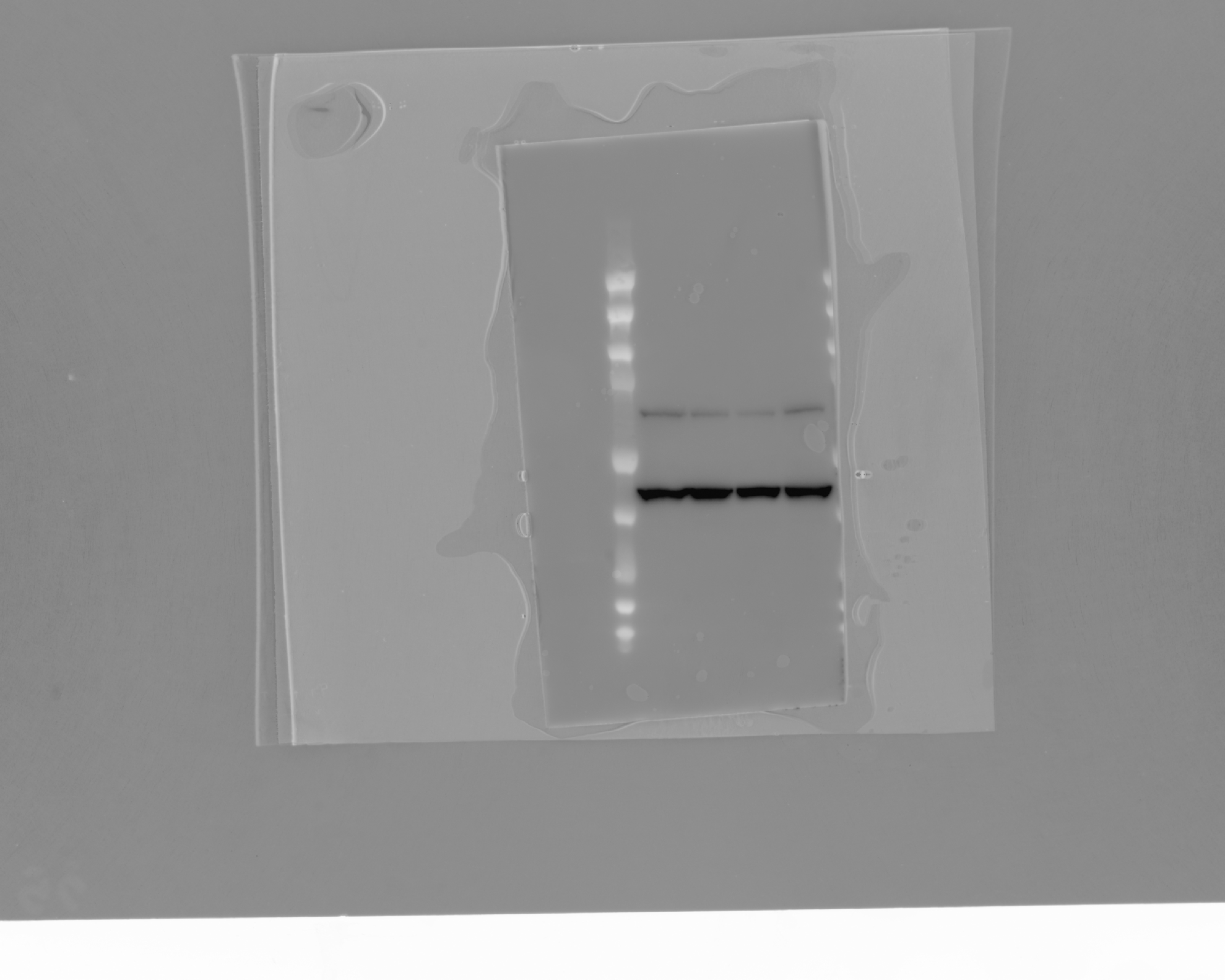

Supplement: Multimedia component 1 [file mmc1.zip › WB bands & raw densitometry/WB bands(45min)/6.(P-)NF-kB/B-actin(NF-kB)(2)(Composite).tif]

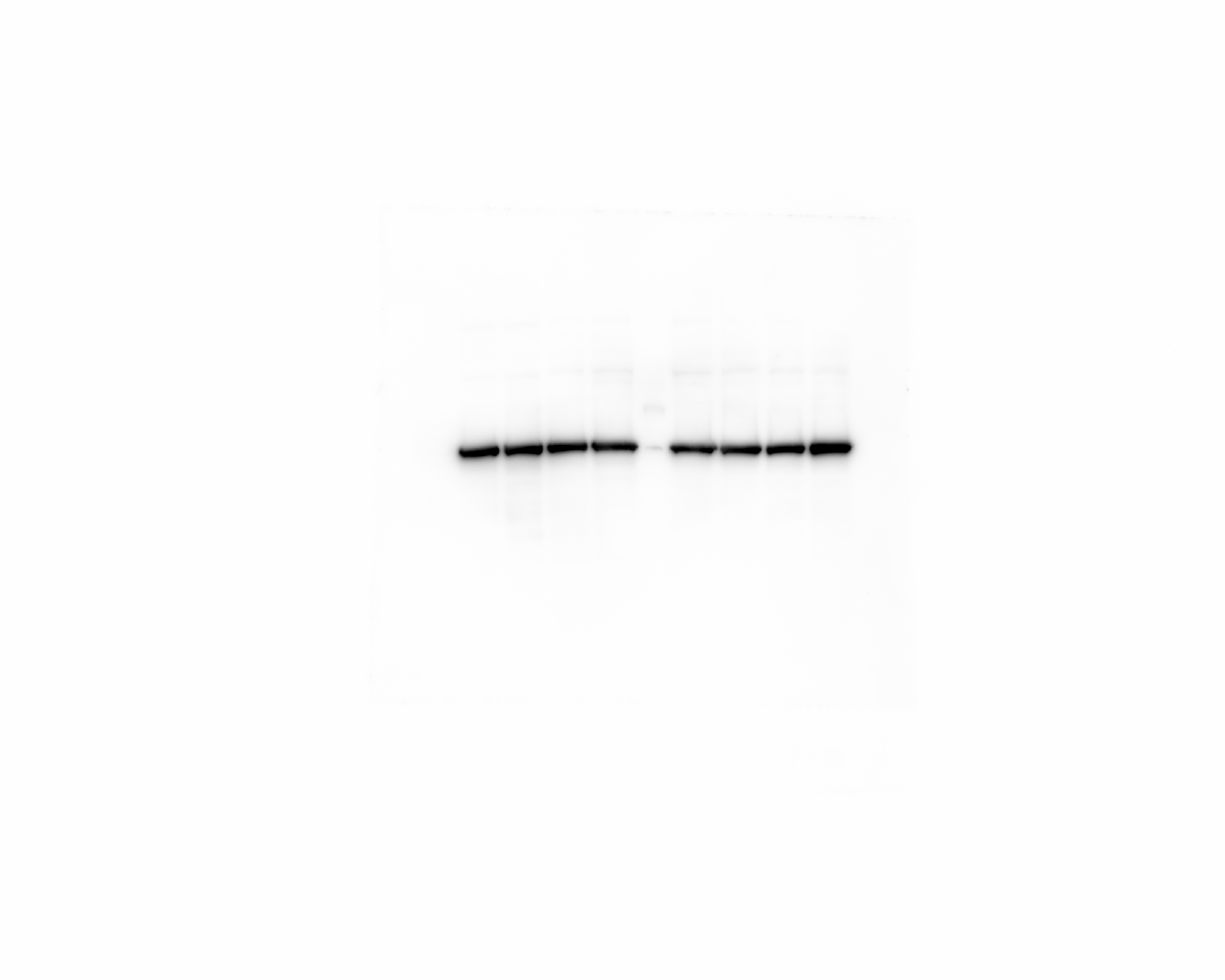

Supplement: Multimedia component 1 [file mmc1.zip › WB bands & raw densitometry/WB bands(45min)/6.(P-)NF-kB/NF-kB(1)(Chemiluminescence).tif]

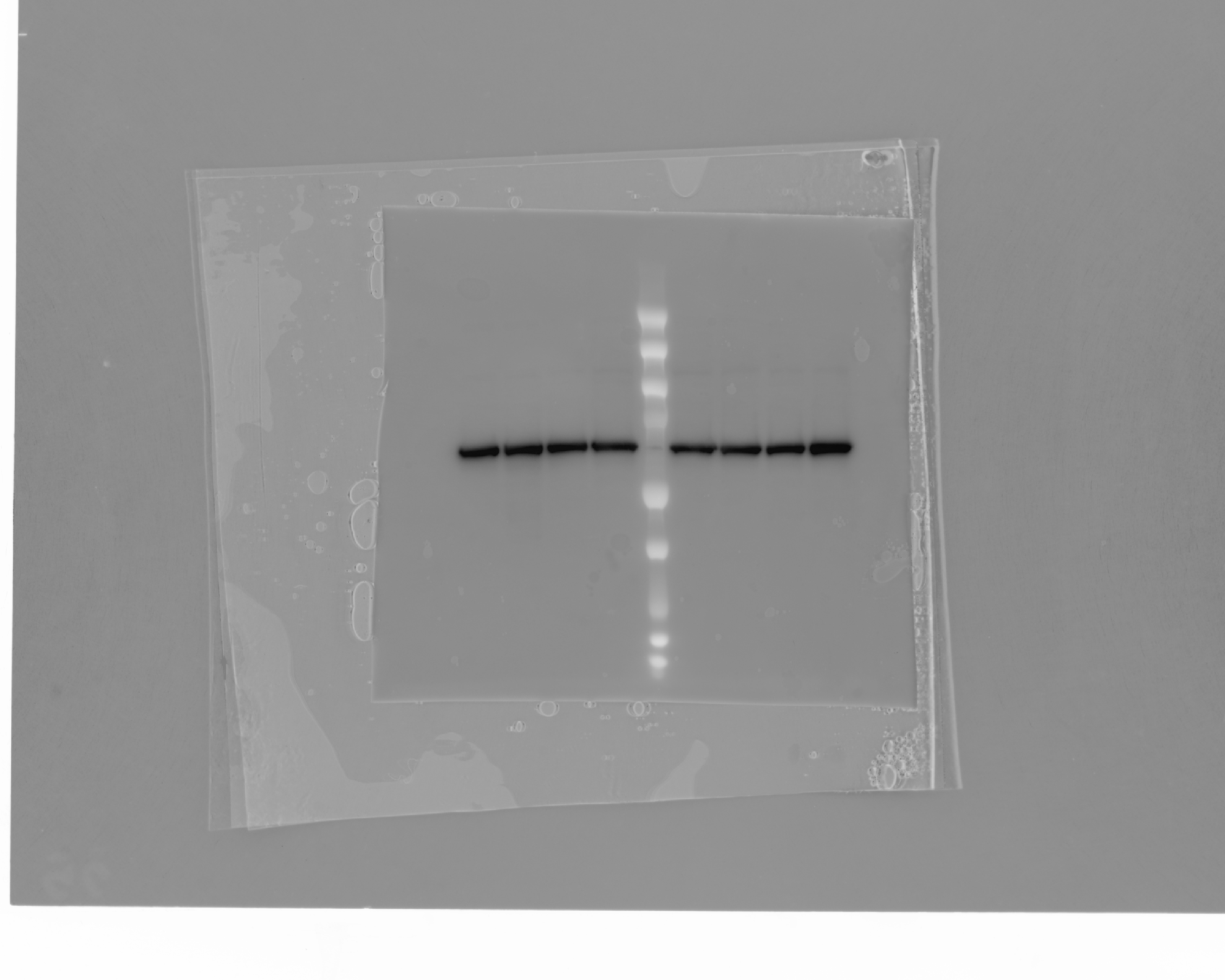

Supplement: Multimedia component 1 [file mmc1.zip › WB bands & raw densitometry/WB bands(45min)/6.(P-)NF-kB/NF-kB(1)(Composite).tif]

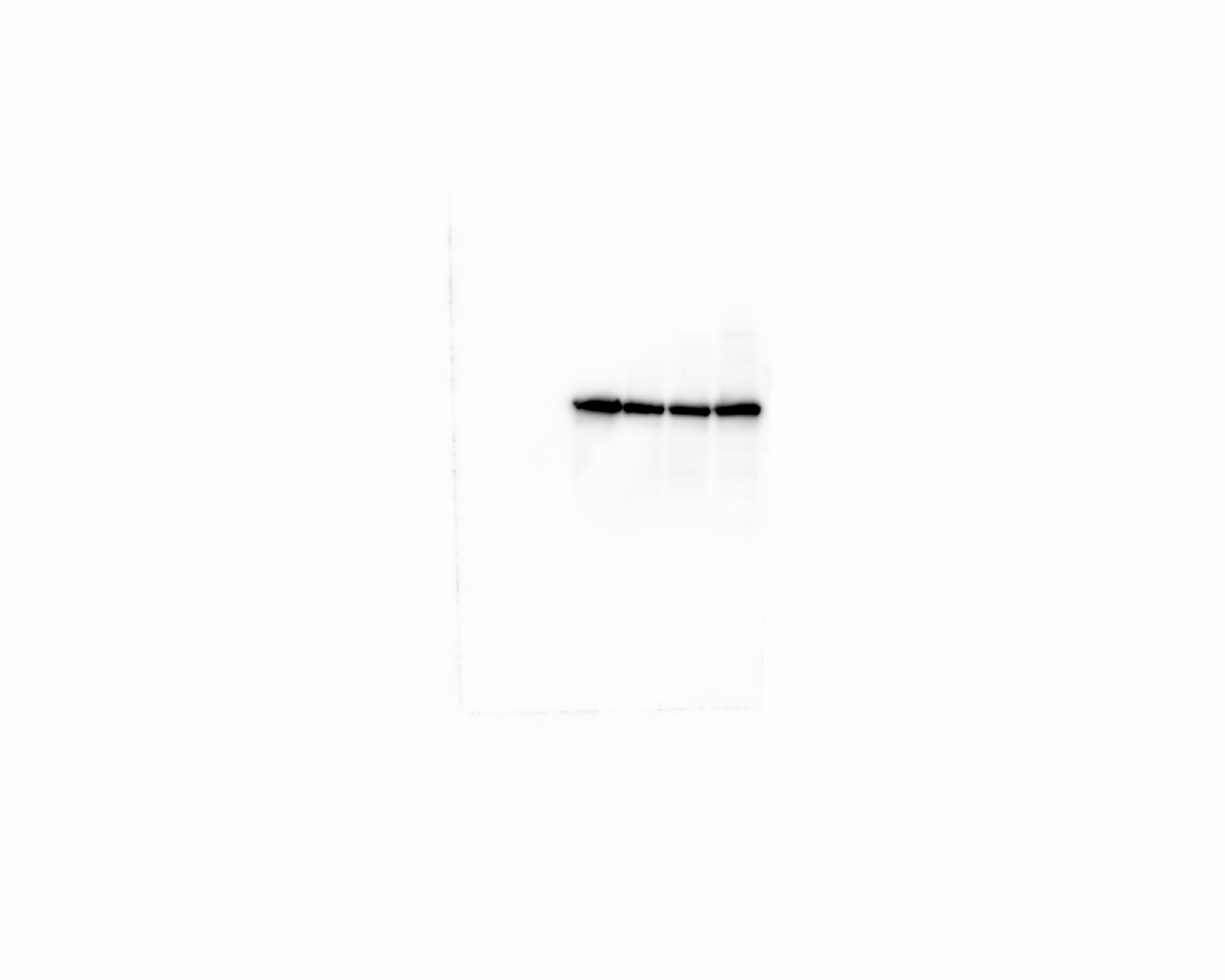

Supplement: Multimedia component 1 [file mmc1.zip › WB bands & raw densitometry/WB bands(45min)/6.(P-)NF-kB/NF-kB(2)(Chemiluminescence).tif]

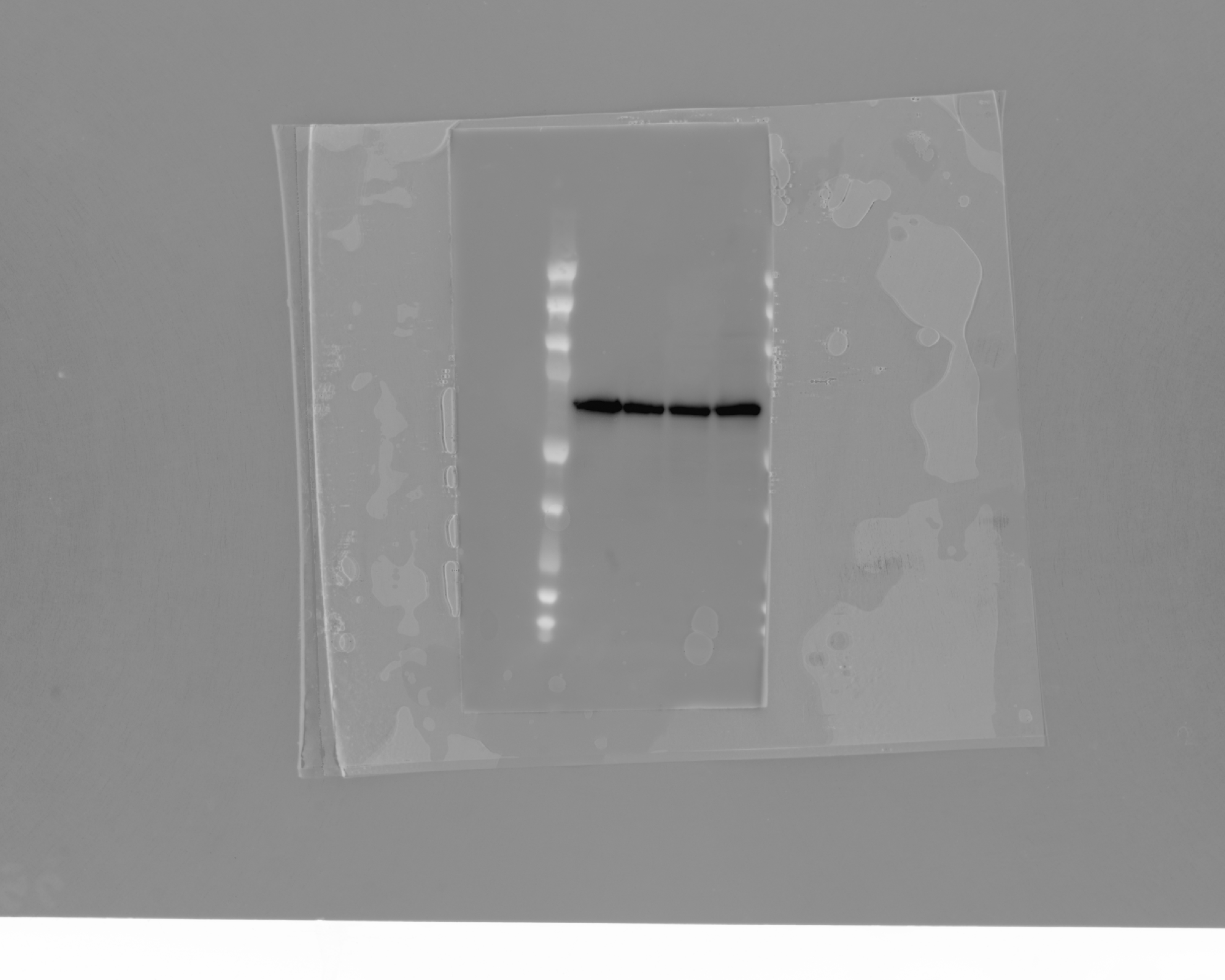

Supplement: Multimedia component 1 [file mmc1.zip › WB bands & raw densitometry/WB bands(45min)/6.(P-)NF-kB/NF-kB(2)(Composite).tif]

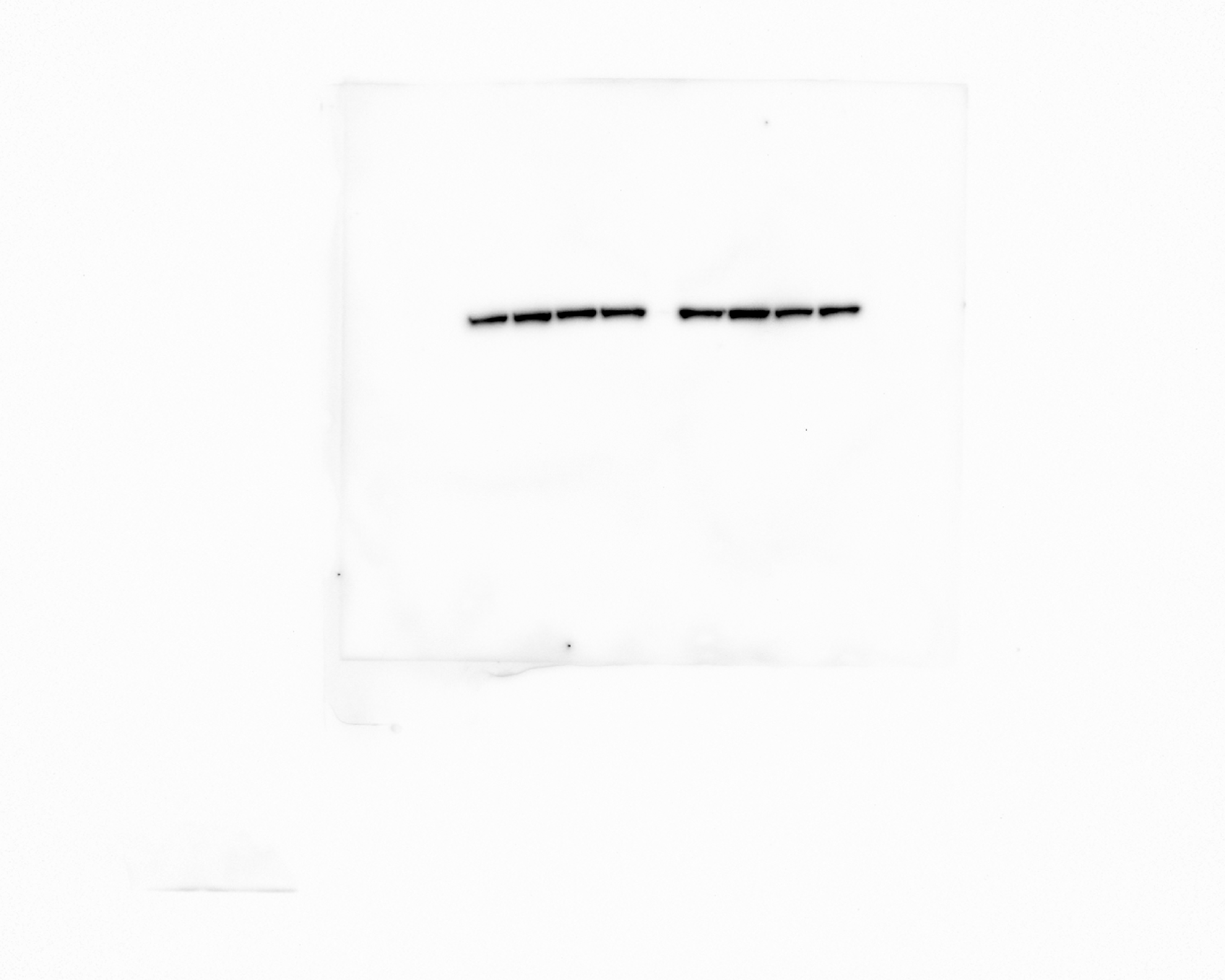

Supplement: Multimedia component 1 [file mmc1.zip › WB bands & raw densitometry/WB bands(45min)/6.(P-)NF-kB/p-NF-KB(1)(Chemiluminescence).tif]

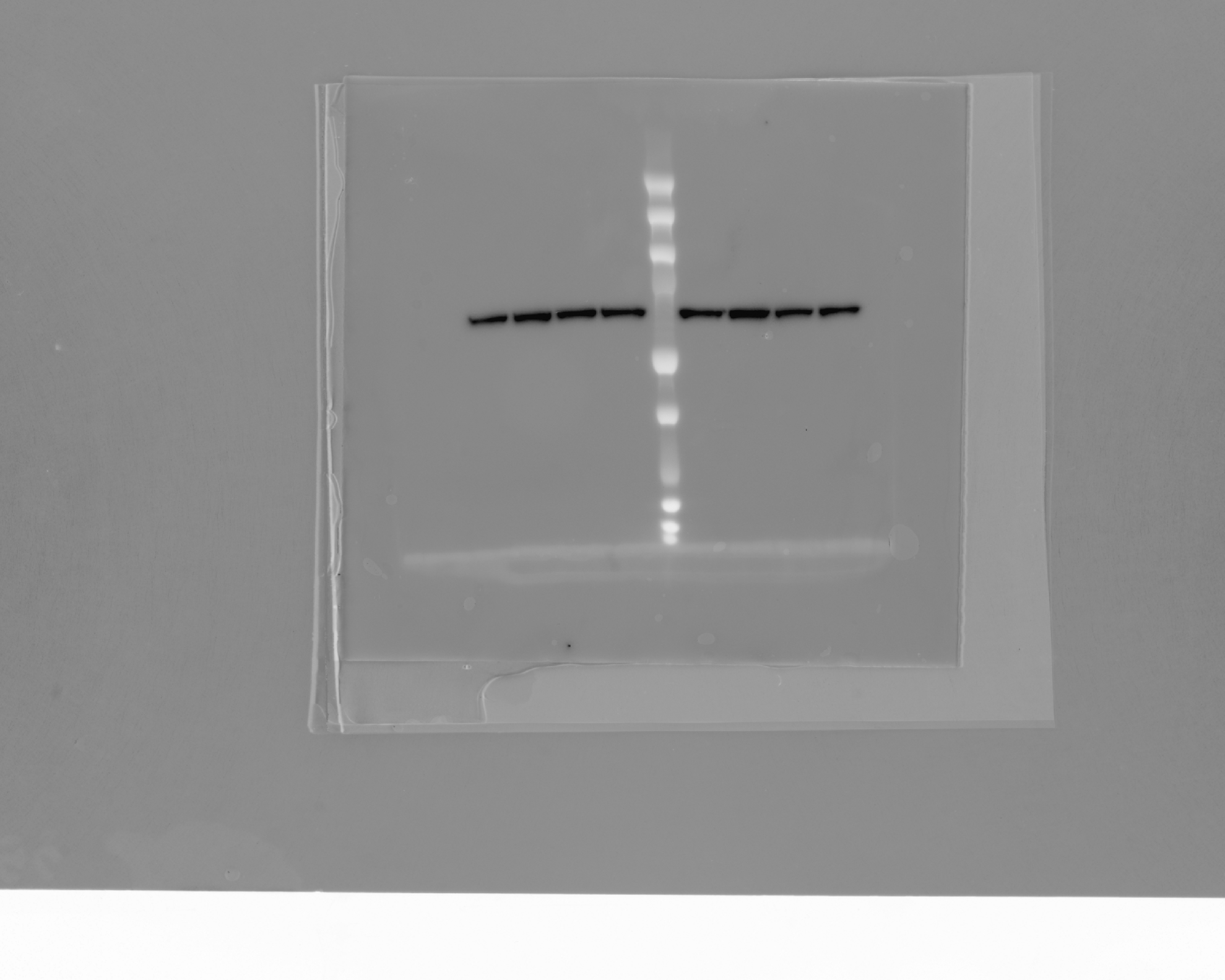

Supplement: Multimedia component 1 [file mmc1.zip › WB bands & raw densitometry/WB bands(45min)/6.(P-)NF-kB/p-NF-KB(1)(Composite).tif]

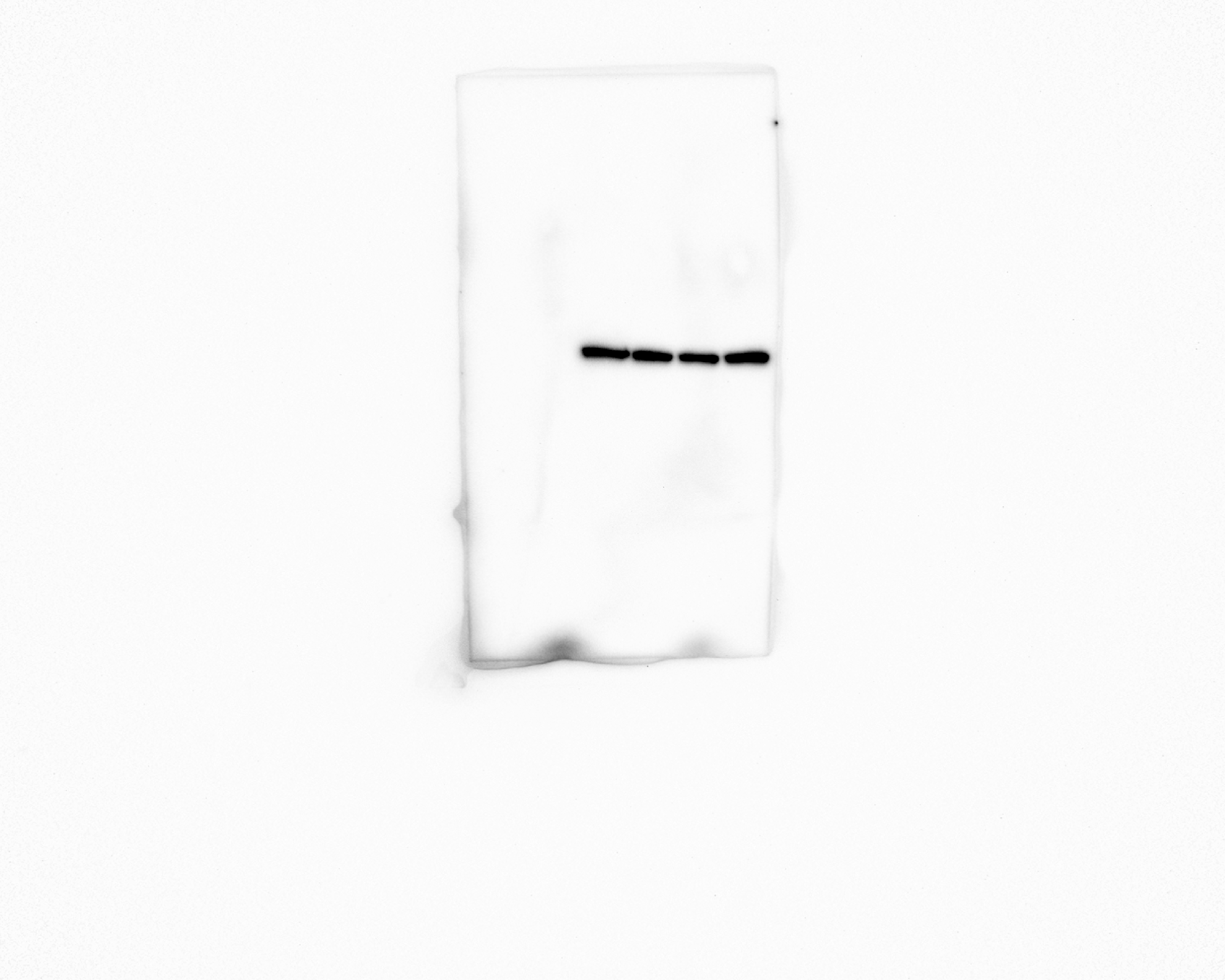

Supplement: Multimedia component 1 [file mmc1.zip › WB bands & raw densitometry/WB bands(45min)/6.(P-)NF-kB/p-NF-kB(2)(Chemiluminescence).tif]

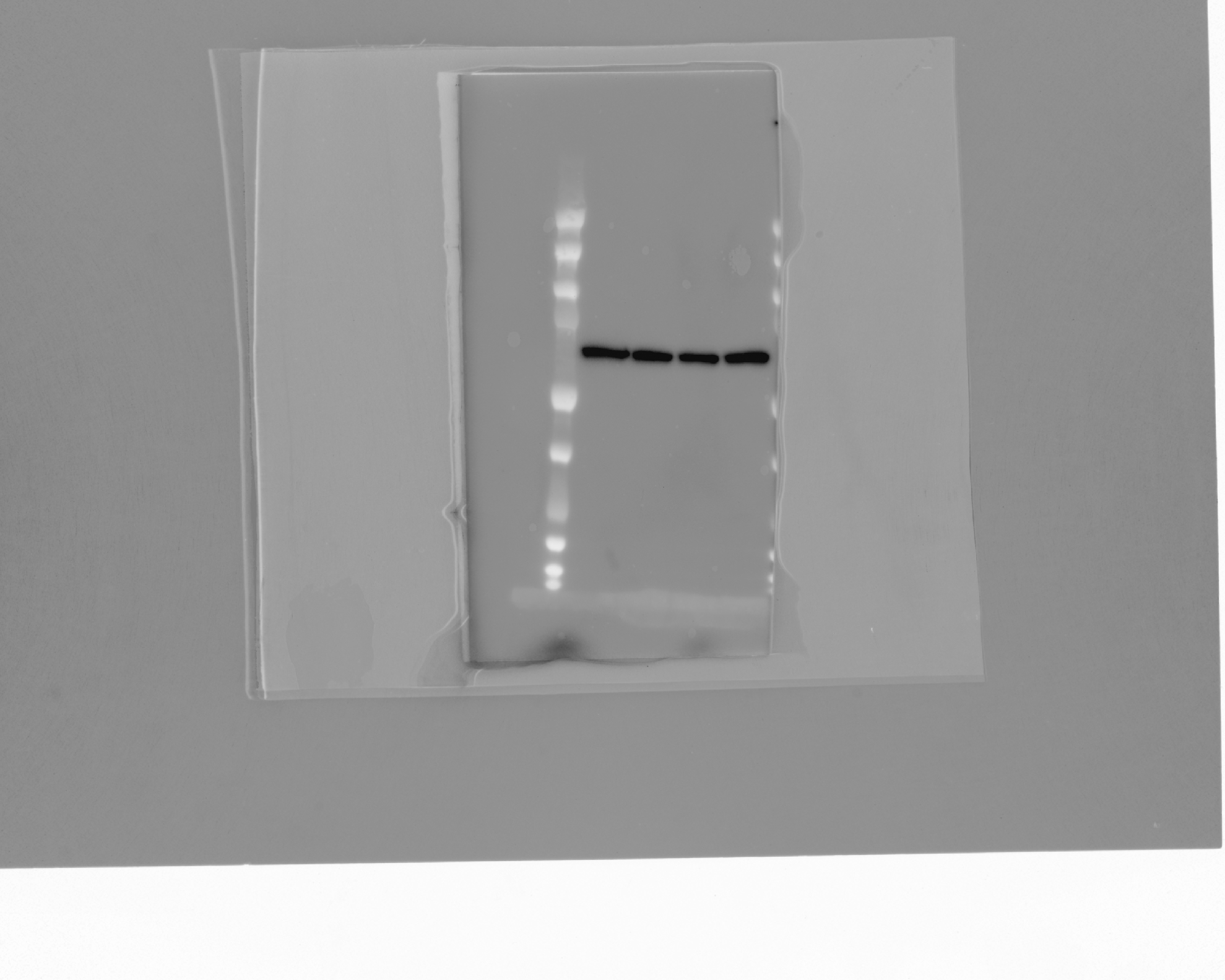

Supplement: Multimedia component 1 [file mmc1.zip › WB bands & raw densitometry/WB bands(45min)/6.(P-)NF-kB/p-NF-kB(2)(Composite).tif]
