# Supplementary figures and images for: Effects of branched-chain amino acids on iron deficiency-induced muscle atrophy (part 1 of 2)
Source: Biochem Biophys Rep. 2026 Jan 17;45:102451. doi: 10.1016/j.bbrep.2026.102451 (PMC12854053; doi:10.1016/j.bbrep.2026.102451)

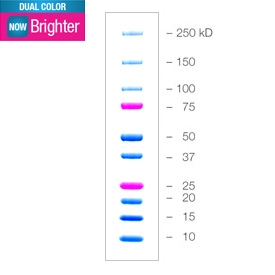

Supplement: Multimedia component 1 [file mmc1.zip › WB bands & raw densitometry/standard(MW markers).jpg]

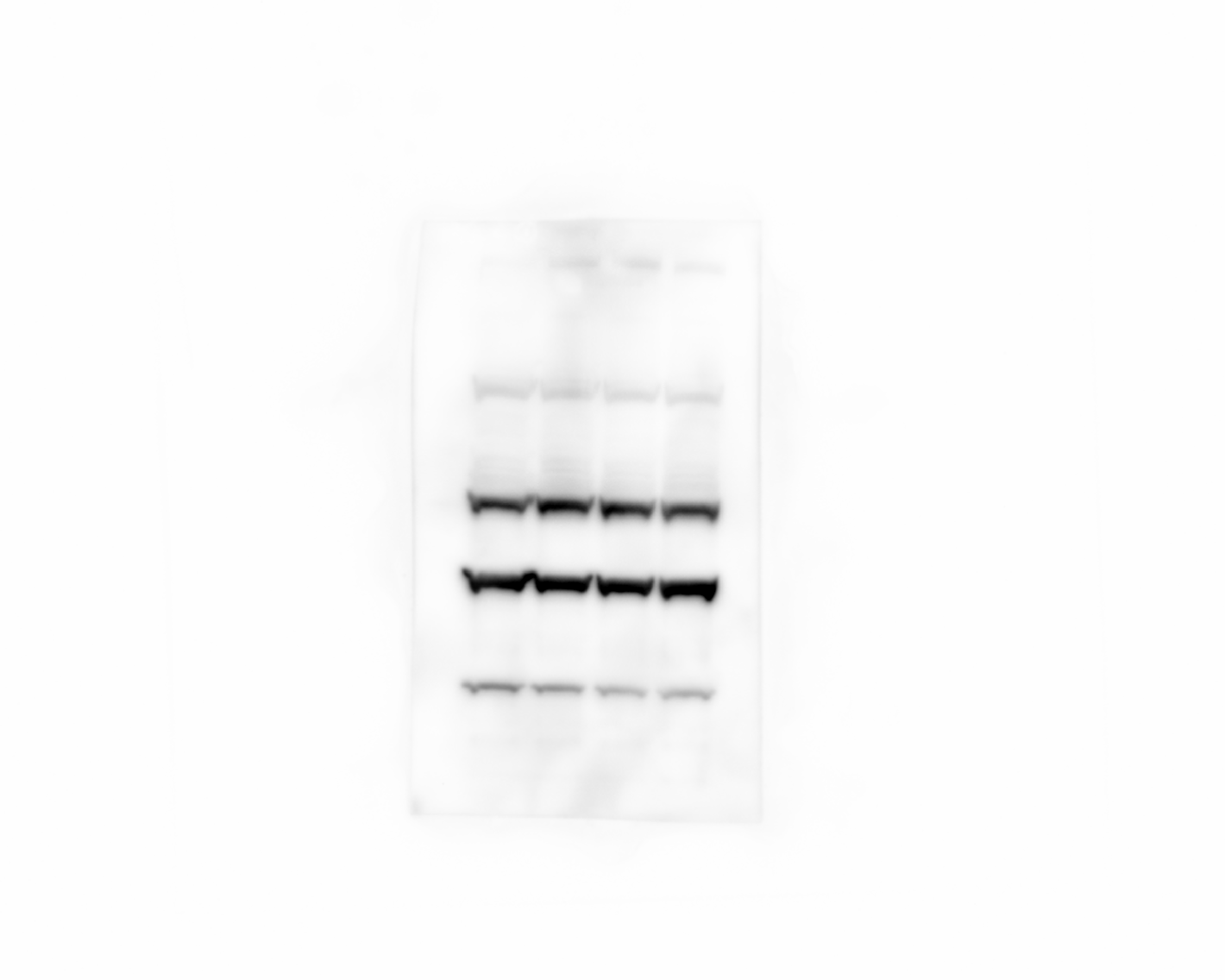

Supplement: Multimedia component 1 [file mmc1.zip › WB bands & raw densitometry/WB bands(24h)/1.B-actin(Representative)/1.Bactin(Chemiluminescence).tif]

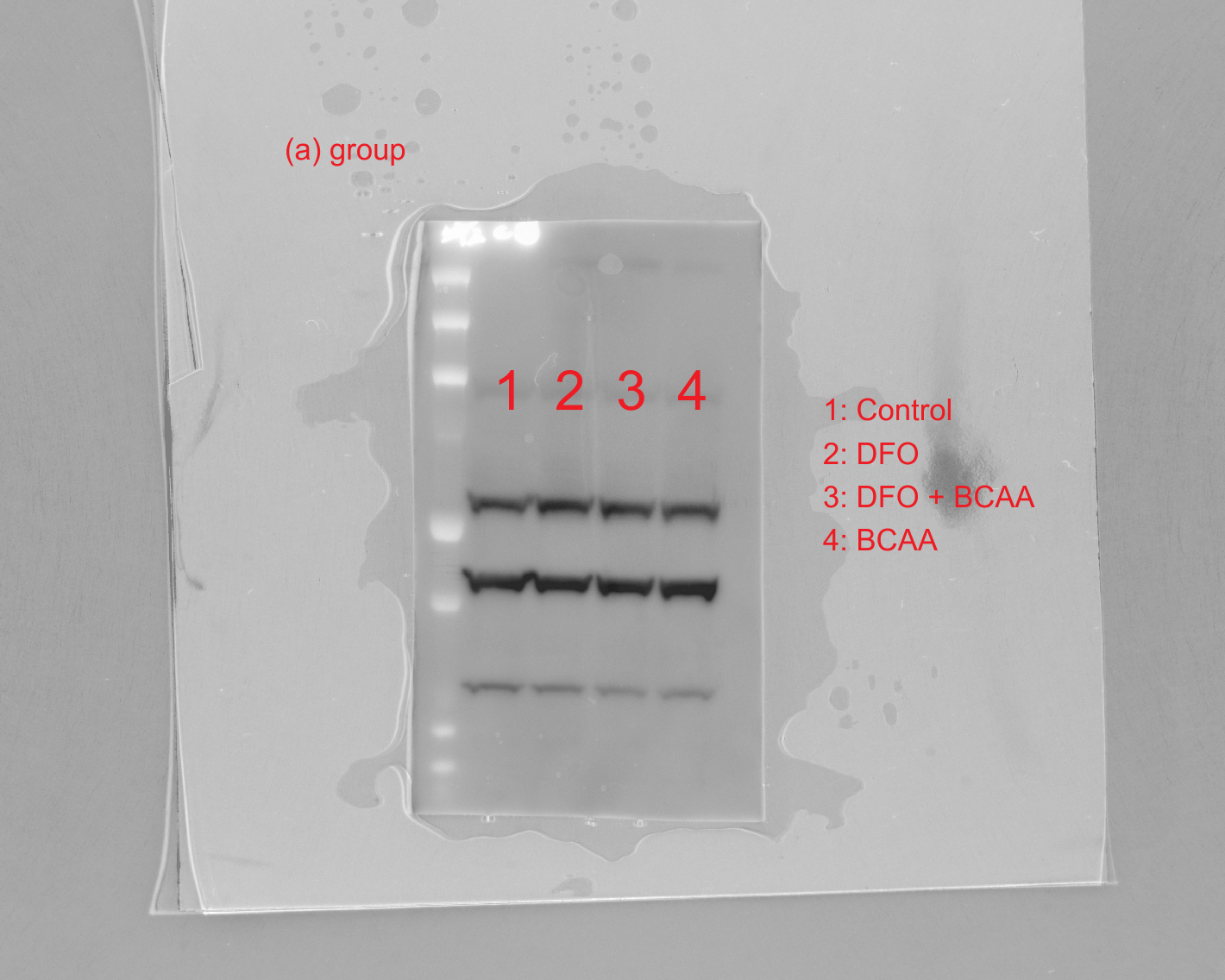

Supplement: Multimedia component 1 [file mmc1.zip › WB bands & raw densitometry/WB bands(24h)/1.B-actin(Representative)/1.Bactin(Composite).tif]

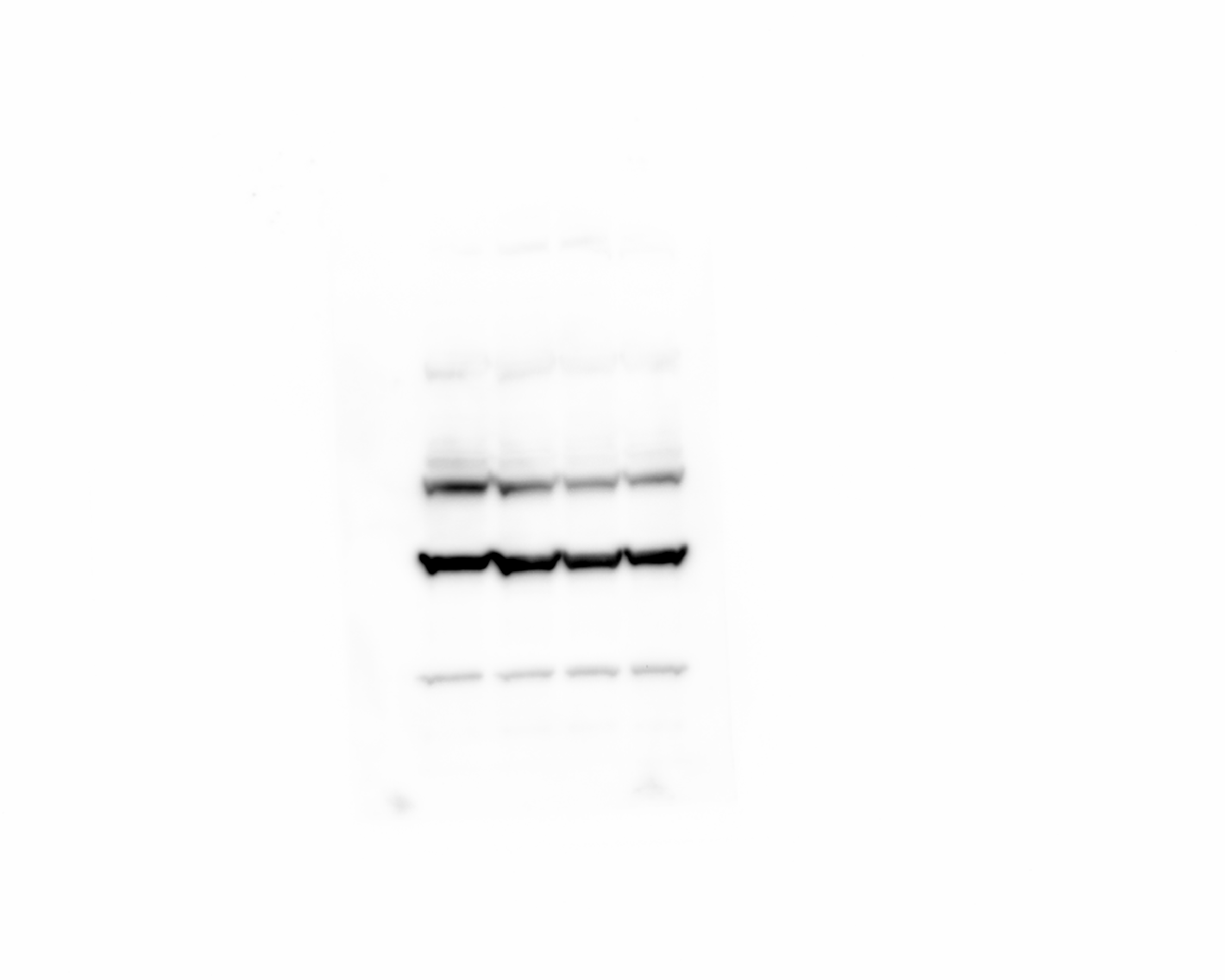

Supplement: Multimedia component 1 [file mmc1.zip › WB bands & raw densitometry/WB bands(24h)/1.B-actin(Representative)/2.Bactin(Chemiluminescence).tif]

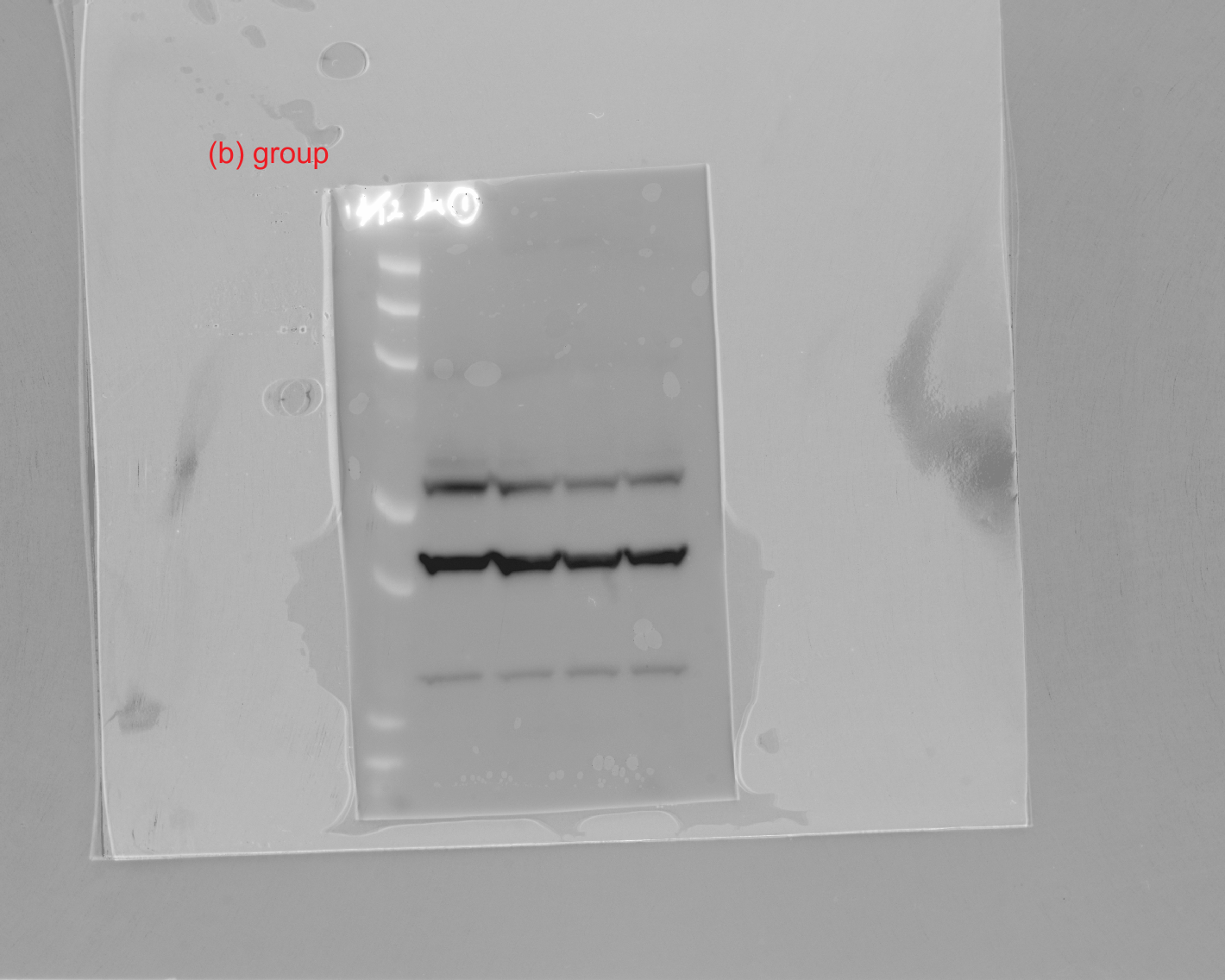

Supplement: Multimedia component 1 [file mmc1.zip › WB bands & raw densitometry/WB bands(24h)/1.B-actin(Representative)/2.Bactin(Composite).tif]

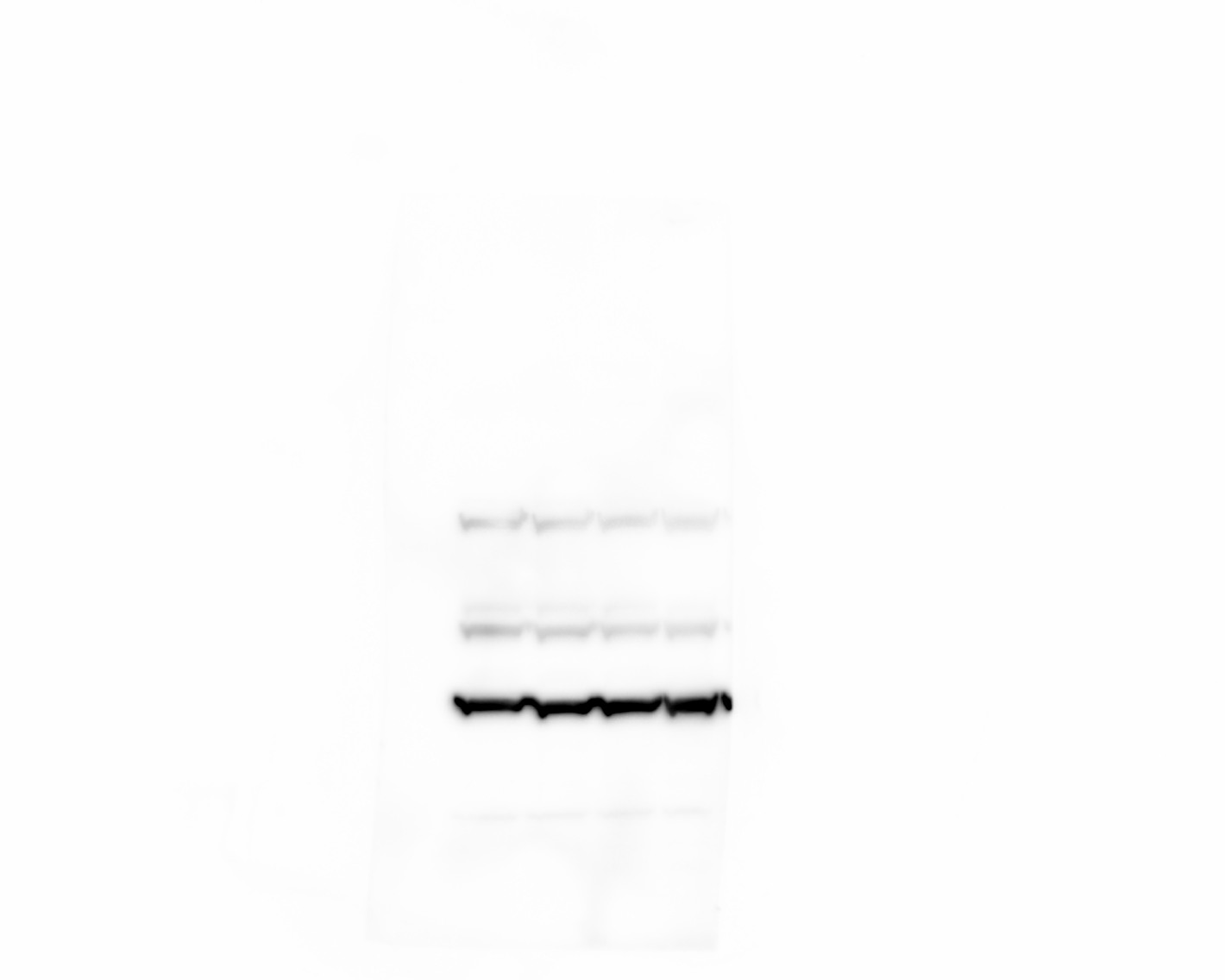

Supplement: Multimedia component 1 [file mmc1.zip › WB bands & raw densitometry/WB bands(24h)/1.B-actin(Representative)/3.Bactin(Chemiluminescence).tif]

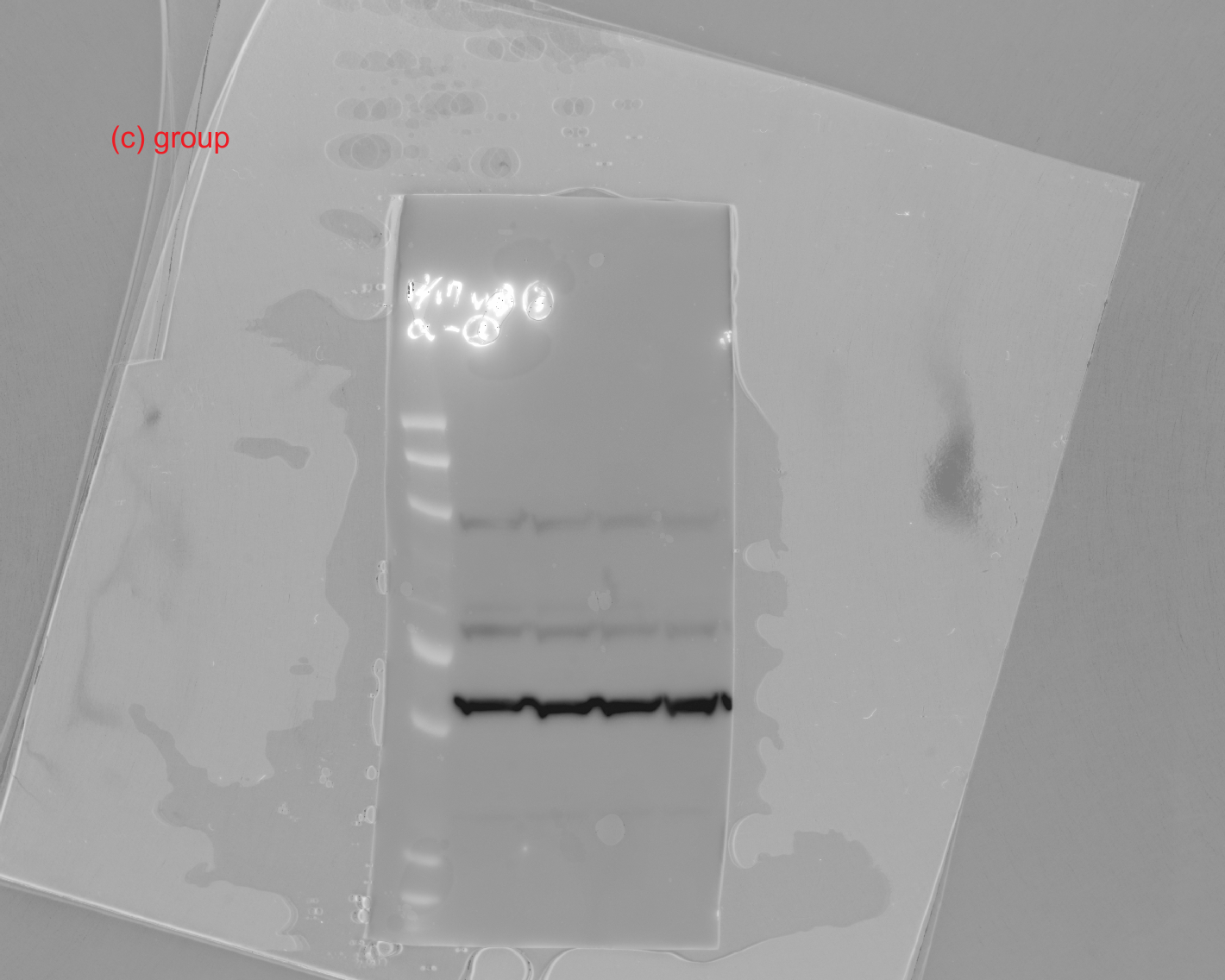

Supplement: Multimedia component 1 [file mmc1.zip › WB bands & raw densitometry/WB bands(24h)/1.B-actin(Representative)/3.Bactin(Composite).tif]

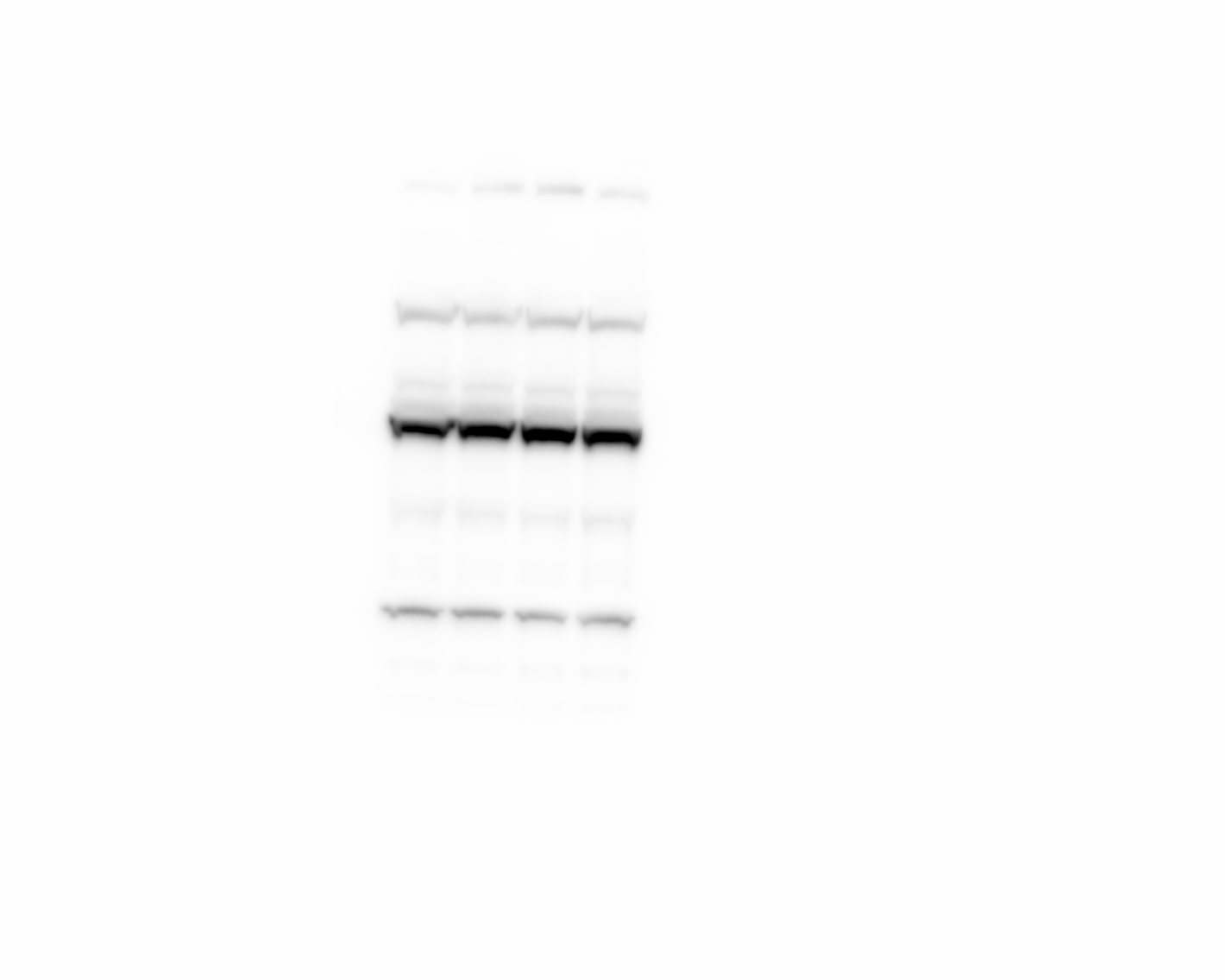

Supplement: Multimedia component 1 [file mmc1.zip › WB bands & raw densitometry/WB bands(24h)/2.Akt/1.Akt(Chemiluminescence).tif]

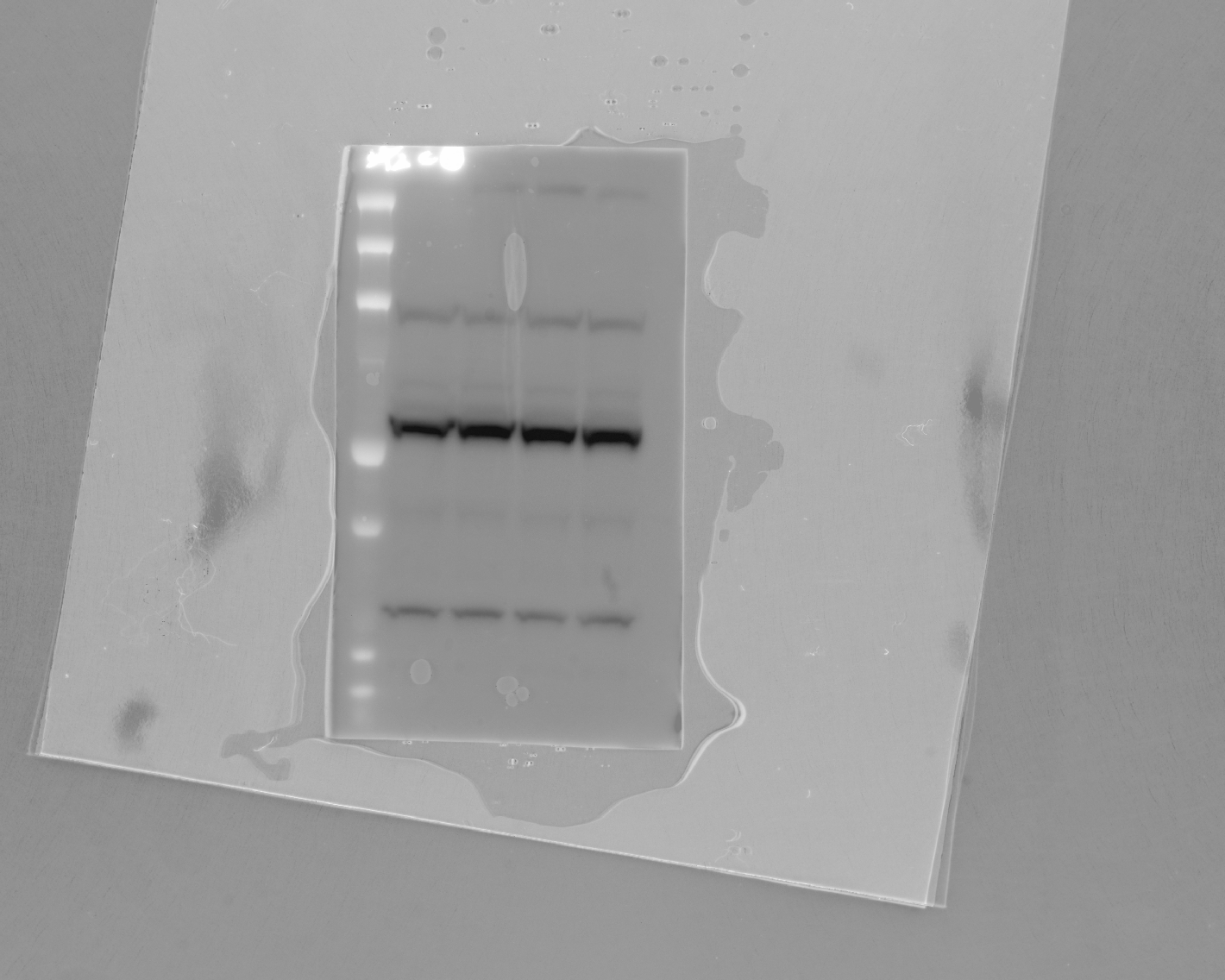

Supplement: Multimedia component 1 [file mmc1.zip › WB bands & raw densitometry/WB bands(24h)/2.Akt/1.Akt(Composite).tif]

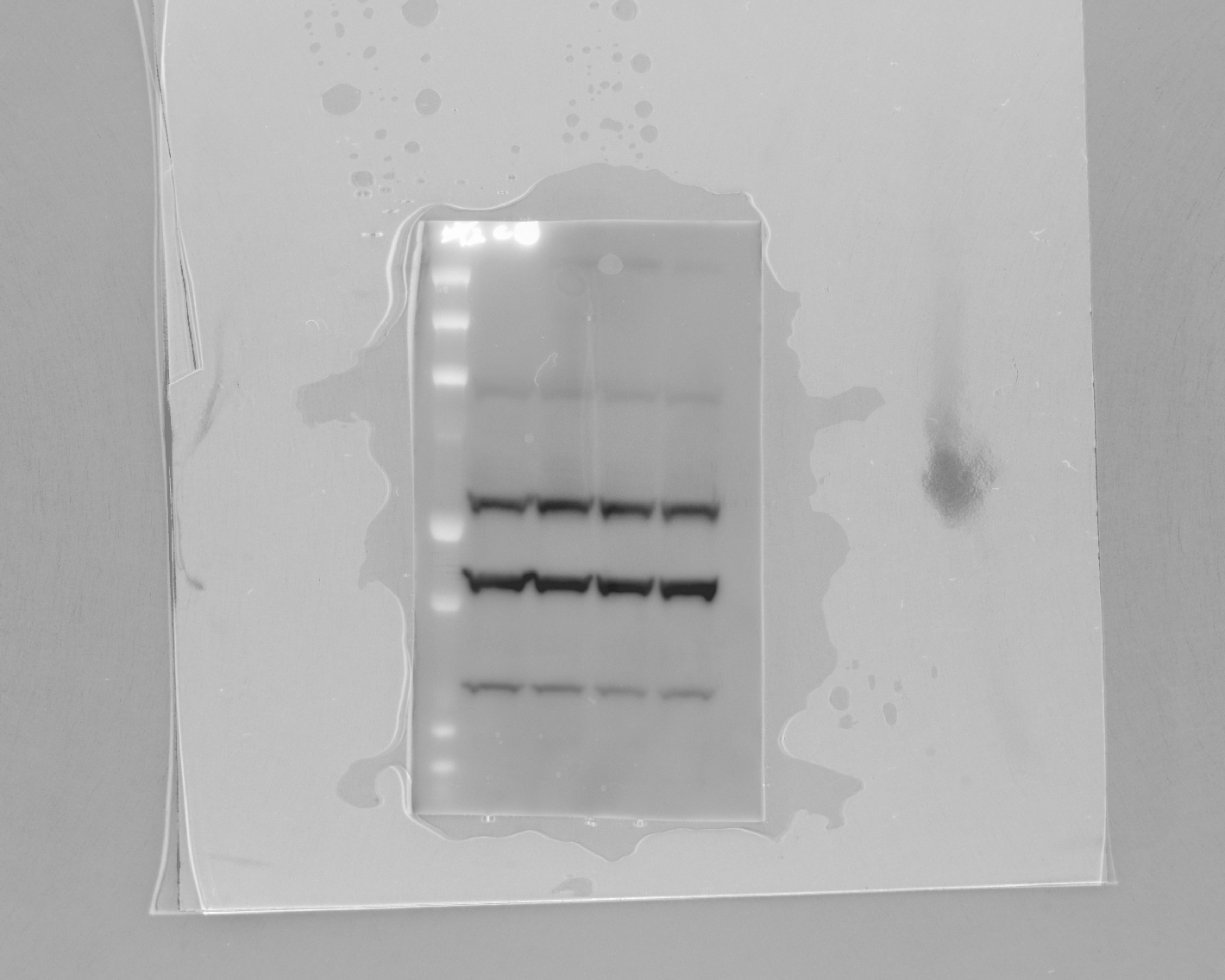

Supplement: Multimedia component 1 [file mmc1.zip › WB bands & raw densitometry/WB bands(24h)/2.Akt/1.Bactin(Composite).tif]

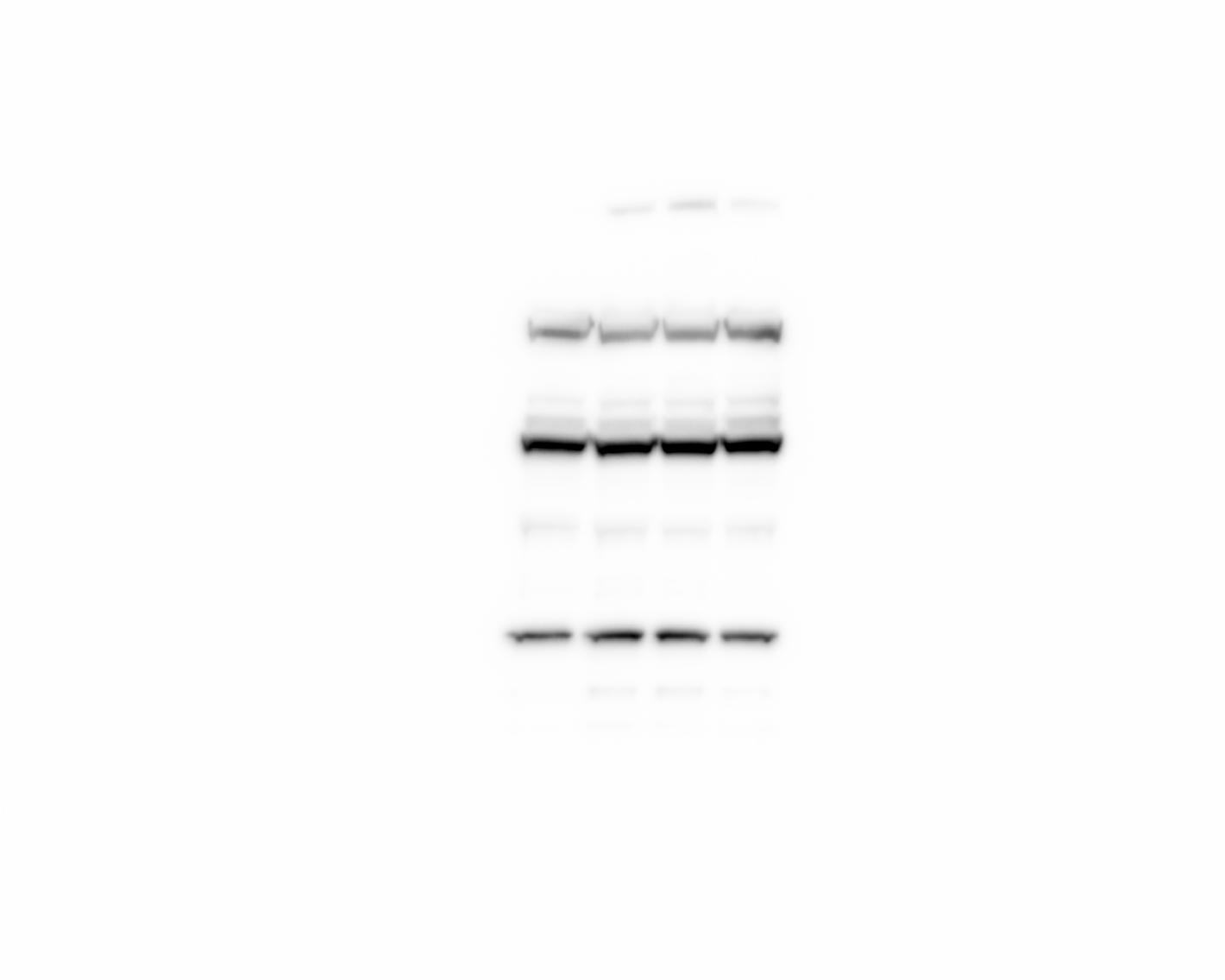

Supplement: Multimedia component 1 [file mmc1.zip › WB bands & raw densitometry/WB bands(24h)/2.Akt/2.Akt(Chemiluminescence).tif]

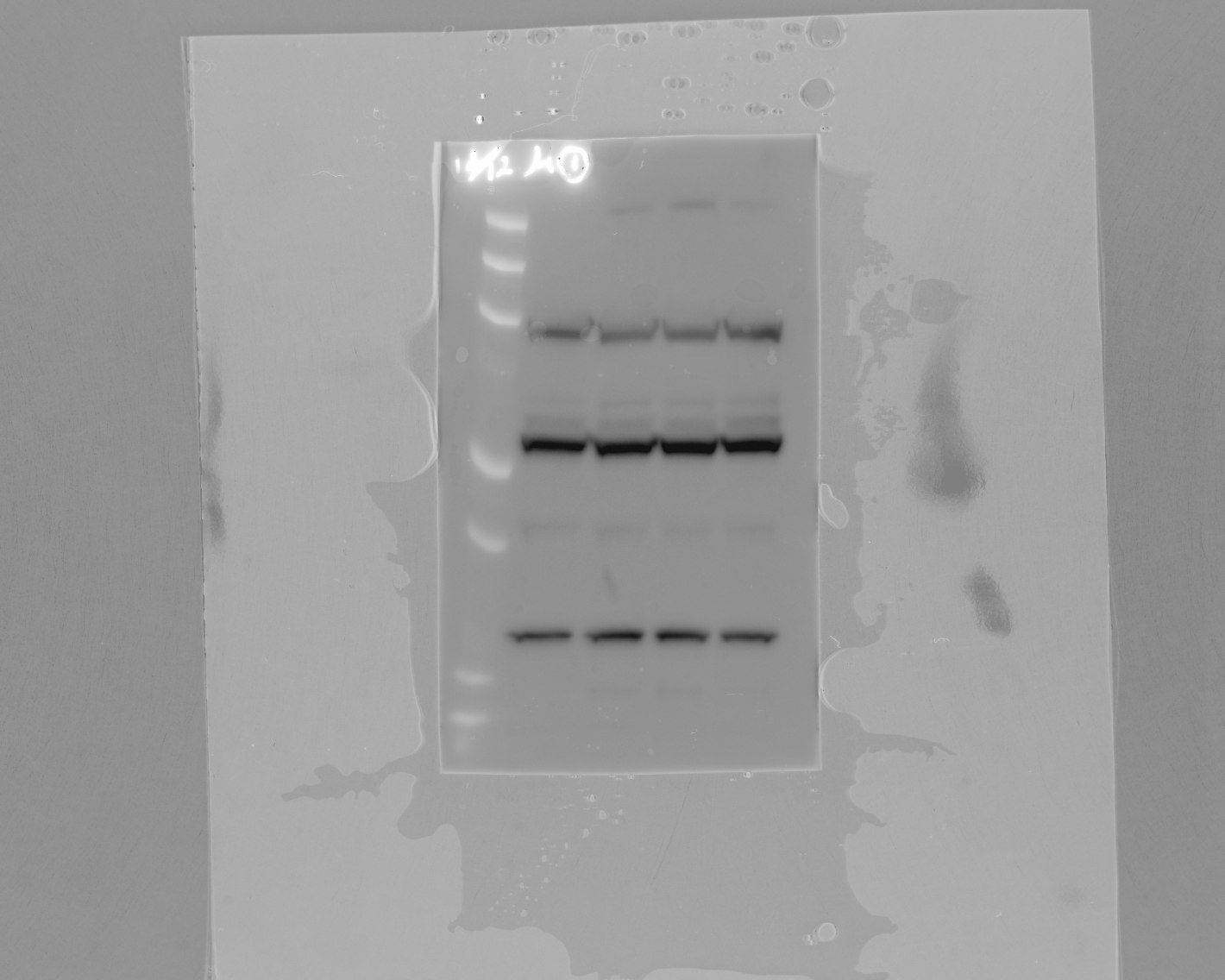

Supplement: Multimedia component 1 [file mmc1.zip › WB bands & raw densitometry/WB bands(24h)/2.Akt/2.Akt(Composite).tif]

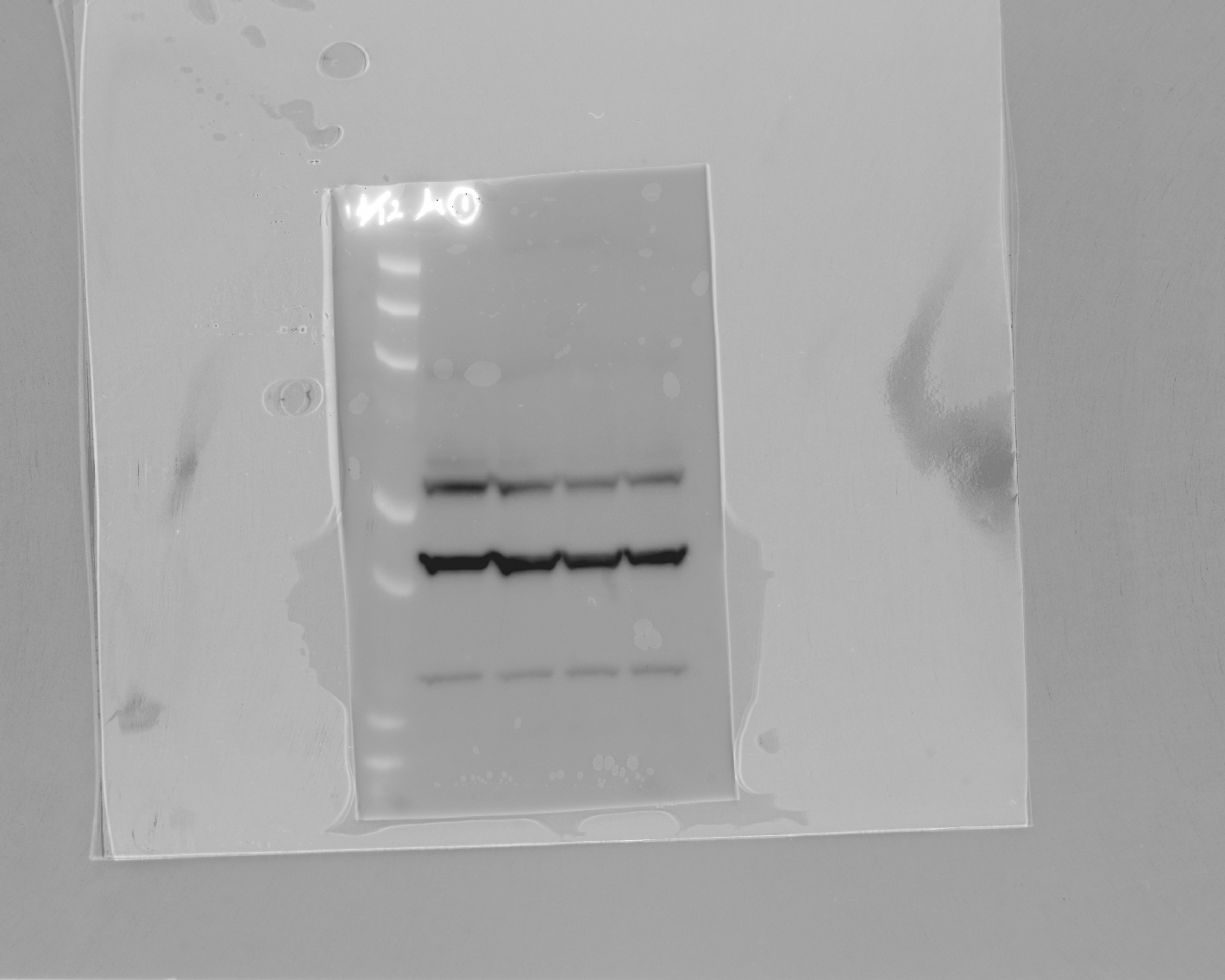

Supplement: Multimedia component 1 [file mmc1.zip › WB bands & raw densitometry/WB bands(24h)/2.Akt/2.Bactin(Composite).tif]

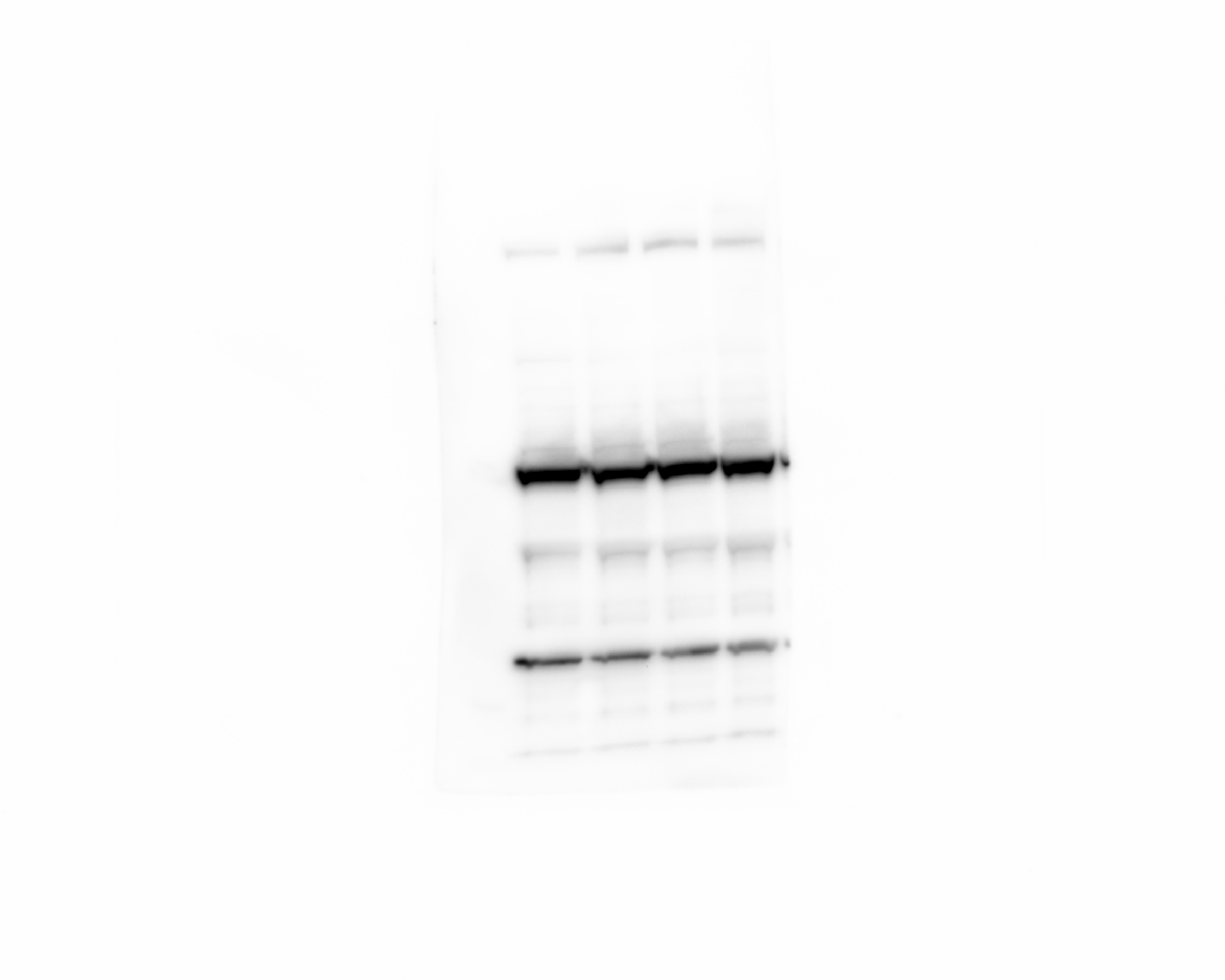

Supplement: Multimedia component 1 [file mmc1.zip › WB bands & raw densitometry/WB bands(24h)/2.Akt/3.Akt(Chemiluminescence).tif]

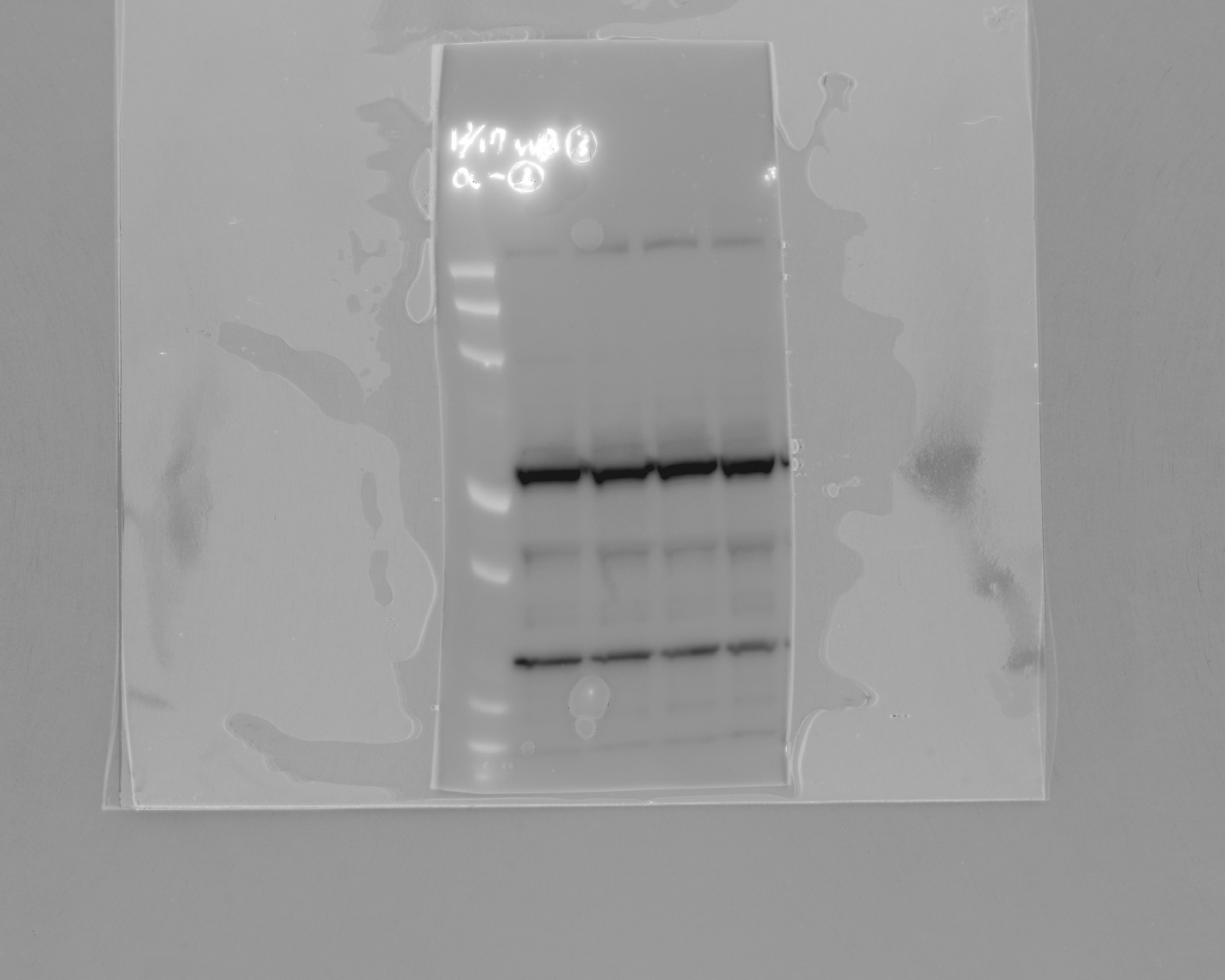

Supplement: Multimedia component 1 [file mmc1.zip › WB bands & raw densitometry/WB bands(24h)/2.Akt/3.Akt(Composite).tif]

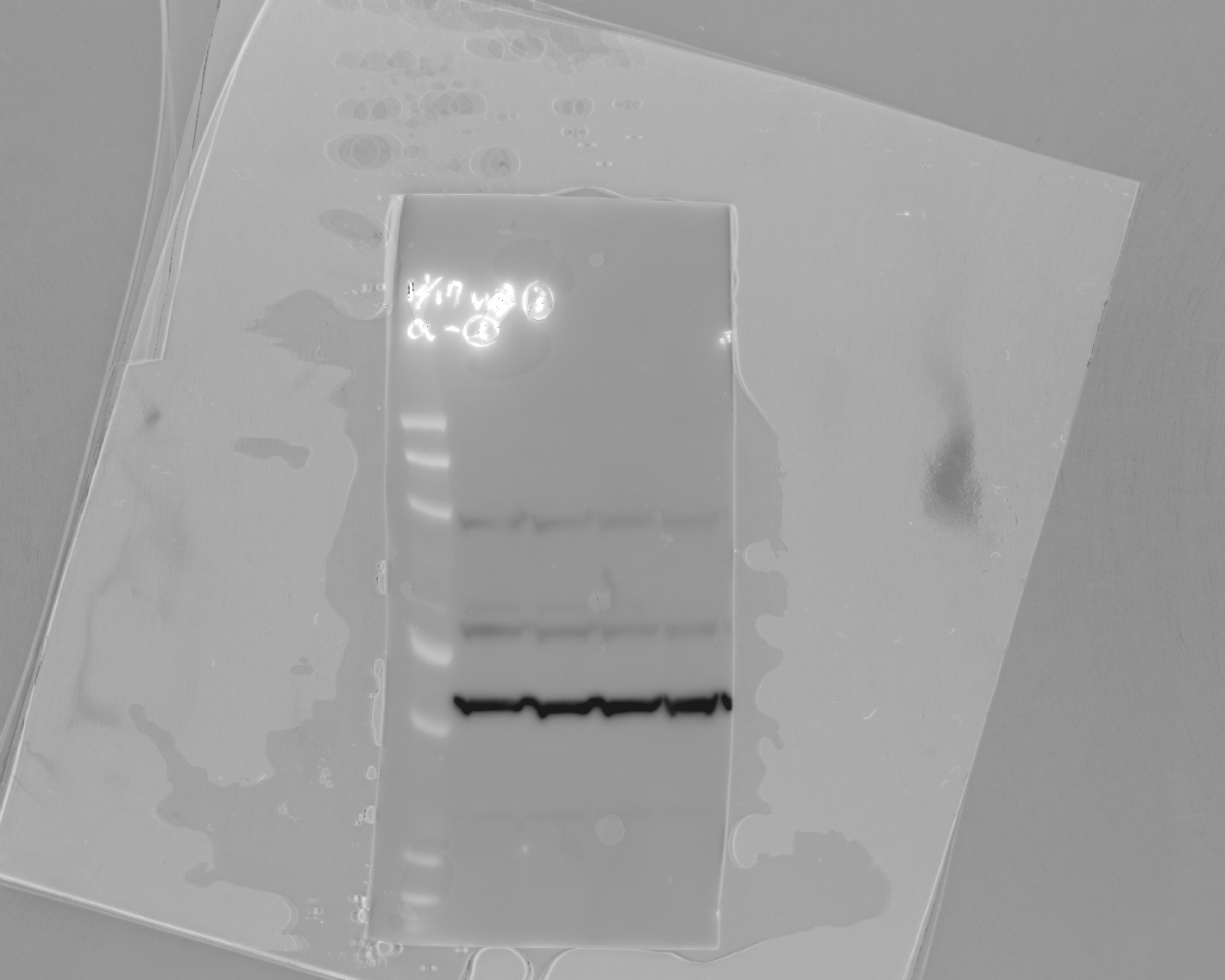

Supplement: Multimedia component 1 [file mmc1.zip › WB bands & raw densitometry/WB bands(24h)/2.Akt/3.Bactin(Composite).tif]

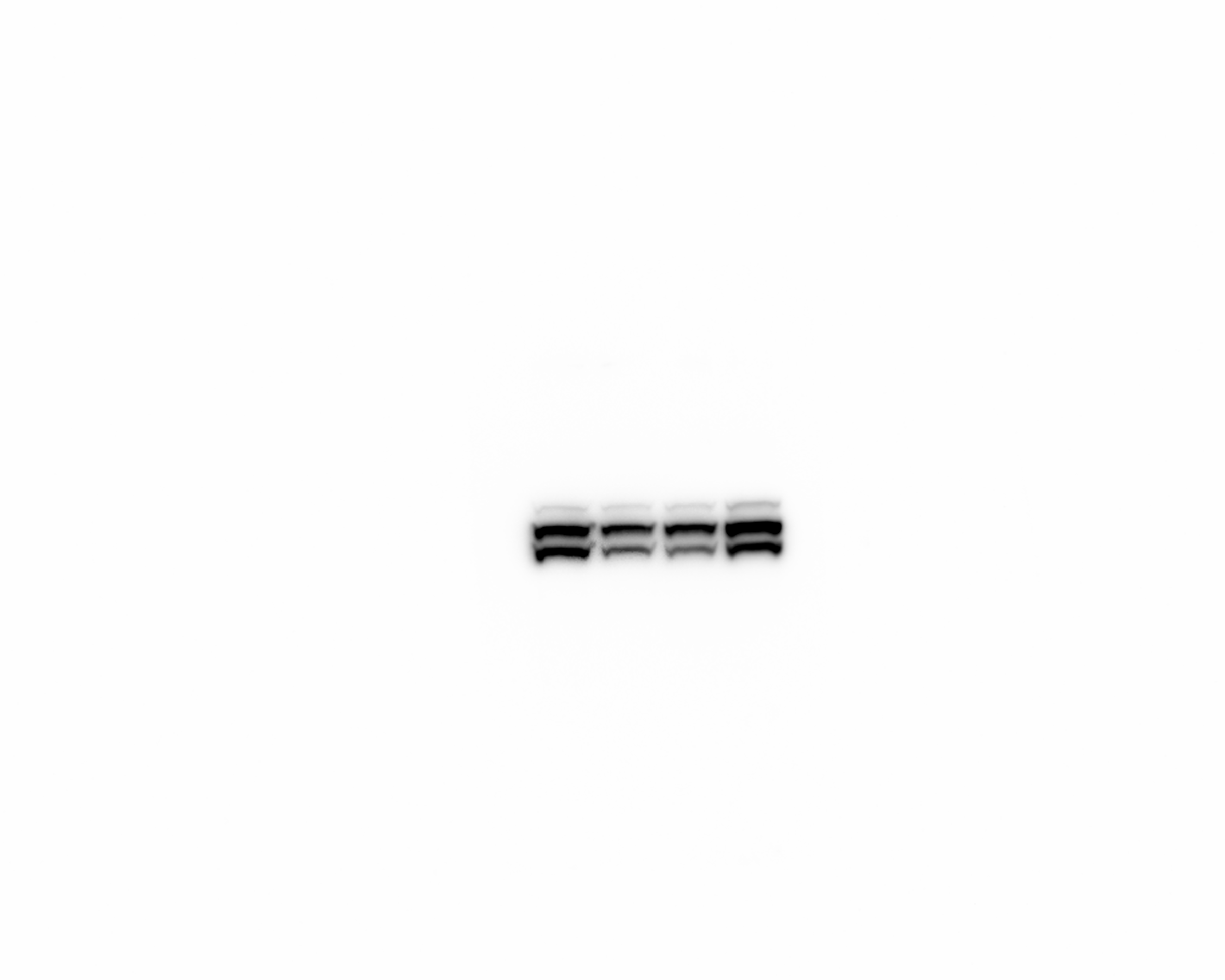

Supplement: Multimedia component 1 [file mmc1.zip › WB bands & raw densitometry/WB bands(24h)/3.p-Akt/1.P-Akt(Chemiluminescence).tif]

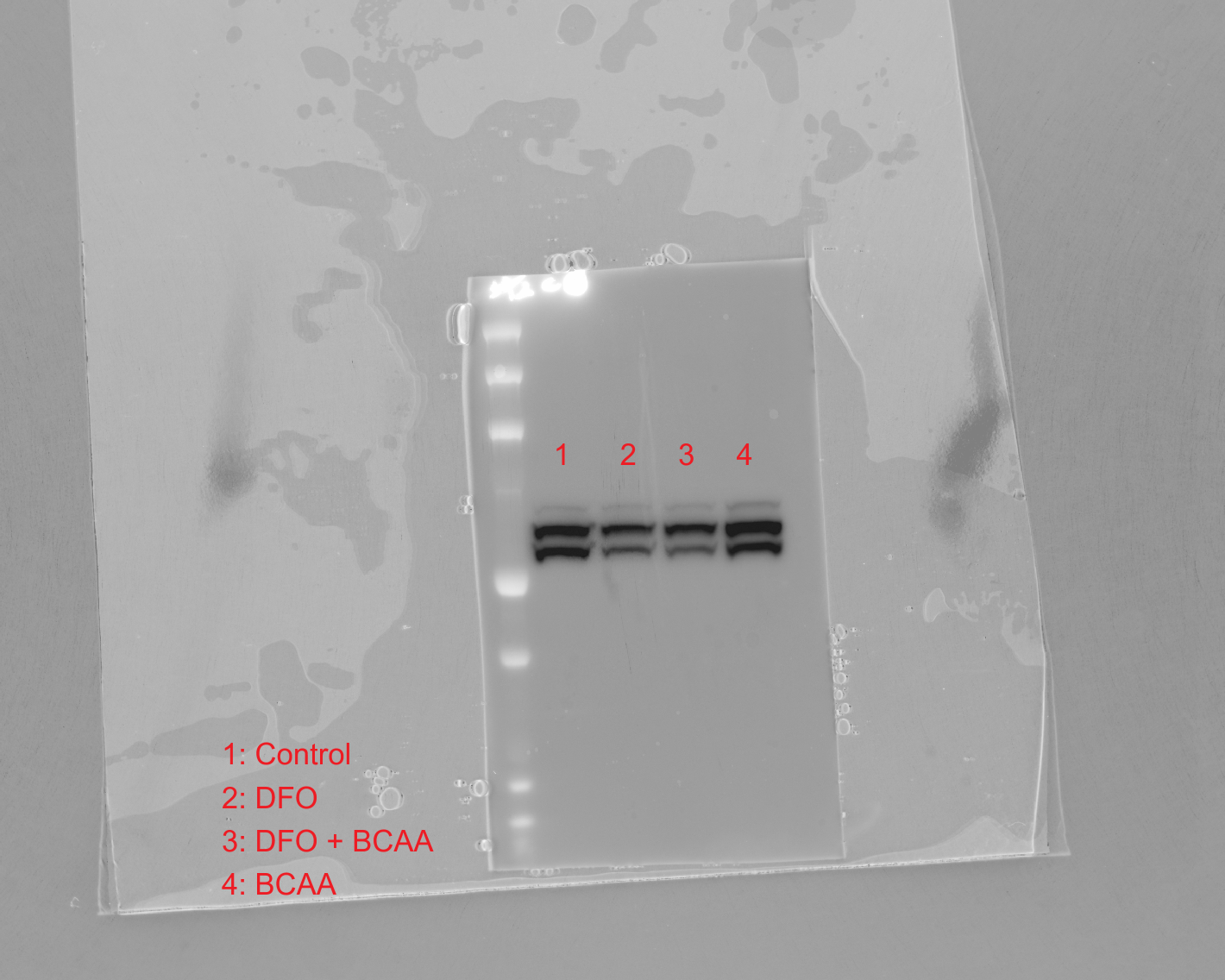

Supplement: Multimedia component 1 [file mmc1.zip › WB bands & raw densitometry/WB bands(24h)/3.p-Akt/1.P-Akt(Composite).tif]

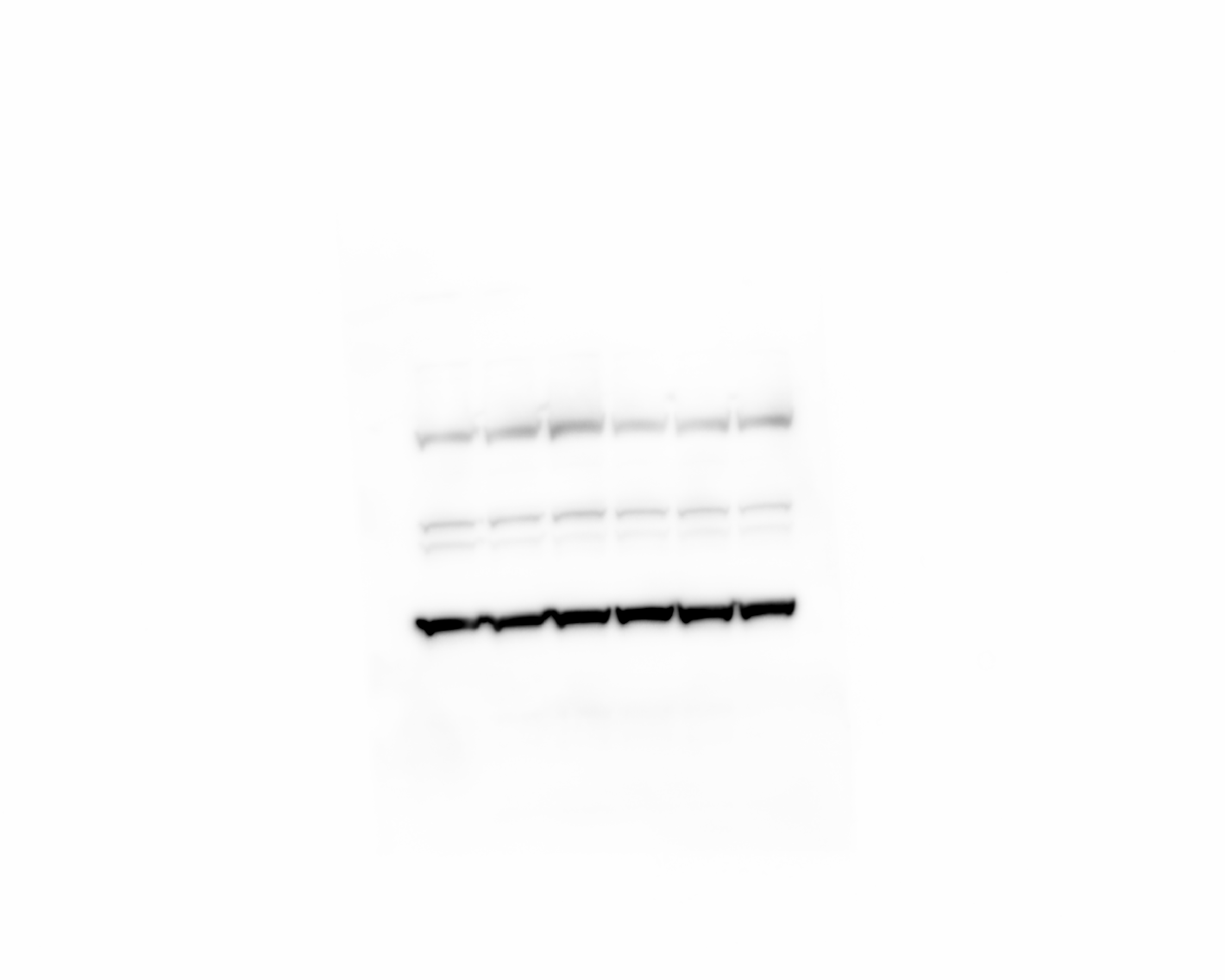

Supplement: Multimedia component 1 [file mmc1.zip › WB bands & raw densitometry/WB bands(24h)/3.p-Akt/2.B-actin(Chemiluminescence).tif]

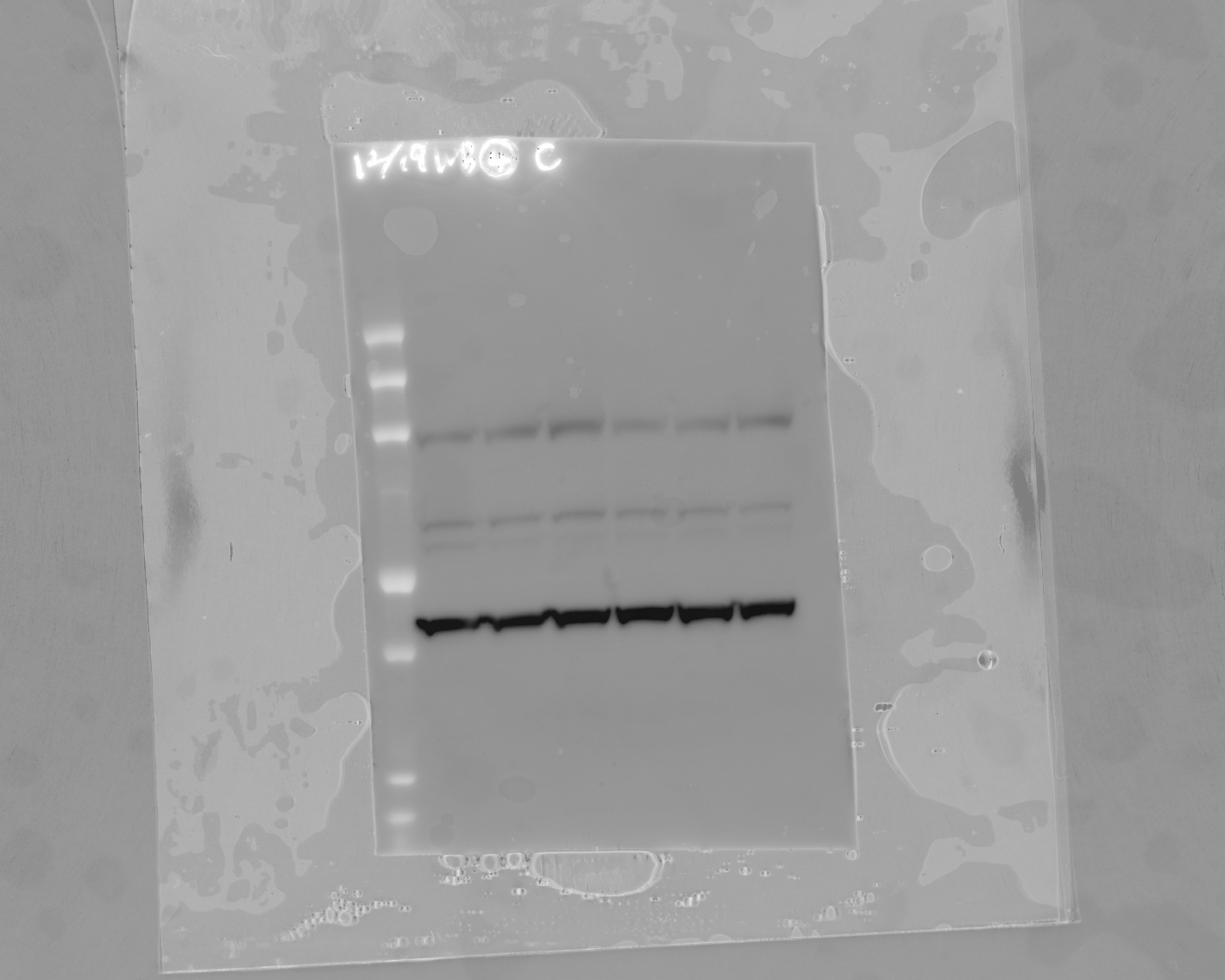

Supplement: Multimedia component 1 [file mmc1.zip › WB bands & raw densitometry/WB bands(24h)/3.p-Akt/2.B-actin(Composite).tif]

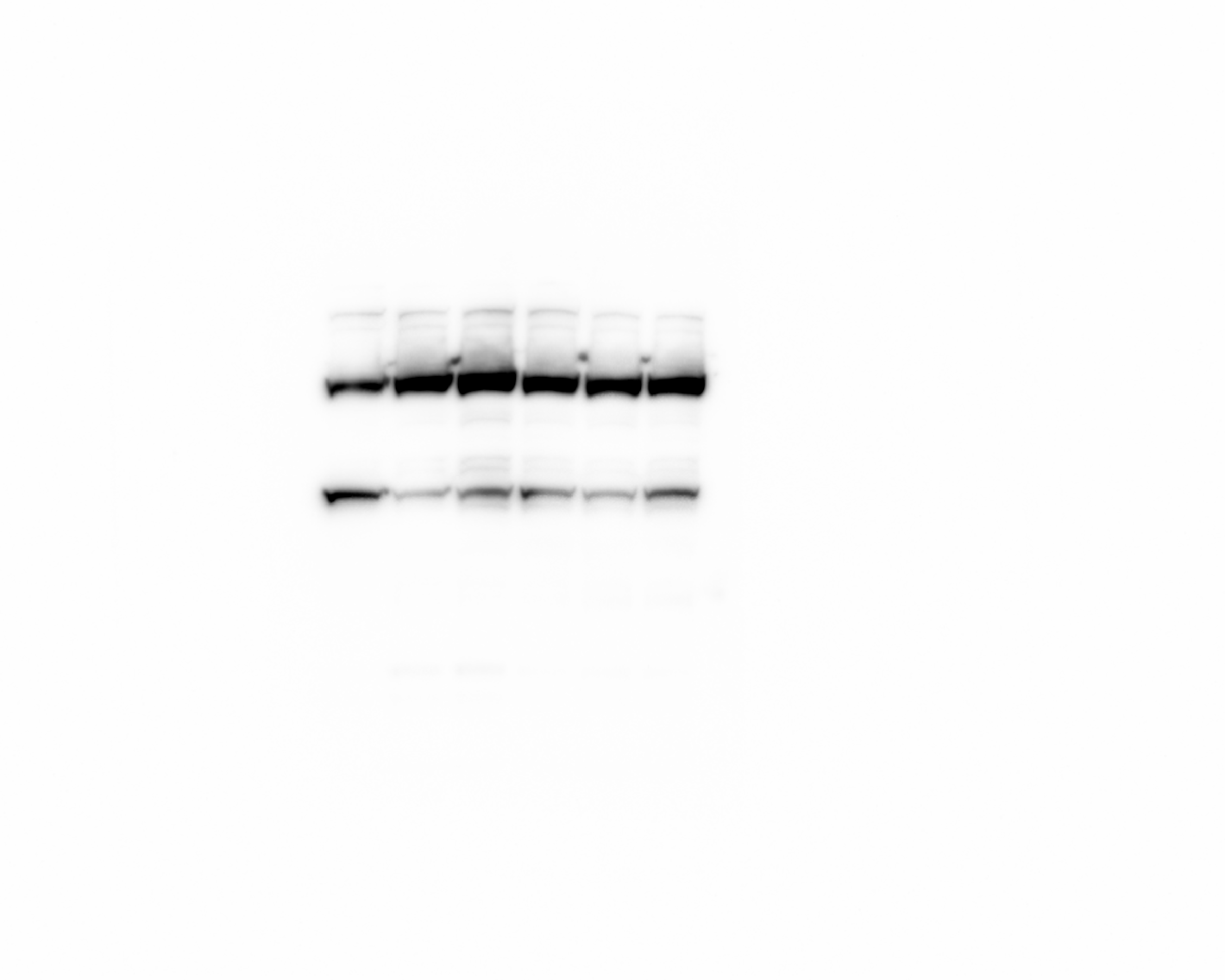

Supplement: Multimedia component 1 [file mmc1.zip › WB bands & raw densitometry/WB bands(24h)/3.p-Akt/2.P-Akt(Chemiluminescence).tif]

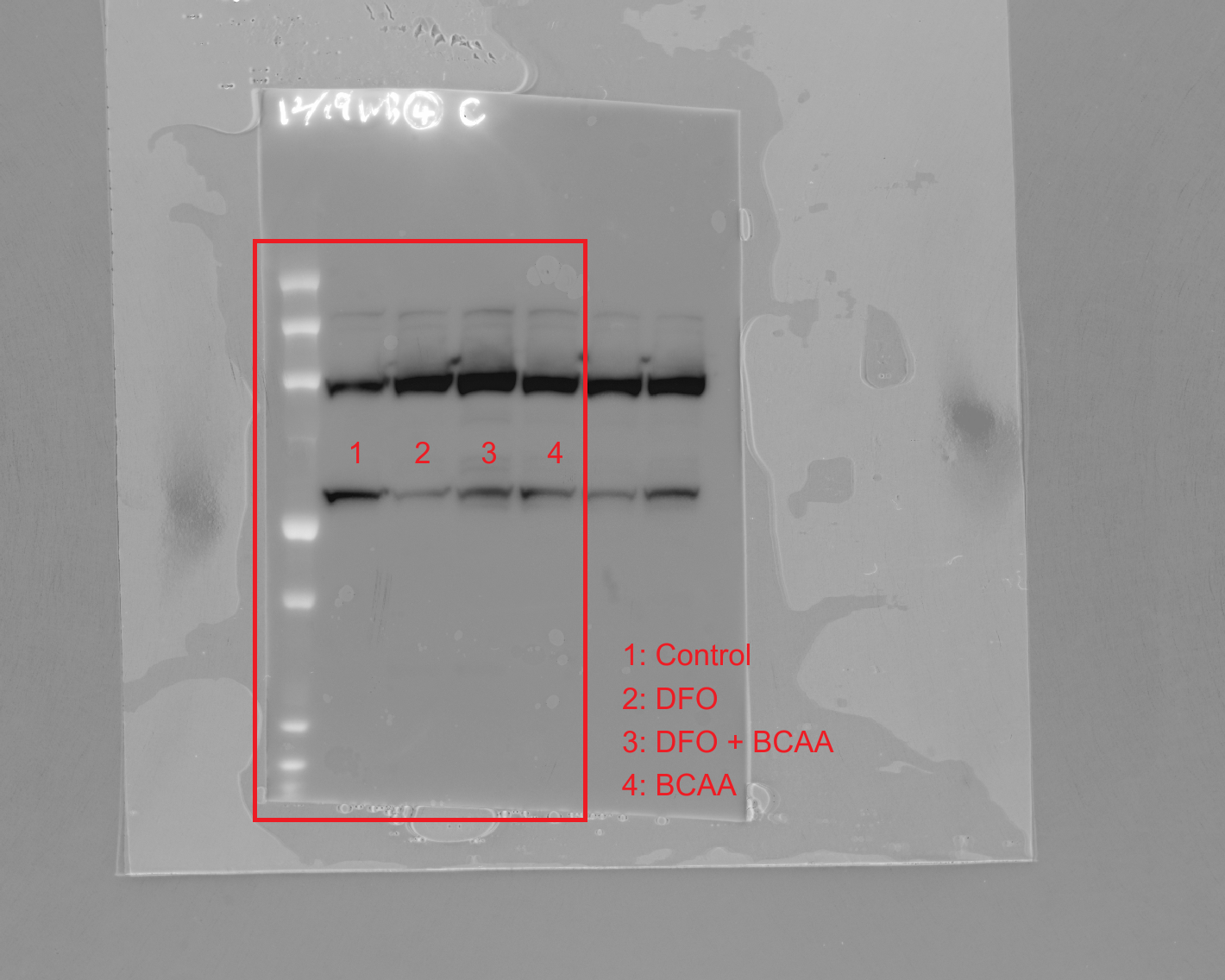

Supplement: Multimedia component 1 [file mmc1.zip › WB bands & raw densitometry/WB bands(24h)/3.p-Akt/2.P-Akt(Composite).tif]

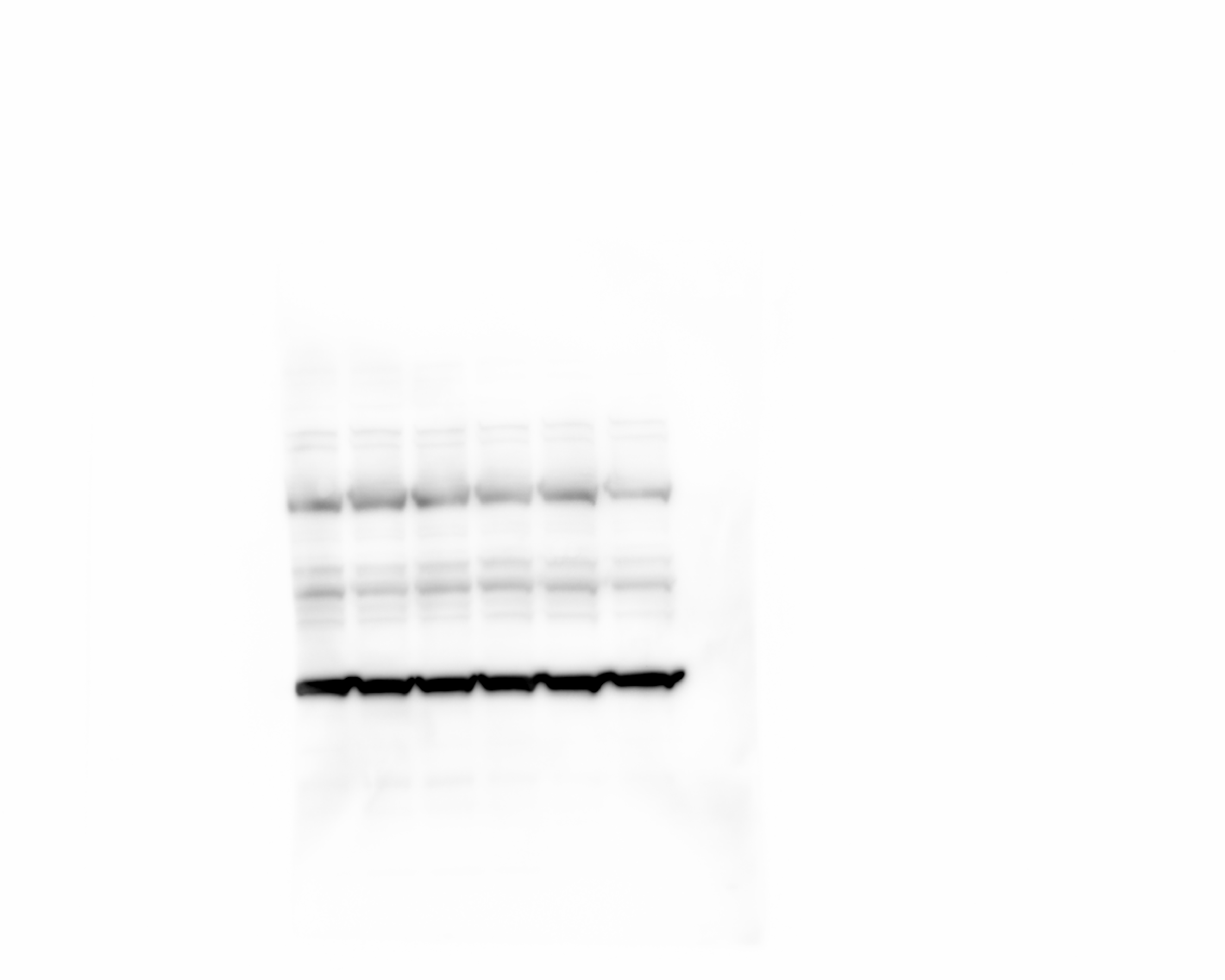

Supplement: Multimedia component 1 [file mmc1.zip › WB bands & raw densitometry/WB bands(24h)/3.p-Akt/3.B-actin(Chemiluminescence).tif]

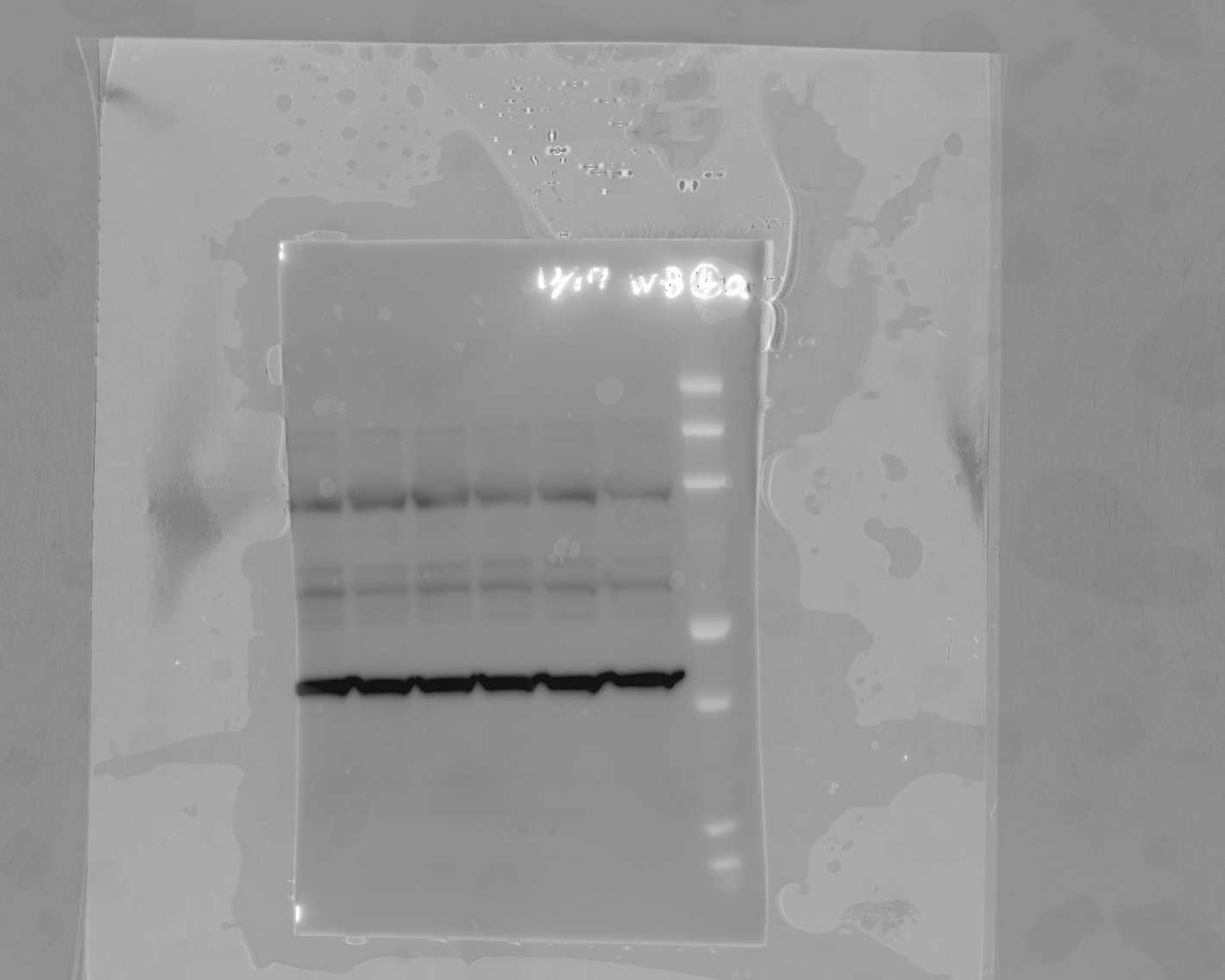

Supplement: Multimedia component 1 [file mmc1.zip › WB bands & raw densitometry/WB bands(24h)/3.p-Akt/3.B-actin(Composite).tif]

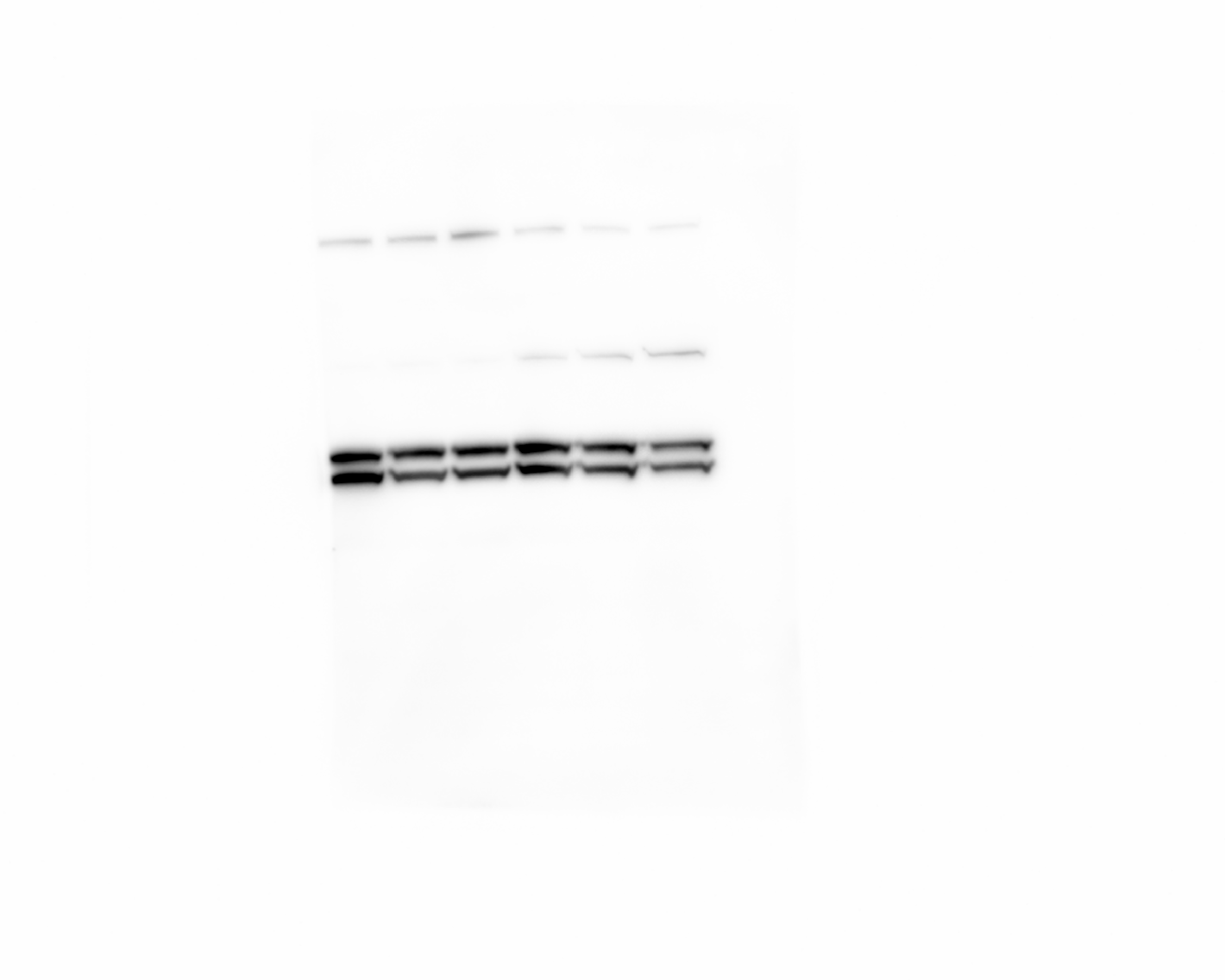

Supplement: Multimedia component 1 [file mmc1.zip › WB bands & raw densitometry/WB bands(24h)/3.p-Akt/3.P-Akt(Chemiluminescence).tif]

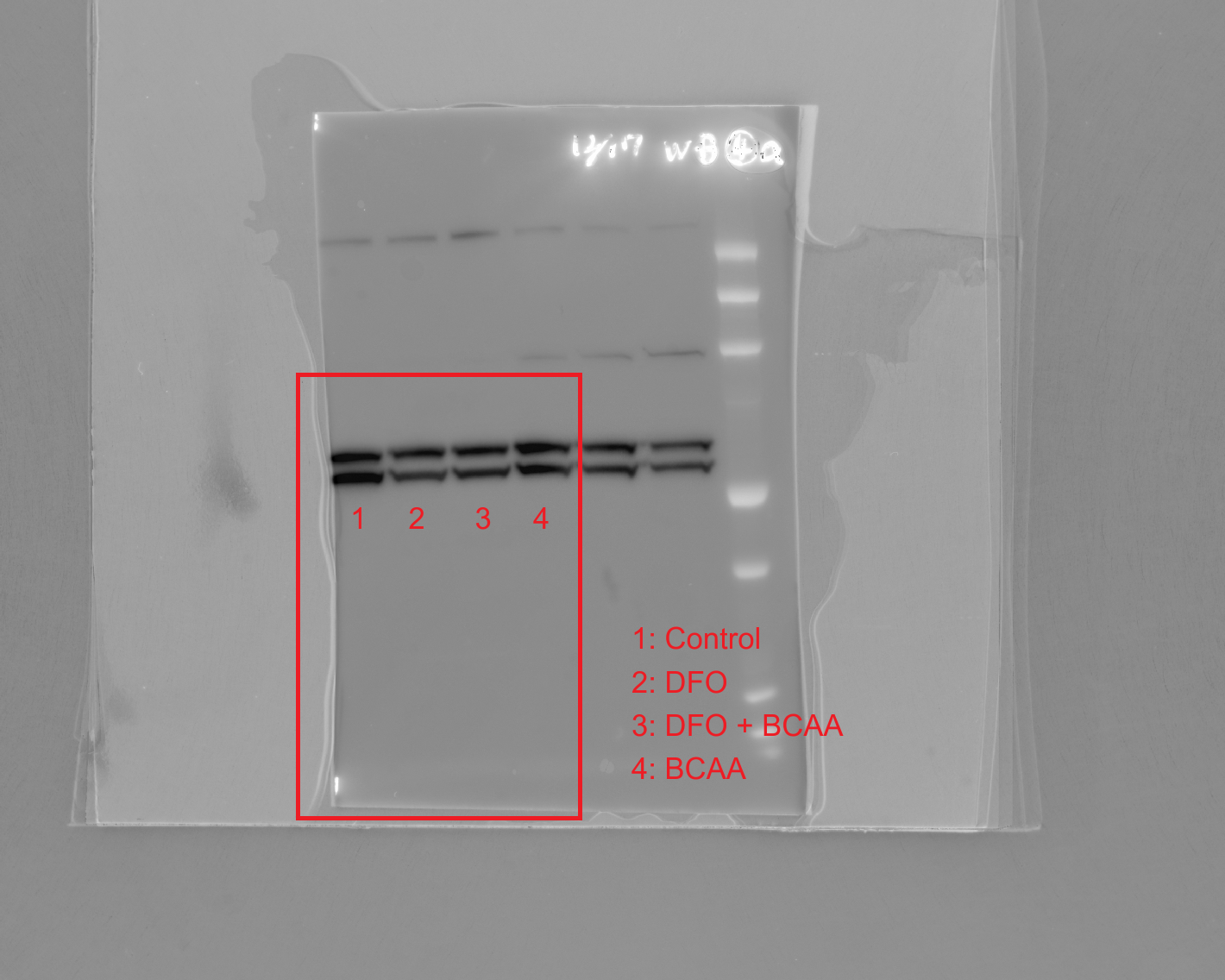

Supplement: Multimedia component 1 [file mmc1.zip › WB bands & raw densitometry/WB bands(24h)/3.p-Akt/3.P-Akt(Composite).tif]

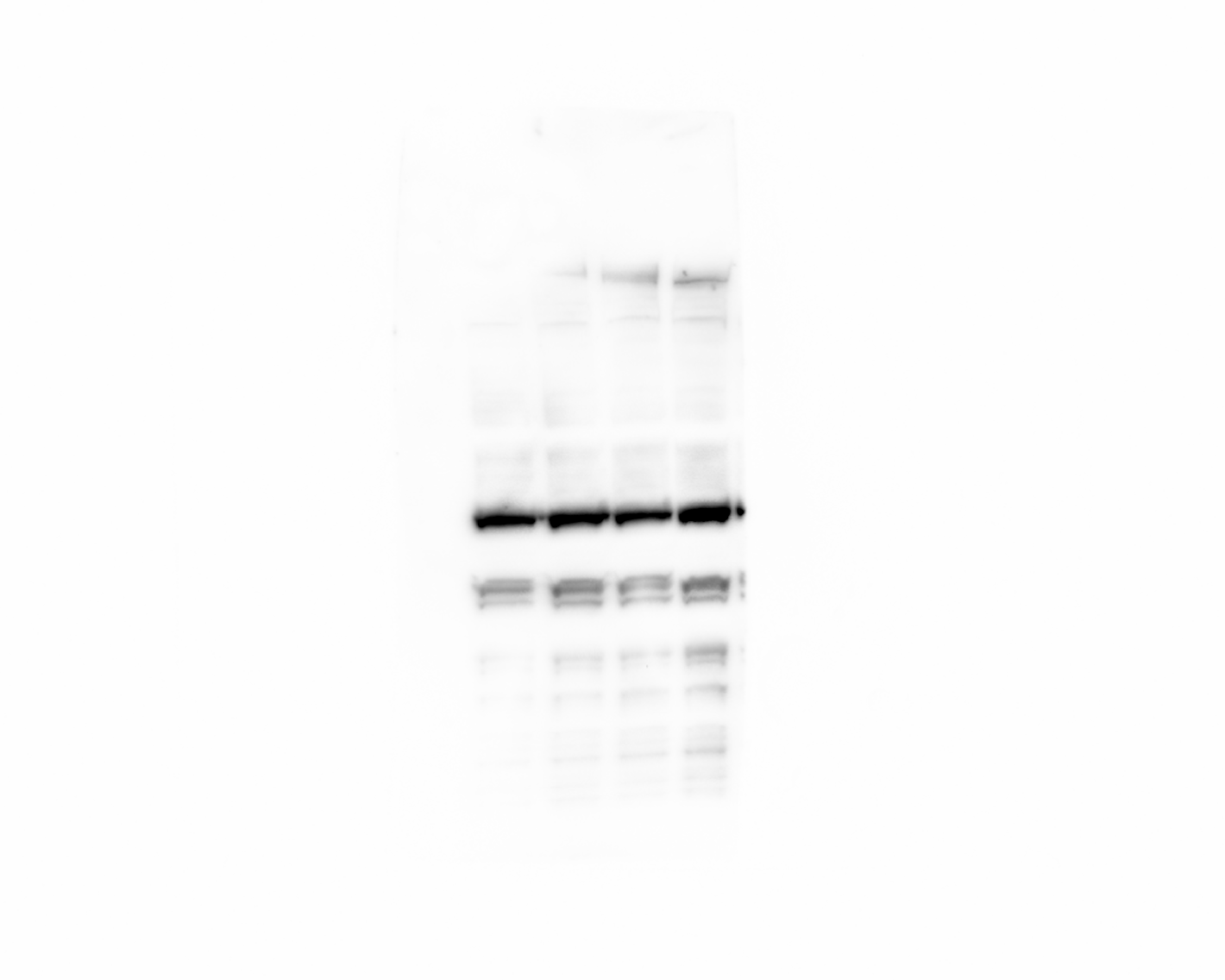

Supplement: Multimedia component 1 [file mmc1.zip › WB bands & raw densitometry/WB bands(24h)/4.AMPK/1.AMPK(Chemiluminescence).tif]

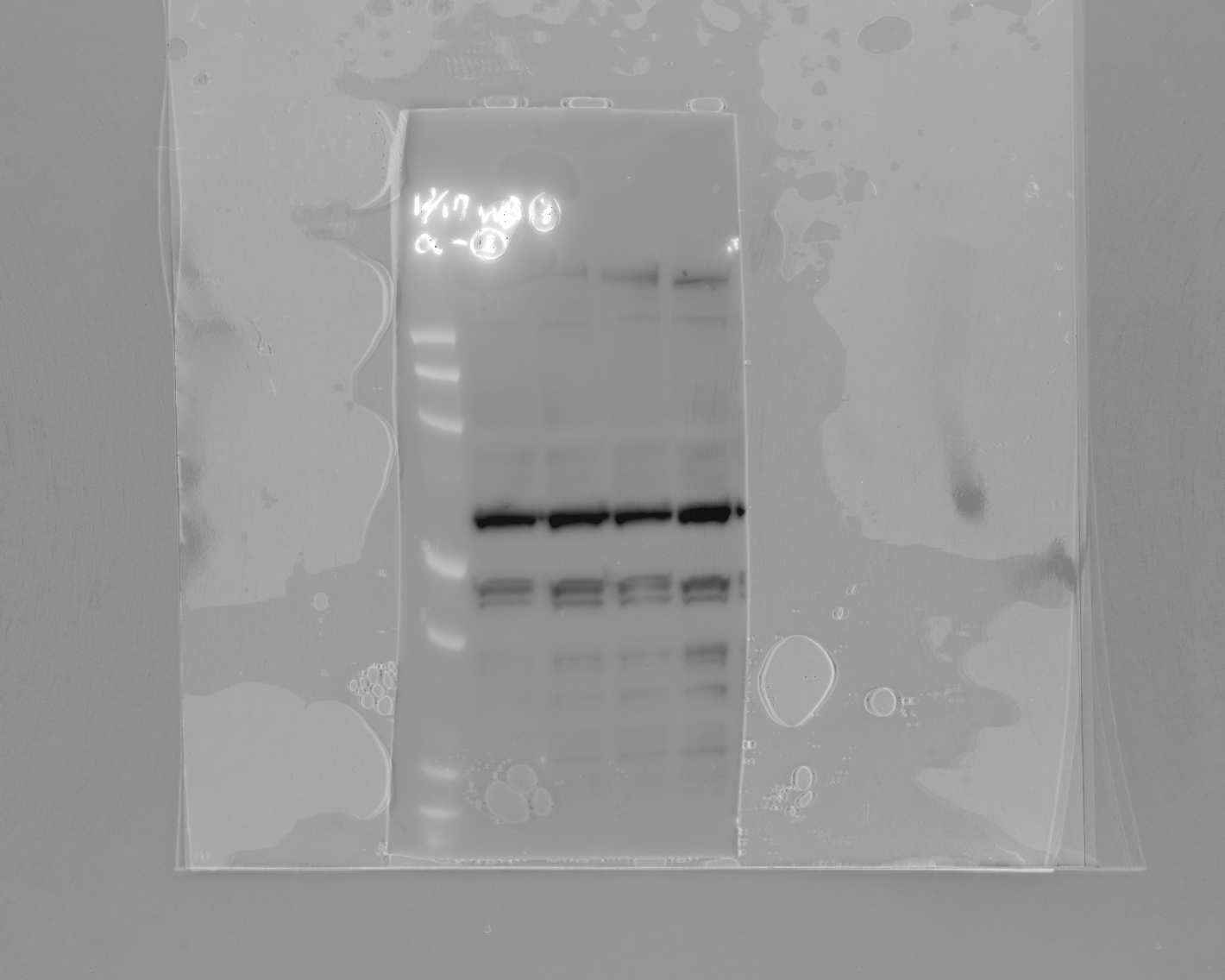

Supplement: Multimedia component 1 [file mmc1.zip › WB bands & raw densitometry/WB bands(24h)/4.AMPK/1.AMPK(Composite).tif]

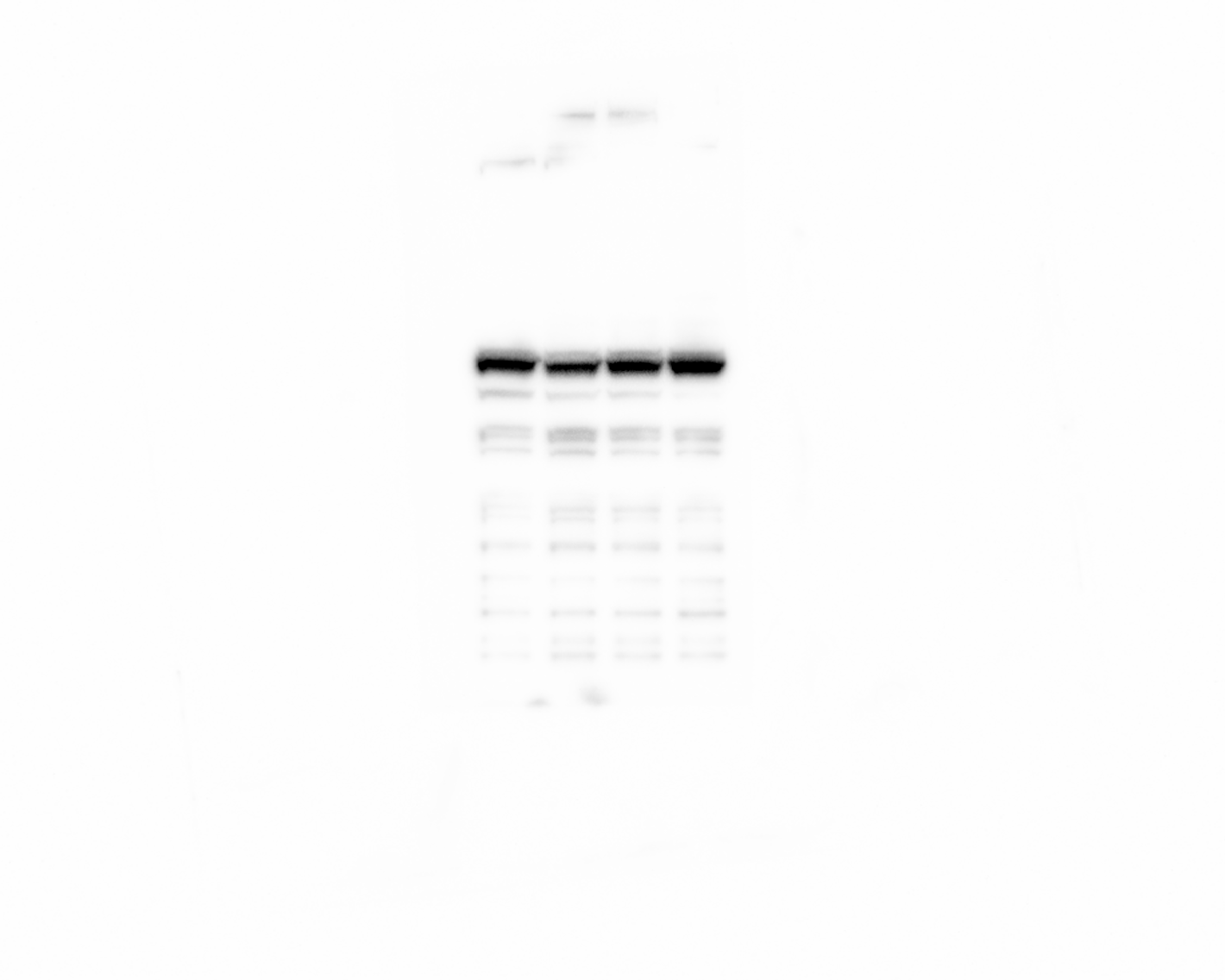

Supplement: Multimedia component 1 [file mmc1.zip › WB bands & raw densitometry/WB bands(24h)/4.AMPK/2.AMPK(Chemiluminescence).tif]

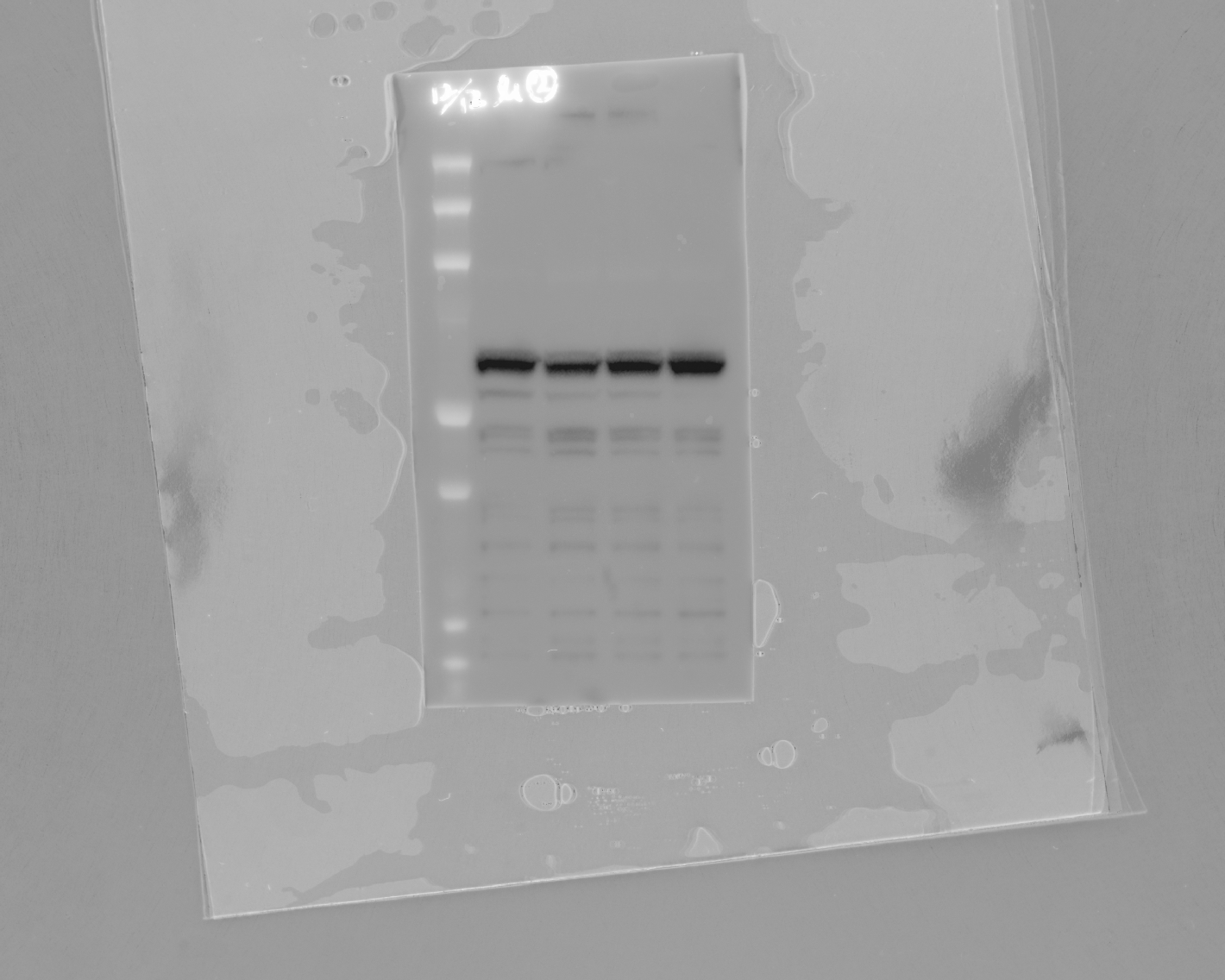

Supplement: Multimedia component 1 [file mmc1.zip › WB bands & raw densitometry/WB bands(24h)/4.AMPK/2.AMPK(Composite).tif]

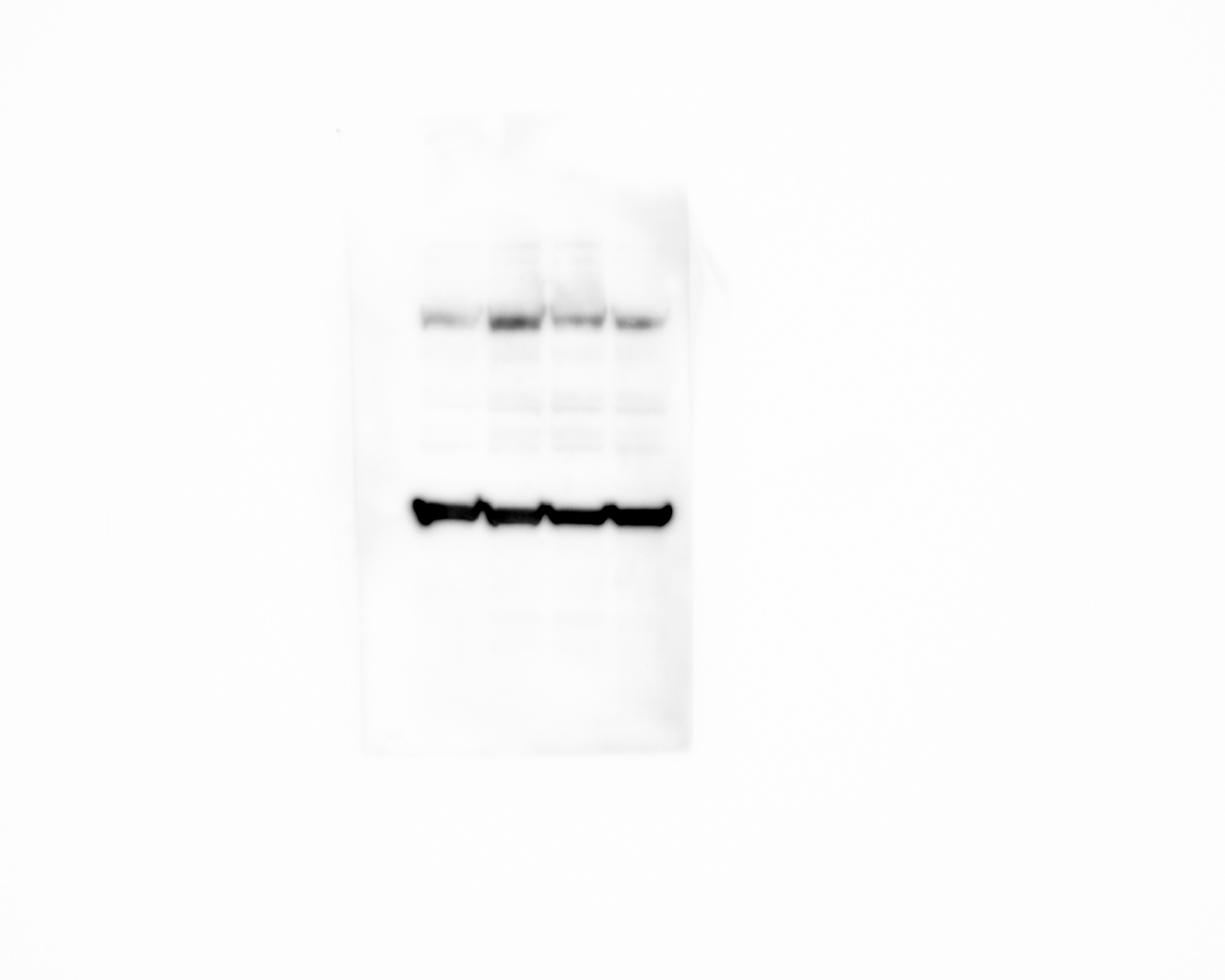

Supplement: Multimedia component 1 [file mmc1.zip › WB bands & raw densitometry/WB bands(24h)/4.AMPK/2.B-actin(Chemiluminescence).tif]

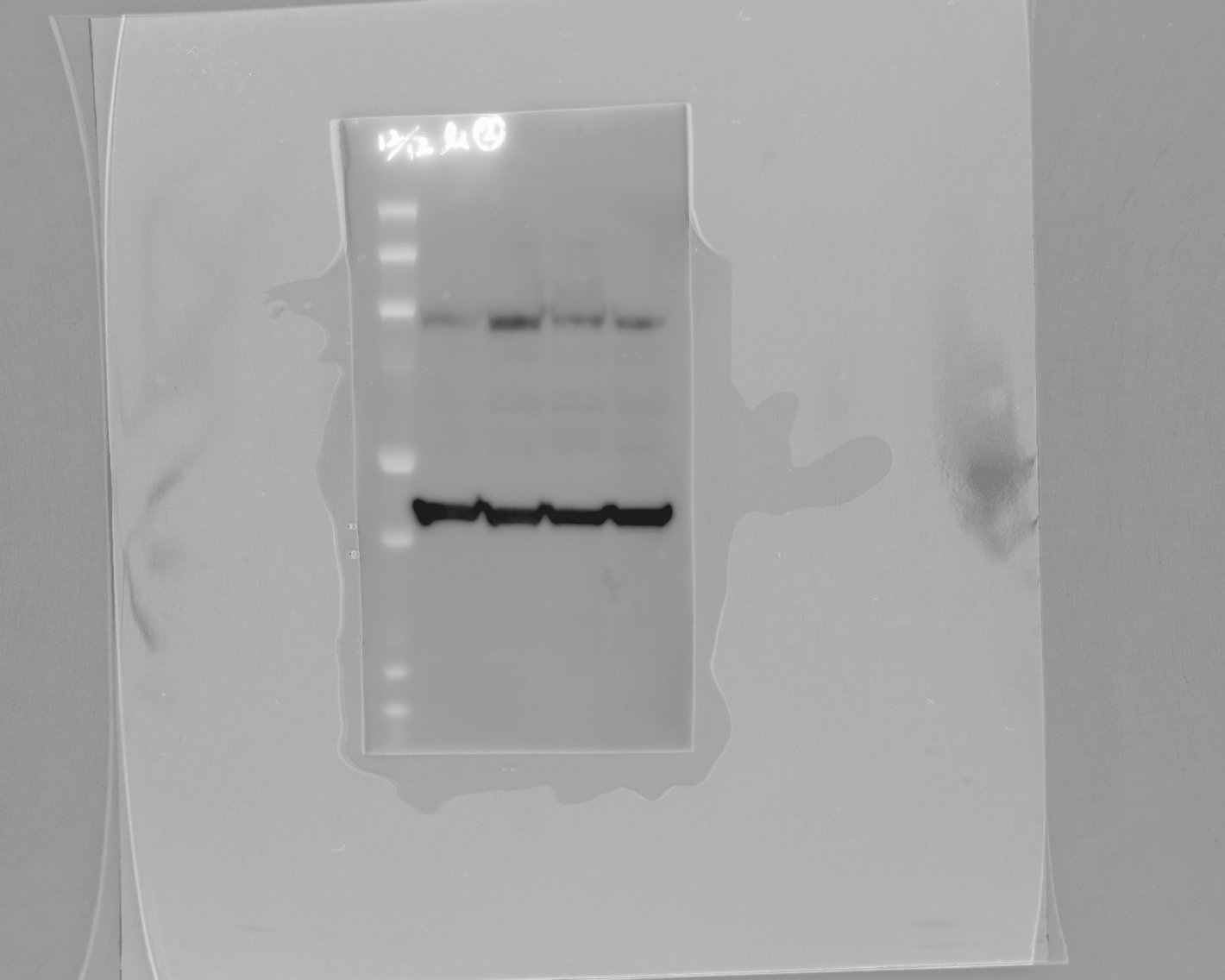

Supplement: Multimedia component 1 [file mmc1.zip › WB bands & raw densitometry/WB bands(24h)/4.AMPK/2.B-actin(Composite).tif]

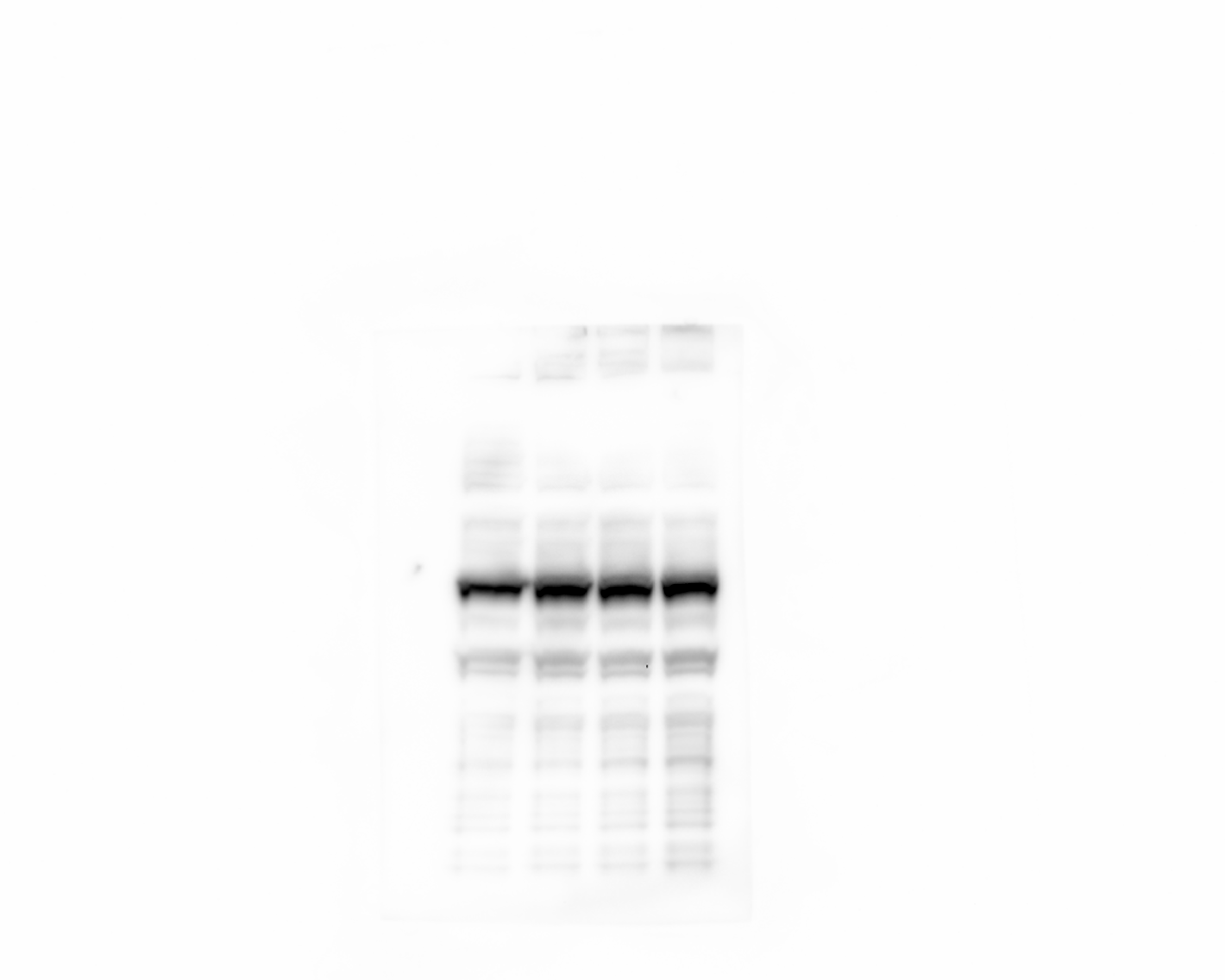

Supplement: Multimedia component 1 [file mmc1.zip › WB bands & raw densitometry/WB bands(24h)/4.AMPK/3.AMPK(Chemiluminescence).tif]

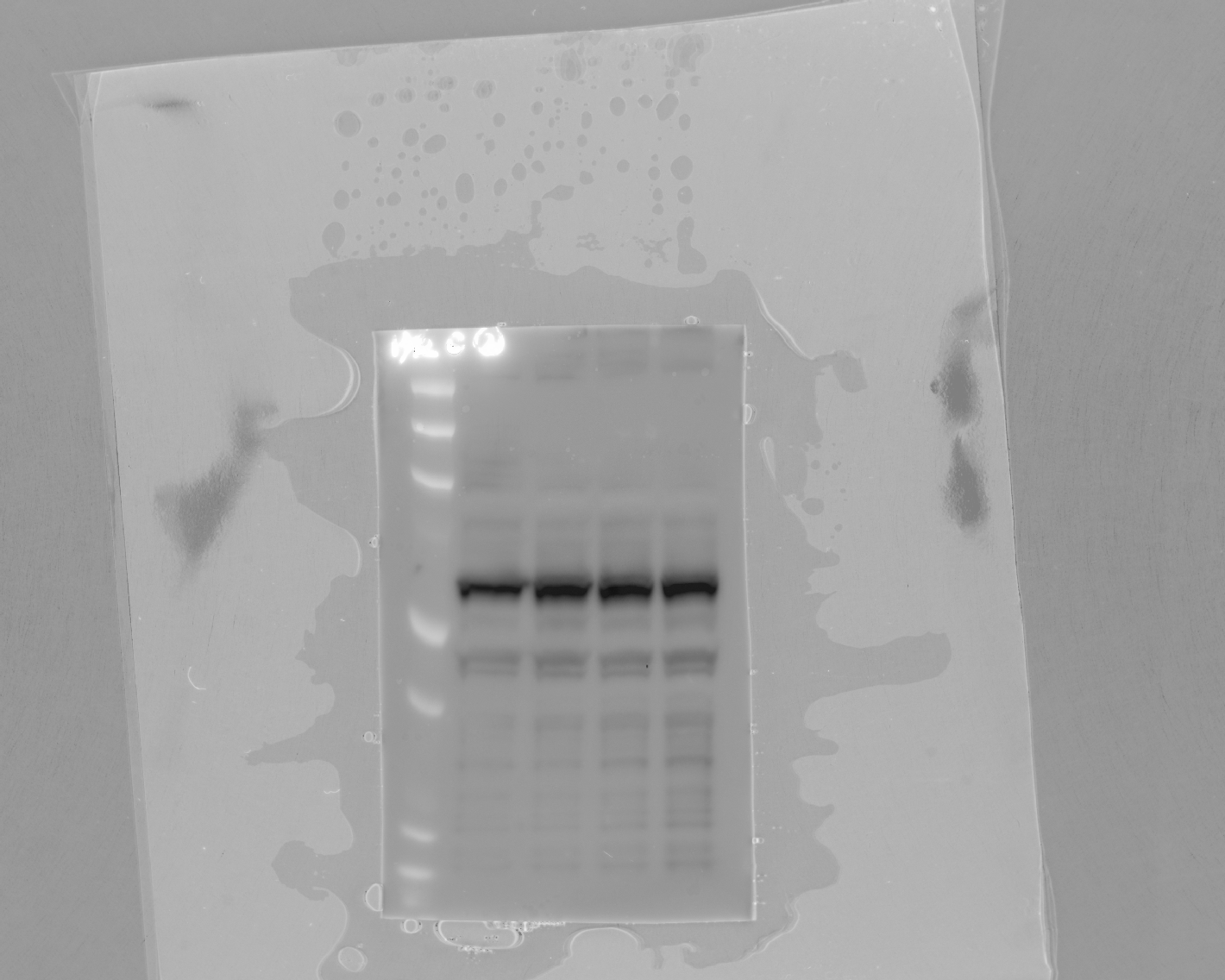

Supplement: Multimedia component 1 [file mmc1.zip › WB bands & raw densitometry/WB bands(24h)/4.AMPK/3.AMPK(Composite).tif]

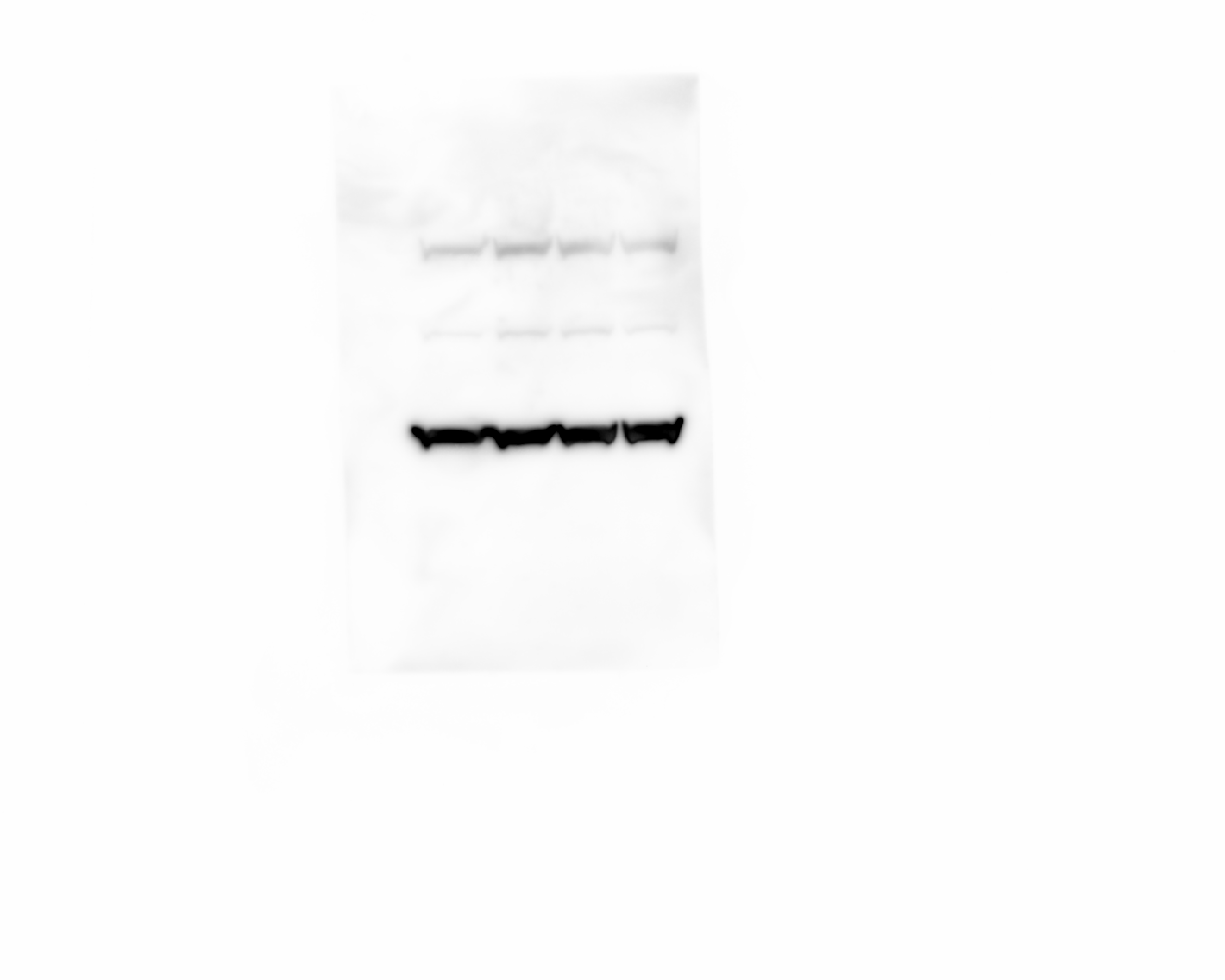

Supplement: Multimedia component 1 [file mmc1.zip › WB bands & raw densitometry/WB bands(24h)/4.AMPK/3.B-actin(Chemiluminescence).tif]

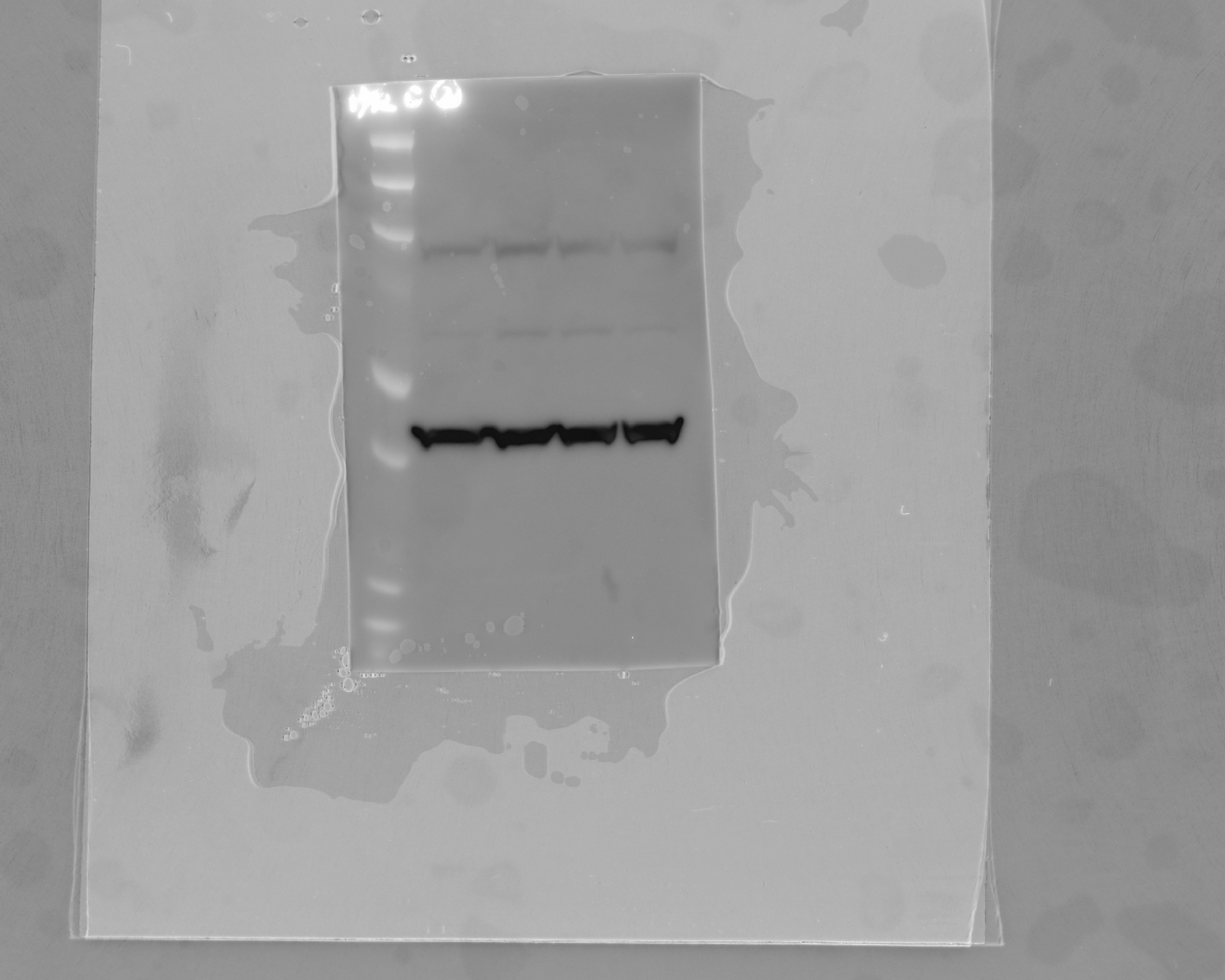

Supplement: Multimedia component 1 [file mmc1.zip › WB bands & raw densitometry/WB bands(24h)/4.AMPK/3.B-actin(Composite).tif]

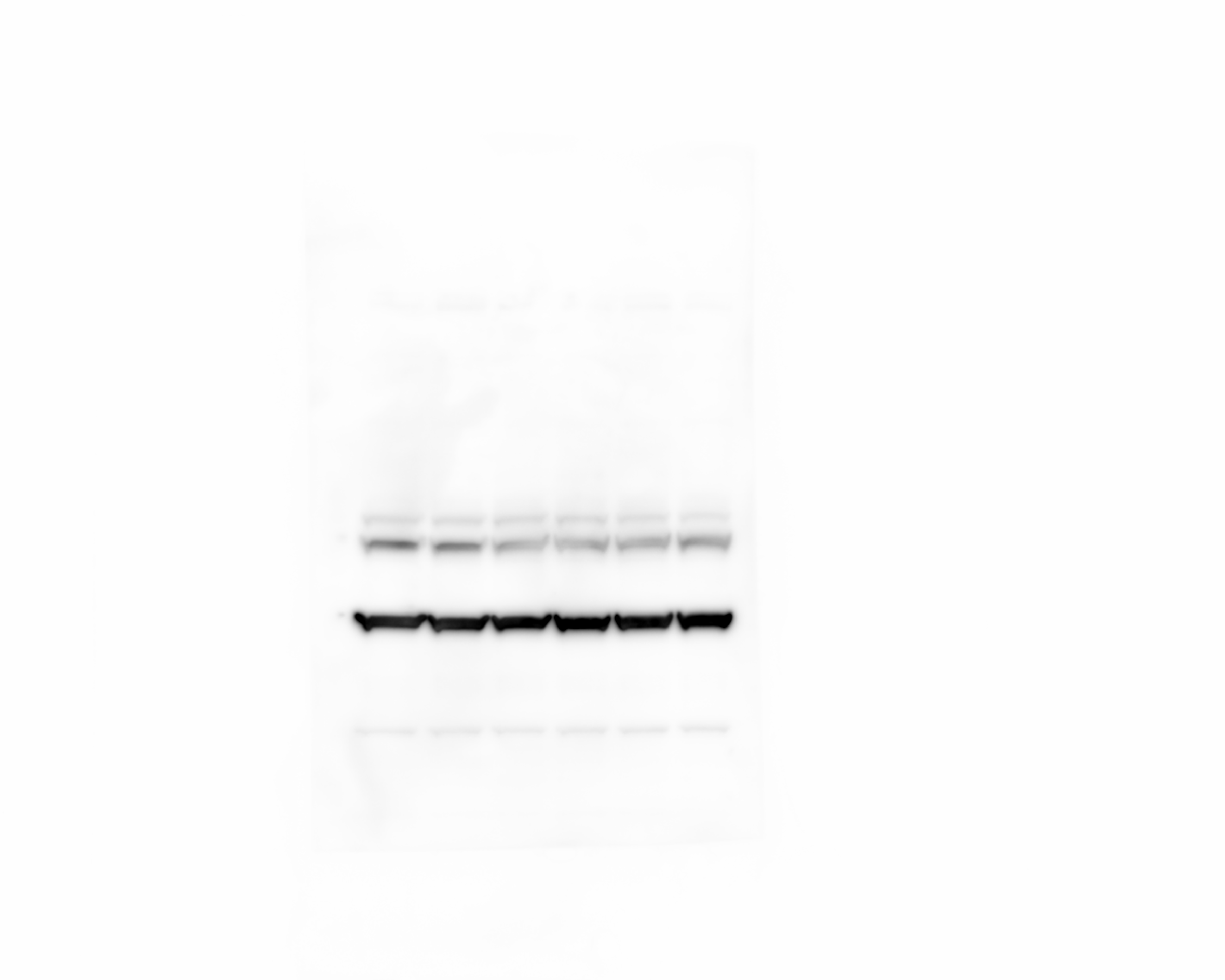

Supplement: Multimedia component 1 [file mmc1.zip › WB bands & raw densitometry/WB bands(24h)/5.p-AMPK/1.B-actin(Chemiluminescence).tif]

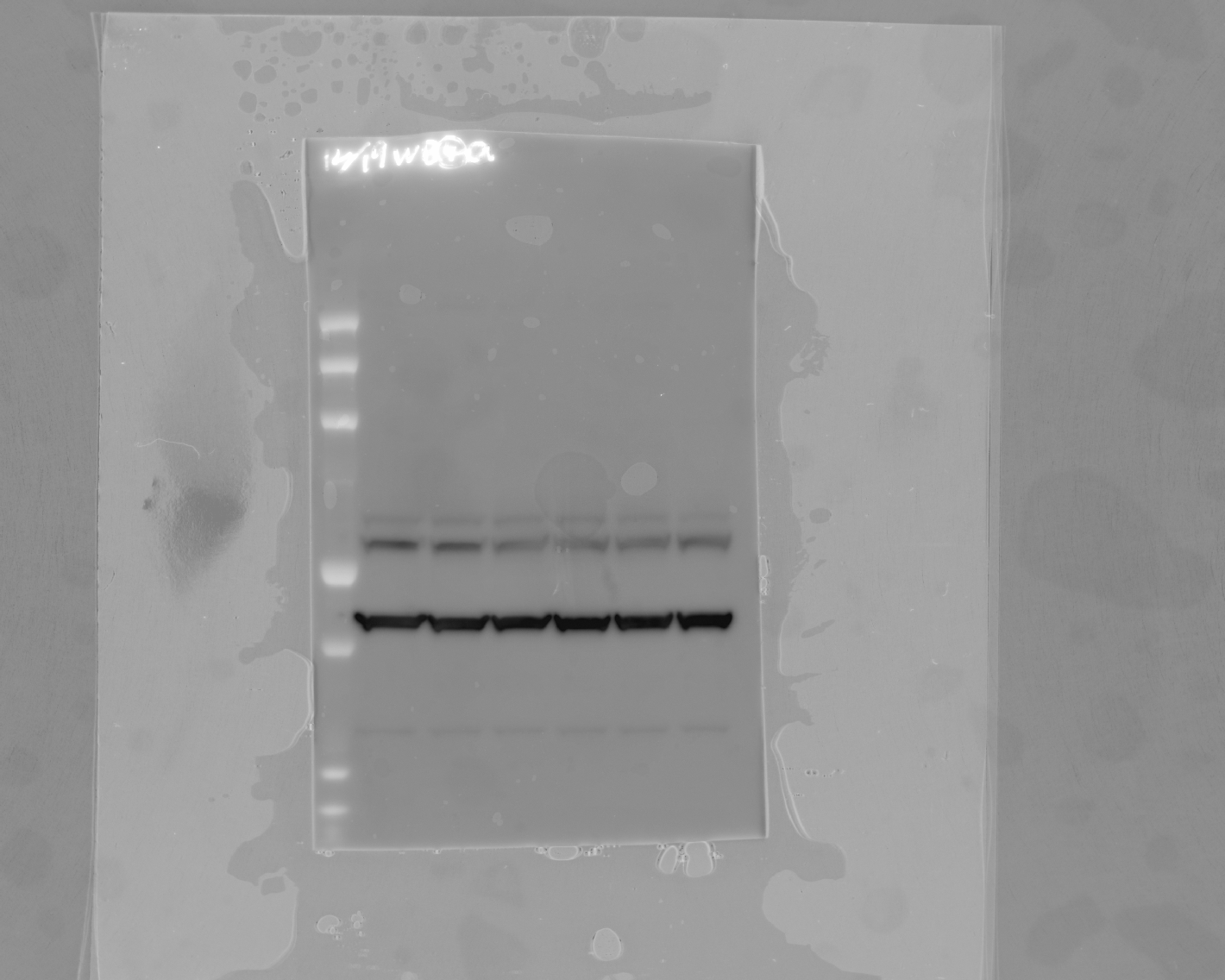

Supplement: Multimedia component 1 [file mmc1.zip › WB bands & raw densitometry/WB bands(24h)/5.p-AMPK/1.B-actin(Composite).tif]

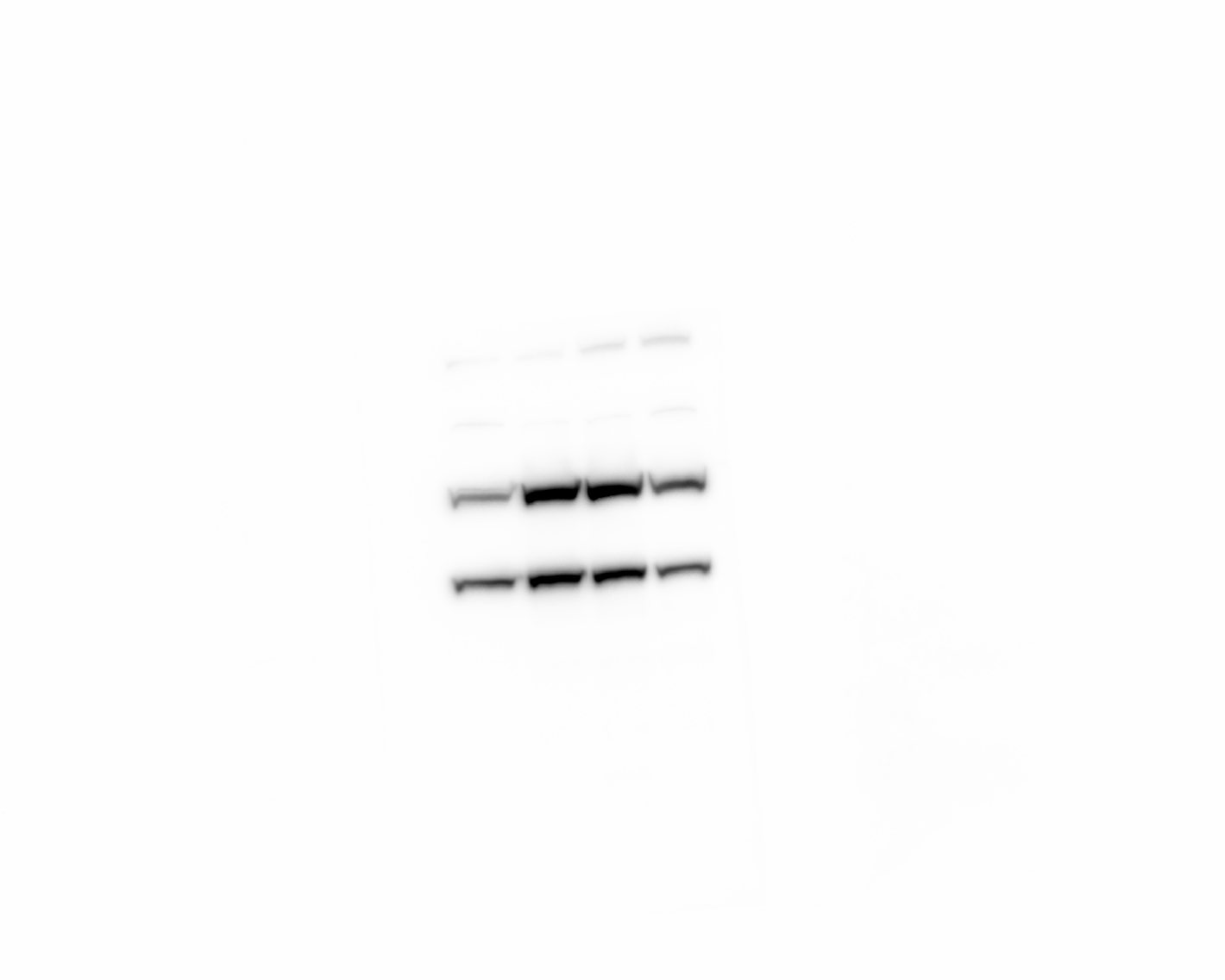

Supplement: Multimedia component 1 [file mmc1.zip › WB bands & raw densitometry/WB bands(24h)/5.p-AMPK/1.P-AMPK(Chemiluminescence).tif]

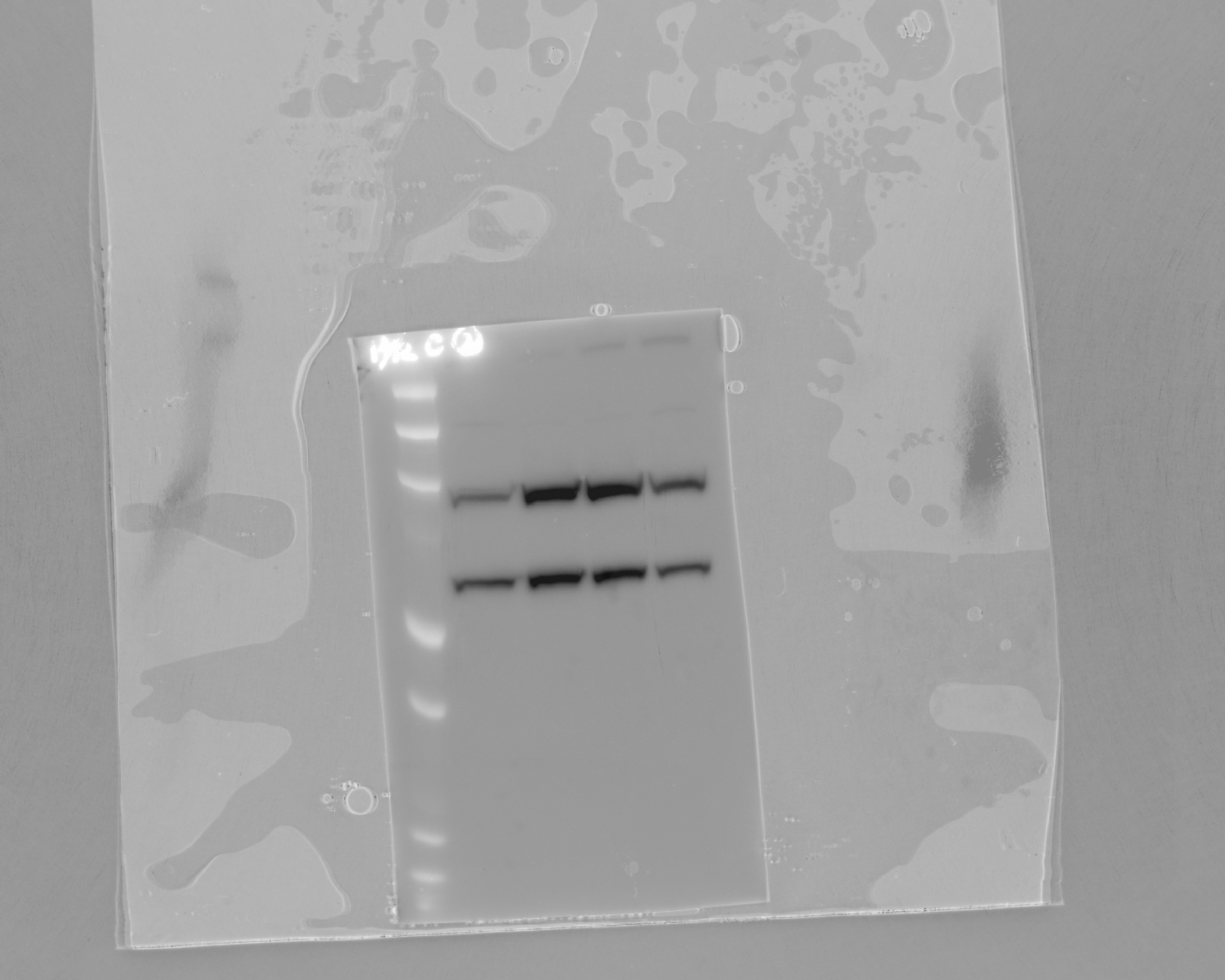

Supplement: Multimedia component 1 [file mmc1.zip › WB bands & raw densitometry/WB bands(24h)/5.p-AMPK/1.P-AMPK(Composite).tif]

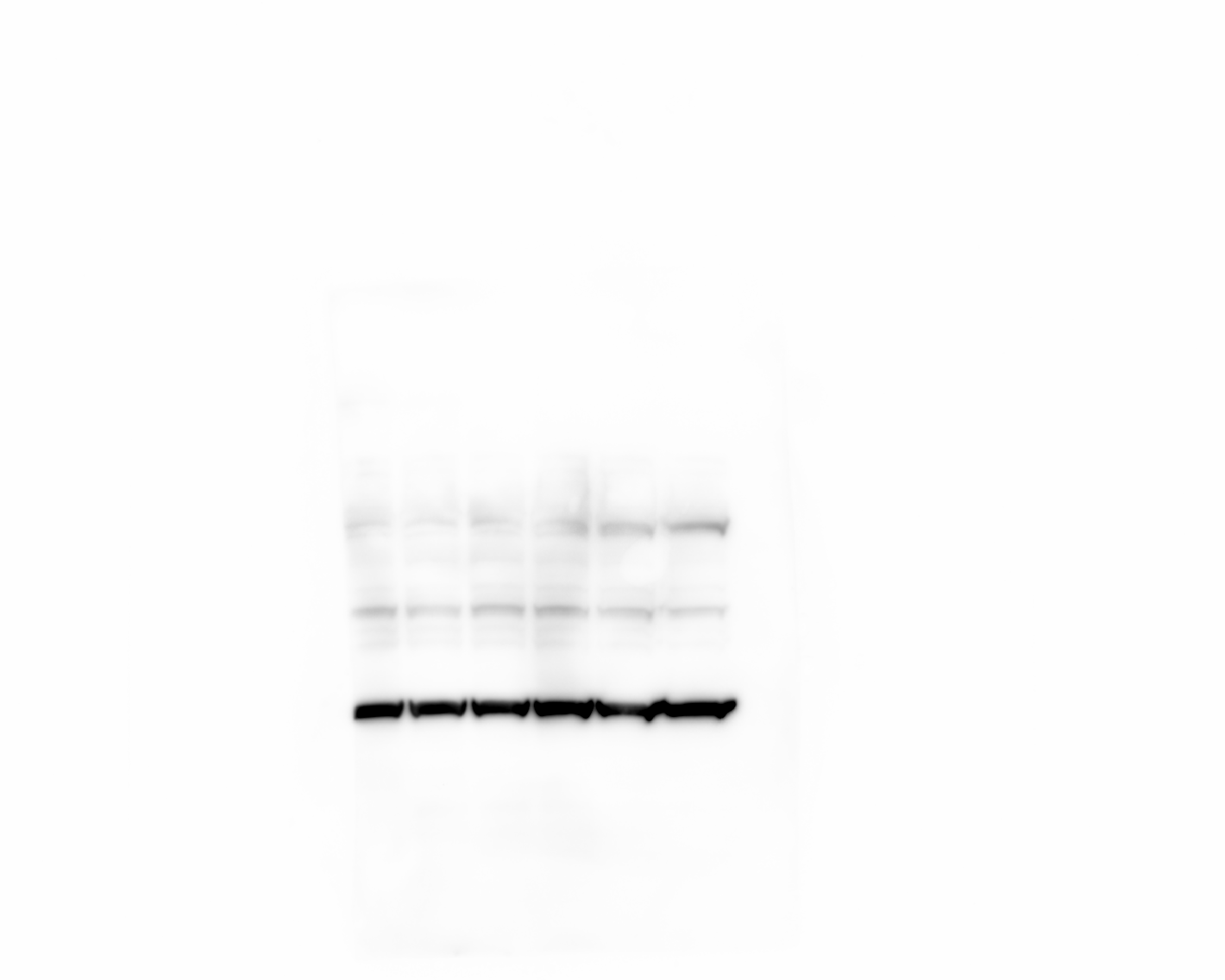

Supplement: Multimedia component 1 [file mmc1.zip › WB bands & raw densitometry/WB bands(24h)/5.p-AMPK/2.B-actin(Chemiluminescence).tif]

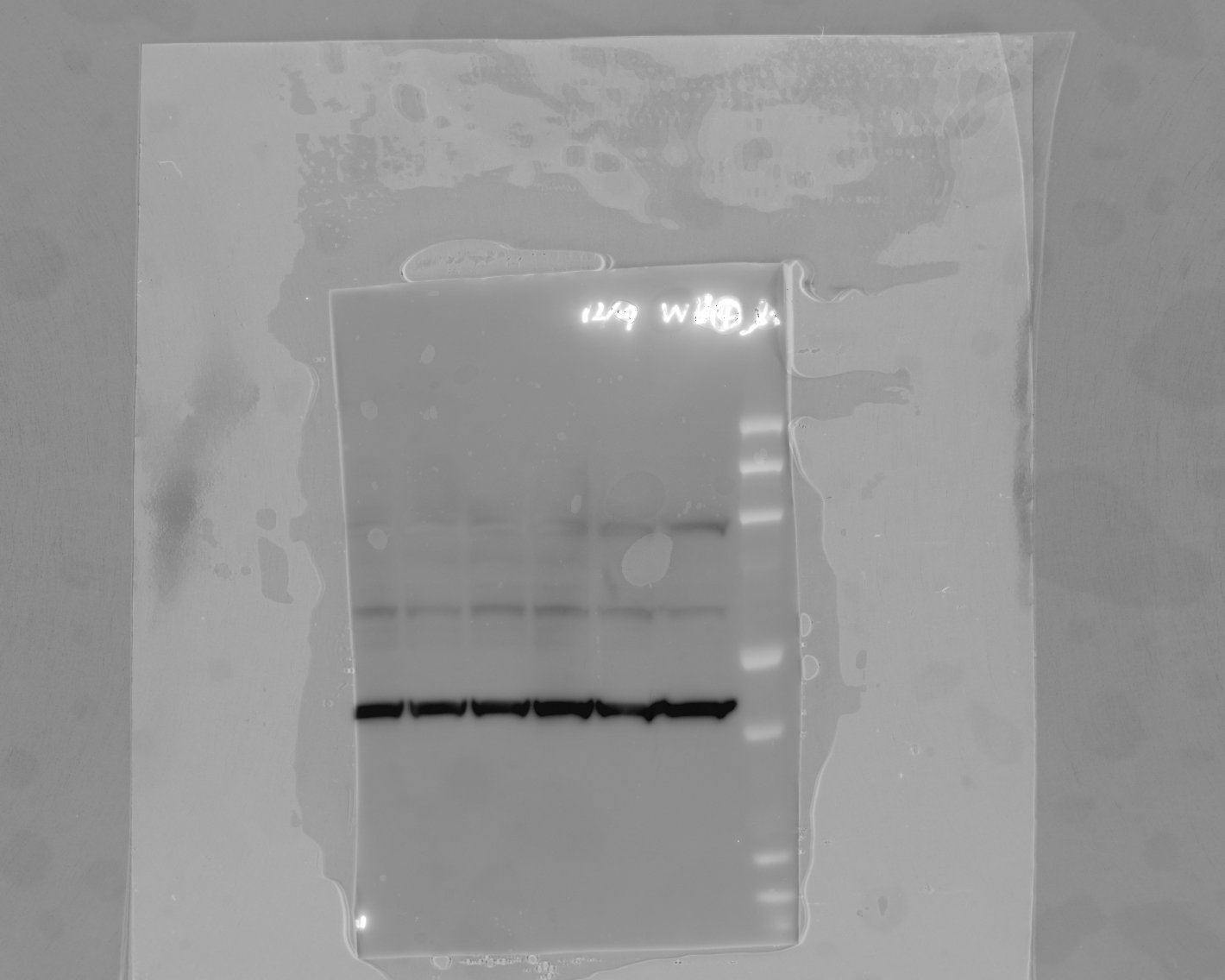

Supplement: Multimedia component 1 [file mmc1.zip › WB bands & raw densitometry/WB bands(24h)/5.p-AMPK/2.B-actin(Composite).tif]

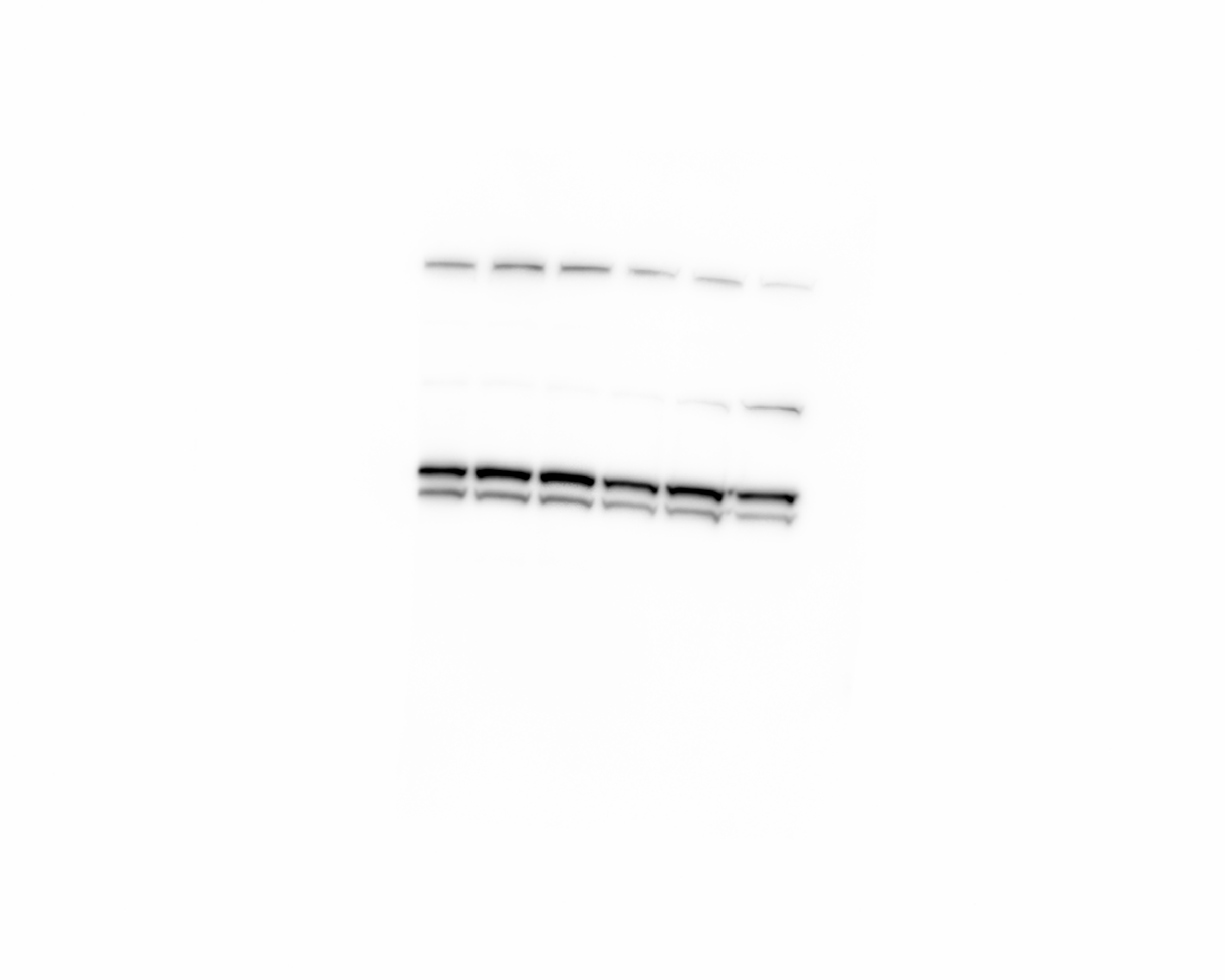

Supplement: Multimedia component 1 [file mmc1.zip › WB bands & raw densitometry/WB bands(24h)/5.p-AMPK/2.P-AMPK(Chemiluminescence).tif]

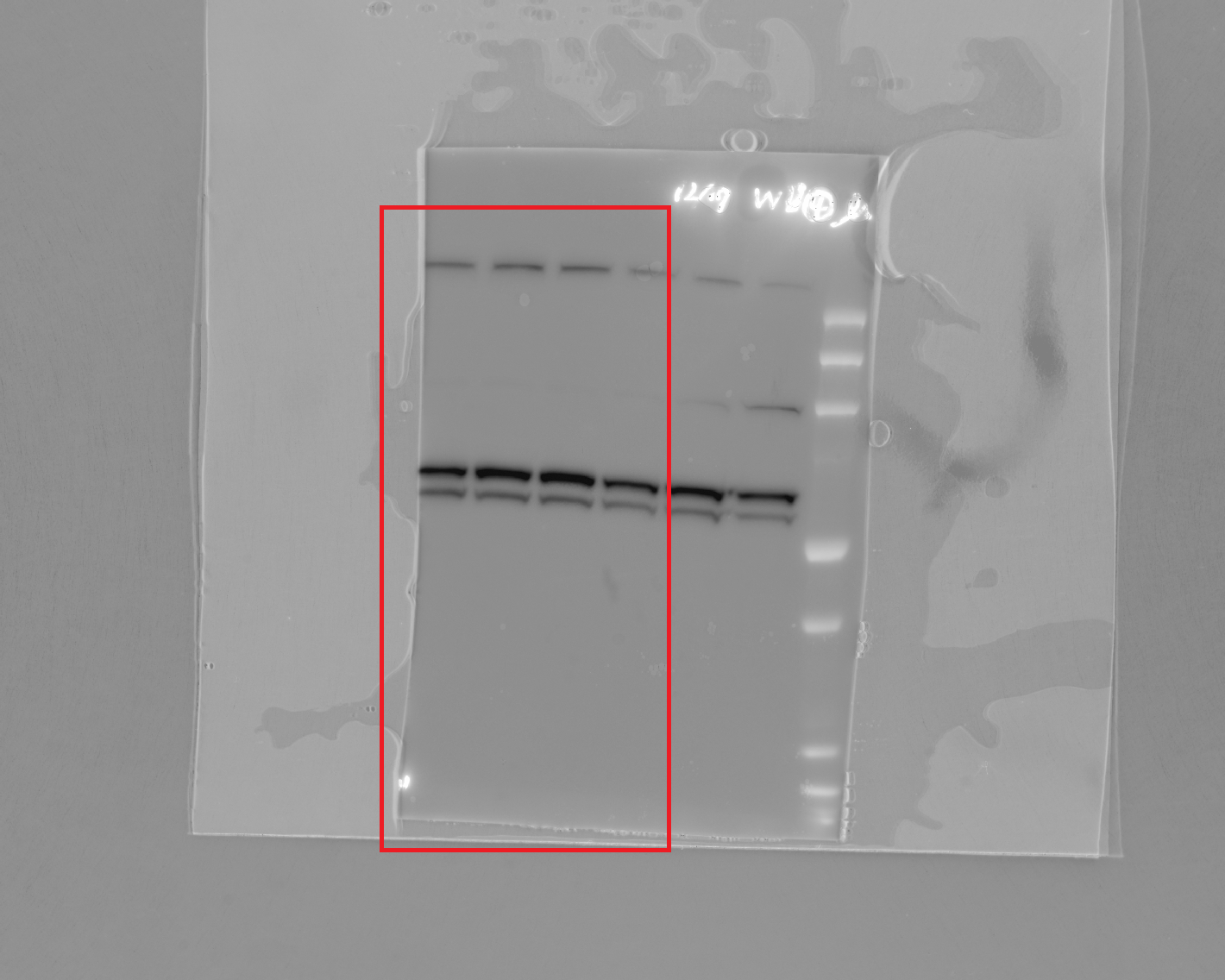

Supplement: Multimedia component 1 [file mmc1.zip › WB bands & raw densitometry/WB bands(24h)/5.p-AMPK/2.P-AMPK(Composite).tif]

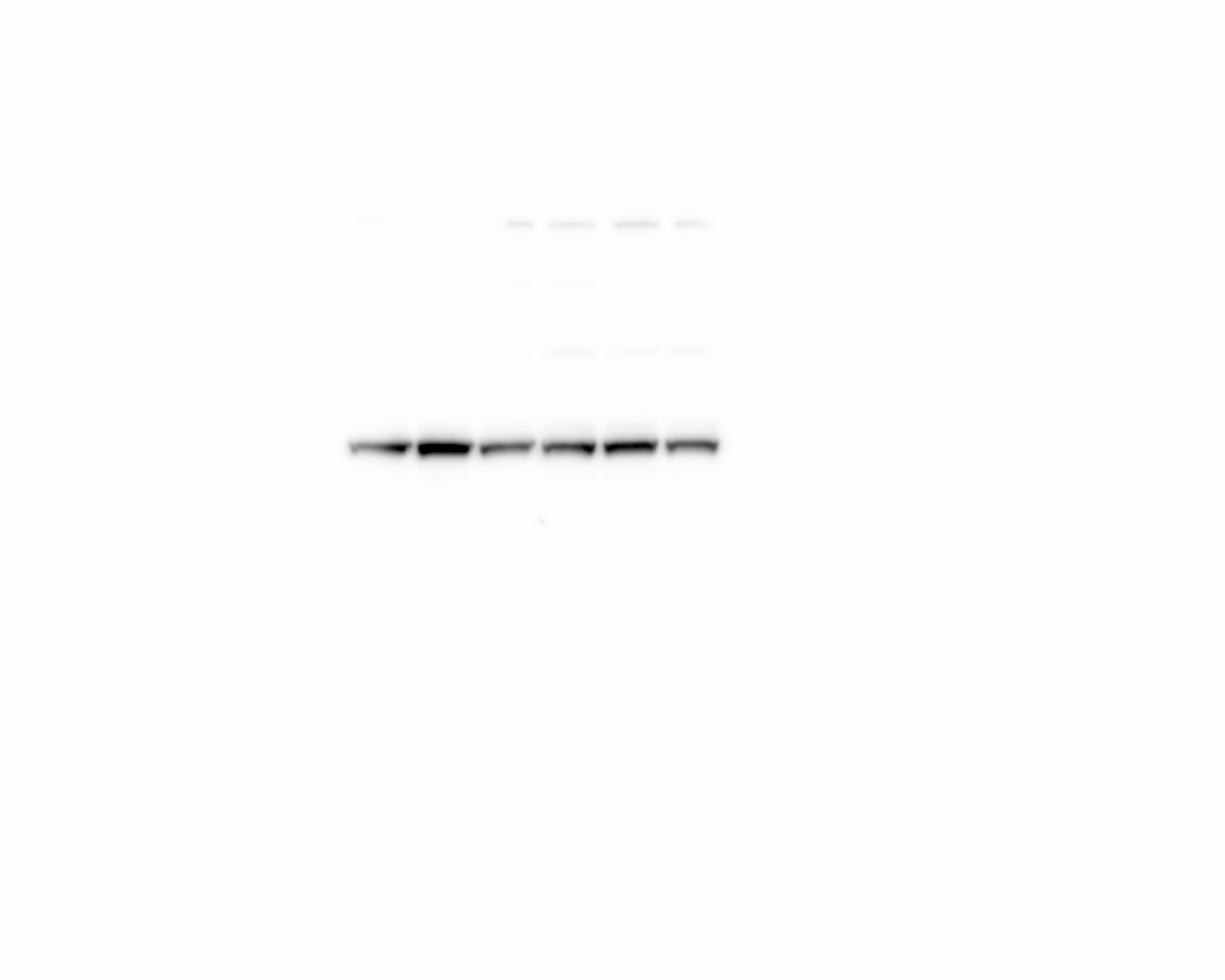

Supplement: Multimedia component 1 [file mmc1.zip › WB bands & raw densitometry/WB bands(24h)/5.p-AMPK/3.P-AMPK(Chemiluminescence).tif]

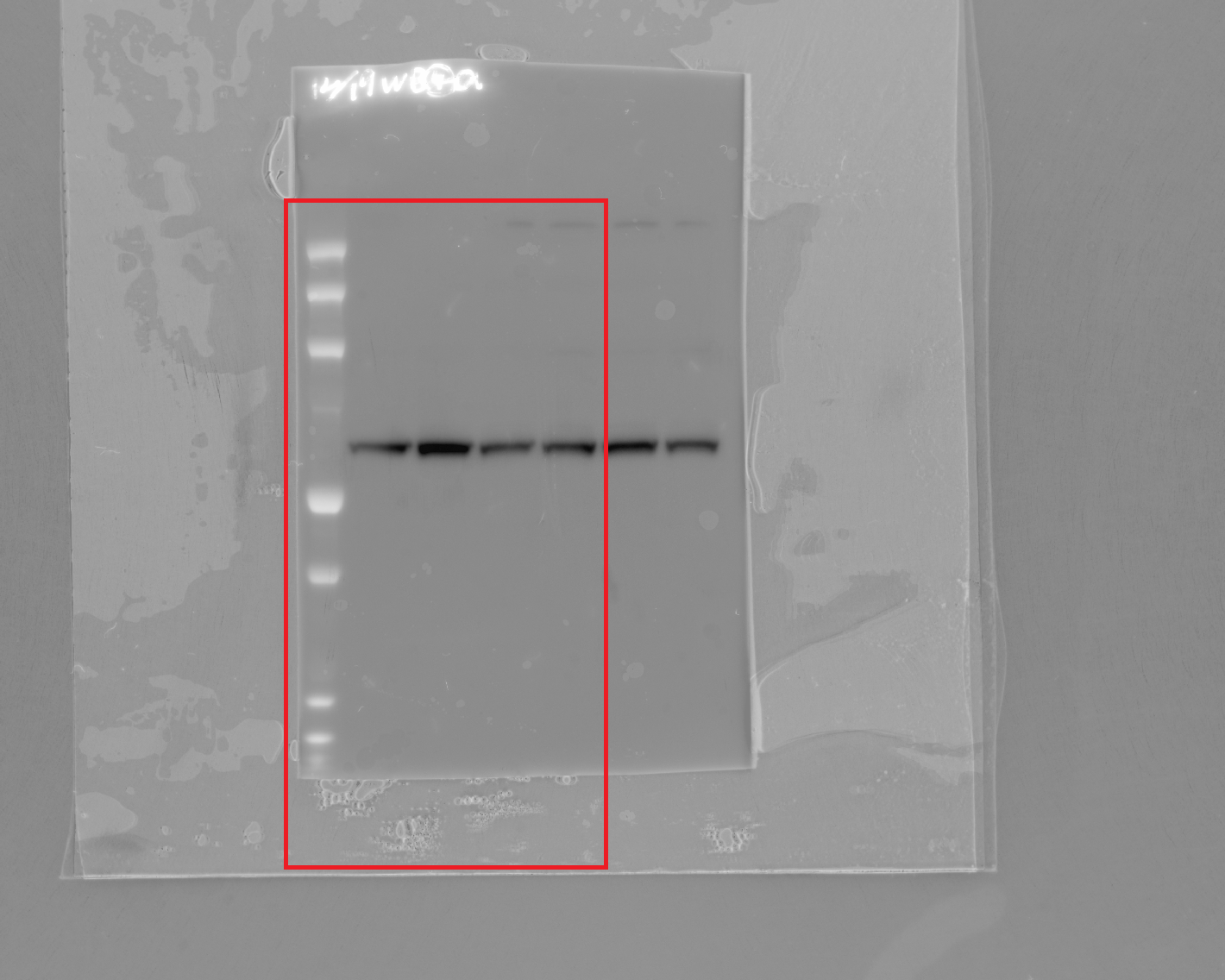

Supplement: Multimedia component 1 [file mmc1.zip › WB bands & raw densitometry/WB bands(24h)/5.p-AMPK/3.P-AMPK(Composite).tif]

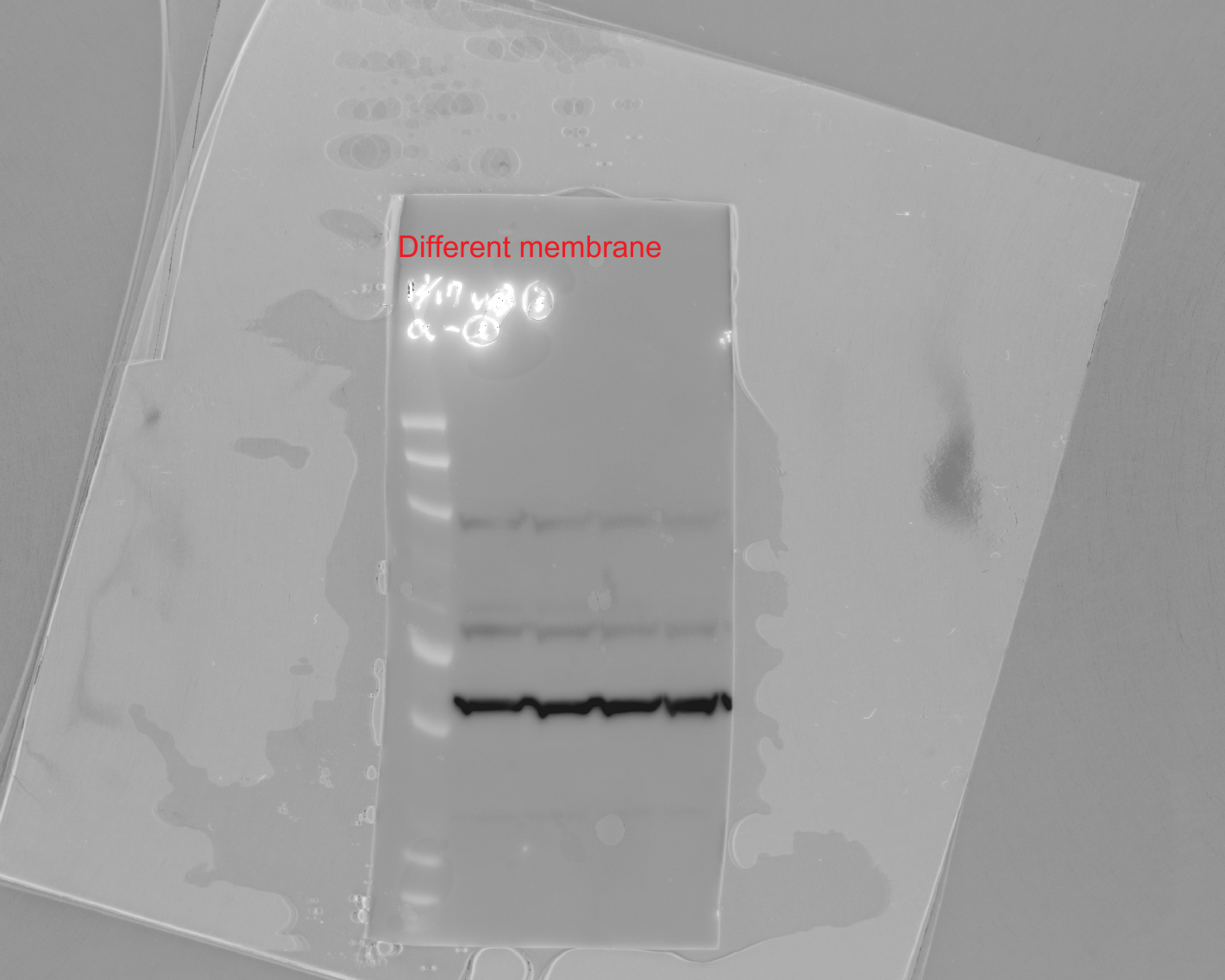

Supplement: Multimedia component 1 [file mmc1.zip › WB bands & raw densitometry/WB bands(24h)/6.eEF2/1.B-actin(Composite).tif]

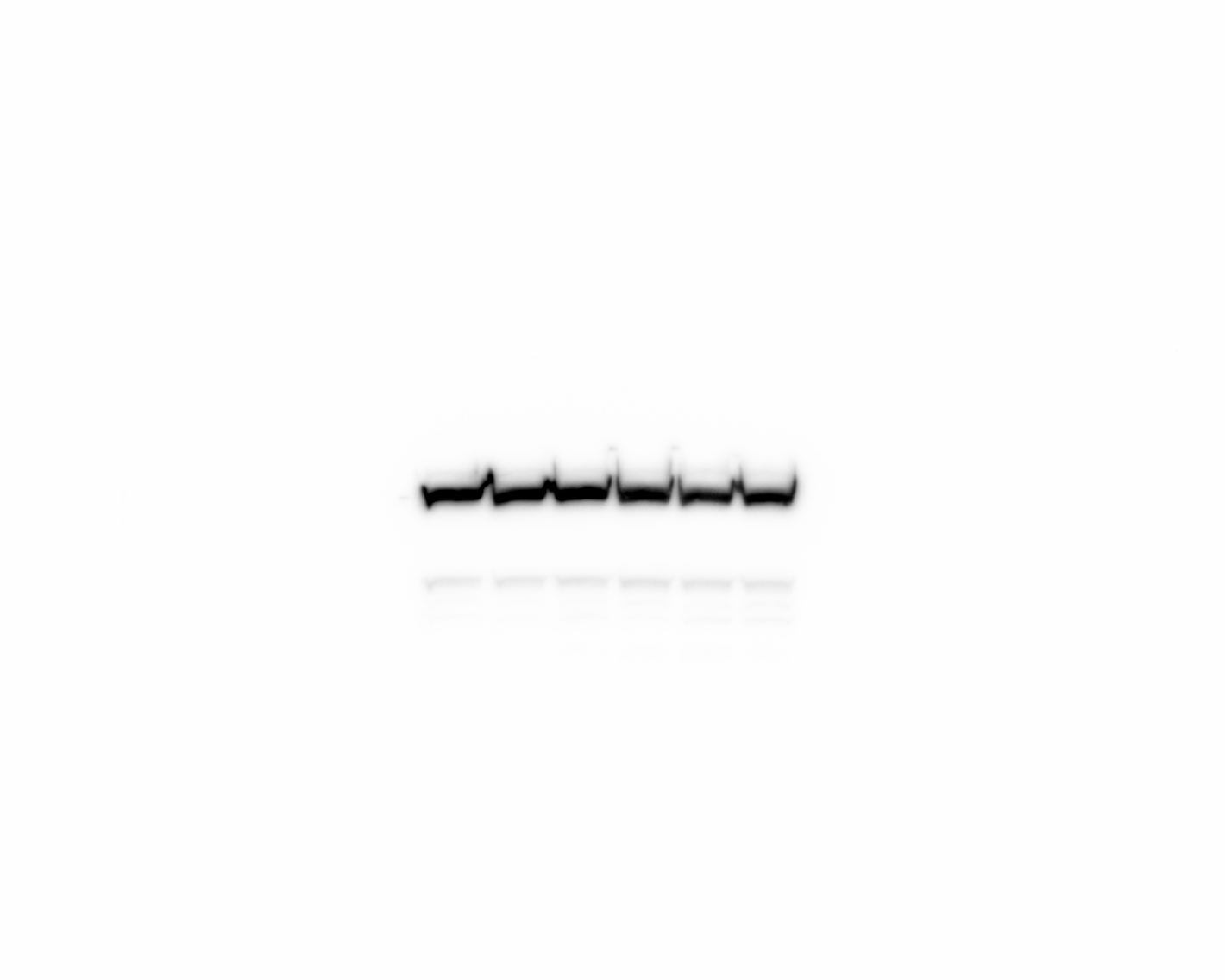

Supplement: Multimedia component 1 [file mmc1.zip › WB bands & raw densitometry/WB bands(24h)/6.eEF2/1.eEF2(Chemiluminescence).tif]

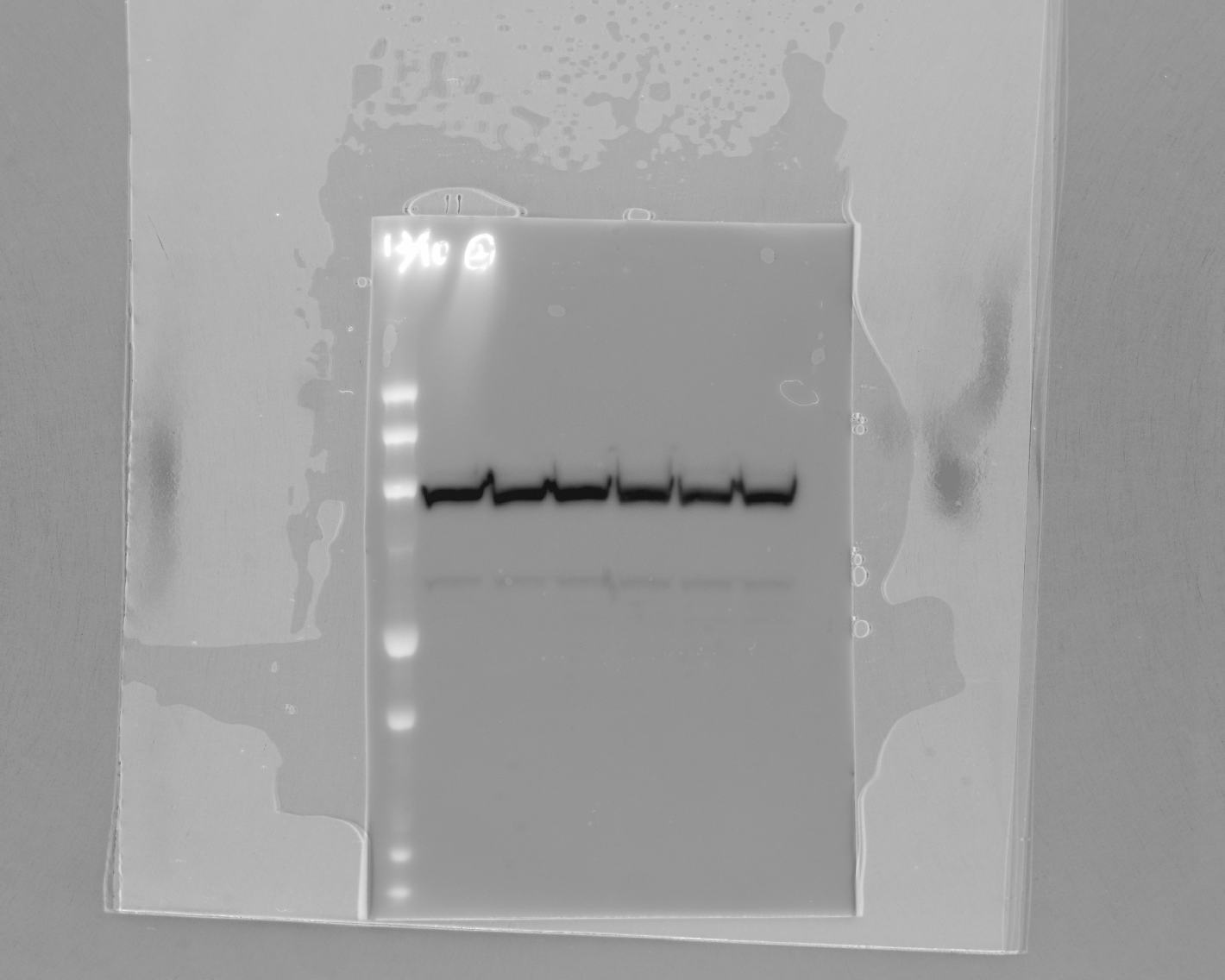

Supplement: Multimedia component 1 [file mmc1.zip › WB bands & raw densitometry/WB bands(24h)/6.eEF2/1.eEF2(Composite).tif]

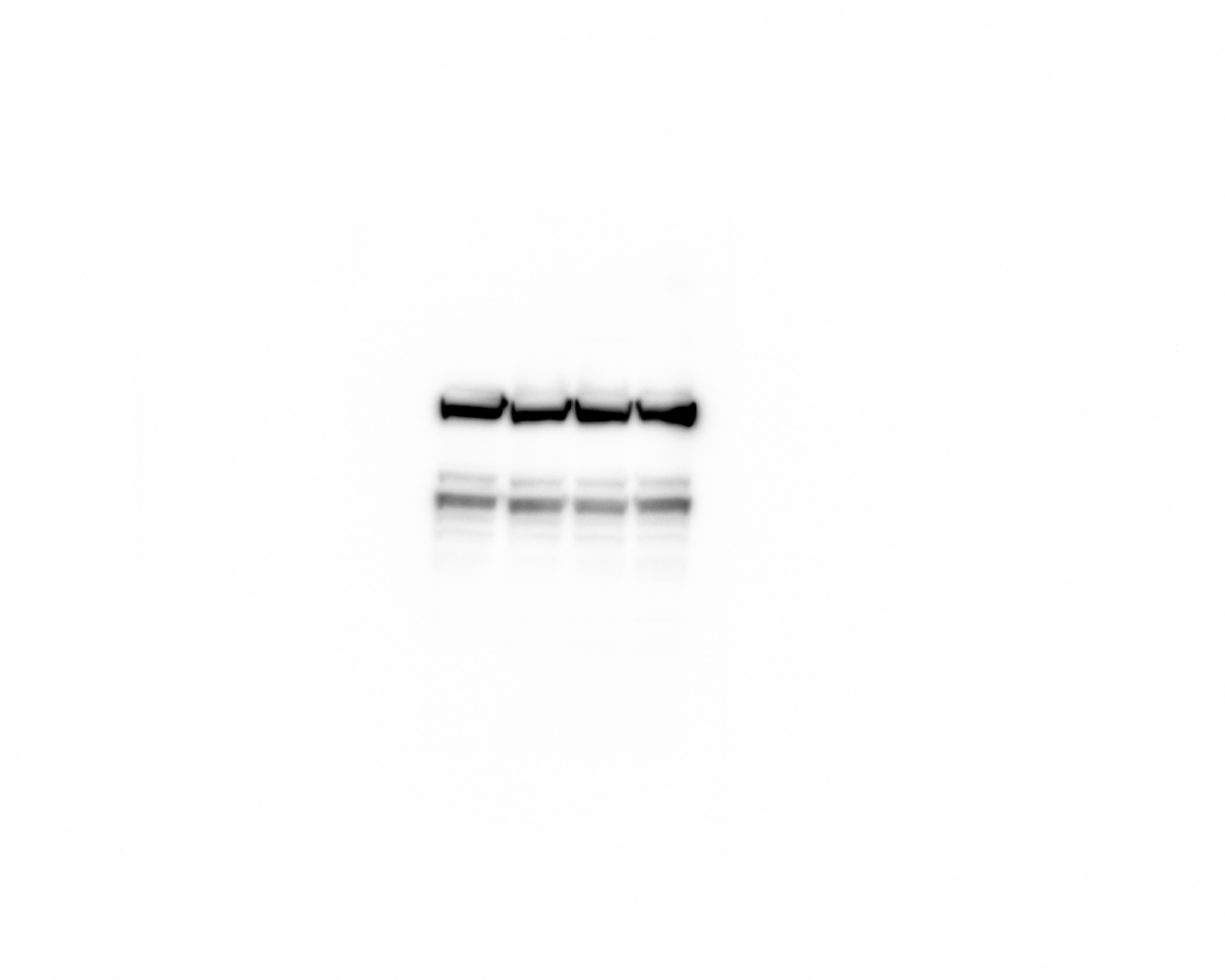

Supplement: Multimedia component 1 [file mmc1.zip › WB bands & raw densitometry/WB bands(24h)/6.eEF2/2.eEF2(Chemiluminescence).tif]

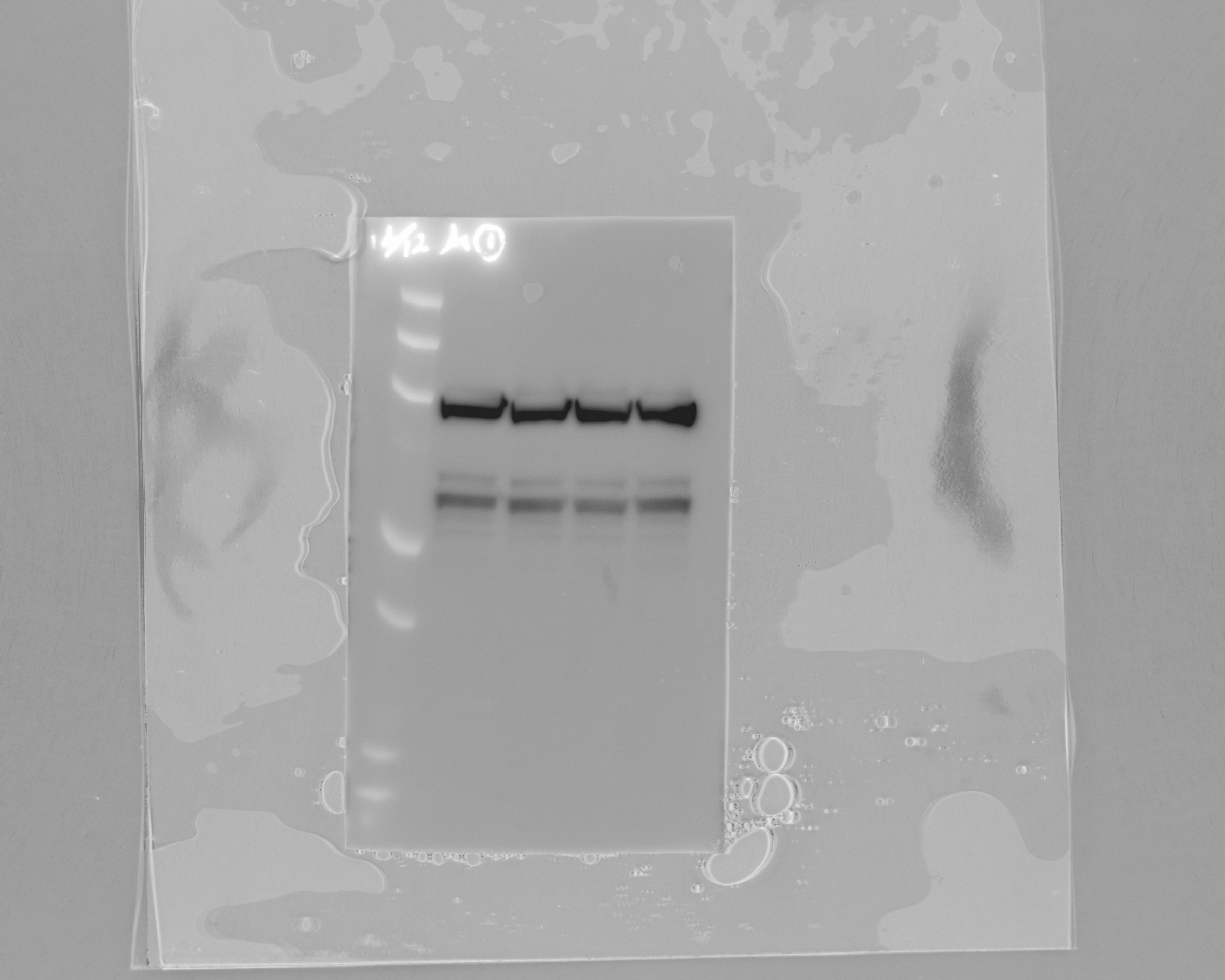

Supplement: Multimedia component 1 [file mmc1.zip › WB bands & raw densitometry/WB bands(24h)/6.eEF2/2.eEF2(Composite).tif]

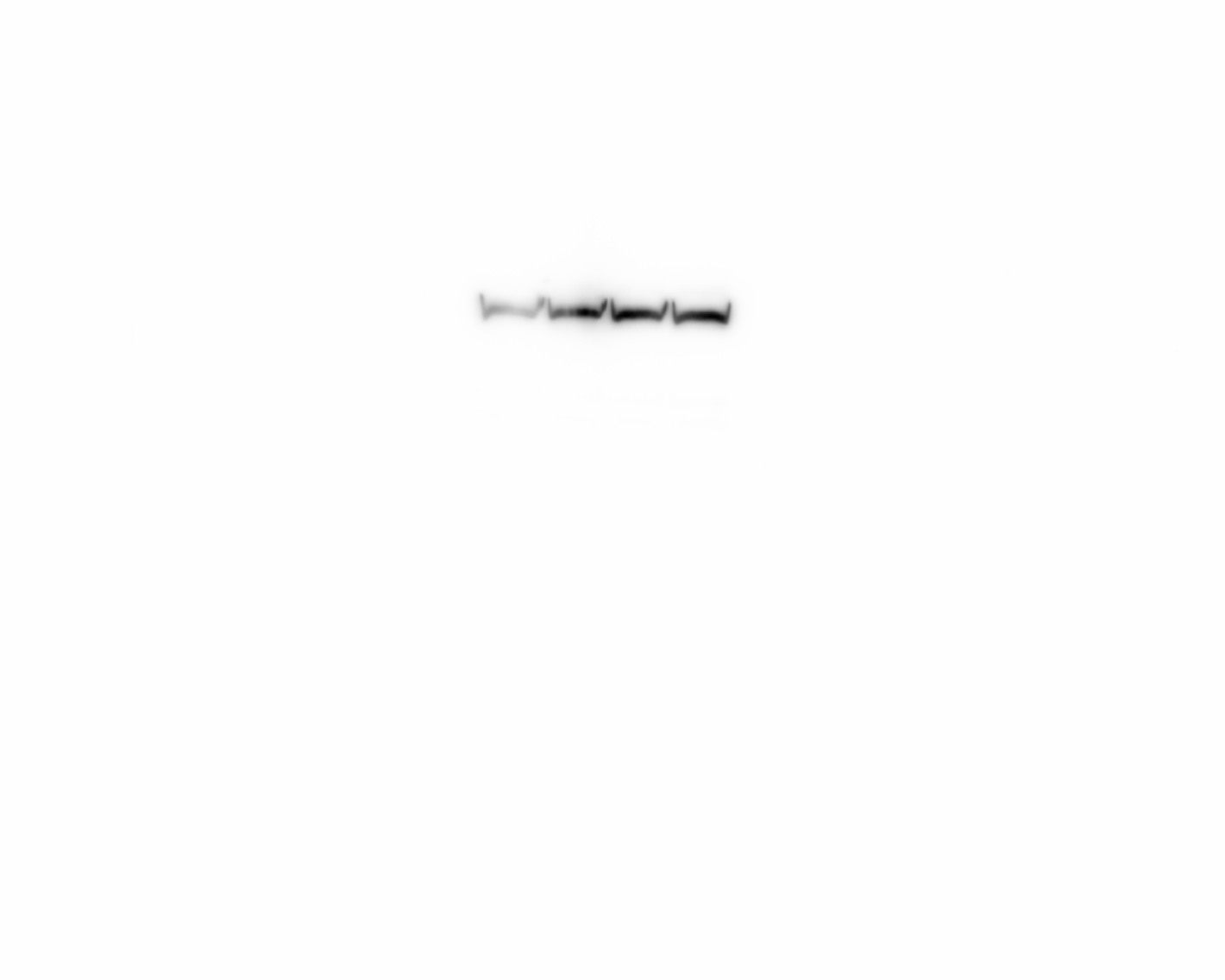

Supplement: Multimedia component 1 [file mmc1.zip › WB bands & raw densitometry/WB bands(24h)/6.eEF2/3.eEF2(Chemiluminescence).tif]

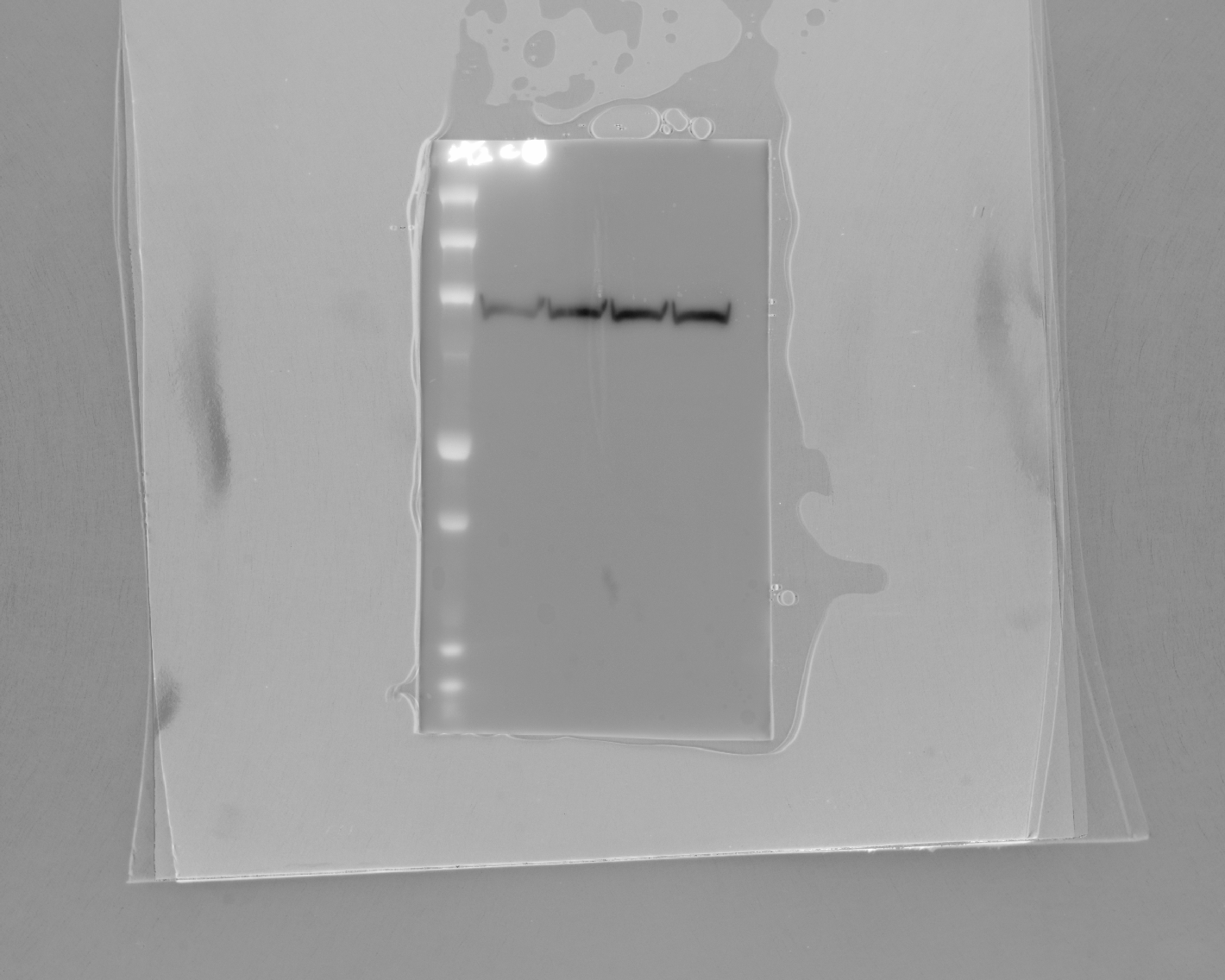

Supplement: Multimedia component 1 [file mmc1.zip › WB bands & raw densitometry/WB bands(24h)/6.eEF2/3.eEF2(Composite).tif]

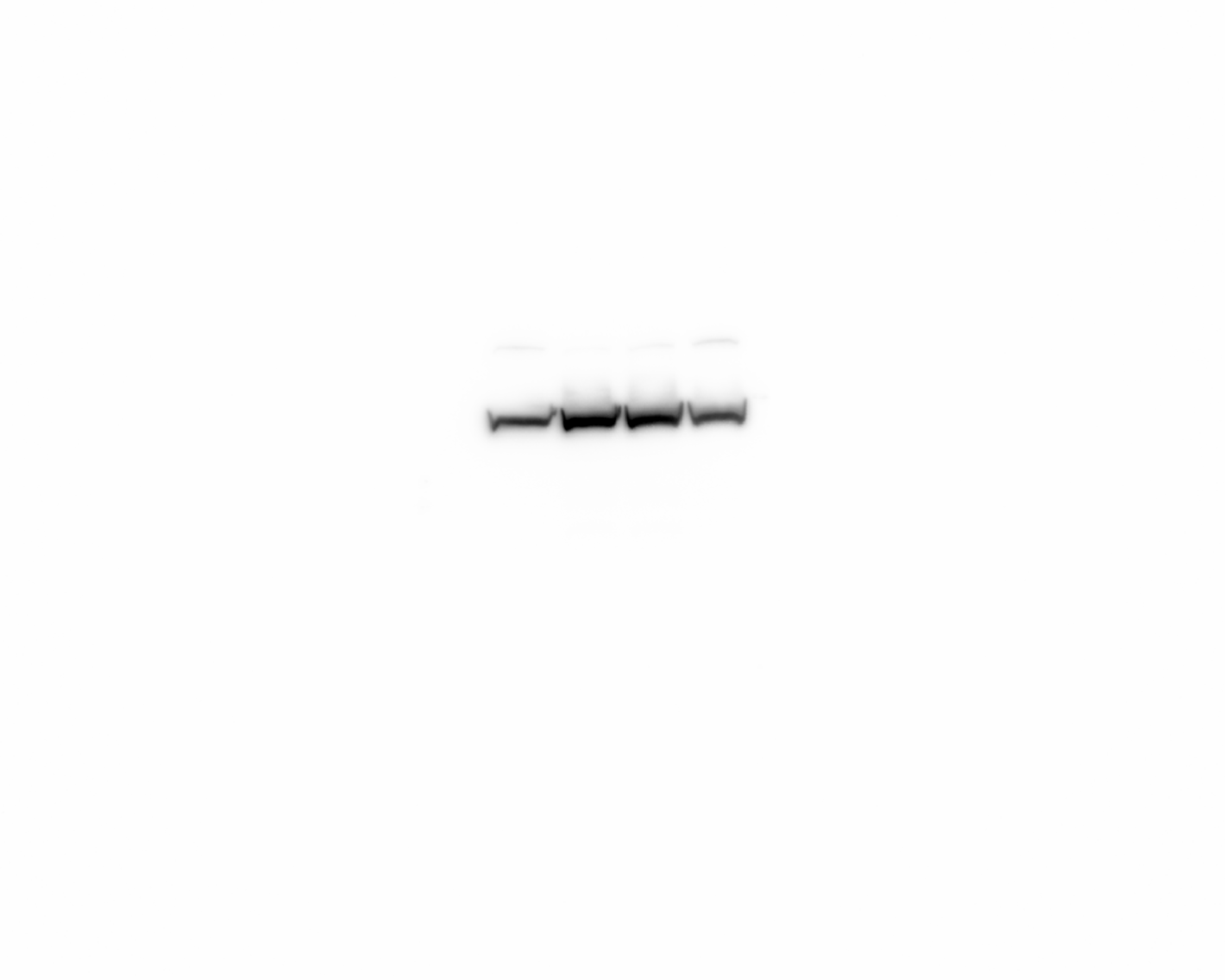

Supplement: Multimedia component 1 [file mmc1.zip › WB bands & raw densitometry/WB bands(24h)/7.P-eEF2/1.P-eEF2(Chemiluminescence).tif]

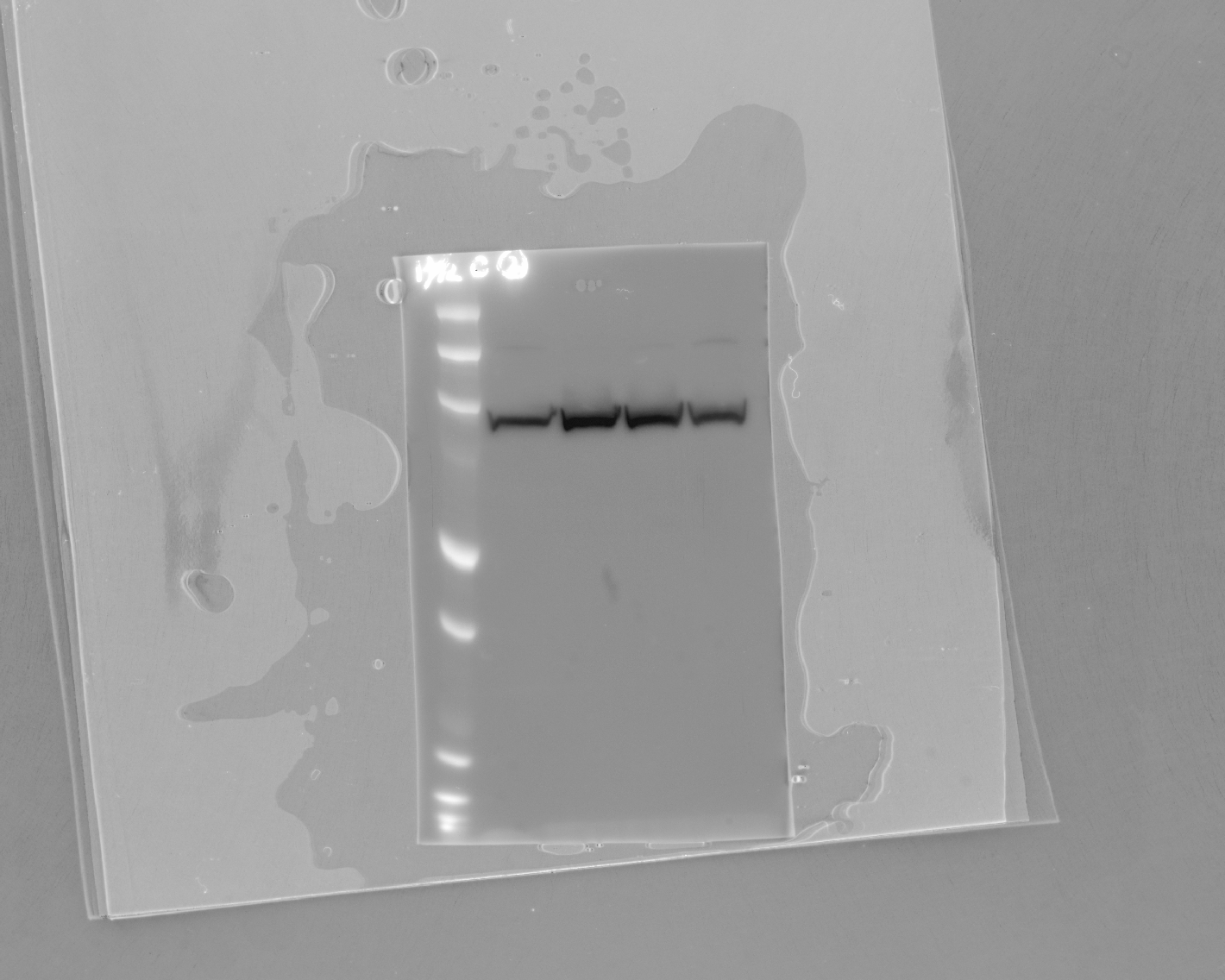

Supplement: Multimedia component 1 [file mmc1.zip › WB bands & raw densitometry/WB bands(24h)/7.P-eEF2/1.P-eEF2(Composite).tif]

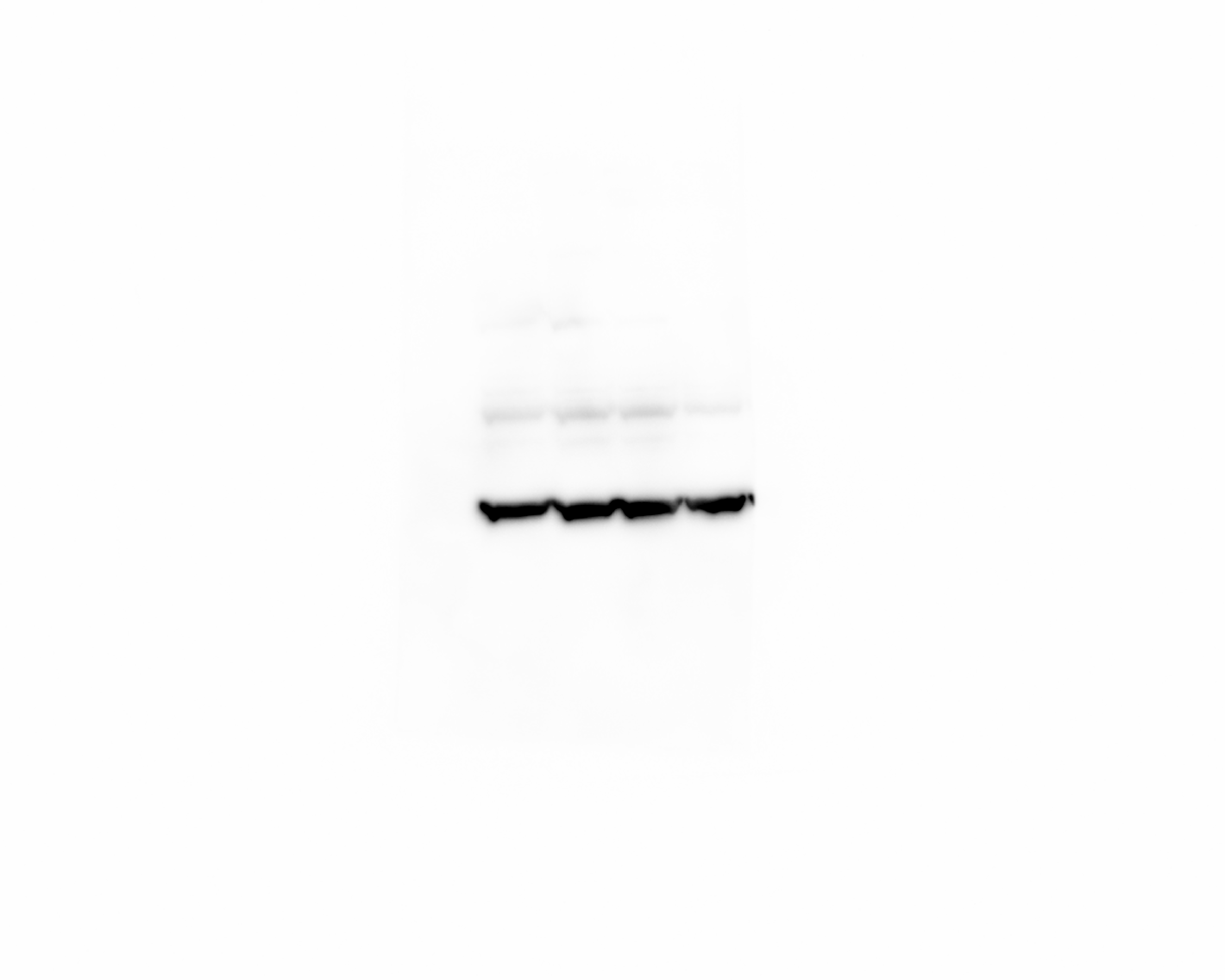

Supplement: Multimedia component 1 [file mmc1.zip › WB bands & raw densitometry/WB bands(24h)/7.P-eEF2/2.Bactin(Chemiluminescence).tif]

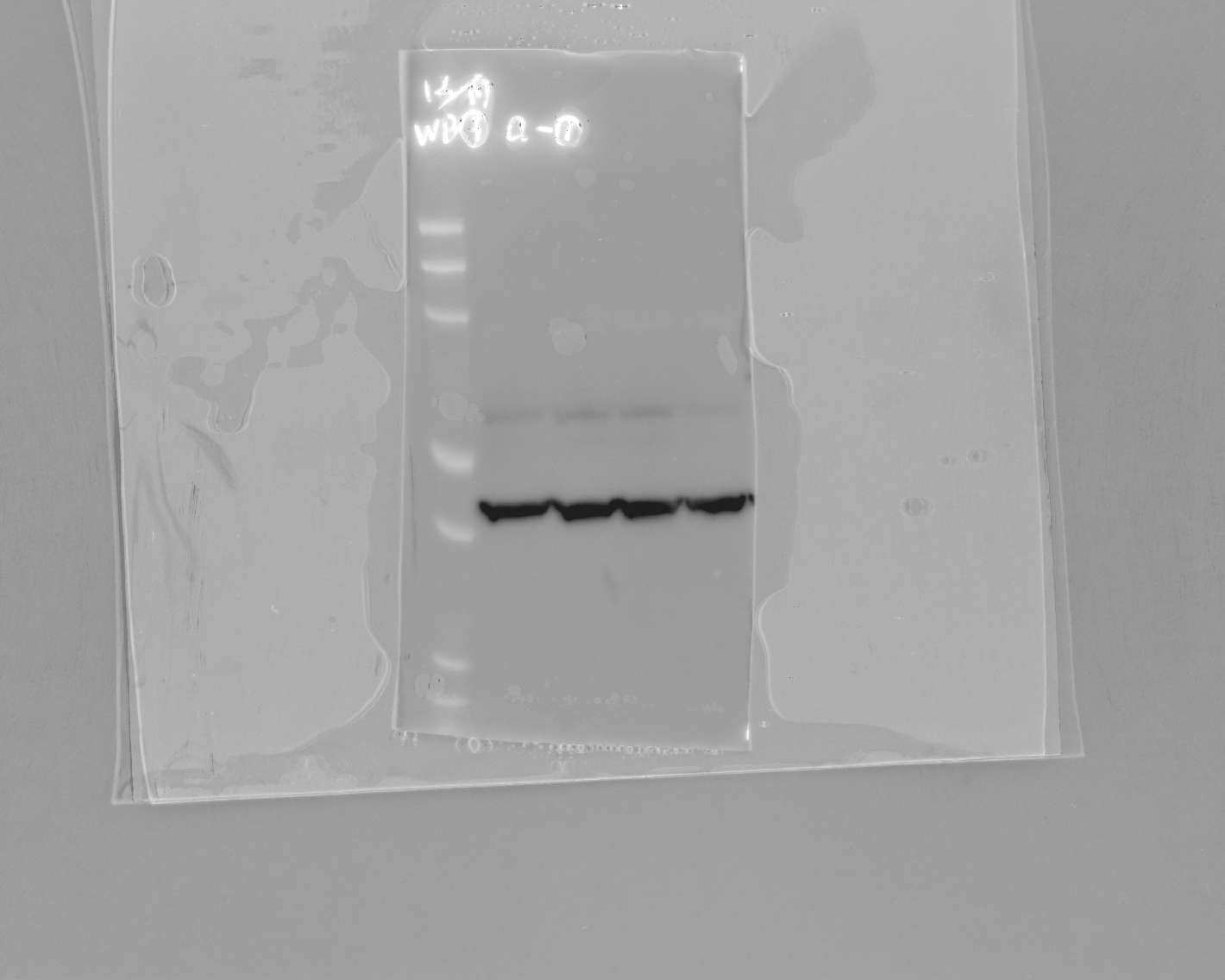

Supplement: Multimedia component 1 [file mmc1.zip › WB bands & raw densitometry/WB bands(24h)/7.P-eEF2/2.Bactin(Composite).tif]

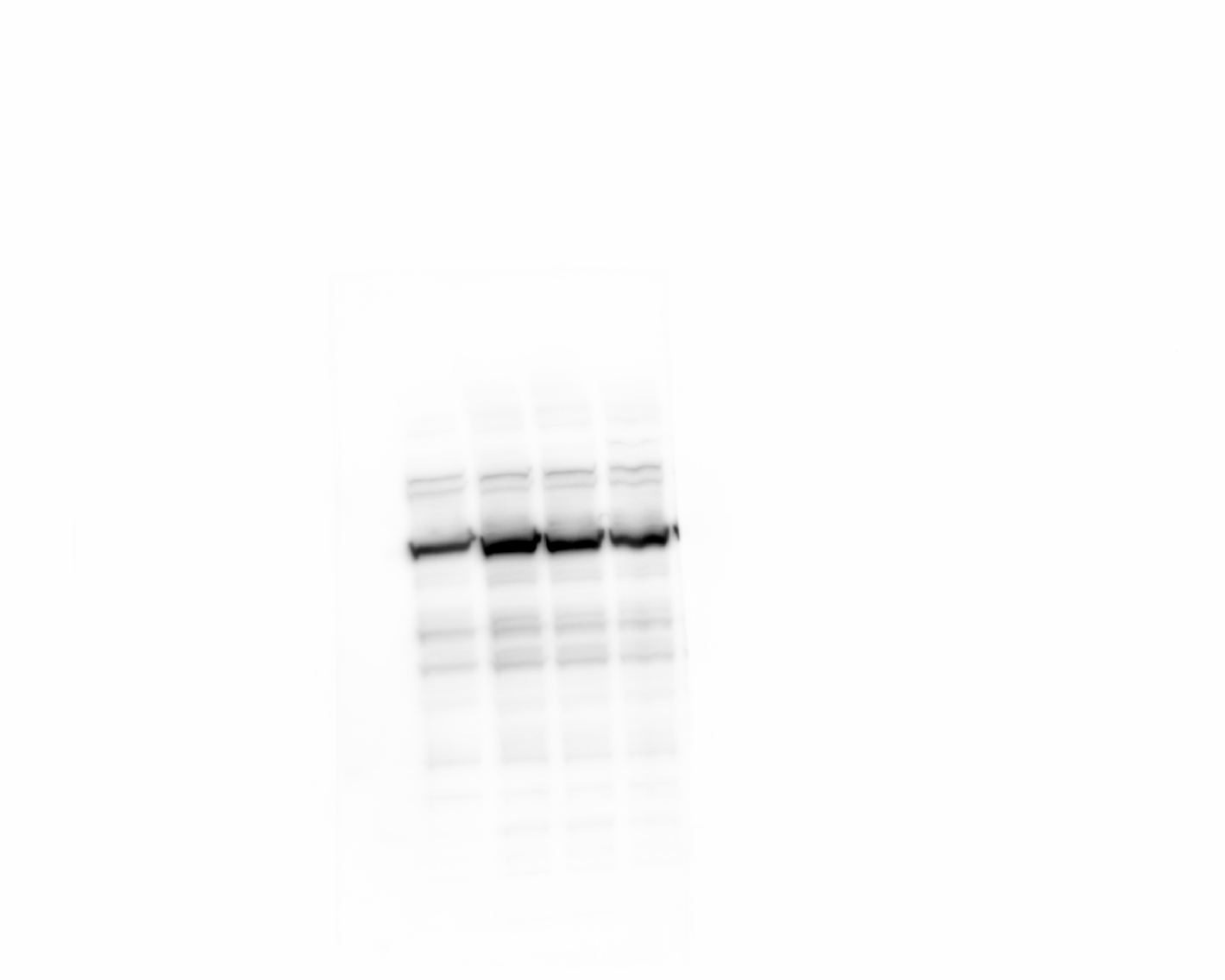

Supplement: Multimedia component 1 [file mmc1.zip › WB bands & raw densitometry/WB bands(24h)/7.P-eEF2/2.P-eEF2(Chemiluminescence).tif]

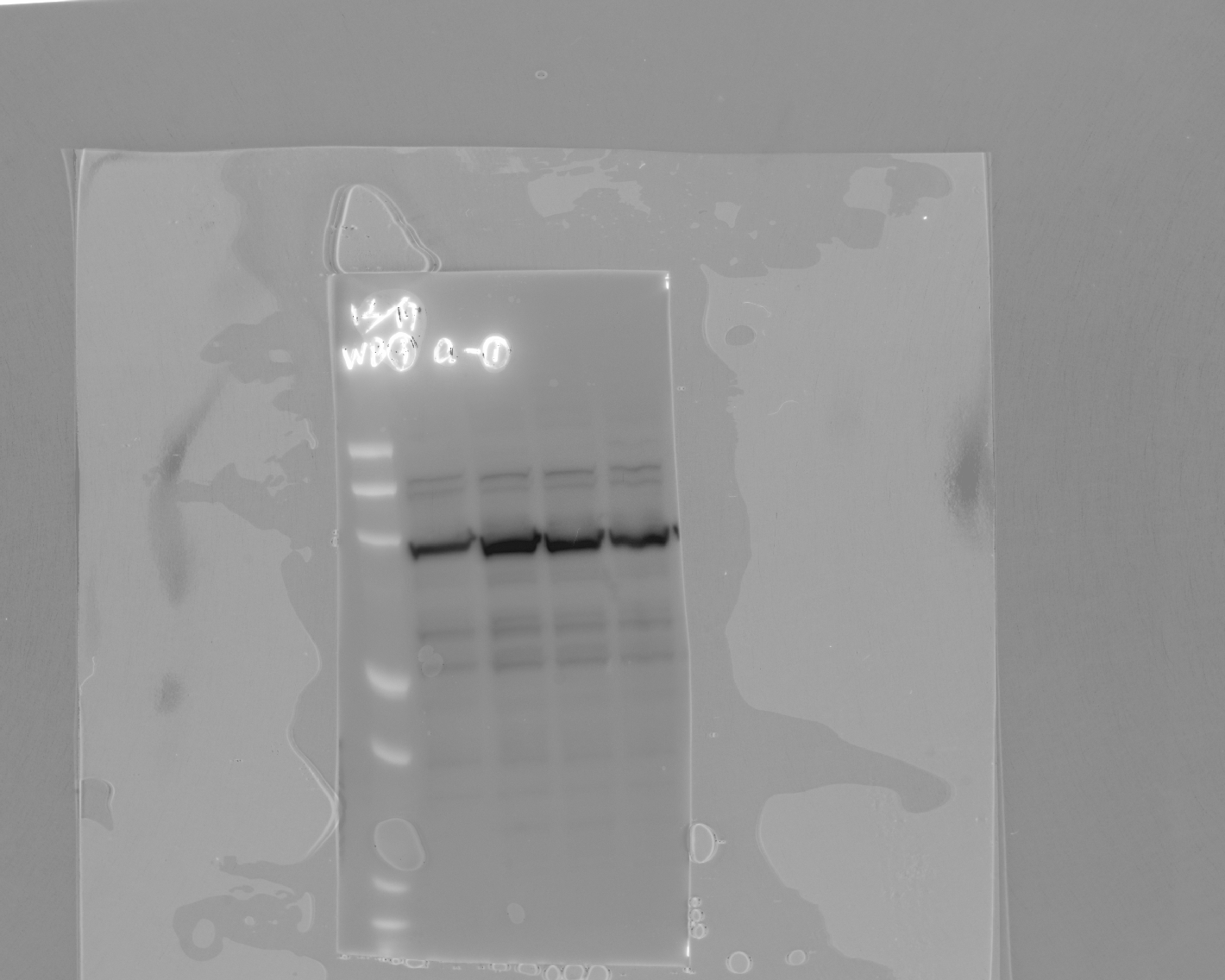

Supplement: Multimedia component 1 [file mmc1.zip › WB bands & raw densitometry/WB bands(24h)/7.P-eEF2/2.P-eEF2(Composite).tif]

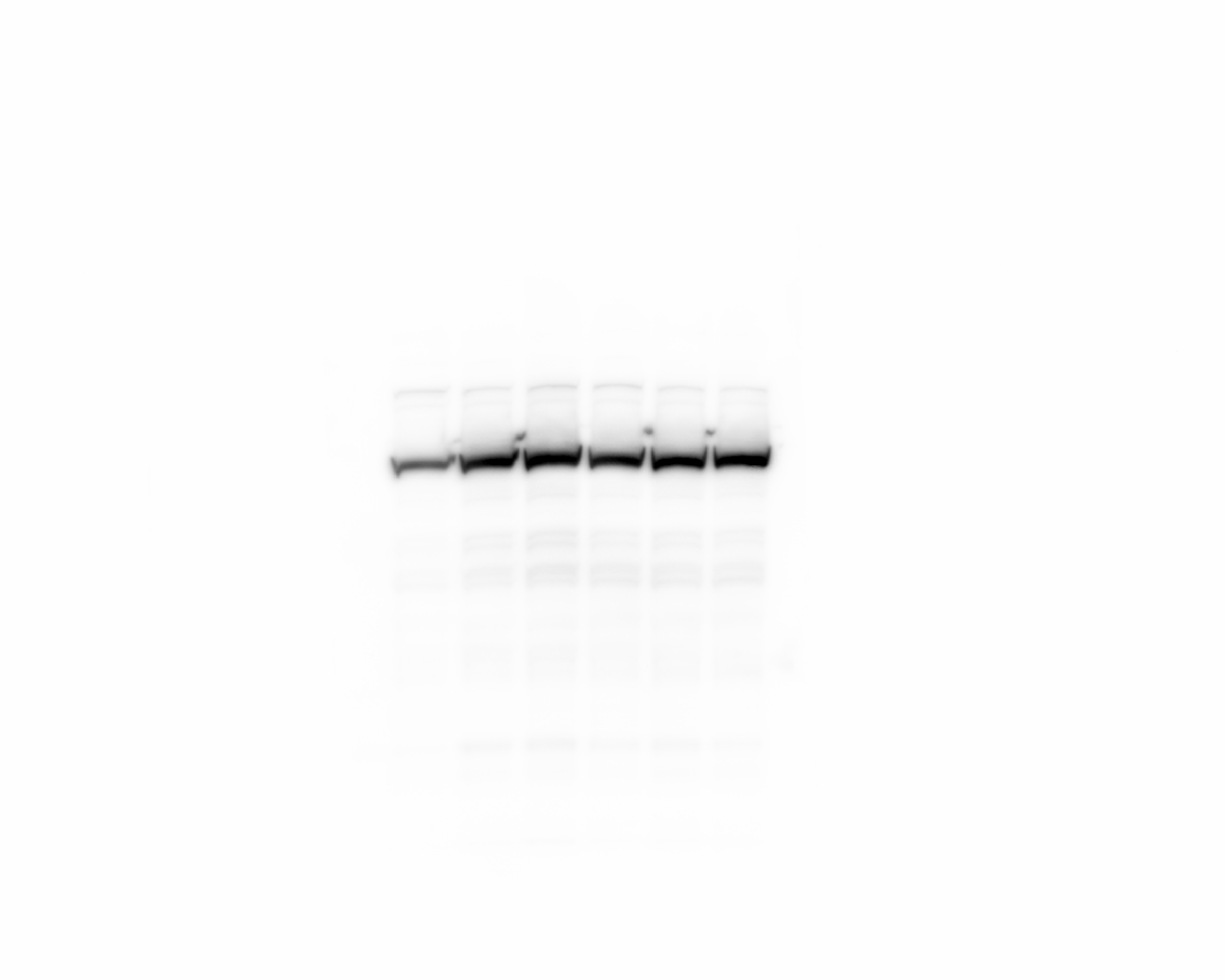

Supplement: Multimedia component 1 [file mmc1.zip › WB bands & raw densitometry/WB bands(24h)/7.P-eEF2/3.P-eEF2(Chemiluminescence).tif]

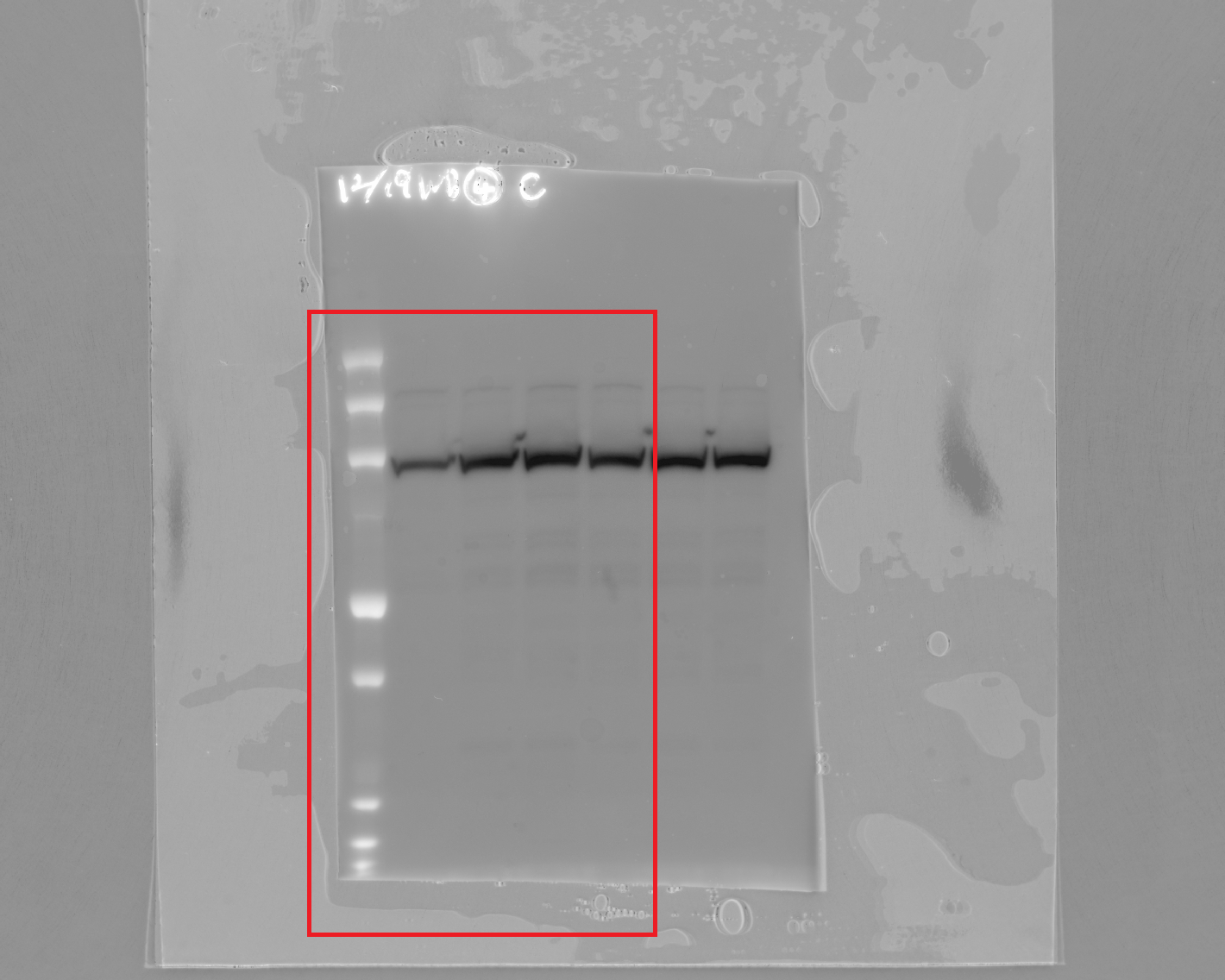

Supplement: Multimedia component 1 [file mmc1.zip › WB bands & raw densitometry/WB bands(24h)/7.P-eEF2/3.P-eEF2(Composite).tif]

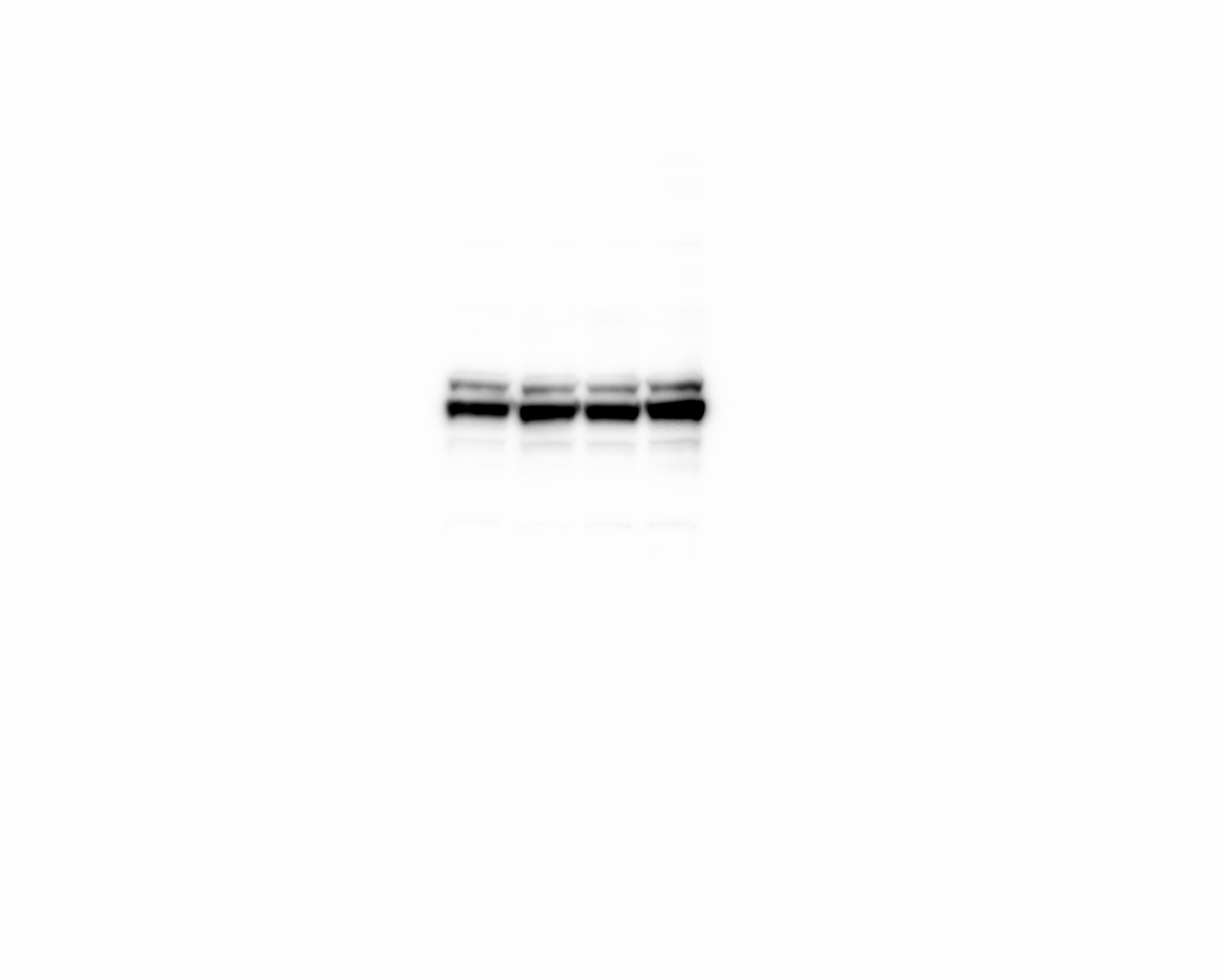

Supplement: Multimedia component 1 [file mmc1.zip › WB bands & raw densitometry/WB bands(24h)/8.70s6k/1.s6k(Chemiluminescence).tif]

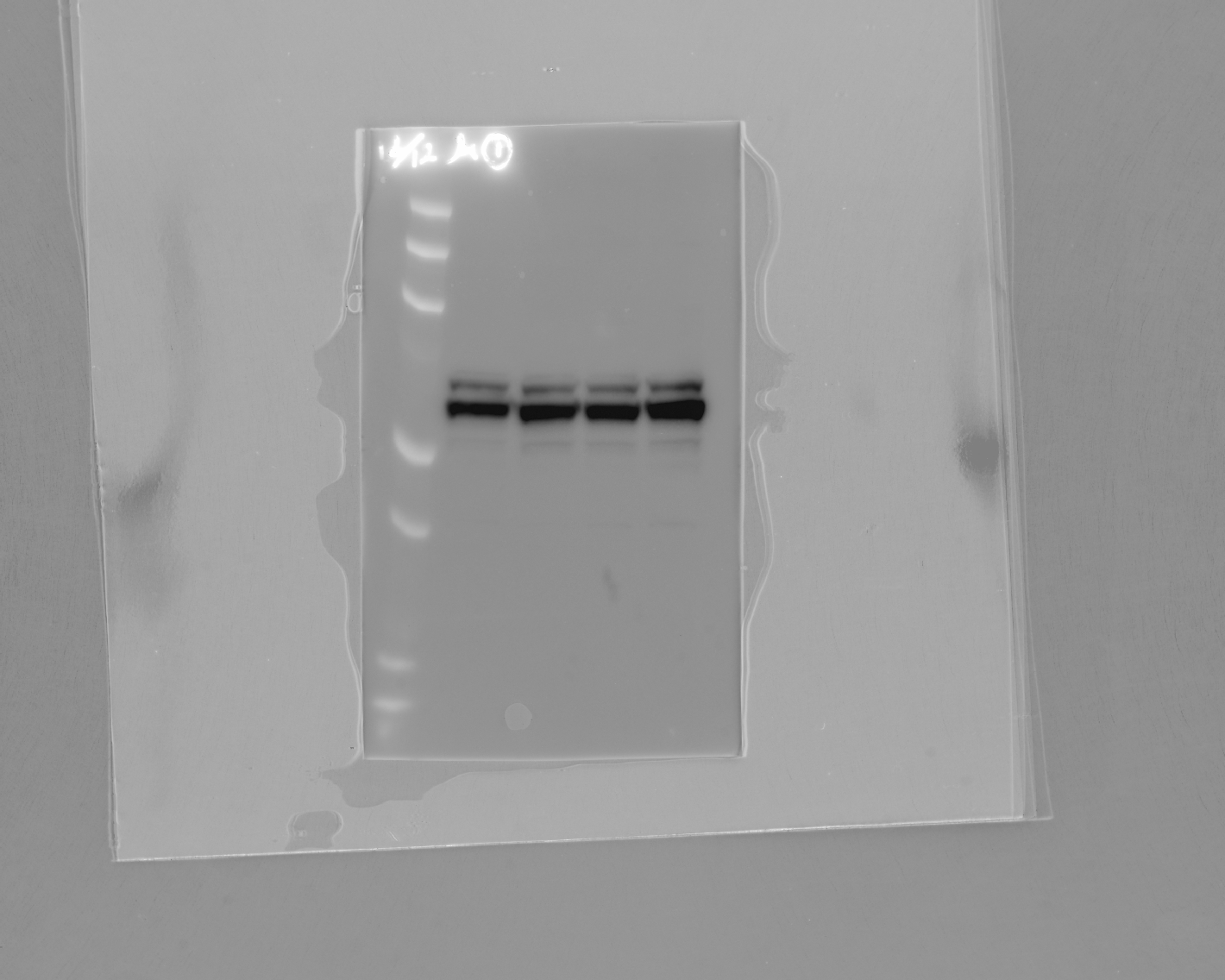

Supplement: Multimedia component 1 [file mmc1.zip › WB bands & raw densitometry/WB bands(24h)/8.70s6k/1.s6k(Composite).tif]

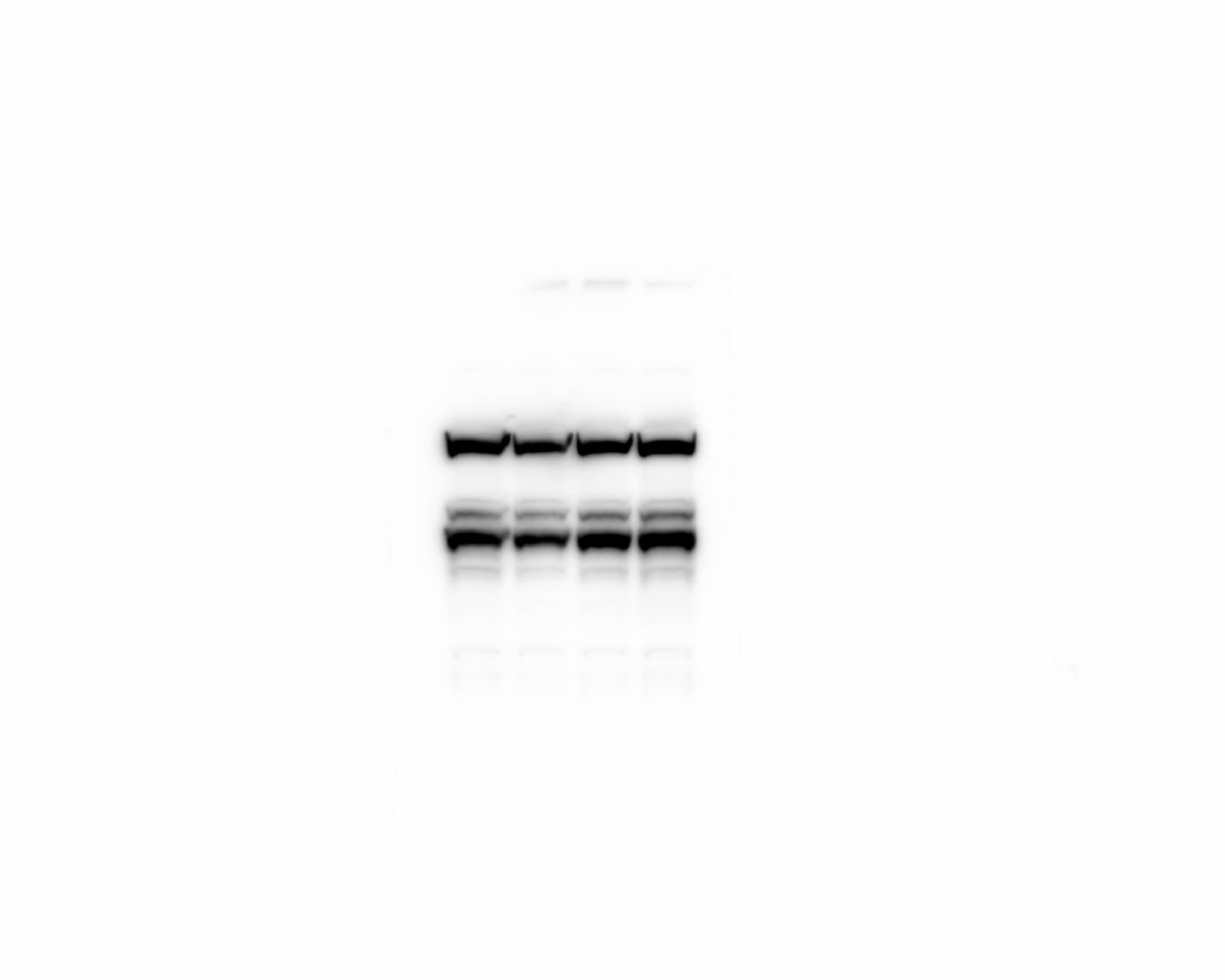

Supplement: Multimedia component 1 [file mmc1.zip › WB bands & raw densitometry/WB bands(24h)/8.70s6k/2.s6k(Chemiluminescence).tif]

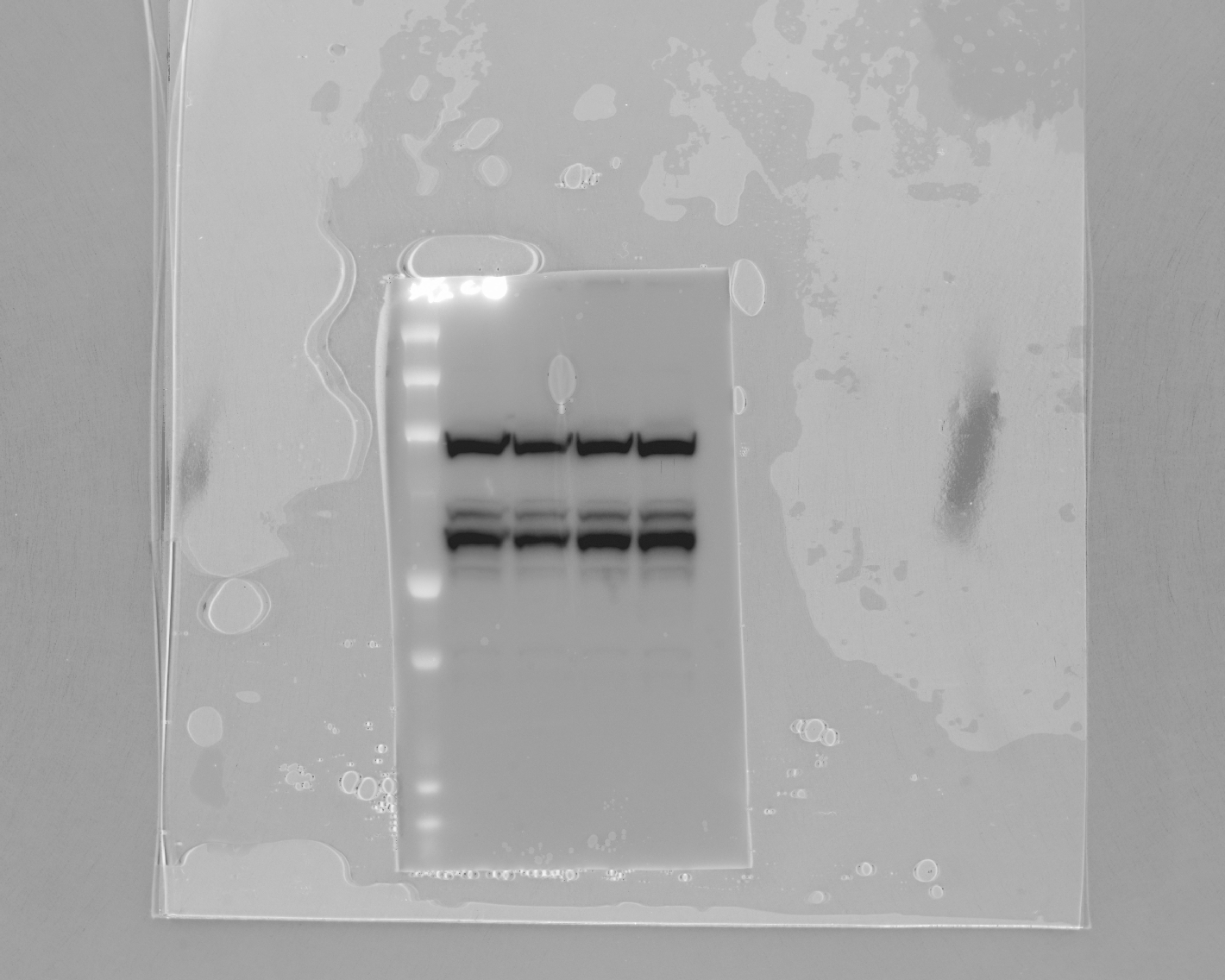

Supplement: Multimedia component 1 [file mmc1.zip › WB bands & raw densitometry/WB bands(24h)/8.70s6k/2.s6k(Composite).tif]

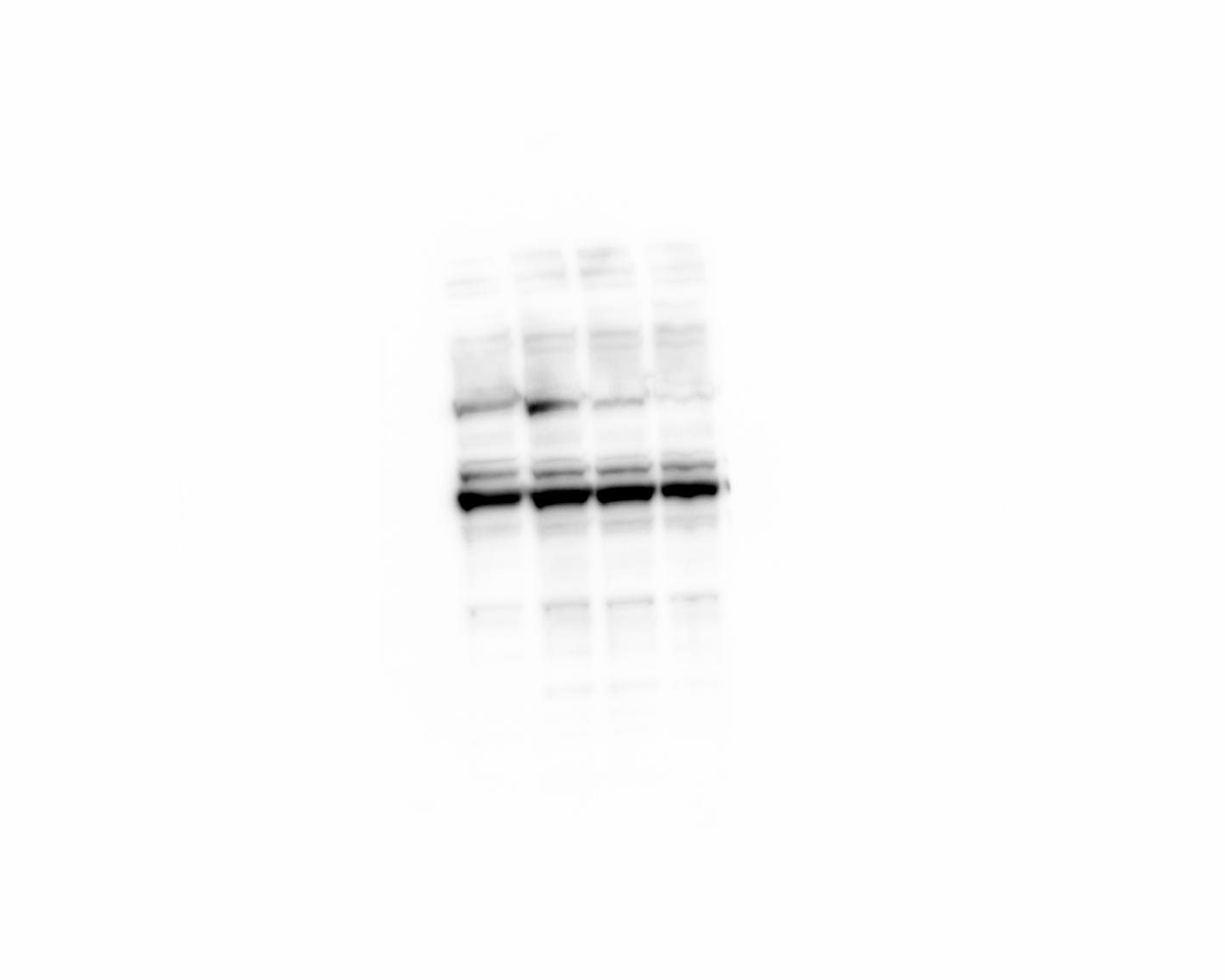

Supplement: Multimedia component 1 [file mmc1.zip › WB bands & raw densitometry/WB bands(24h)/8.70s6k/3.s6k(Chemiluminescence).tif]

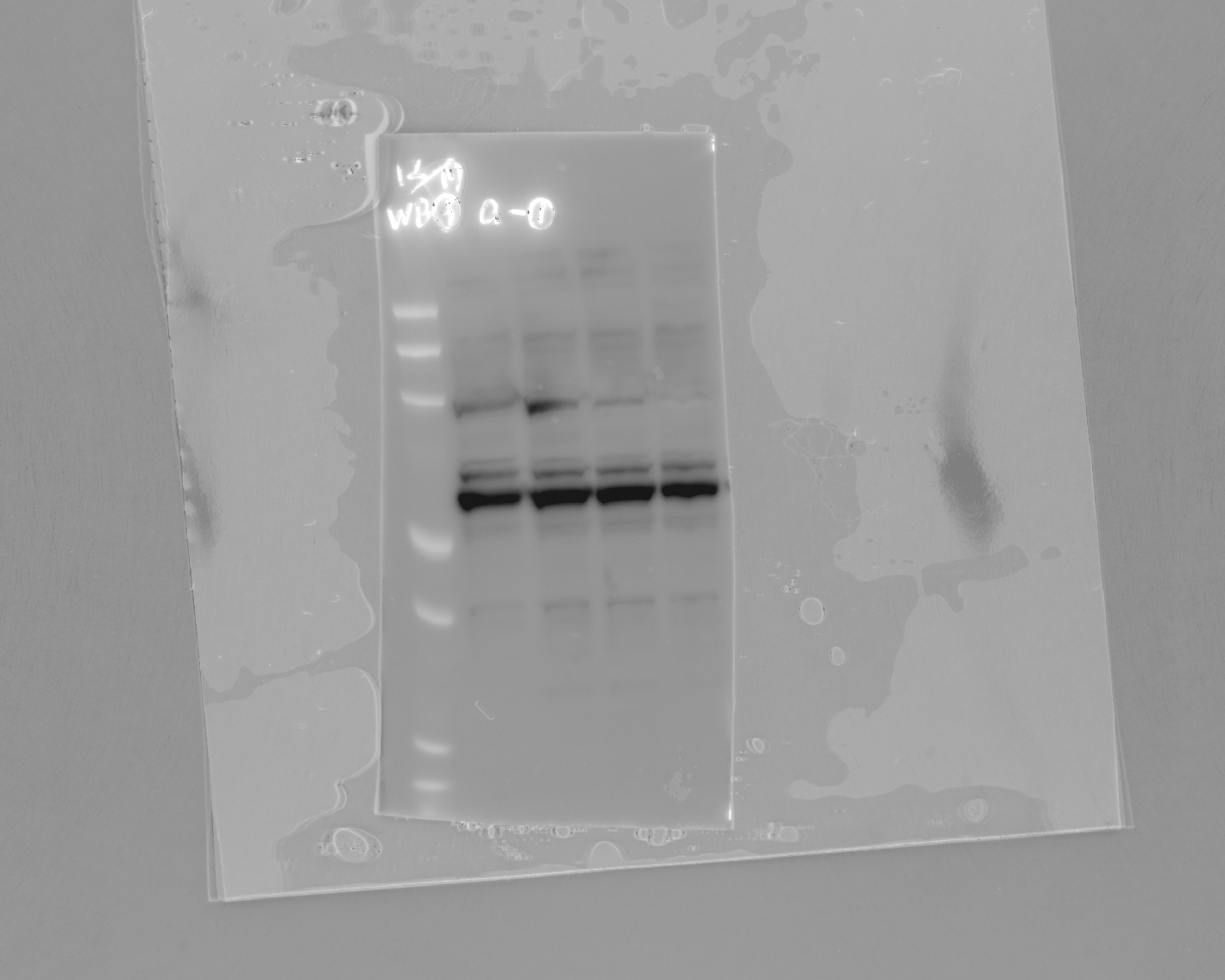

Supplement: Multimedia component 1 [file mmc1.zip › WB bands & raw densitometry/WB bands(24h)/8.70s6k/3.s6k(Composite).tif]

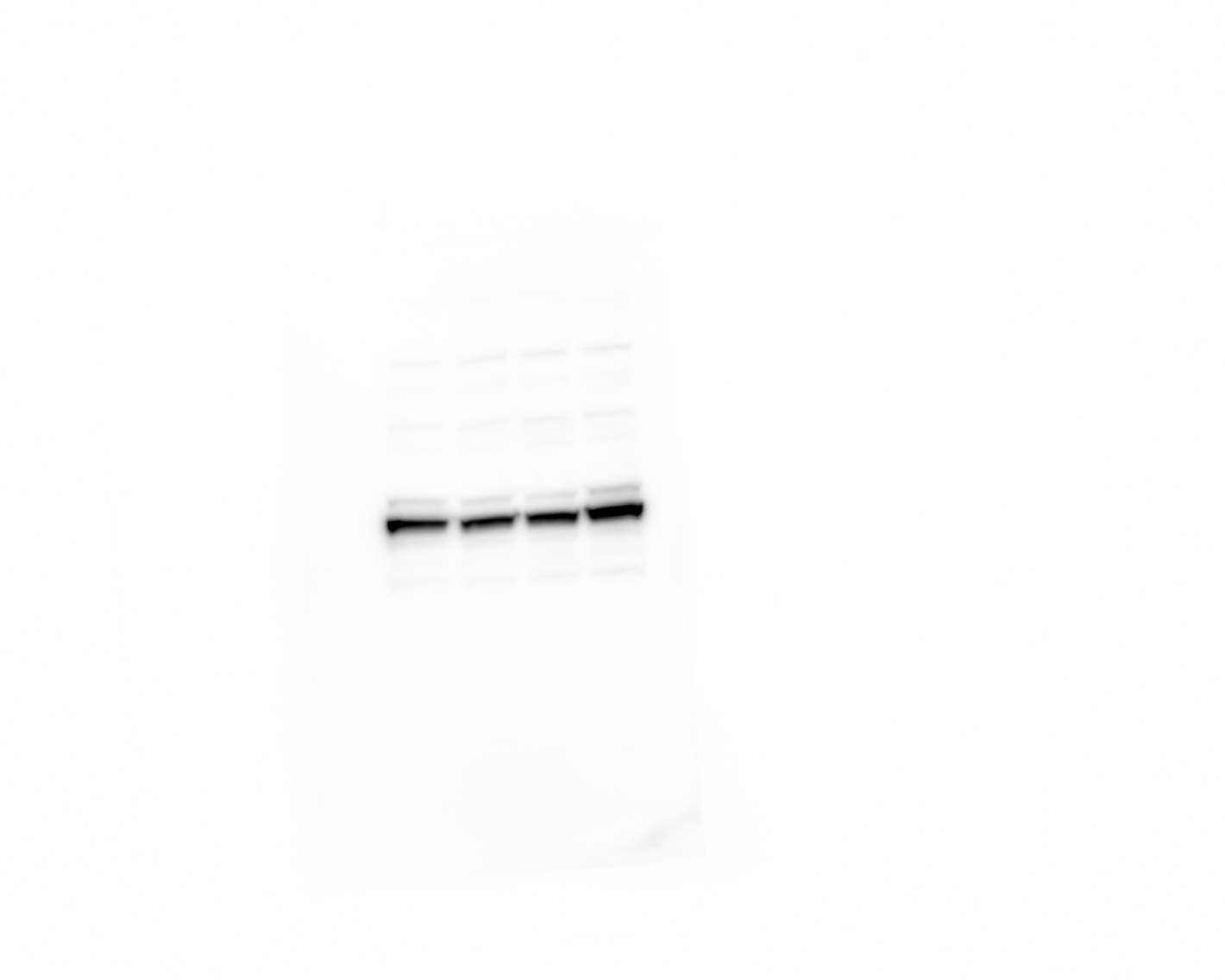

Supplement: Multimedia component 1 [file mmc1.zip › WB bands & raw densitometry/WB bands(24h)/9.P-70s6k/1.P-s6k(Chemiluminescence).tif]

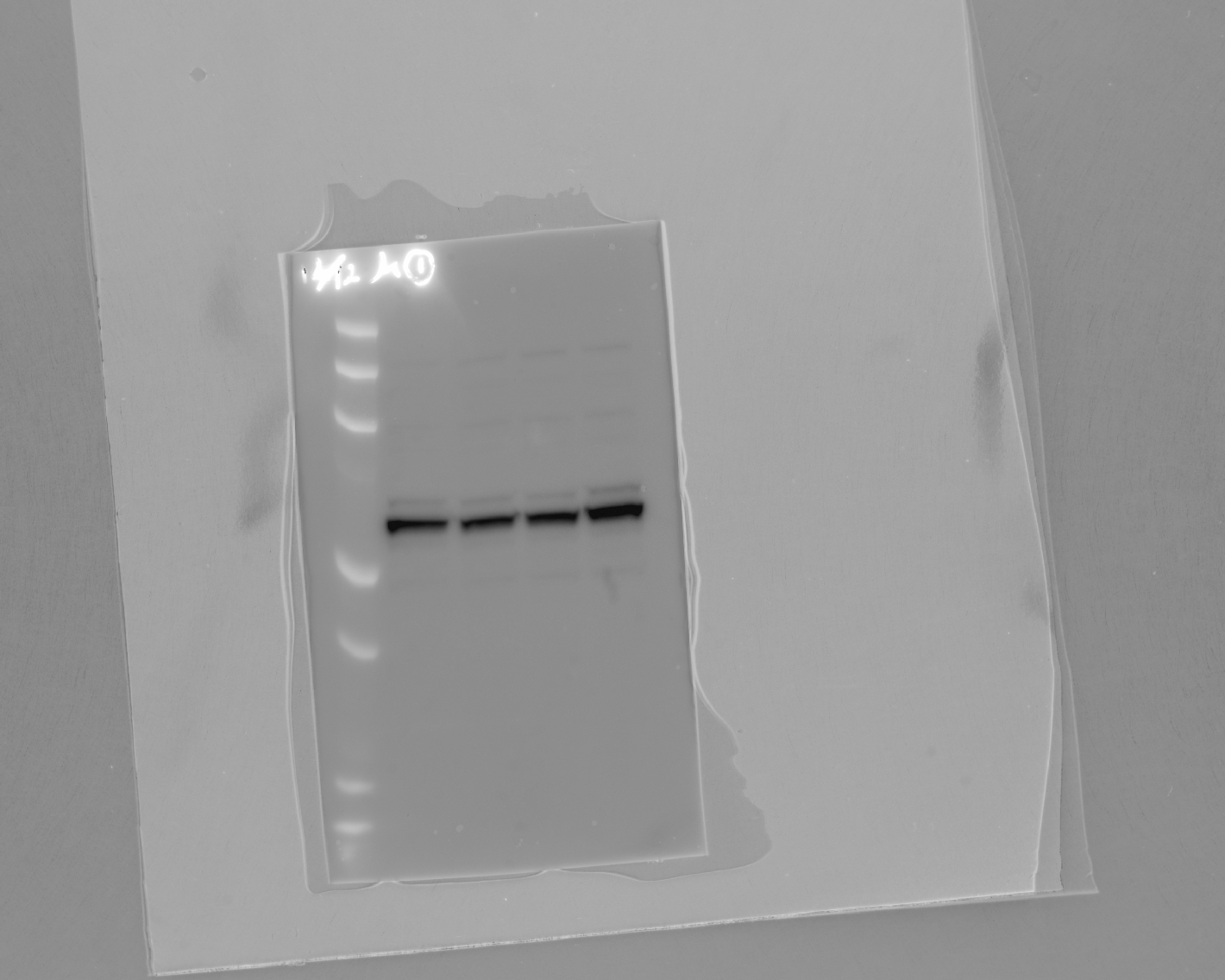

Supplement: Multimedia component 1 [file mmc1.zip › WB bands & raw densitometry/WB bands(24h)/9.P-70s6k/1.P-s6k(Composite).tif]

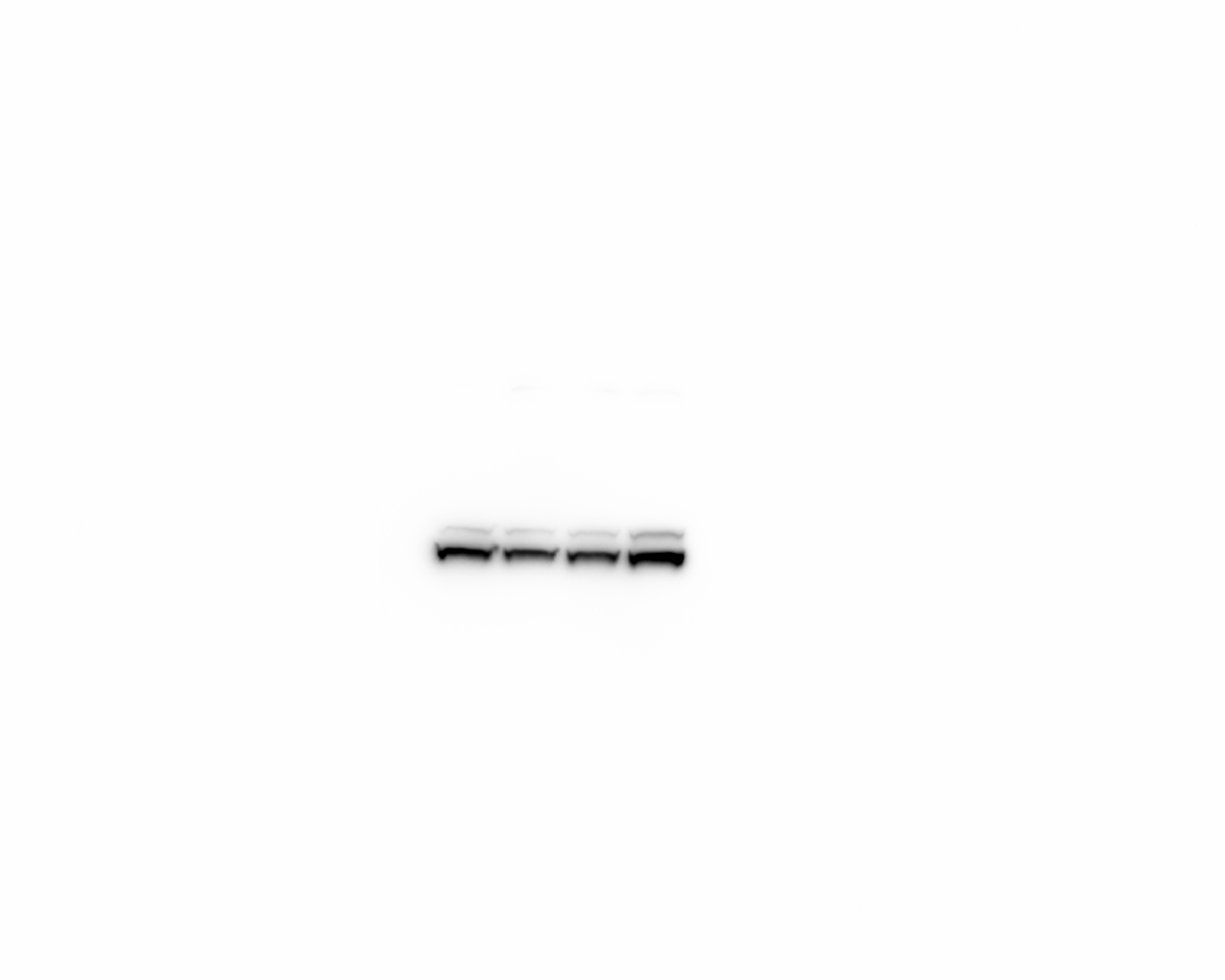

Supplement: Multimedia component 1 [file mmc1.zip › WB bands & raw densitometry/WB bands(24h)/9.P-70s6k/2.P-s6k(Chemiluminescence).tif]

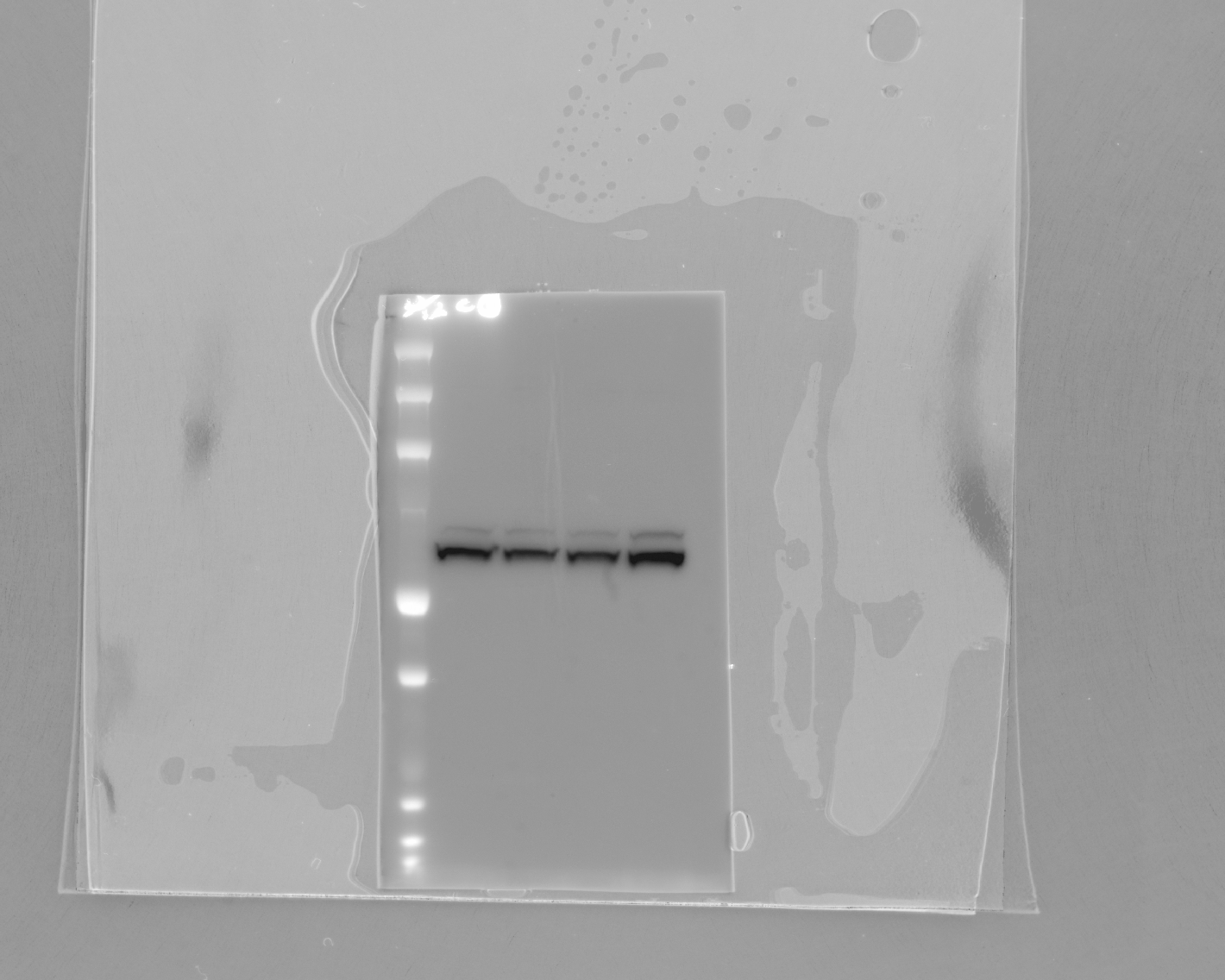

Supplement: Multimedia component 1 [file mmc1.zip › WB bands & raw densitometry/WB bands(24h)/9.P-70s6k/2.P-s6k(Composite).tif]

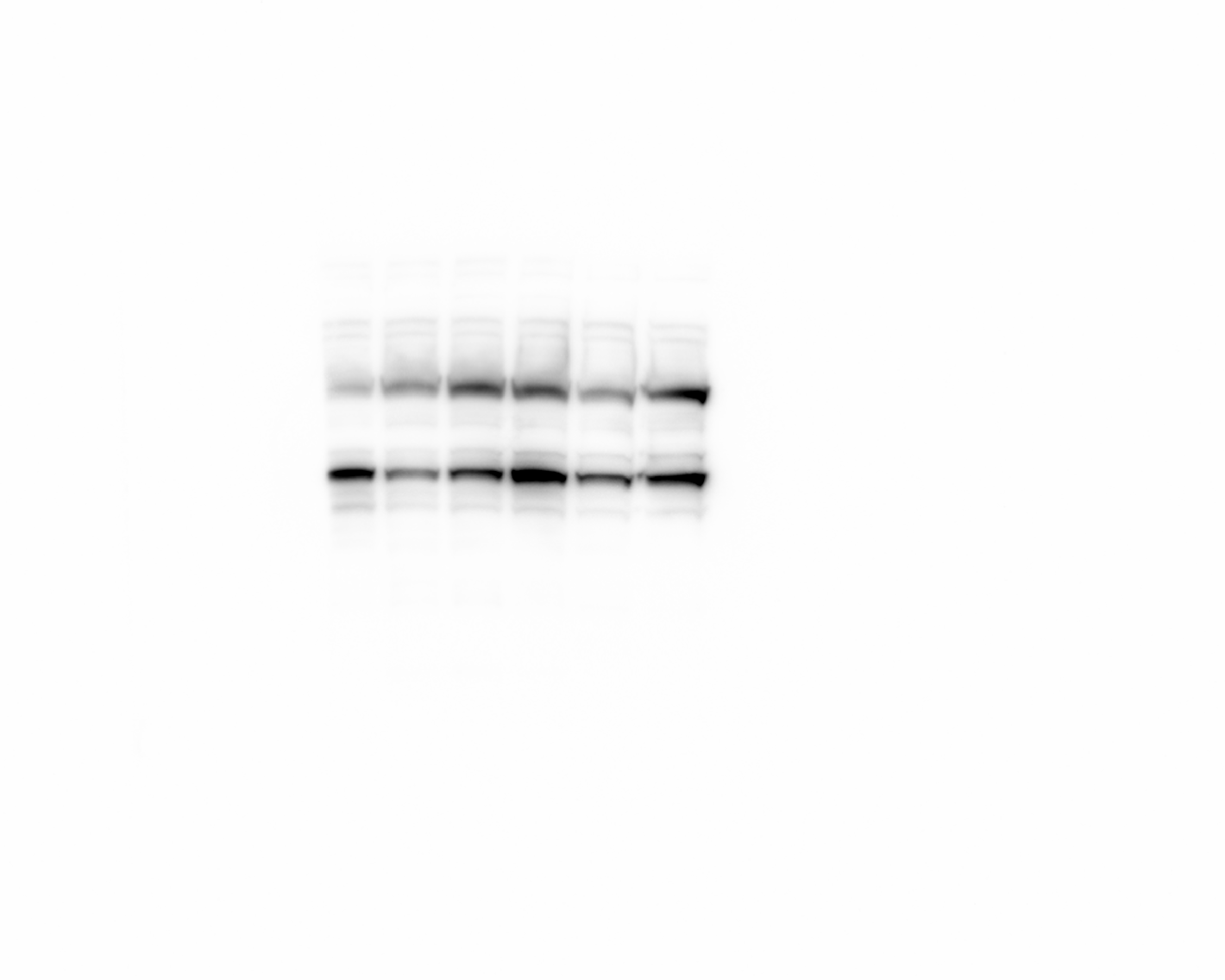

Supplement: Multimedia component 1 [file mmc1.zip › WB bands & raw densitometry/WB bands(24h)/9.P-70s6k/3.P-s6k(Chemiluminescence).tif]

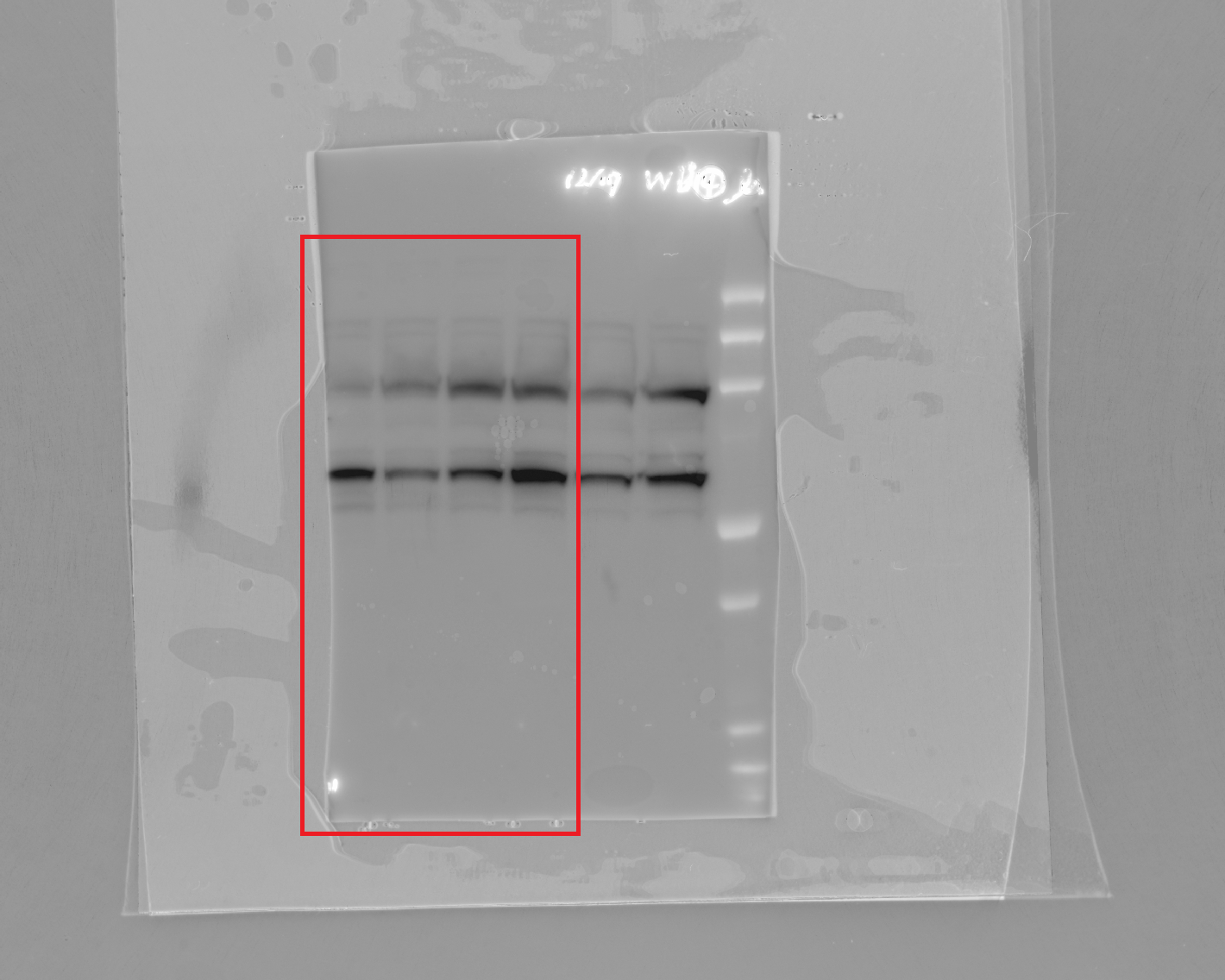

Supplement: Multimedia component 1 [file mmc1.zip › WB bands & raw densitometry/WB bands(24h)/9.P-70s6k/3.P-s6k(Composite).tif]

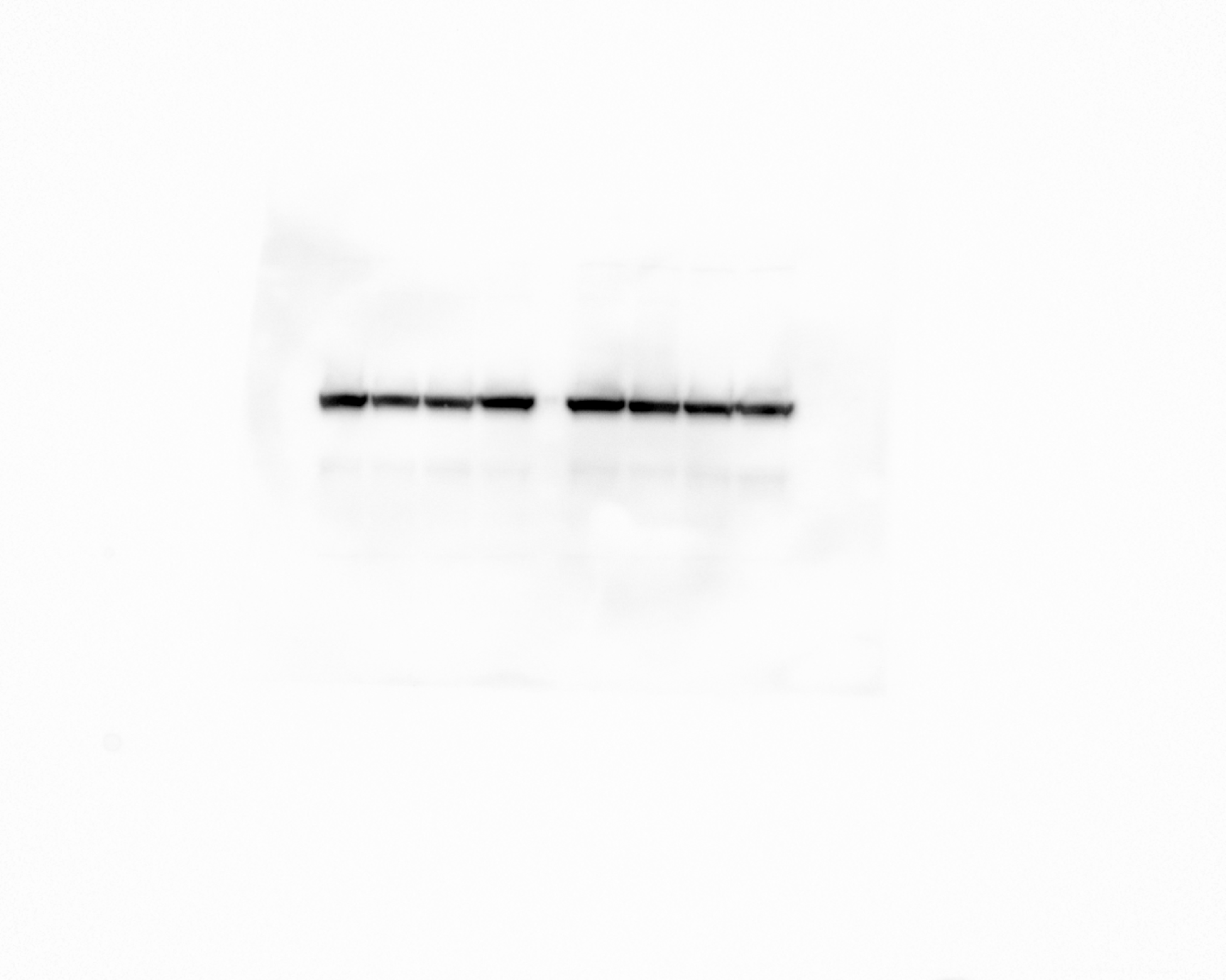

Supplement: Multimedia component 1 [file mmc1.zip › WB bands & raw densitometry/WB bands(45min)/1.(P-)AKT/AKT(1) com(Chemiluminescence).tif]

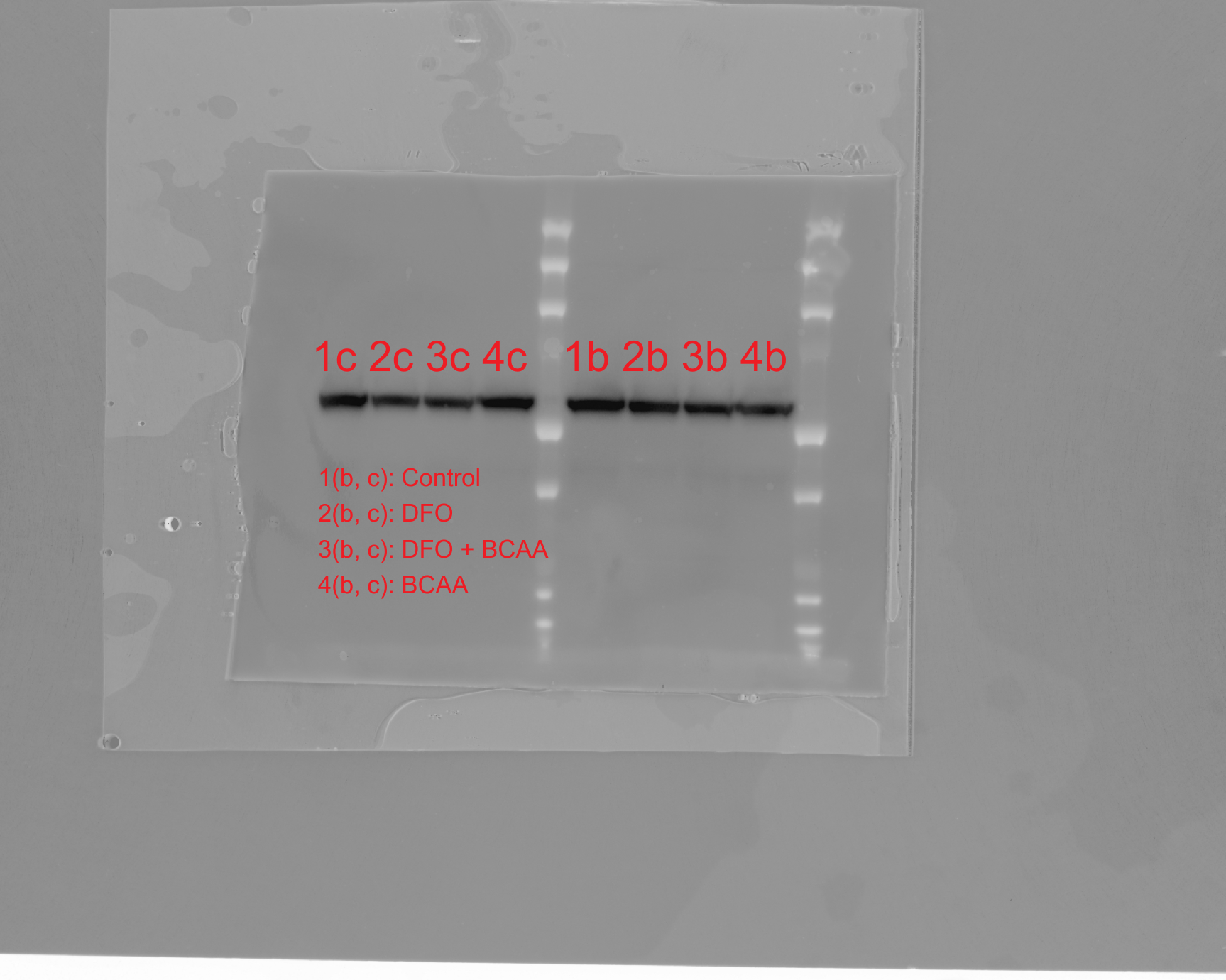

Supplement: Multimedia component 1 [file mmc1.zip › WB bands & raw densitometry/WB bands(45min)/1.(P-)AKT/AKT(1) com(Composite).tif]

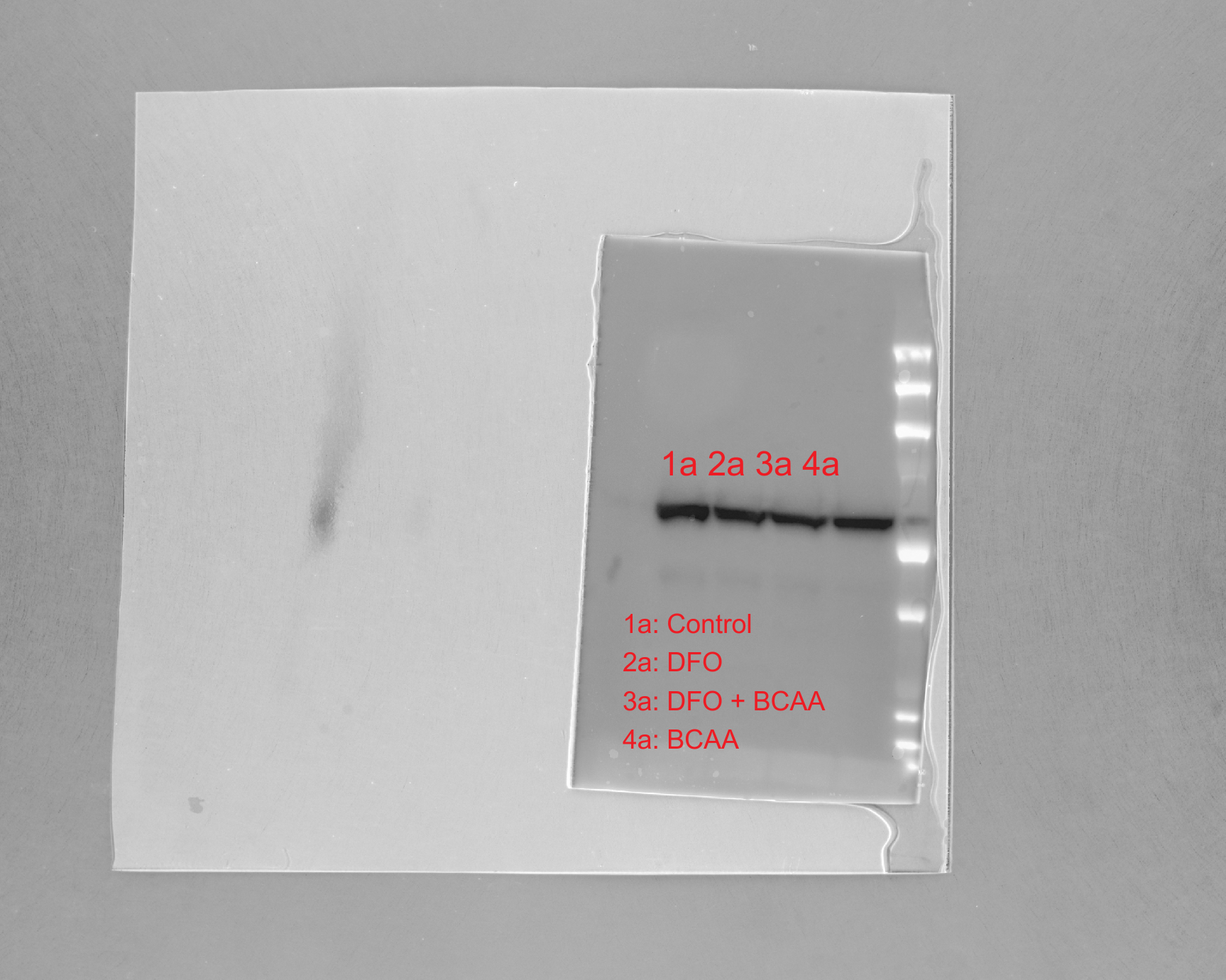

Supplement: Multimedia component 1 [file mmc1.zip › WB bands & raw densitometry/WB bands(45min)/1.(P-)AKT/AKT(2) com(Composite).tif]

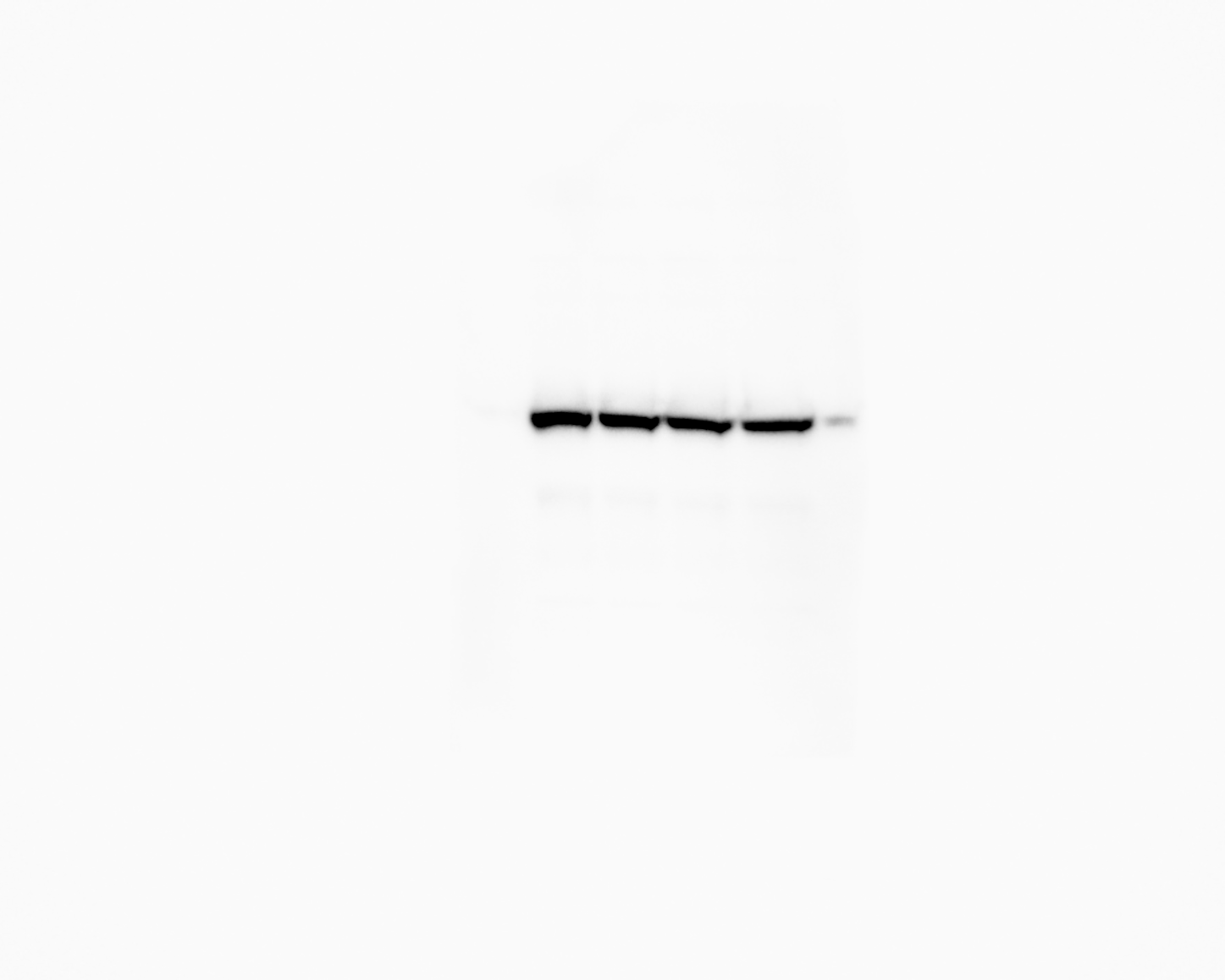

Supplement: Multimedia component 1 [file mmc1.zip › WB bands & raw densitometry/WB bands(45min)/1.(P-)AKT/AKT(2)(Chemiluminescence).tif]

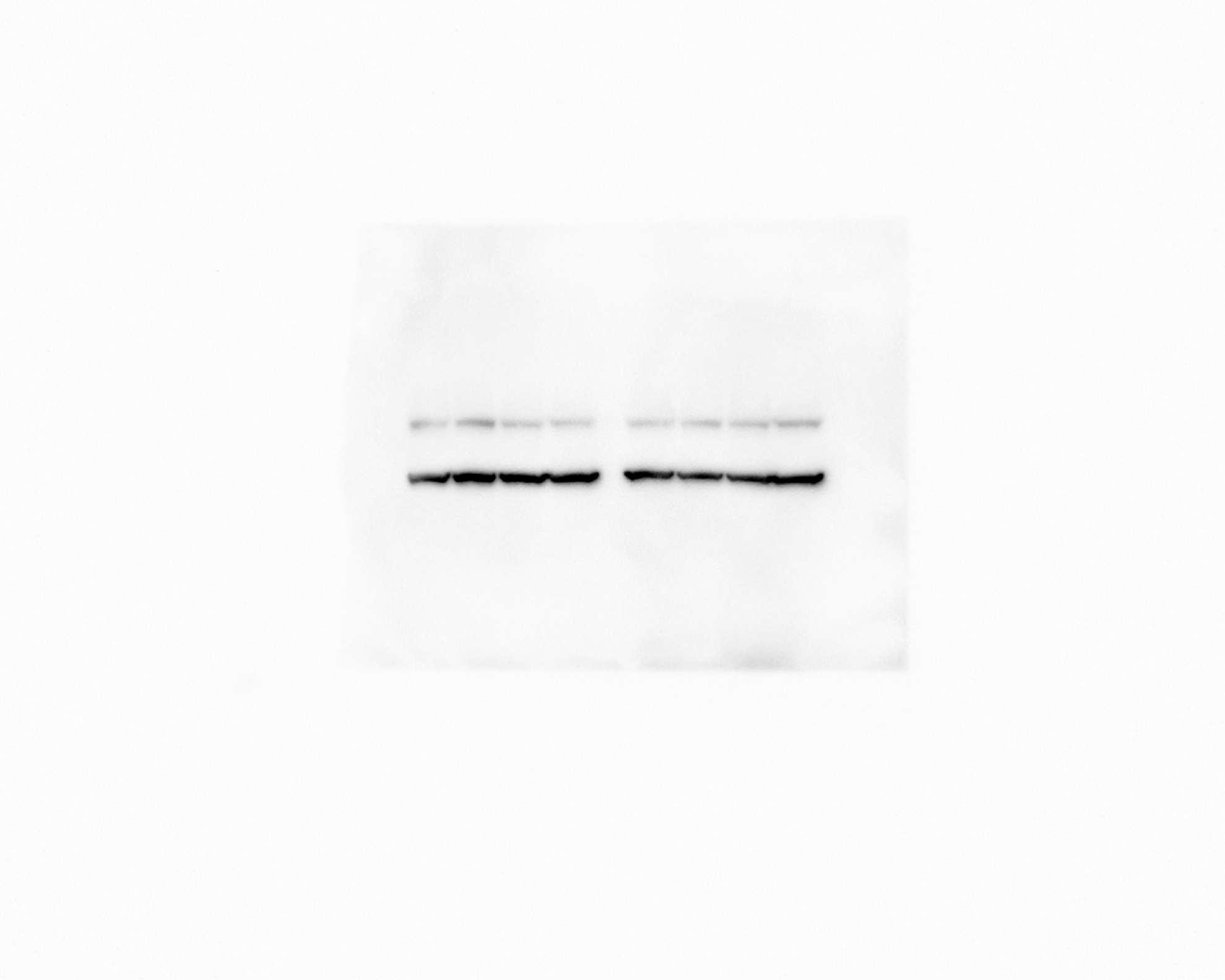

Supplement: Multimedia component 1 [file mmc1.zip › WB bands & raw densitometry/WB bands(45min)/1.(P-)AKT/B-actin(1)(Chemiluminescence).tif]

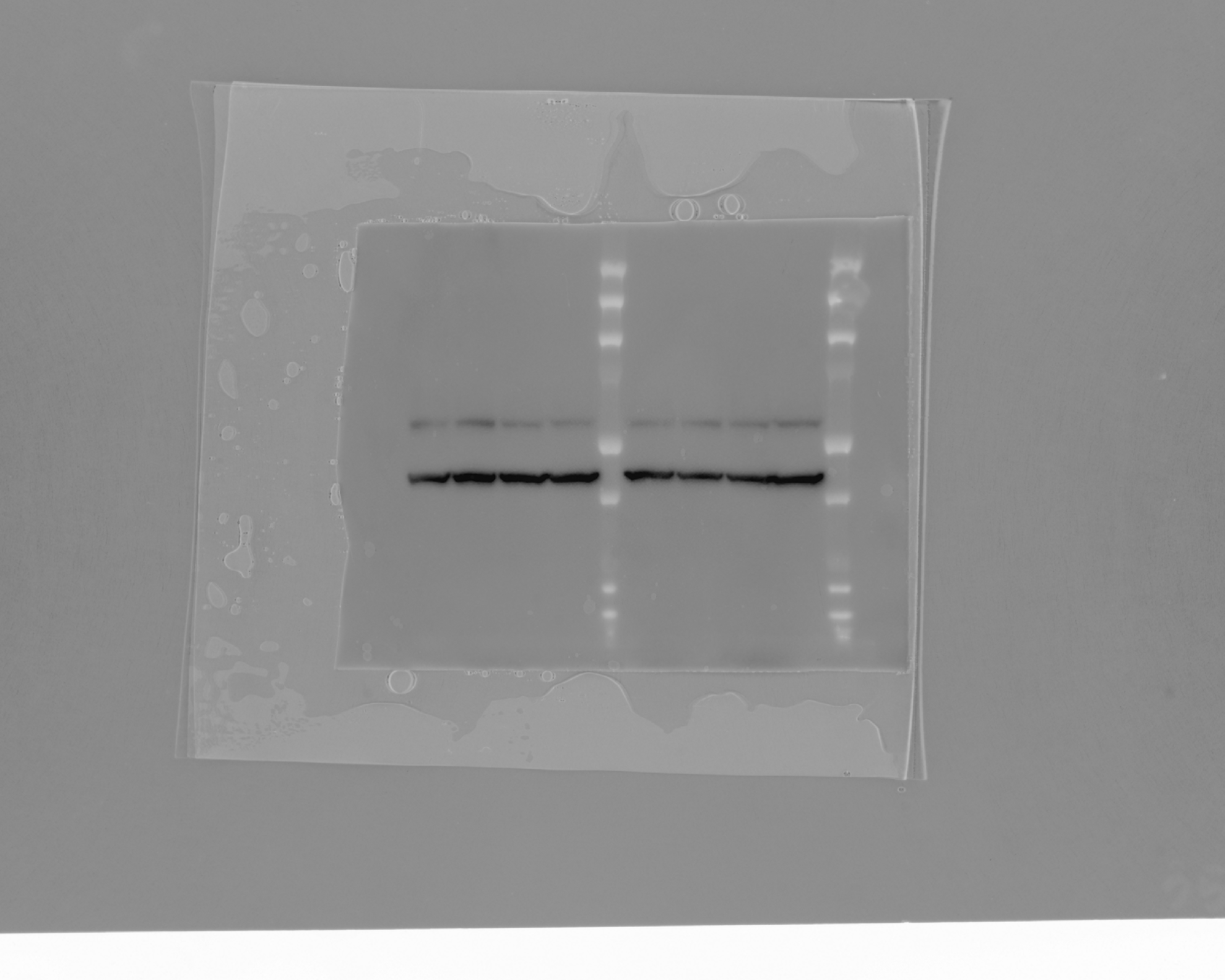

Supplement: Multimedia component 1 [file mmc1.zip › WB bands & raw densitometry/WB bands(45min)/1.(P-)AKT/B-actin(1)(Composite).tif]

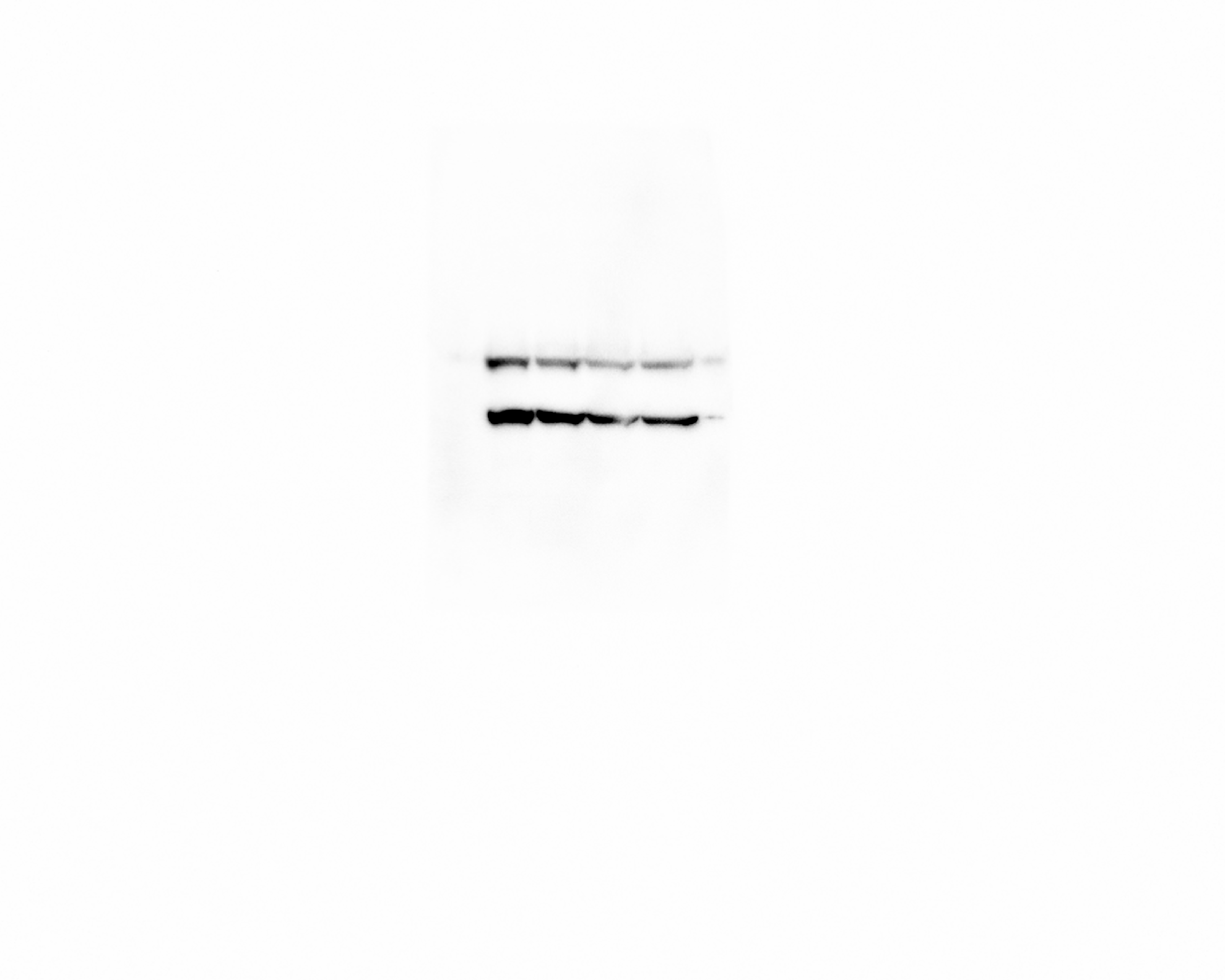

Supplement: Multimedia component 1 [file mmc1.zip › WB bands & raw densitometry/WB bands(45min)/1.(P-)AKT/B-actin(Chemiluminescence).tif]

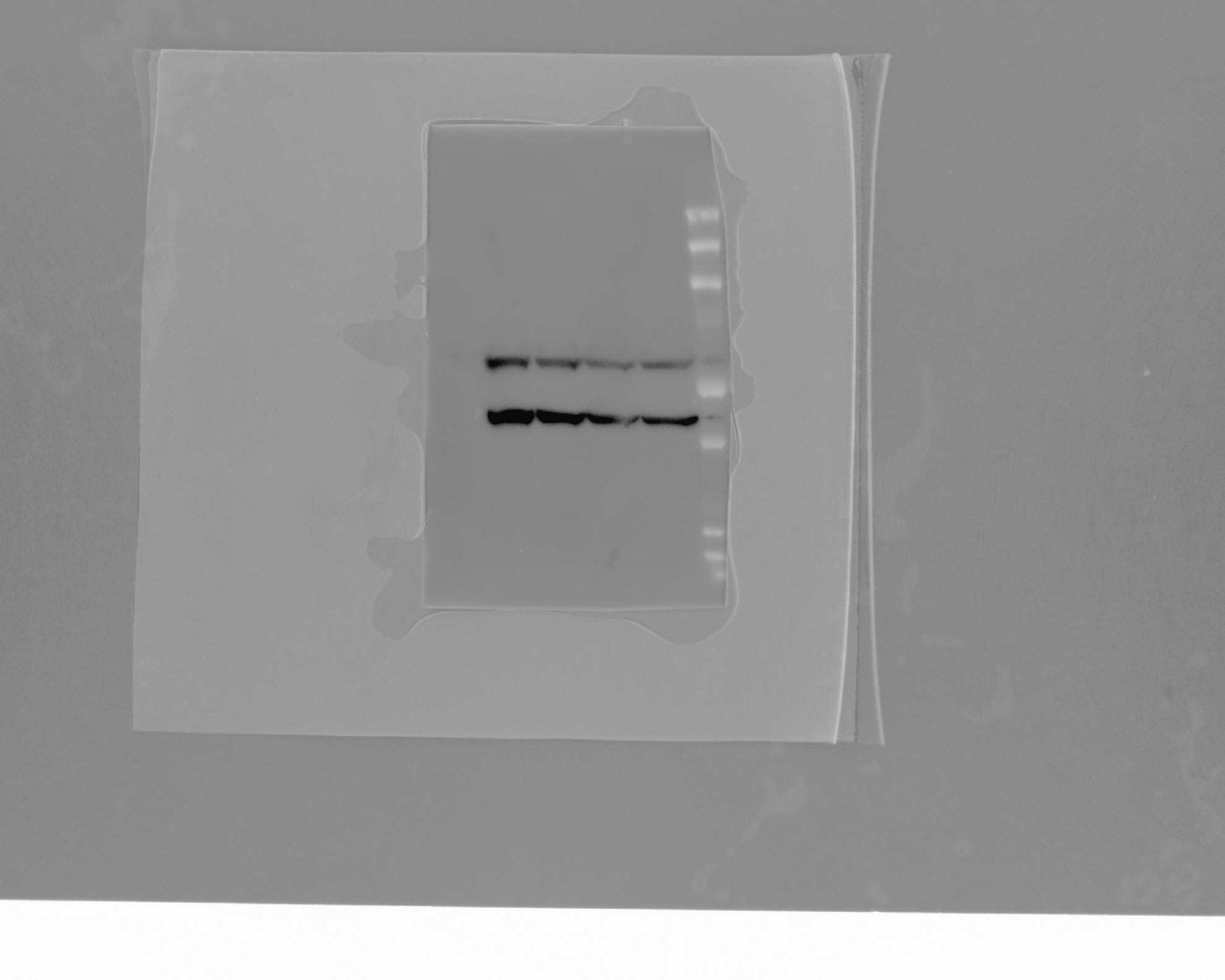

Supplement: Multimedia component 1 [file mmc1.zip › WB bands & raw densitometry/WB bands(45min)/1.(P-)AKT/B-actin(Composite).tif]

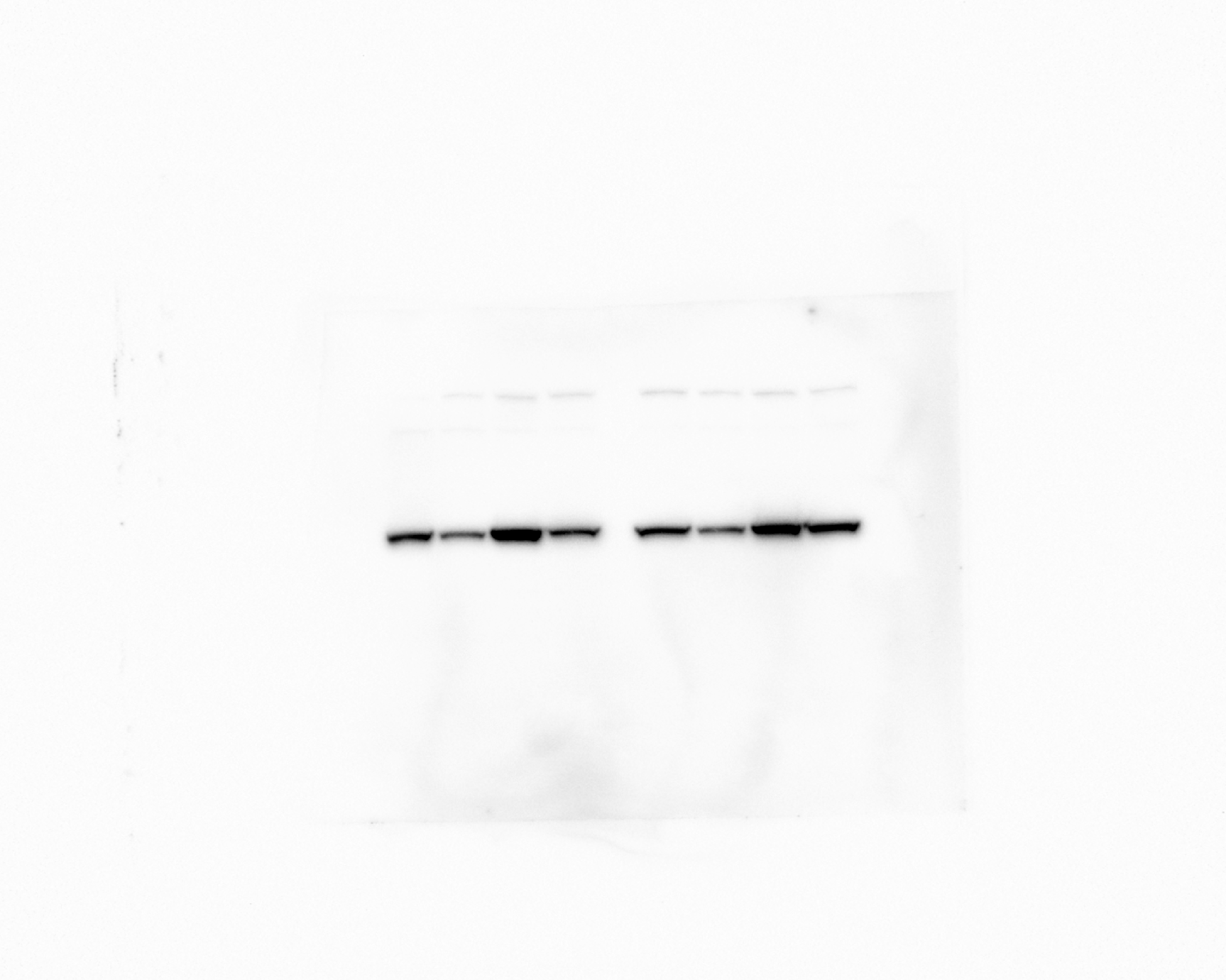

Supplement: Multimedia component 1 [file mmc1.zip › WB bands & raw densitometry/WB bands(45min)/1.(P-)AKT/p-Akt(1) composite(Chemiluminescence).tif]

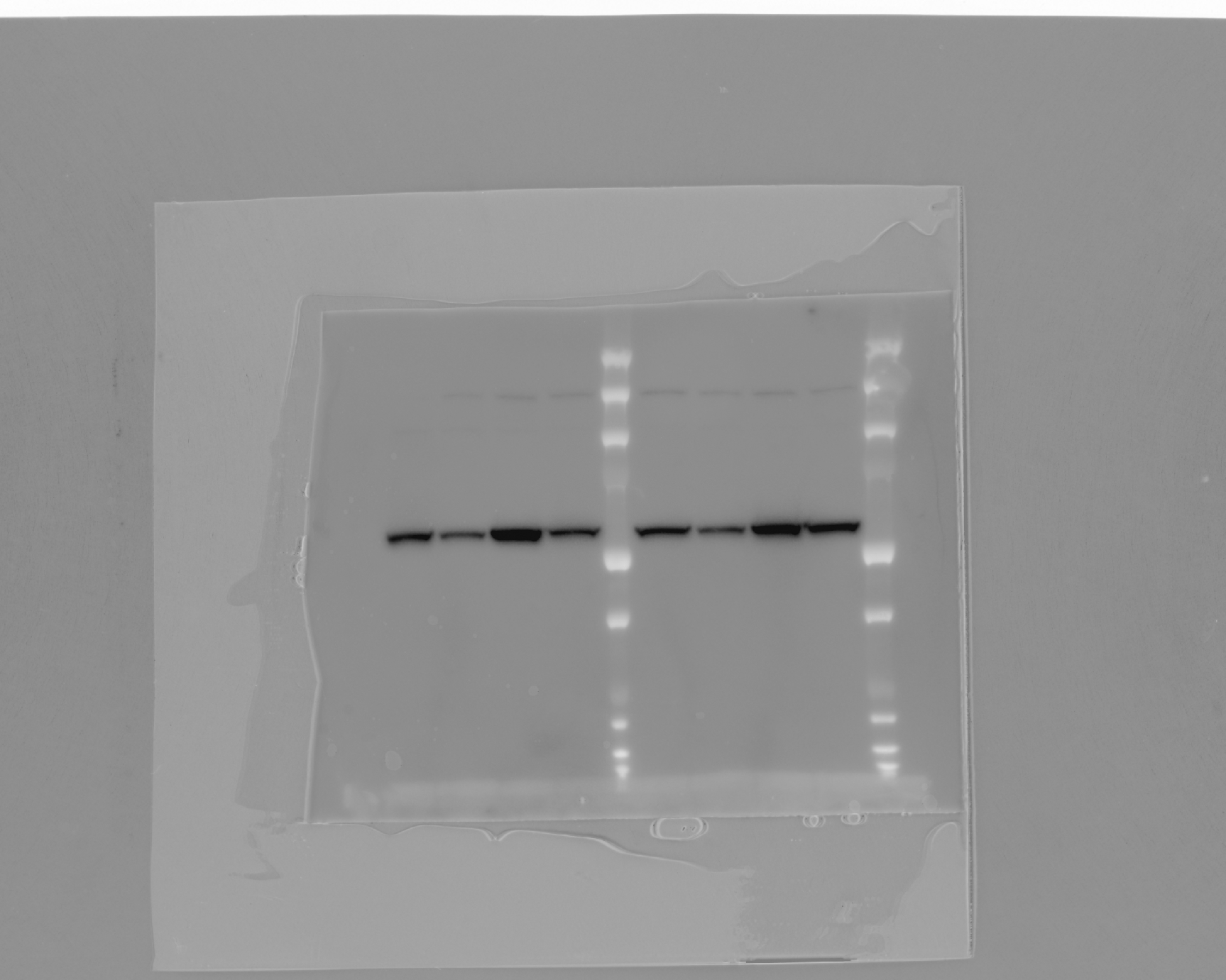

Supplement: Multimedia component 1 [file mmc1.zip › WB bands & raw densitometry/WB bands(45min)/1.(P-)AKT/p-Akt(1) composite(Composite).tif]

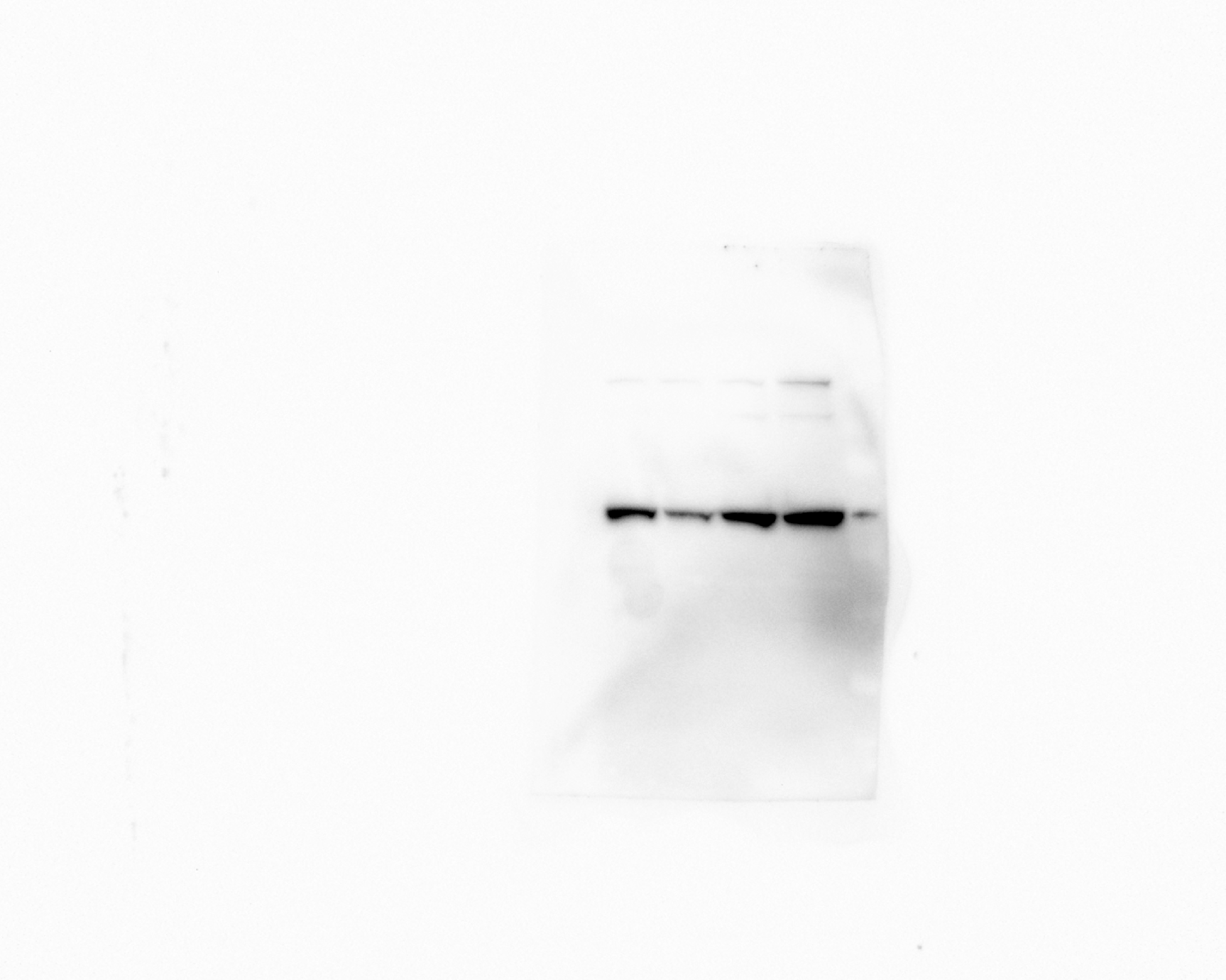

Supplement: Multimedia component 1 [file mmc1.zip › WB bands & raw densitometry/WB bands(45min)/1.(P-)AKT/p-Akt(2) composite(Chemiluminescence).tif]

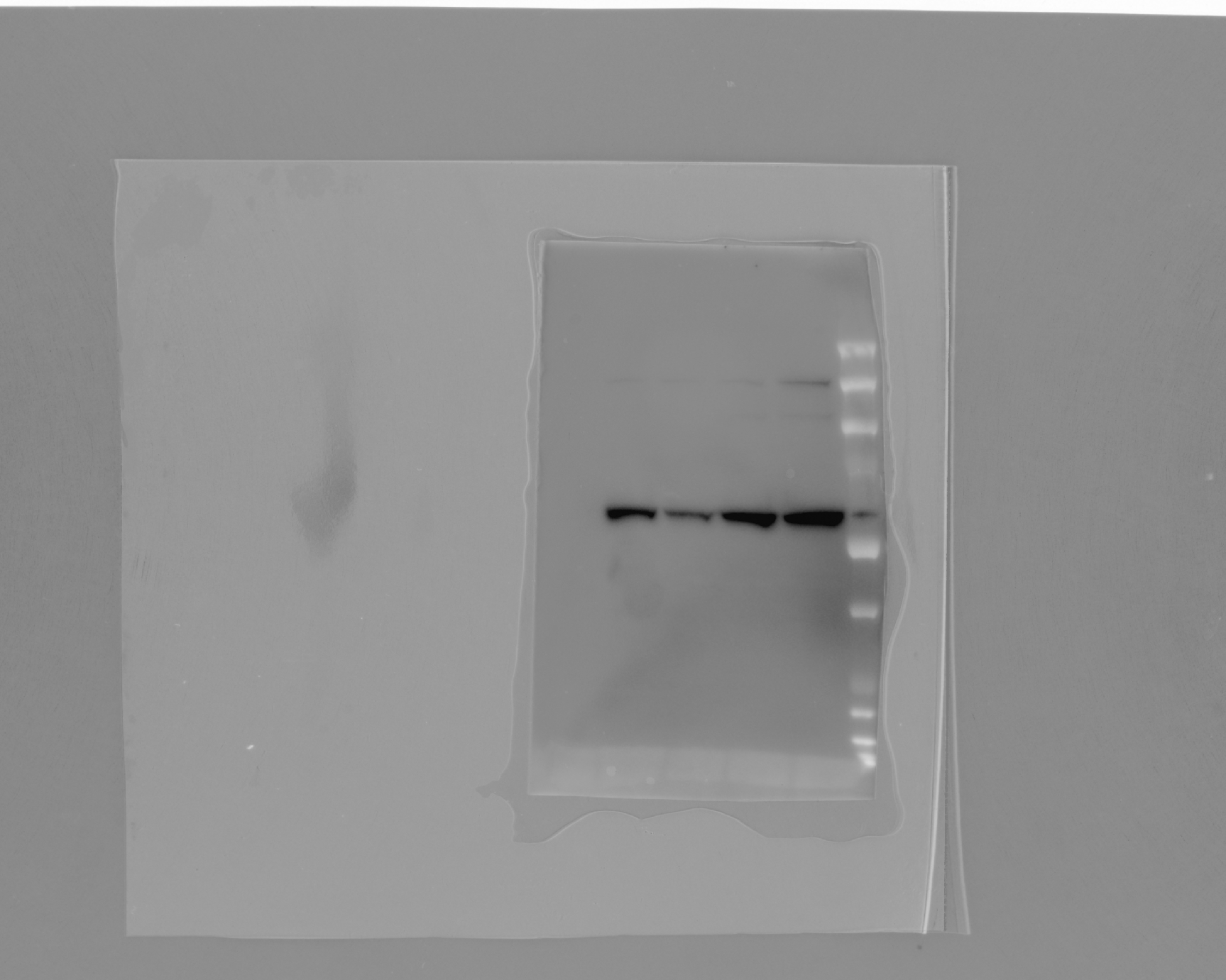

Supplement: Multimedia component 1 [file mmc1.zip › WB bands & raw densitometry/WB bands(45min)/1.(P-)AKT/p-Akt(2) composite(Composite).tif]

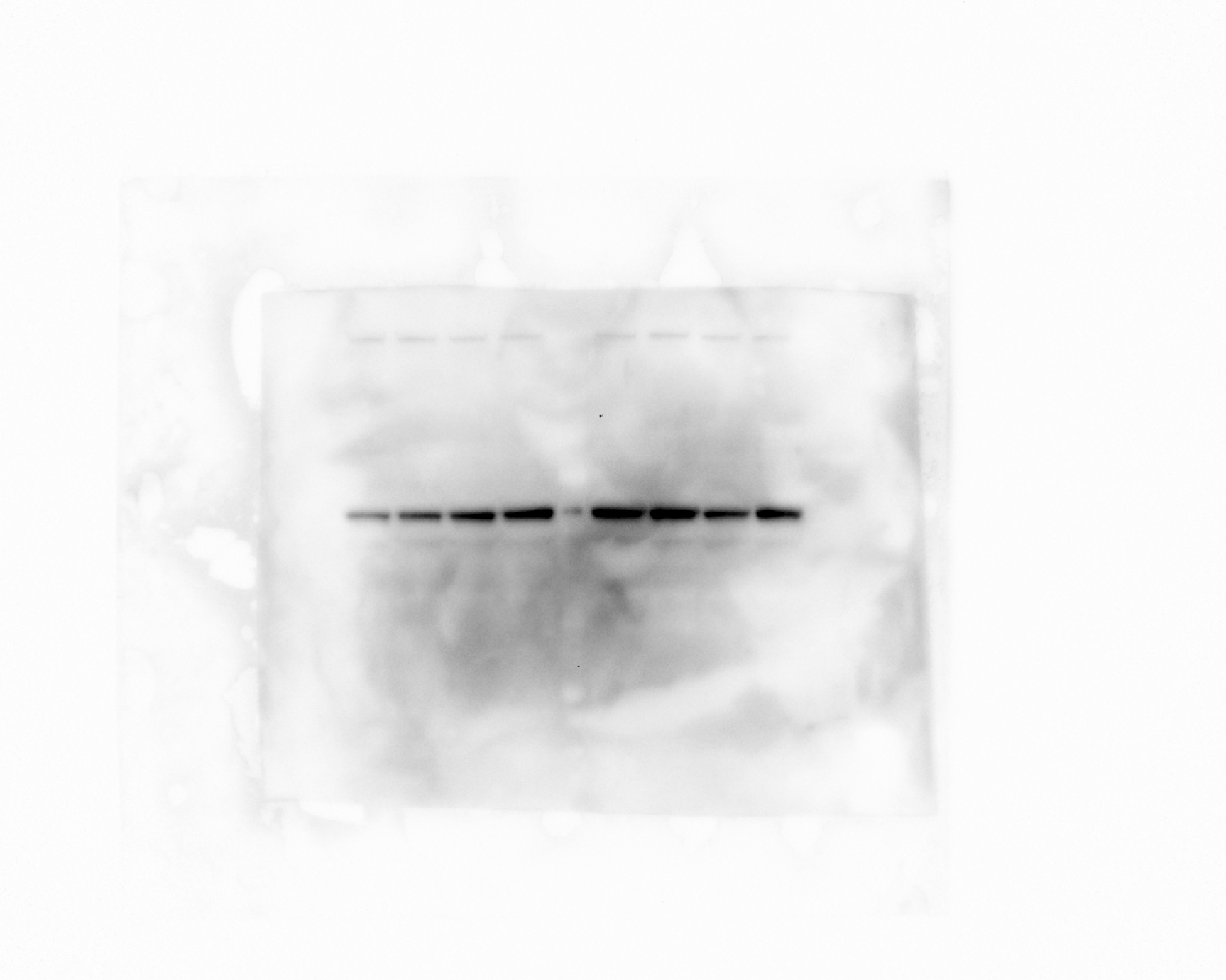

Supplement: Multimedia component 1 [file mmc1.zip › WB bands & raw densitometry/WB bands(45min)/1.(P-)AMPK/User 2025-09-15 p-AMPK(1)composite(Chemiluminescence).tif]

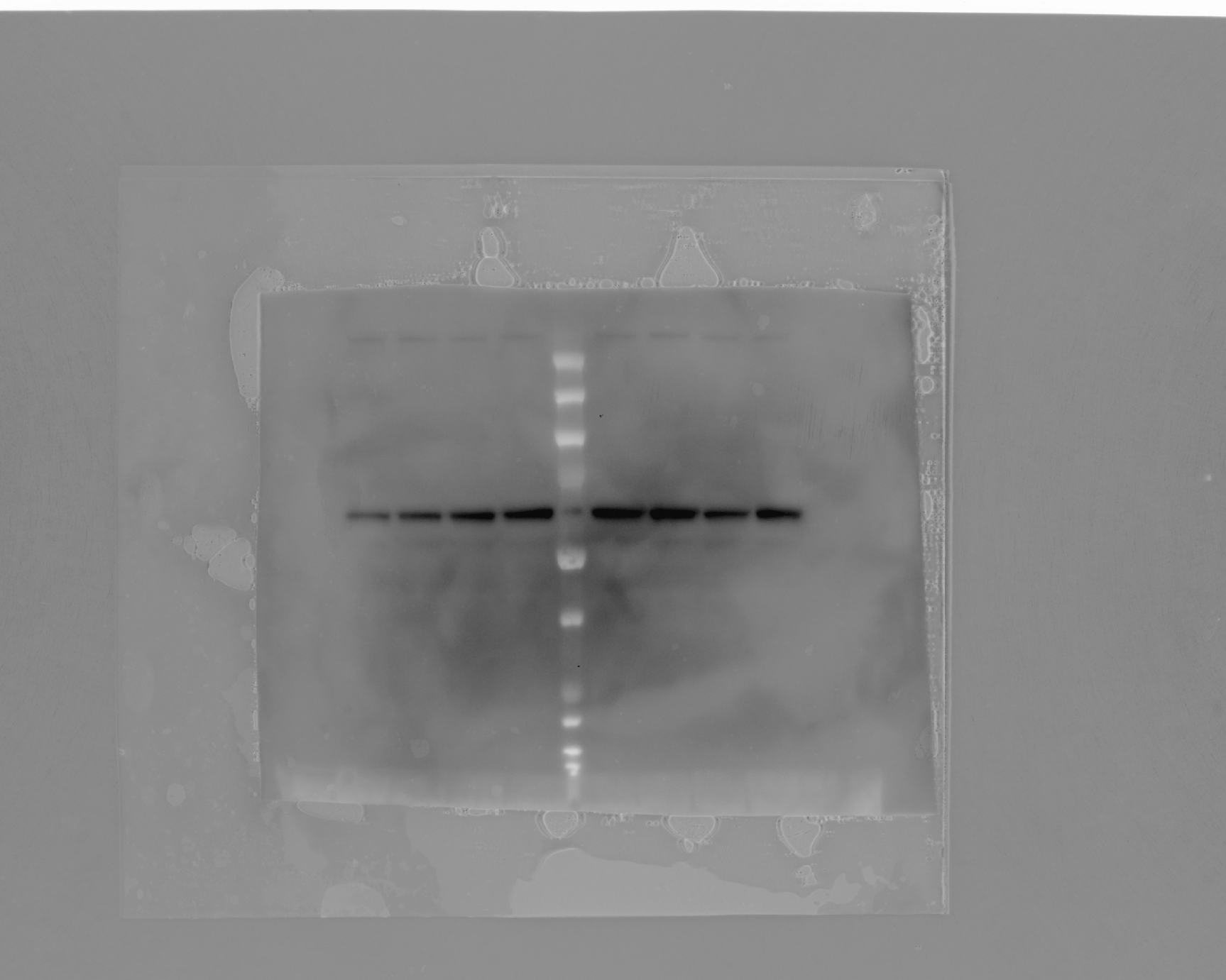

Supplement: Multimedia component 1 [file mmc1.zip › WB bands & raw densitometry/WB bands(45min)/1.(P-)AMPK/User 2025-09-15 p-AMPK(1)composite(Composite).tif]

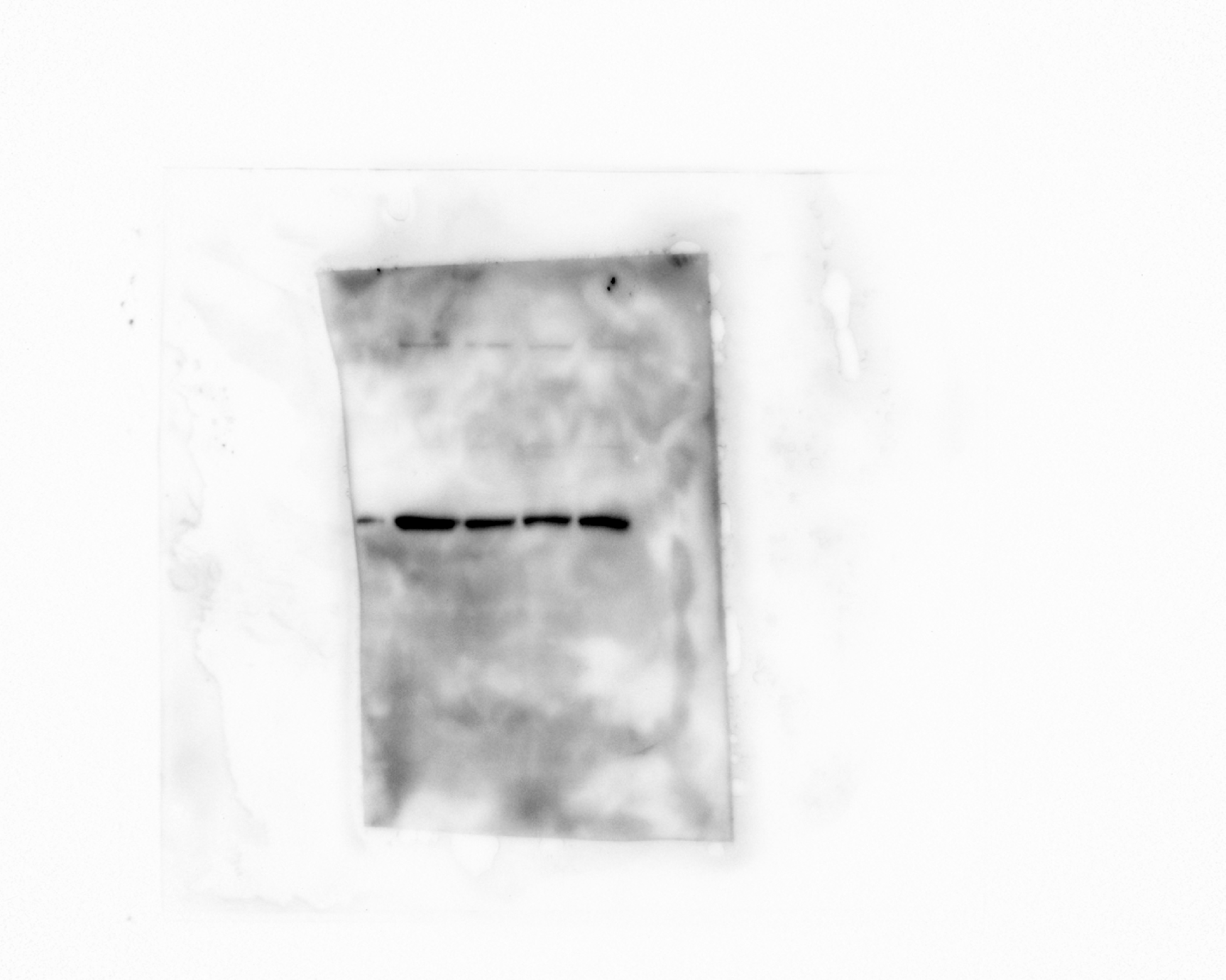

Supplement: Multimedia component 1 [file mmc1.zip › WB bands & raw densitometry/WB bands(45min)/1.(P-)AMPK/User 2025-09-15 p-AMPK(2)composite(Chemiluminescence).tif]

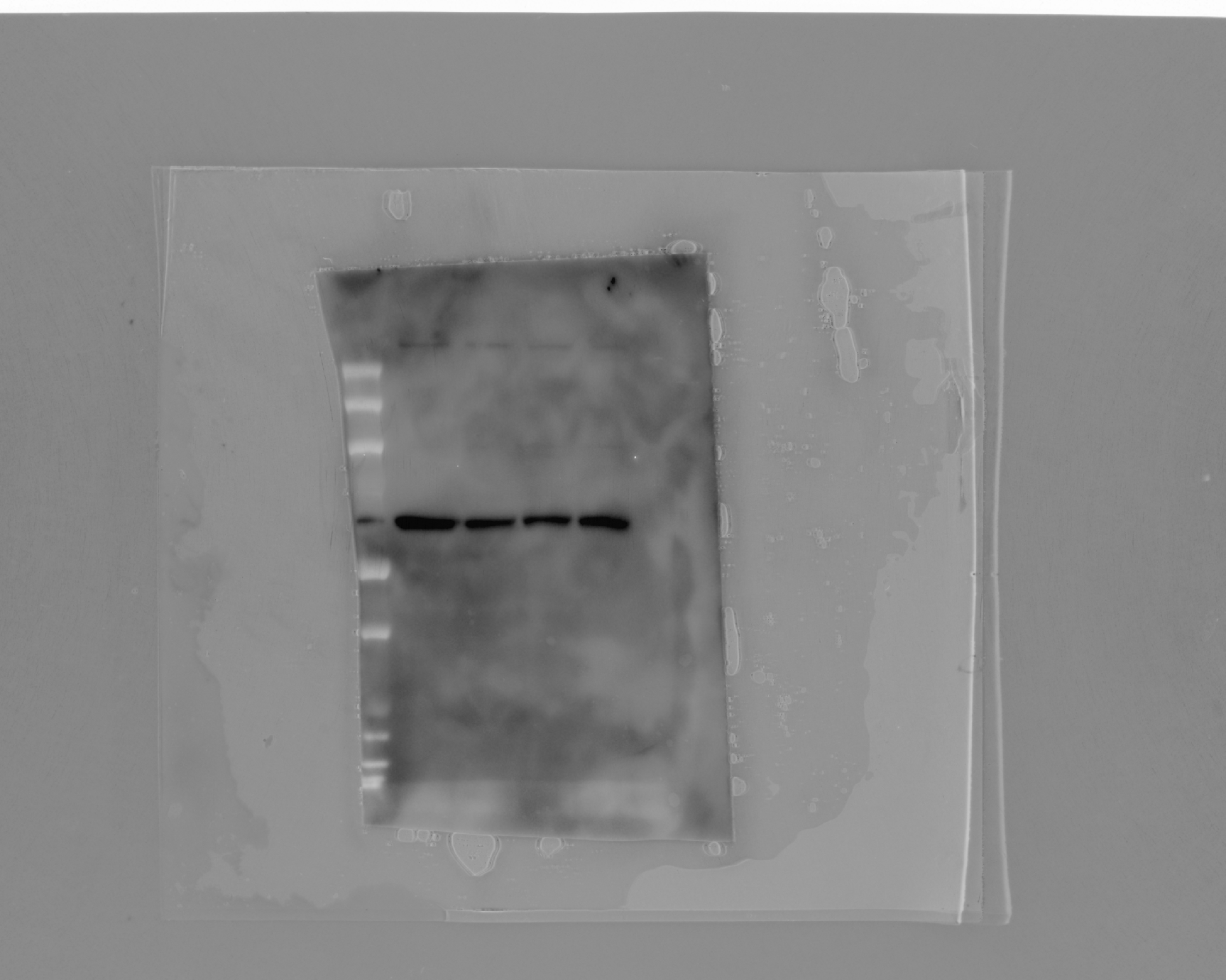

Supplement: Multimedia component 1 [file mmc1.zip › WB bands & raw densitometry/WB bands(45min)/1.(P-)AMPK/User 2025-09-15 p-AMPK(2)composite(Composite).tif]

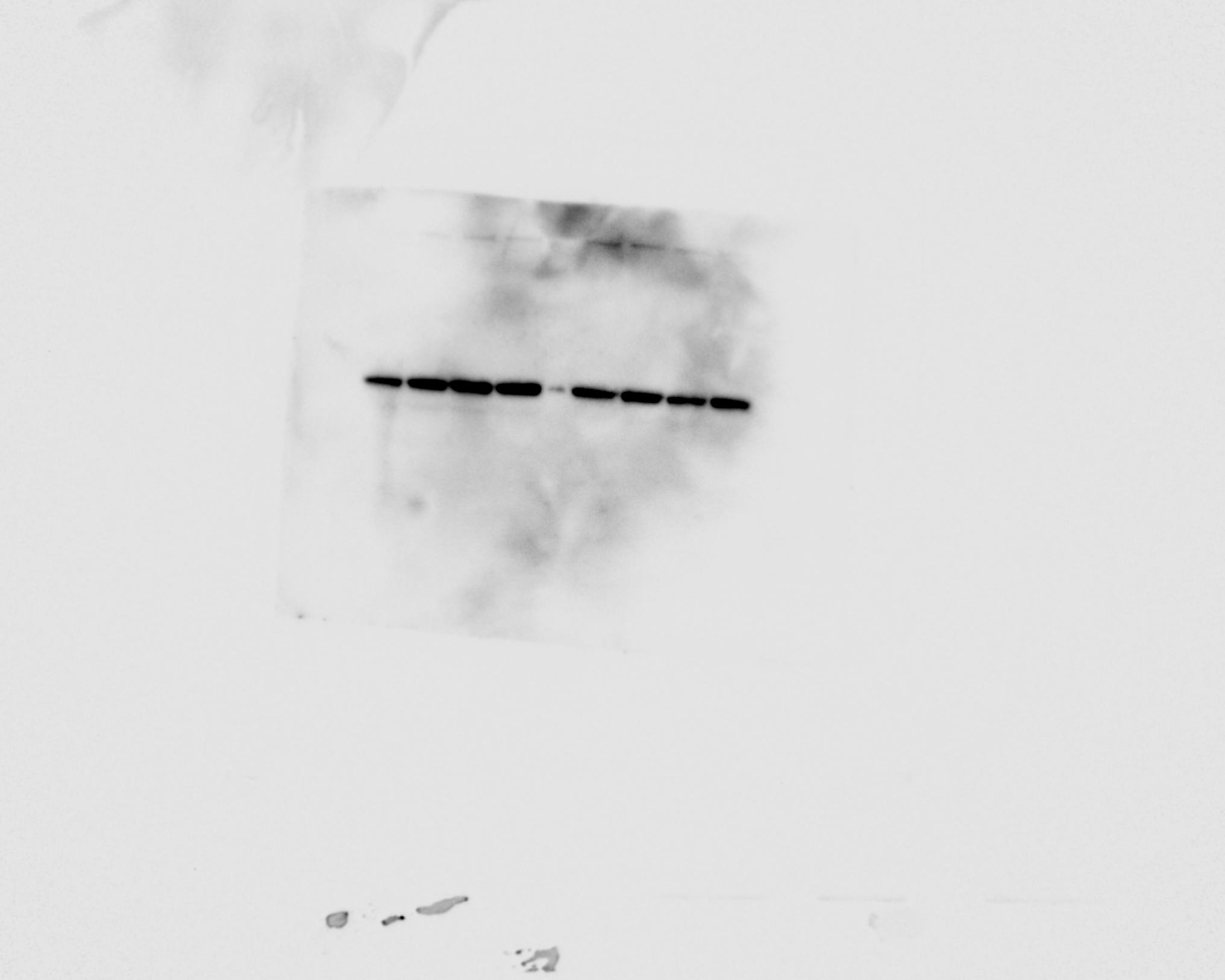

Supplement: Multimedia component 1 [file mmc1.zip › WB bands & raw densitometry/WB bands(45min)/1.(P-)AMPK/User 2025-09-16 45min AMPK(1)(Chemiluminescence).tif]

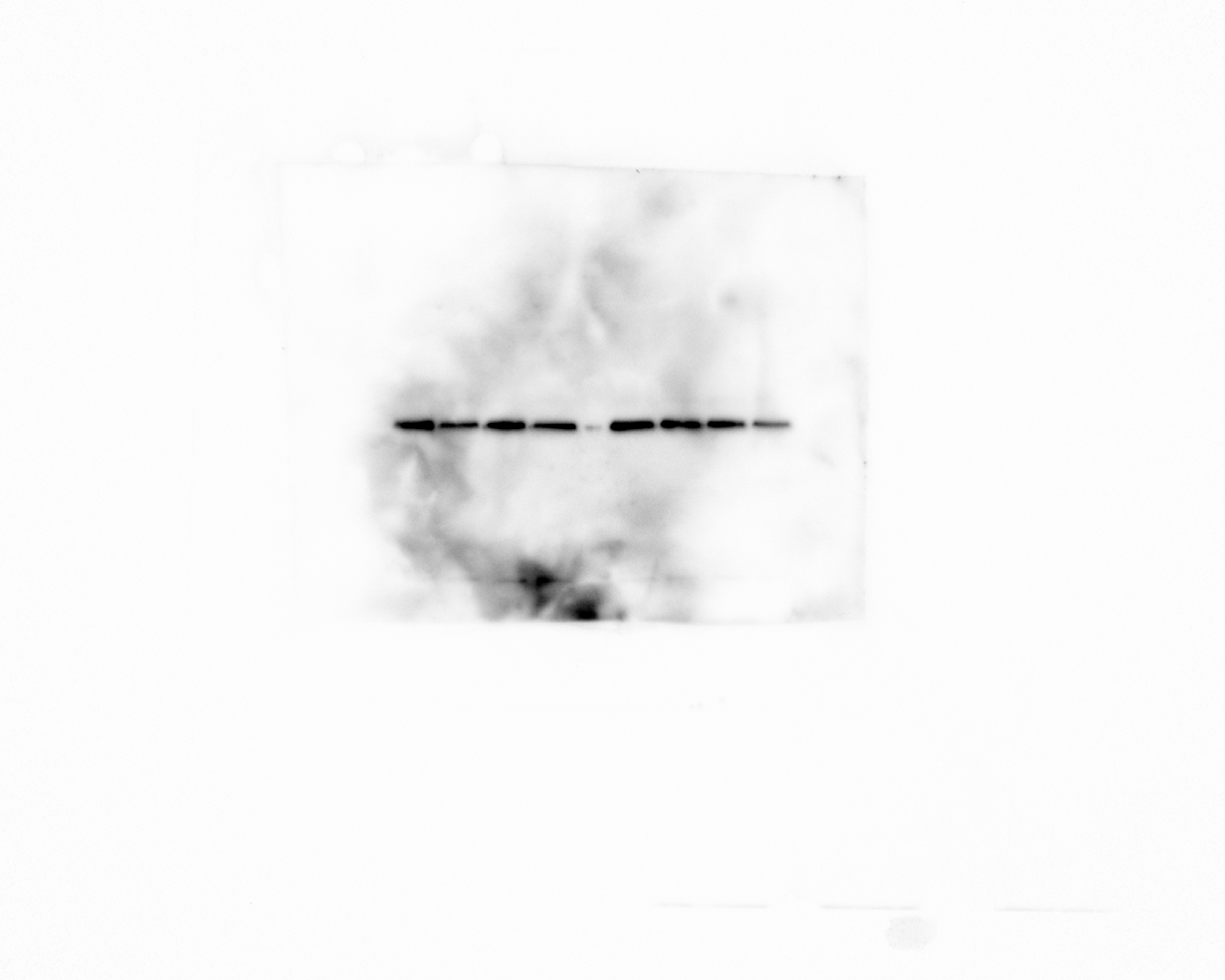

Supplement: Multimedia component 1 [file mmc1.zip › WB bands & raw densitometry/WB bands(45min)/1.(P-)AMPK/User 2025-09-16 45min AMPK(1)con(Chemiluminescence).tif]

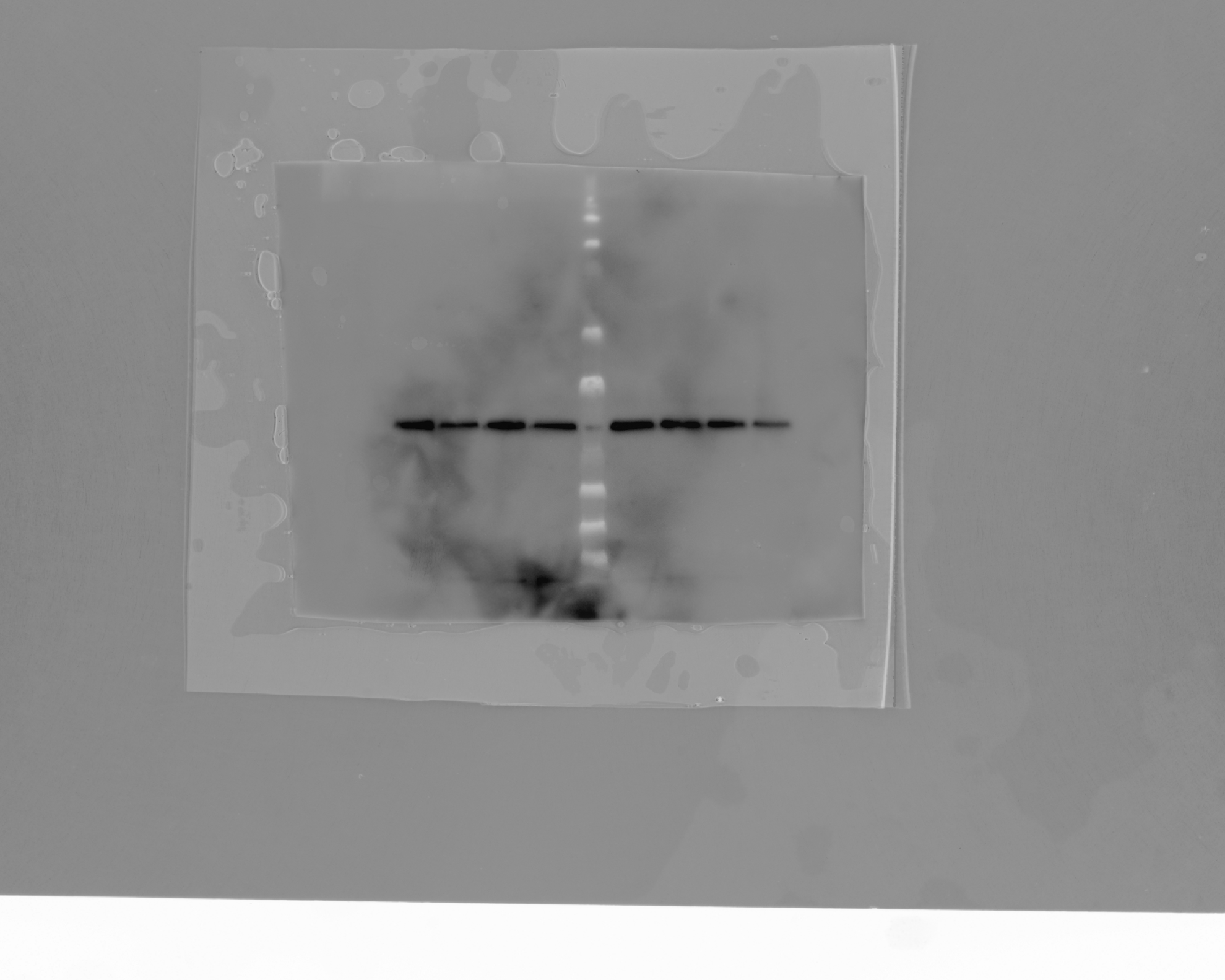

Supplement: Multimedia component 1 [file mmc1.zip › WB bands & raw densitometry/WB bands(45min)/1.(P-)AMPK/User 2025-09-16 45min AMPK(1)con(Composite).tif]

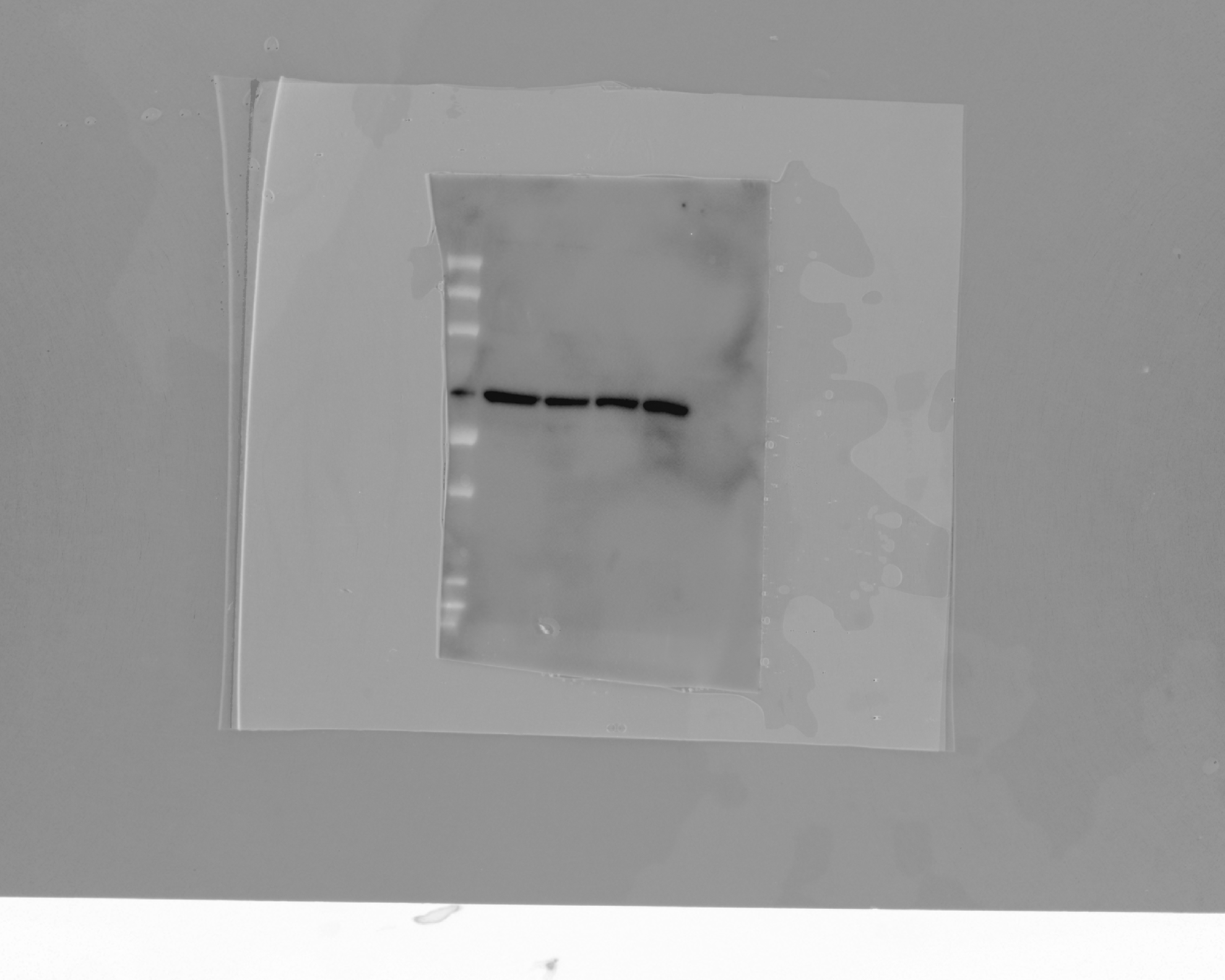

Supplement: Multimedia component 1 [file mmc1.zip › WB bands & raw densitometry/WB bands(45min)/1.(P-)AMPK/User 2025-09-16 45min AMPK(2) con(Composite).tif]

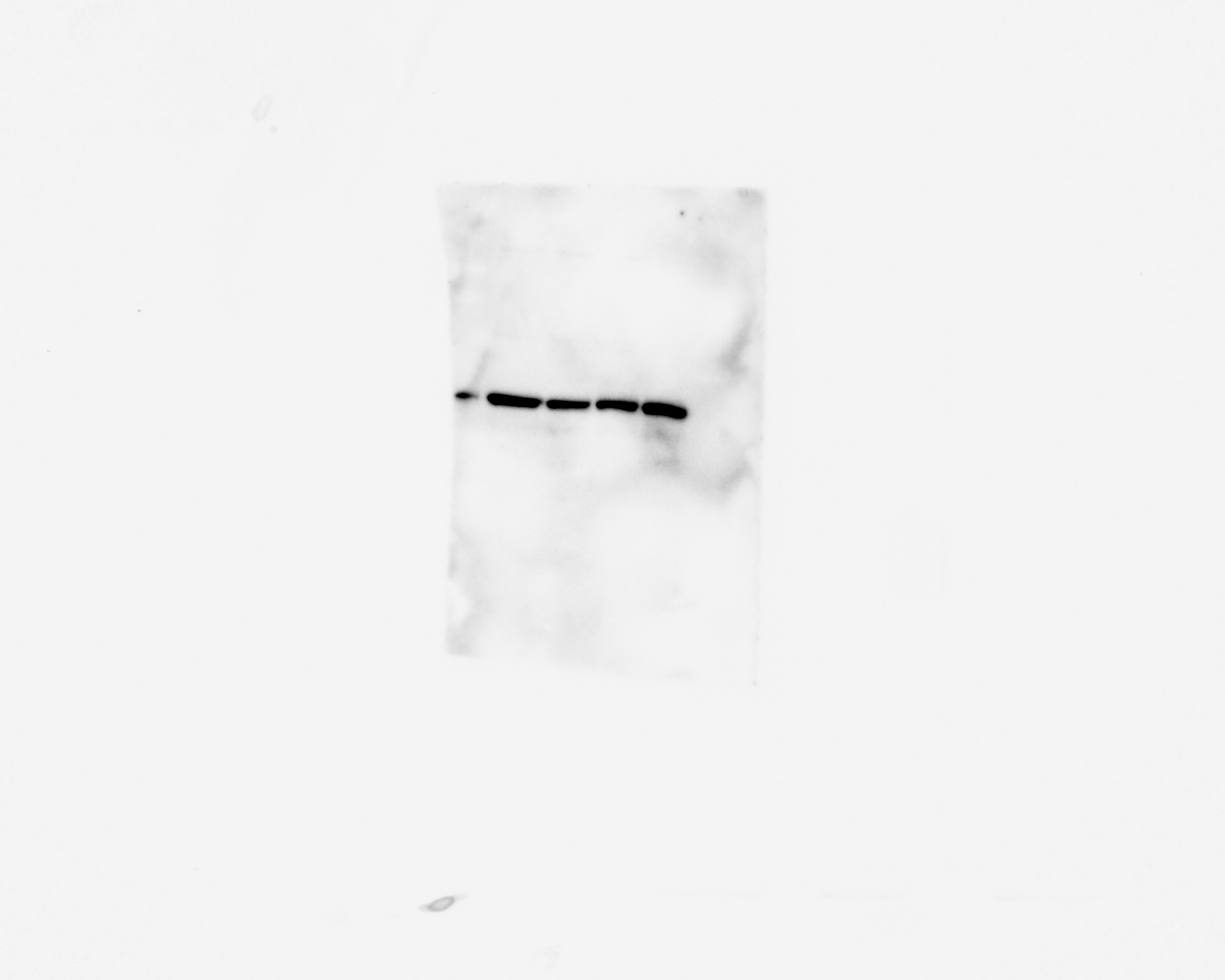

Supplement: Multimedia component 1 [file mmc1.zip › WB bands & raw densitometry/WB bands(45min)/1.(P-)AMPK/User 2025-09-16 45min AMPK(2)(Chemiluminescence).tif]

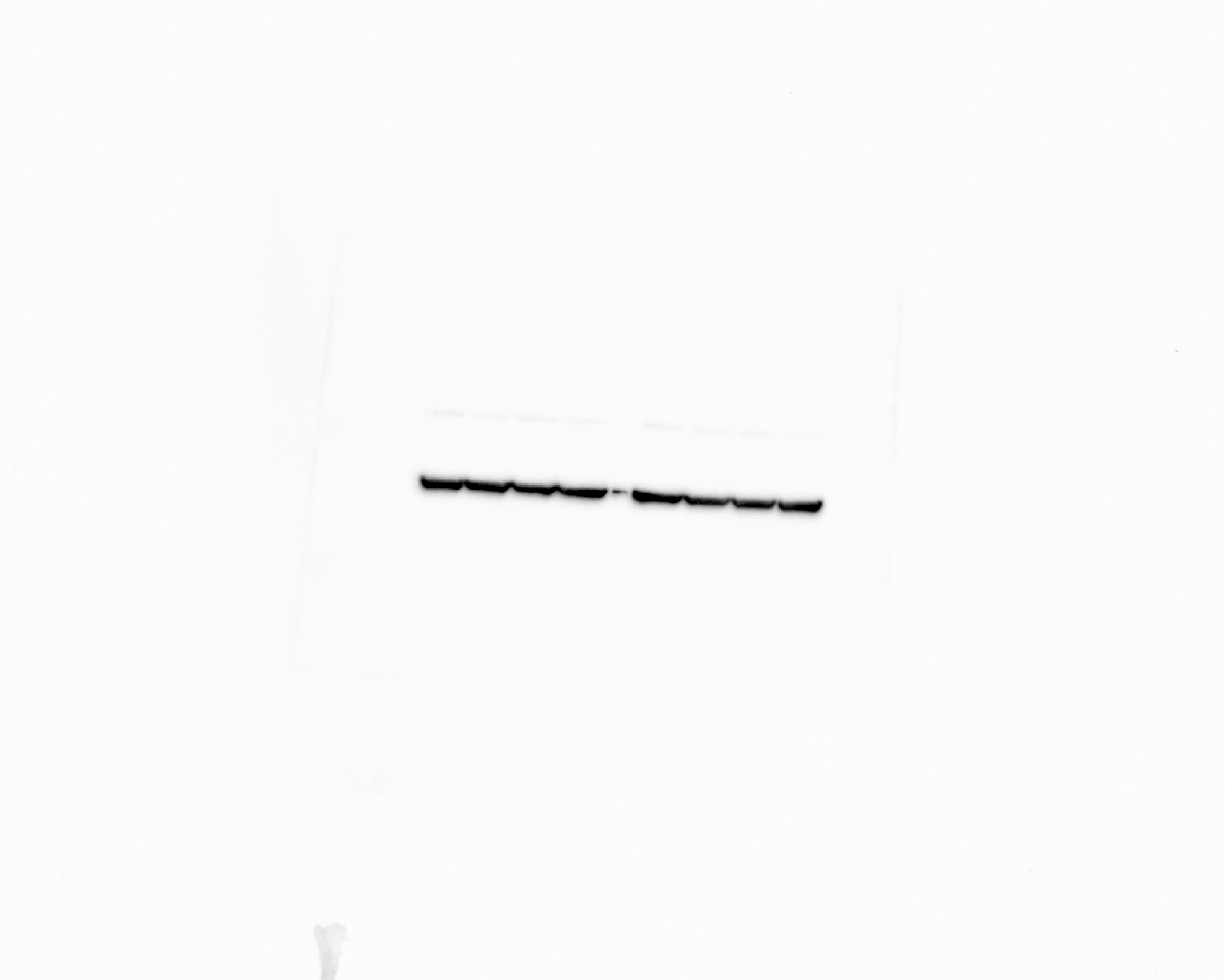

Supplement: Multimedia component 1 [file mmc1.zip › WB bands & raw densitometry/WB bands(45min)/1.(P-)AMPK/User 2025-09-17 B-actin(AMPK)(1)(Chemiluminescence).tif]

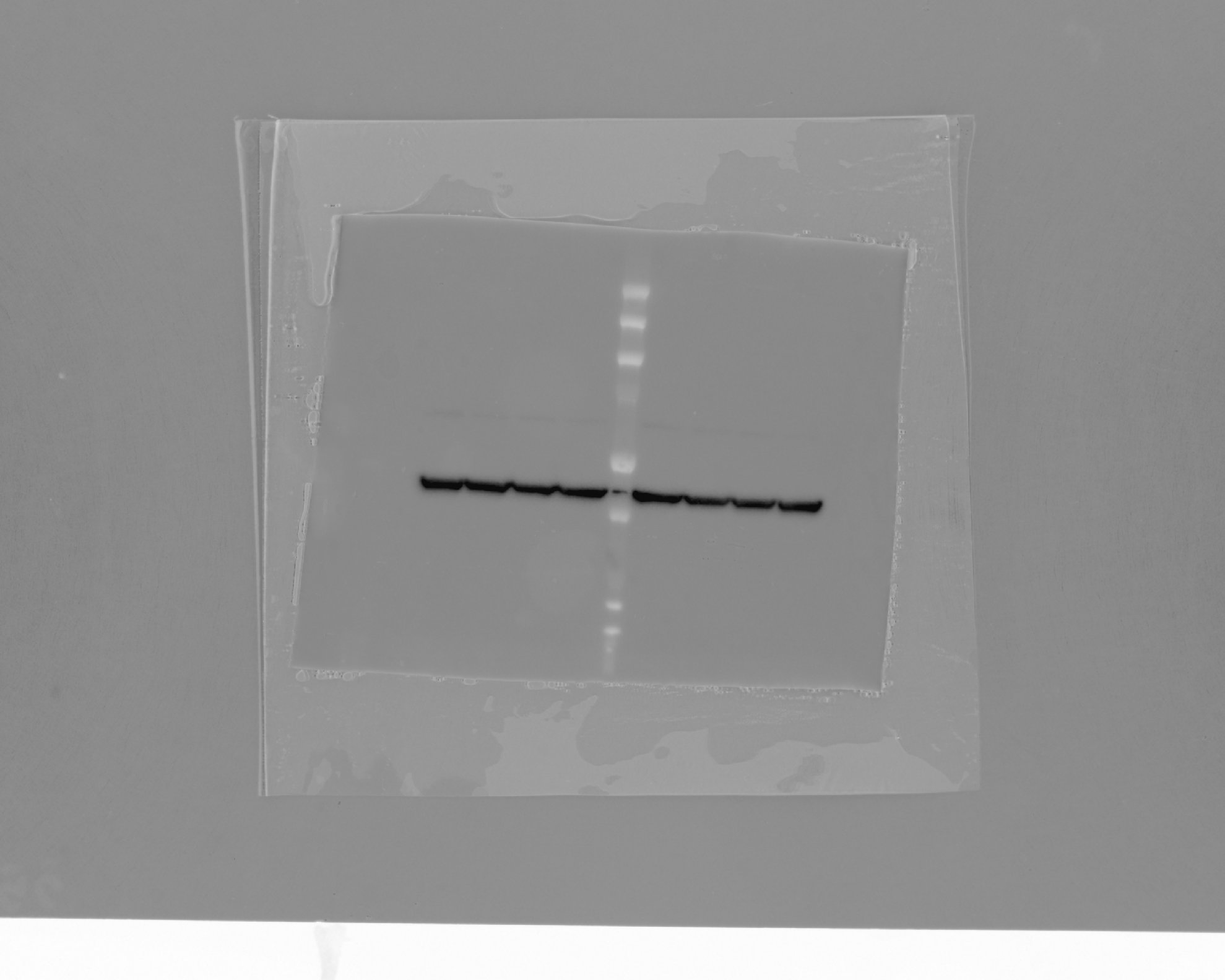

Supplement: Multimedia component 1 [file mmc1.zip › WB bands & raw densitometry/WB bands(45min)/1.(P-)AMPK/User 2025-09-17 B-actin(AMPK)(1)(Composite).tif]

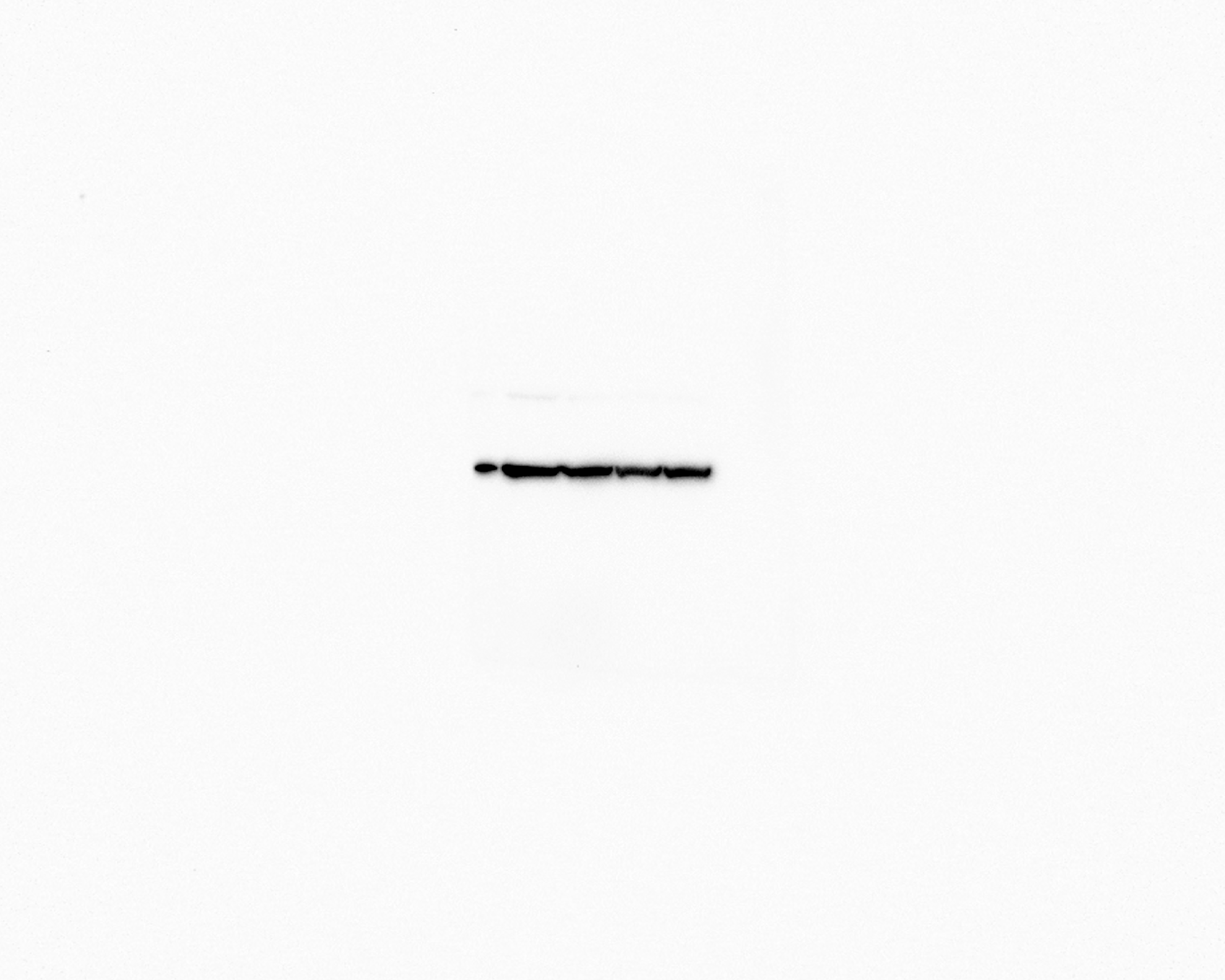

Supplement: Multimedia component 1 [file mmc1.zip › WB bands & raw densitometry/WB bands(45min)/1.(P-)AMPK/User 2025-09-17 B-actin(AMPK)(Chemiluminescence).tif]

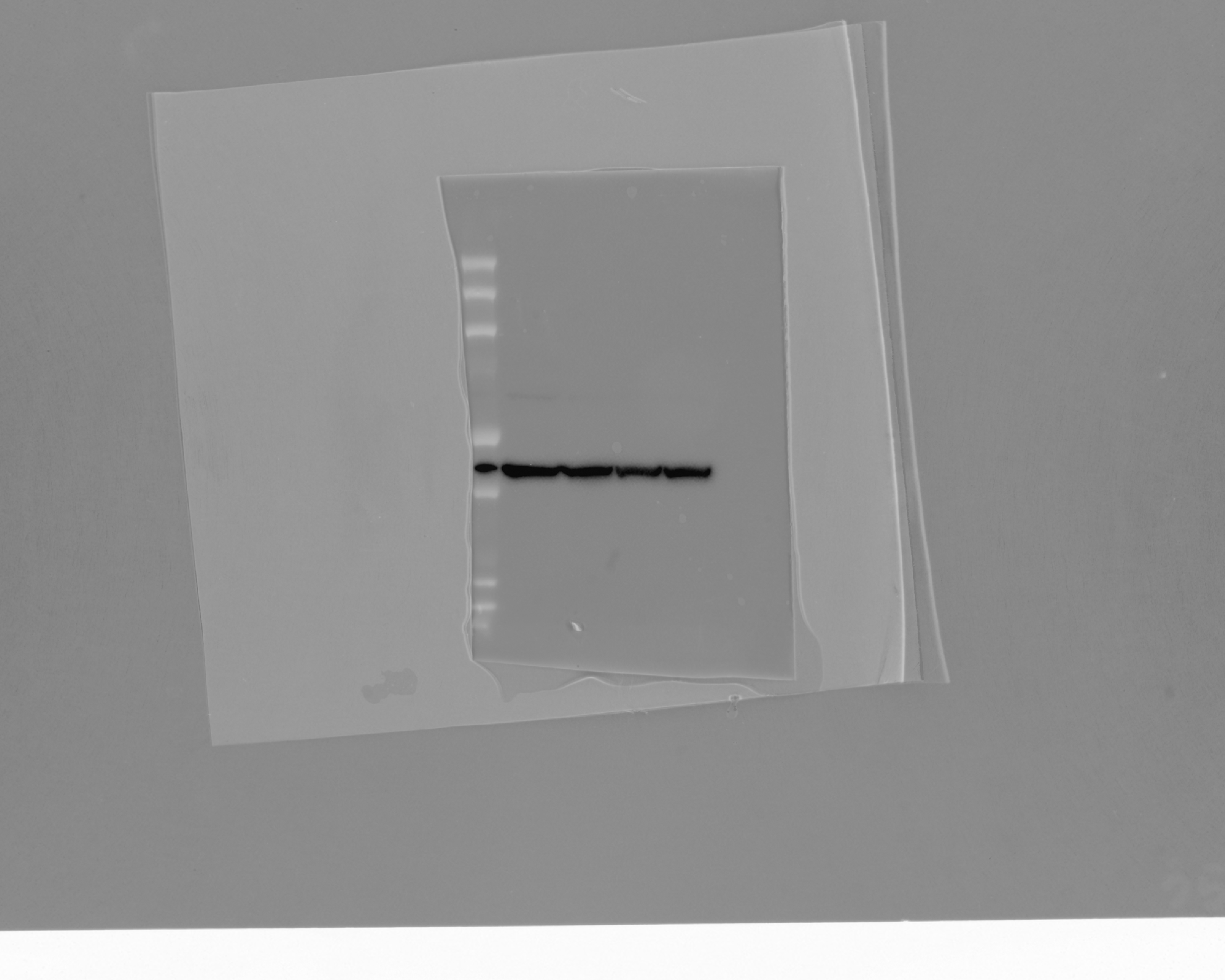

Supplement: Multimedia component 1 [file mmc1.zip › WB bands & raw densitometry/WB bands(45min)/1.(P-)AMPK/User 2025-09-17 B-actin(AMPK)(Composite).tif]

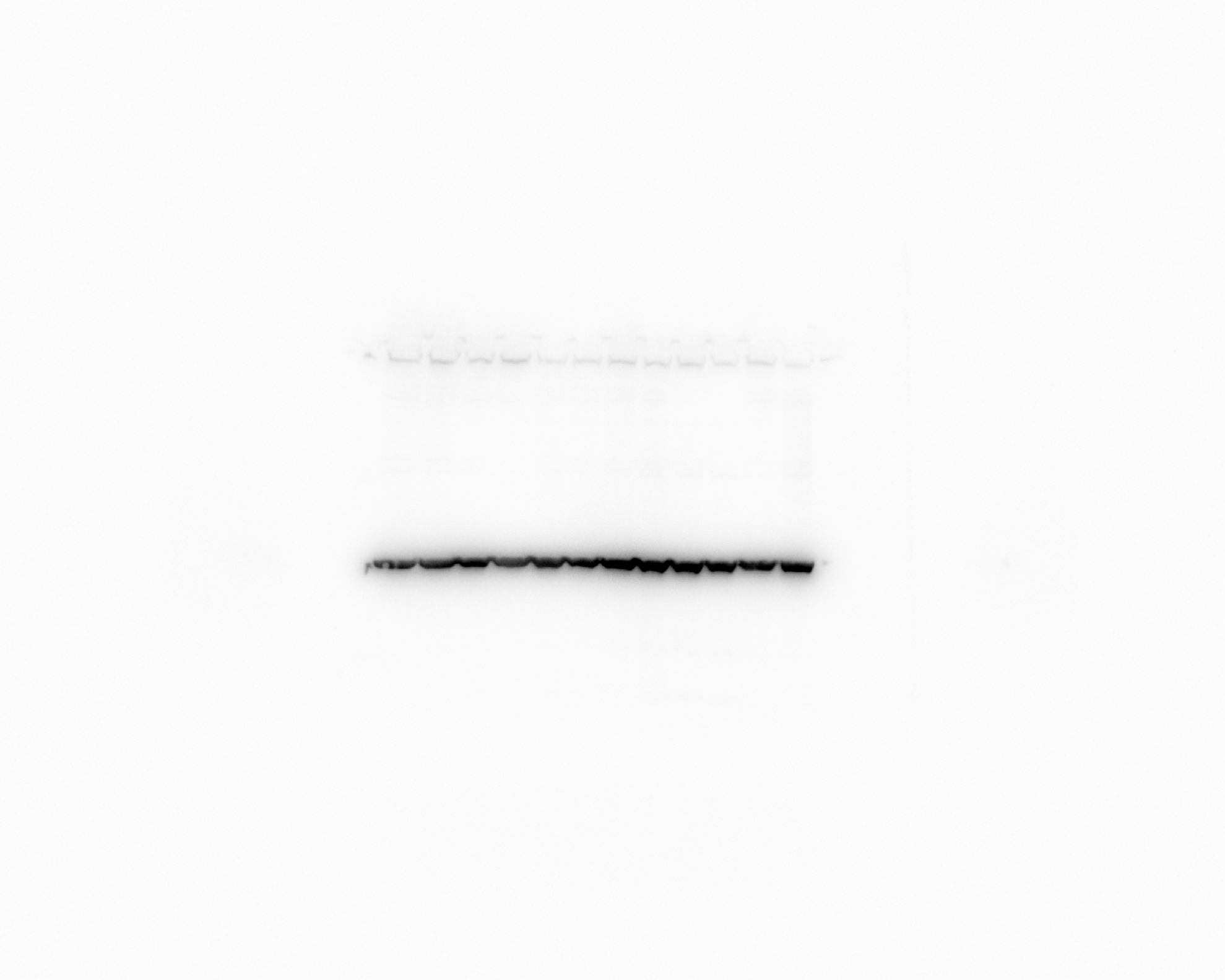

Supplement: Multimedia component 1 [file mmc1.zip › WB bands & raw densitometry/WB bands(45min)/10.(P-)mTOR/B-actin(mTOR)(Chemiluminescence).tif]

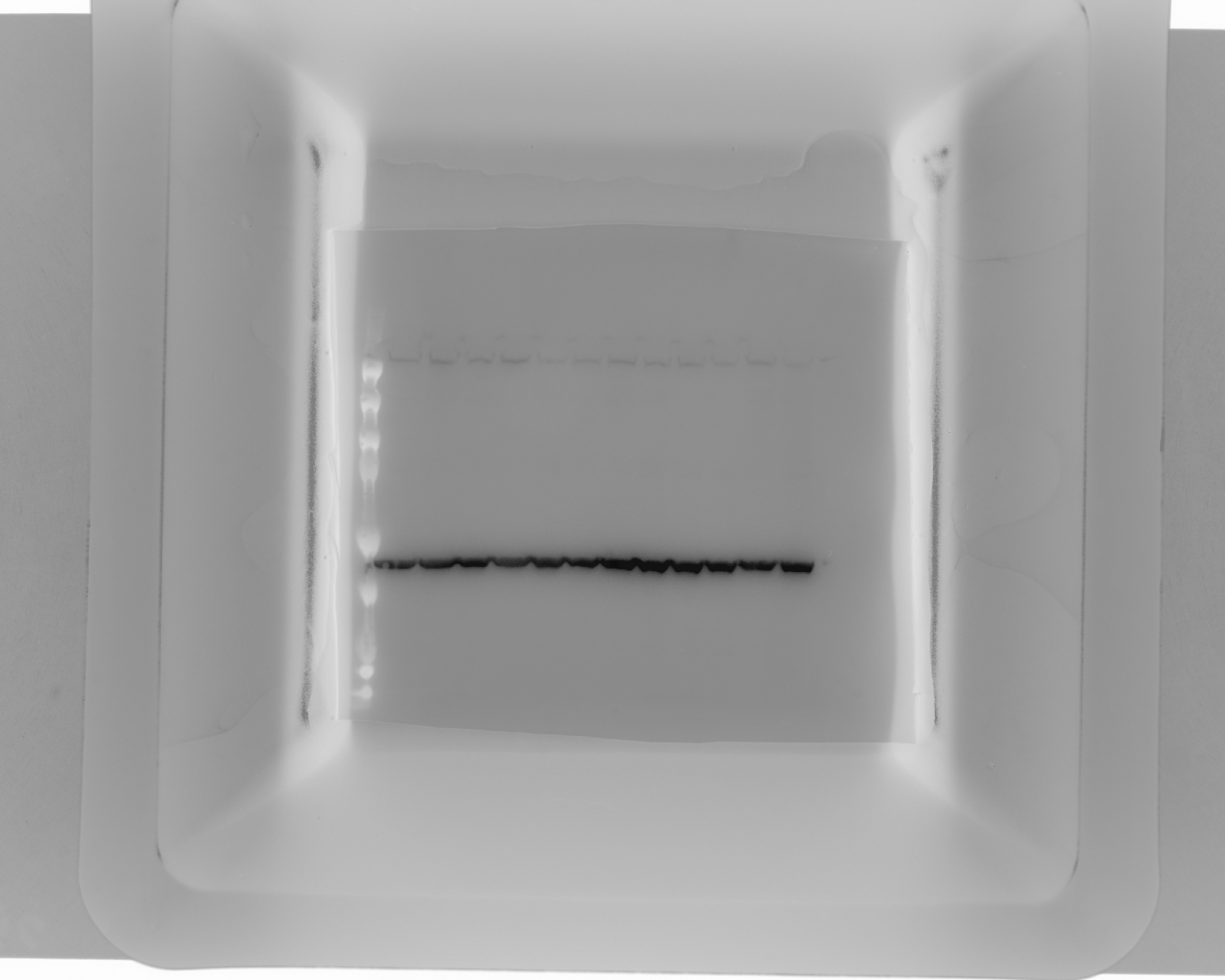

Supplement: Multimedia component 1 [file mmc1.zip › WB bands & raw densitometry/WB bands(45min)/10.(P-)mTOR/B-actin(mTOR)(Composite).tif]
